# Highly Nucleophilic Pyridinamide Anions in Apolar Organic Solvents due to Asymmetric Ion Pair Association

**DOI:** 10.1021/jacs.4c14825

**Published:** 2025-01-24

**Authors:** Veronika Burger, Maximilian Franta, Armin R. Ofial, Ruth M. Gschwind, Hendrik Zipse

**Affiliations:** † Department of Chemistry, Ludwig-Maximilians-Universität München, Butenandtstr. 5-13, 81377 München, Germany; ‡ Institute for Organic Chemistry, University Regensburg, Universitätsstr. 31, 93053 Regensburg, Germany

## Abstract

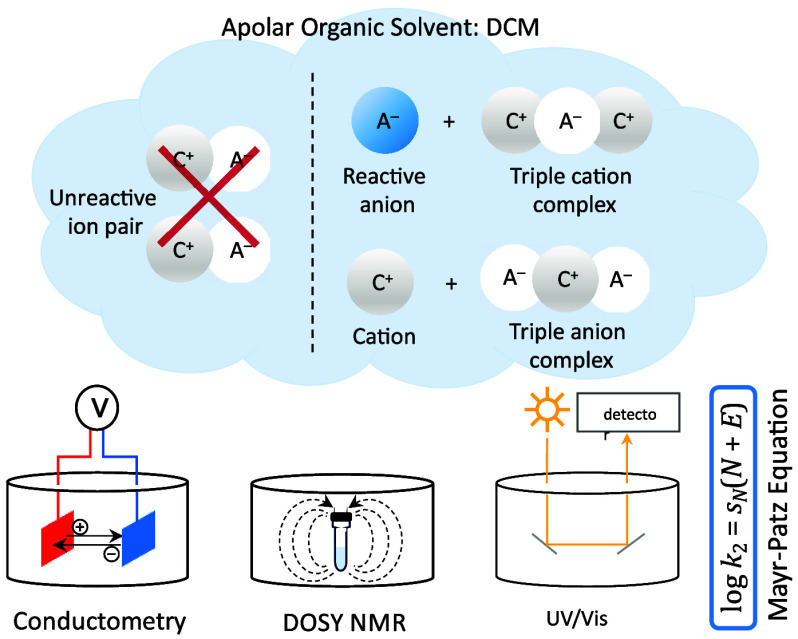

Free ions in organic solvents of low polarity would be valuable tools for the activation of low-reactivity substrates. However, the formation of unreactive ion pairs at concentrations relevant for synthesis has prevented the success of this concept so far. On the example of highly nucleophilic pyridinamide phosphonium salts in dichloromethane, we show that asymmetric aggregation offers a solution to this general problem. A combination of conductivity, diffusion-ordered NMR (DOSY), and kinetic measurements utilizing a refined ionic strength-controlled benzhydrylium ion methodology enables unique insight into the aggregation/association state of the ions and the nucleophilicity of the involved anions. This approach reveals that pyridinamide tetraphenylphosphonium salts aggregate in dichloromethane solution asymmetrically to form sandwich-type cations and anions together with their free counterions. The nucleophilicity of free pyridinamide ions exceeds that of the neutral reference nucleophile 9-azajulolidine (TCAP) by up to 2 orders of magnitude. Based on these results, we suggest that asymmetric aggregation in organic solvents of low polarity might be a general pathway to boost the reactivity of anionic nucleophiles.

## Introduction

**Chart 1:**
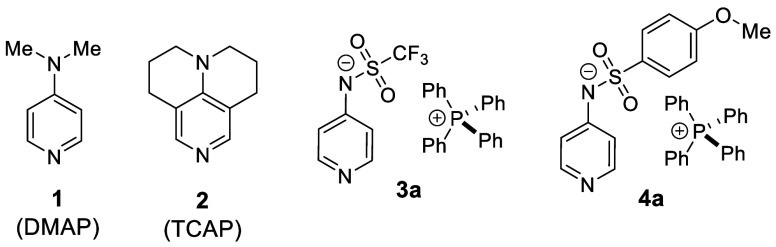
Structures of Neutral Organocatalysts DMAP (**1**) and TCAP (**2**) and of Pyridinamide Ion Pair Catalysts **3a** and **4a**

Lewis basic pyridines, such as 4-(dimethylamino)pyridine (DMAP, **1**)^1^ or the more reactive 9-azajulolidine (TCAP, **2**),^2^ are frequently used catalysts for group transfer reactions such as acylations,^3−6^ esterifications,^4,6^ alkylations,^7^ and silylations (Chart 1).^8,9^ The nucleophilicity of these catalysts, together with other donor-substituted pyridines, has been quantified using Mayr’s benzhydrylium ion method.^10−13^ Even higher nucleophilicities and possibly also higher catalytic activities in Lewis base-mediated reactions may be expected for anionic Lewis bases. Given that anionic reagents unavoidably require a countercation, such salts tend to form ion pairs when dissolved in organic solvents of low polarity, such as dichloromethane (DCM).^14,15^ This ion clustering has beneficially been used in ion pair catalysis,^16−18^ which extends from classical cationic phase-transfer (PT) catalysis^19^ to applications in asymmetric synthesis.^16,20−22^ Recently, the Zipse group introduced Lewis basic pyridinamide ion pair catalysts, which outperformed TCAP and other neutral organocatalysts in selected catalytic benchmark reactions.^23,24^

The pyridinamide phosphonium salts (such as **3a** and **4a** in Chart 1) investigated so far show the general usefulness of the concept of anionic nucleophilic organocatalysis, whose development tails that of neutral systems.^25−27^

In order to minimize ion pairing effects, most kinetic studies aiming at the quantification of the reactivity of anionic nucleophiles have been performed in highly polar solvents (water, DMSO, etc.), often in combination with crown ether additives to further reduce the interactions between metal counterions and the reacting anion.^28^ In solvents of low polarity, the intrinsic nucleophilicity of a free anion should be far higher than that in more polar media. However, the reactivity of anions is attenuated by ion pair formation, which also gives rise to nonlinear effects and, thus, complicates systematic kinetic studies in organic solvents of low polarity [dichloromethane (DCM), tetrahydrofuran (THF), toluene, etc.] commonly employed in organocatalysis.

In order to elucidate the underlying principles responsible for the experimentally observed high nucleophilicity of the anions in salts such as **3a** and **4a**, we report here a combination of conductivity measurements, diffusion-ordered NMR (DOSY) measurements at very low concentrations, and photometric kinetic measurements by utilizing an ionic strength-controlled benzhydrylium methodology. This combination of physicochemical methods is expected to be generally applicable to ion pair chemistry and catalysis and may help to uncover the full potential of this field.

## Results and Discussion

### Conductivity

Conductivity measurements have frequently been employed to quantify ion pairing effects.^29−32^ This method was therefore applied to determine the association of the cationic and anionic components of phosphonium salt **3a** selected here as a reference system in DCM and acetonitrile (MeCN). In both solvents, **3a** is expected to be more reactive toward electrophiles than DMAP (**1**). Conductivity measurements were performed for concentrations ranging from 0.02 to 1.0 mM, as this appears to represent the onset of ion pair formation from free ions. At low electrolyte concentrations and for the case of noninteracting ions, the experimentally determined conductivity κ depends on the specific molar conductivity Λ_m_ and the ion concentration [*A*], as expressed in eq 1.
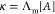
1

**Figure 1:**
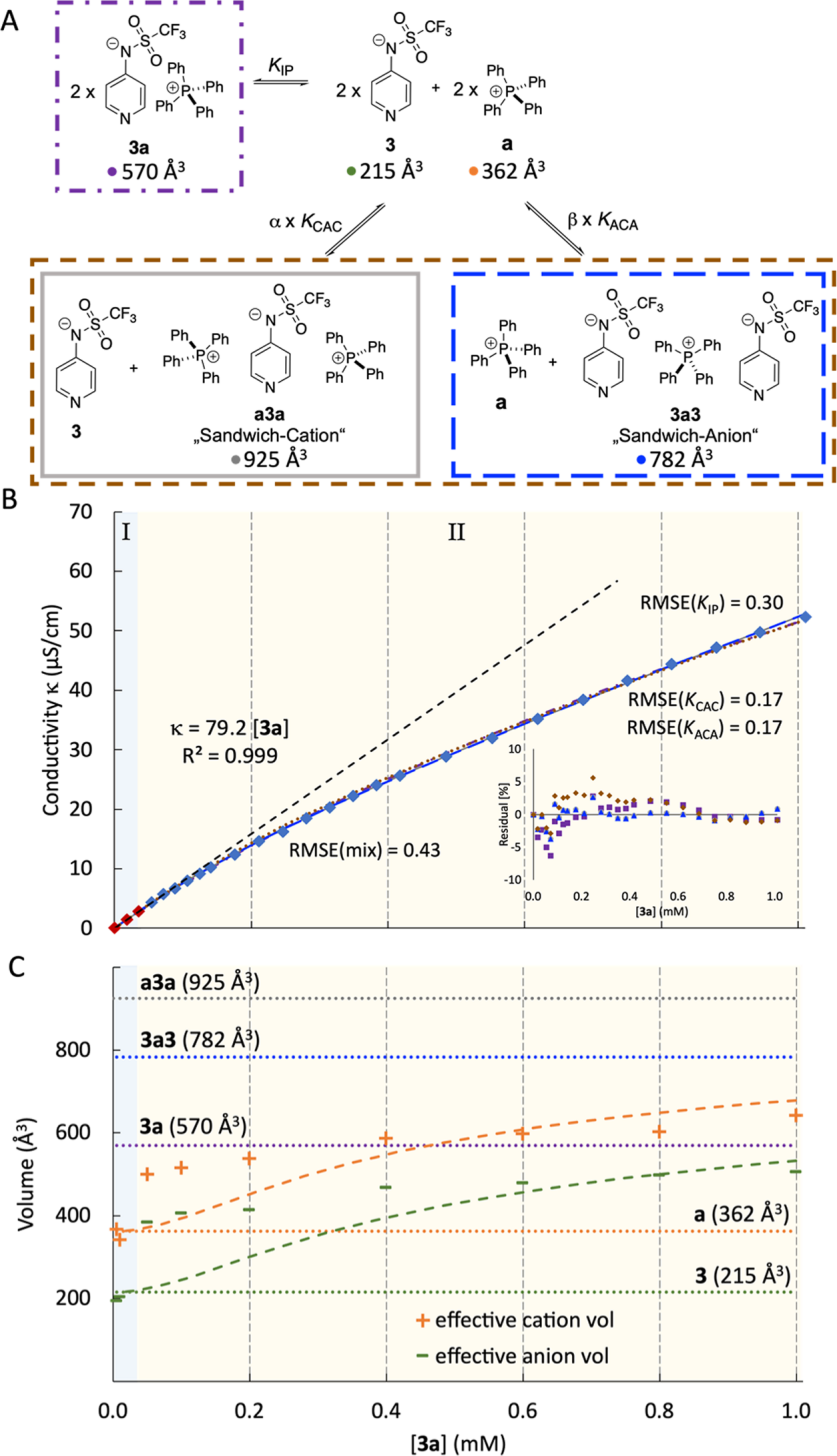
(A) 1:1 ion pair **3a**, sandwich cation **a3a**, and sandwich anion **3a3** as potential association models for anion **3** and cation **a** together with single-molecule dataset (SMD)-derived molecular volumes; (B) conductivity profile for **3a** in DCM fits to the calculated conductivity data for the 1:1 association model (purple dotted/dashed line), and the two sandwich association models (gray and blue line); (C) DOSY-derived ion volumes (in Å^3^) compared to SMD-derived volumes (dashed horizontal lines) for single anion **3**, single cation **a**, ion pair **3a**, cation sandwich **a3a**, and anion sandwich **3a3**.

In the polar aprotic solvent MeCN, the ions of **3a** are well stabilized and exist mainly as free ions, as indicated by a nearly perfect linear increase of conductivity with [**3a**] (see the SI, Figure S1). In the less polar solvent DCM, the situation is more complex, and two different domains can be seen in Figure 1B: (a) At low **3a** concentrations (region I, blue background, [**3a**] < 0.04 mM), the conductivity κ correlates linearly with [**3a**], and (b) a nonlinear part II at higher concentrations of **3a** (see Figure 1B beige background). While linear region I is assumed to represent the behavior of free anions (**3**) and cations (**a**), three ion association models were tested for nonlinear region II. The first corresponds to the formation of ion pair **3a** (purple box in Figure 1A), while the second model involves the formation of “sandwich cation” **a3a** together with free anion **3** (gray box in Figure 1A), and the third model considers the formation of an analogous sandwich anion **3a3** (blue box in Figure 1A). The latter model was originally proposed to account for the properties of tetraalkyl ammonium salts in apolar solution^33^ and subsequently employed for a variety of systems in organic solvents.^34−37^ In order to compare both models on equal footing, the respective equilibrium constants *K*_IP_ and *K*_CAC_ are defined relative to two equivalents, each of free cation **a** and free anion **3**.

The 1:1 ion pair model retains the specific molar conductivity Λ_m_ derived from the linear region I and adds the effects of reducing the number of conducting species through the formation of overall neutral (and thus inactive) ion pairs **3a**. Fitting this model to the observed conductivities up to an overall concentration of 1.0 mM yields ion pair formation constant *K*_IP_ = 6.86 × 10^5^ M^–2^ with good accuracy. The second model involves the formation of sandwich cation **a3a** together with one equivalent of free anion **3** (gray box in Figure 1A), again combined with the specific molar conductivity Λ_m_ value obtained from the linear region I. This model fits the observed conductivity values in the region up to 1.0 mM with a sandwich association constant *K*_CAC_ = 6.38 × 10^6^ M^–2^ with equally good accuracy. This is also true for the third model involving the formation of sandwich anion **3a3** (blue box in Figure 1A), for which the optimized association constant is *K*_ACA_ = 6.38 × 10^6^ M^–2^, numerically identical with *K*_CAC_. The measured conductivity values, together with the model predictions, are depicted in Figure 1B (gray line for the **a3a** sandwich model, blue line for the **3a3** sandwich model, and purple dotted line for the 1:1 ion pair), which illustrates that all models fit the experimental conductivity curve equally well, as indicated by largely similar RMSE values of RMSE(*K*_CAC_) = 0.17, RMSE(*K*_ACA_) = 0.17, and RMSE(*K*_IP_) = 0.30, respectively.

### DOSY-NMR

Since conductivity measurements alone cannot provide direct information on the size of the contributing ions, DOSY-NMR measurements of **3a** were performed in DCM-*d*_2_ for concentrations ranging from 0.005 to 1.0 mM (see Figure 1C and SI, Chapter 4 for detailed information). To enable DOSY measurements at these low concentrations, a 600 MHz spectrometer with a helium cryo probe and measurement times up to 16 h per sample were employed. The DOSY results were compared to calculated volumes of anion **3** (215 Å^3^), cation **a** (362 Å^3^), and contact ion pair **3a** (570 Å^3^), which are based on the van der Waals cavities employed in the SMD continuum solvation model at the SMD(DCM)/B3LYP-D3/6-31+G(d) level of theory and indicated through the horizontal dashed lines in Figure 1C. At [**3a**] = 0.005 mM as the lowest concentration accessible for DOSY measurements, we determined volumes of 367 Å^3^ for cation **a** and 192 Å^3^ for anion **3**, both of which agree closely with the SMD-derived volumes for cation **a** and anion **3**. At any concentration of **3a** > 0.005 mM, considerably larger cation and anion volumes were observed already in region I (for full data, see the SI, Chapter 4). In the 1:1 association model shown in the purple box in Figure 1A, the volumes of both species are expected to converge to the SMD-derived value of 570 Å^3^ for 1:1 ion pair **3a**. Instead, we persistently detected substantially different effective volumes for cation **a** and anion **3** also at higher concentrations (region II), and we also note that the DOSY-derived volume for the cationic species exceeds that calculated for the 1:1 ion pair **3a**.

This latter observation can be rationalized with the sandwich ion models, where the DOSY-derived cation volume is expected to approach that of the **a3a** sandwich cation of 925 Å^3^. Combining the SMD-derived molecular volumes of ions with the equilibrium constants obtained from conductivity measurements allows us to predict concentration-dependent effective cation and anion volumes. These are shown in Figure 1C as a green line for the anion and an orange line for the cation volumes. Comparing experimentally derived with theoretically predicted volumes shows these to coincide quite well between 0.4 and 1.0 mM for the cation sandwich model.^38^ In contrast, the 1:1 model predicts volumes for both ions, which are significantly lower than the experimental values (by more than 200 Å^3^ for the cation and >100 Å^3^ for the anion; see the SI). This is also true for the **3a3** anion sandwich model that predicts larger anion than cation volumes, not consistent with the DOSY experiments. The agreement between experimentally determined DOSY volumes and model predictions can be further improved by combining the two sandwich models considered here. This requires optimization of the two scaling factors α and β shown in Figure 1A, such that the agreement with the conductivity data and the DOSY volumes is optimized. The best agreement for ion pair **3a** is found for α = 0.44 and β = 0.21, which implies that 50% of the anion **3** is free while the other 50% is stored in the two sandwich complexes at *I* = 1.0 mM. In contrast, the DOSY experiments for **4a** show inverted relative ion volumes with larger values for the anionic species (see the SI, Chapter 4). This is reflected by the mixing coefficients for salt **4a** amounting to α = 0.12 and β = 0.61. This implies that 34% of anion **4** is free, and 66% is hidden in the two sandwich complexes. The performance of these “mixed sandwich” models is quite satisfactory in concentration region II but less so in region I with its rapid increase of ion volumes with salt concentration. It is an intriguing aspect of the formation of sandwich cation **a3a** that it generates one equivalent of free anion **3** as the counterion. The concentration of free anion **3** will quite obviously impact the efficiency of pyridinamide anion-based catalytic systems, where free anion **3** is expected to account for most of the observed activity.

### Kinetics

To characterize the nucleophilic reactivity of **3a** and **4a** in an organic solvent of low polarity, such as DCM, we refined Mayr’s well-known benzhydrylium ion method by implementing an ionic strength control. This enabled the characterization of free anionic nucleophiles for the first time in DCM. Mayr’s methodology has repeatedly demonstrated its utility to describe the reactivity of a wide range of carbon-, nitrogen, oxygen-, sulfur-, and phosphor-based nucleophiles in different solvents,^39^ including DMAP (**1**) and TCAP (**2**).^10,11,39^ In short, the benzhydrylium ion method involves the photometric monitoring of the reactions of colored benzhydrylium salts, such as **5a**–**c** (Table 1), whose electrophilic reactivities are characterized by the solvent-independent parameters *E*, with nucleophiles used in excess concentration to achieve kinetics under pseudo-first-order conditions. The first-order rate constants *k*_obs_ (s^–1^) can then be obtained by fitting a monoexponential decay function to the decreasing absorption of **5** during the reaction with **3**. The conductivity measurements (see the SI, Figure S1) showed that **3a** and **4a** fully dissociate into anions and cations when dissolved in MeCN. Accordingly, a linear increase of pseudo-first-order rate constants *k*_obs_ with nucleophile concentrations [**3**] (or [**3a**]) was observed in the kinetics of reactions of **3a** with **5** (eq 2).
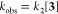
2
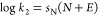
3

**Table 1:**
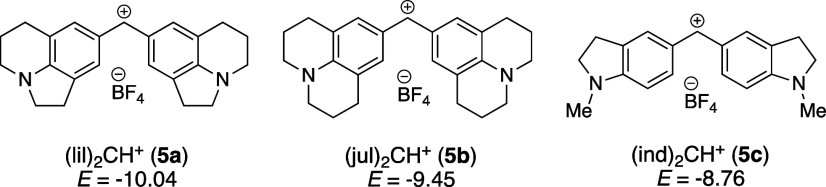
Second-Order Rate Constants *k*_2_ for the Reactions of DMAP (**1**), TCAP (**2**), and Pyridinamide Salts **3a** and **4a** with Reference Electrophiles **5a**, **5b**, and **5c** in MeCN (at 20 °C) Analyzed by Equation 3 to Give the Nucleophile-Specific Reactivity Parameters *N* (and *s*_N_)

	*k*_2_ [M^–1^ s^–1^]			
cat	**5a**	**5b**	**5c**	*N* (*s*_N_)
**1**^a^	2.11 × 10^3^	5.30 × 10^3^	1.29 × 10^4^	15.51 (0.62)^e^
**2**^b^	6.30 × 10^3^		4.17 × 10^4^	15.60 (0.68)^e^
**3**^c^	7.16 × 10^3^	1.53 × 10^4^	4.13 × 10^4^	16.38 (0.60)
**4**^d^	5.11 × 10^4^	1.36 × 10^5^	3.47 × 10^5^	17.28 (0.65)

a Second-order rate constants *k*_2_ from ref (10).

b Second-order rate constants *k*_2_ from ref (11).

c Assuming that [**3**] = [**3a**]_0_.

d Assuming that [**4**] = [**4a**]_0_.

e Additional *k*_2_ values from refs (10,11) were used to determine *N* (and *s*_N_).

Equation 2 thus yields second-order rate constants *k*_2_ (M^–1^ s^–1^) for the reactions of **3** with **5a**–**c** in acetonitrile (Table 1). The rate constants *k*_2_ for **3** are approximately three times larger than those for analogous reactions of **5** with DMAP (**1**) and quite similar to those for reactions with TCAP (**2**). Analyzing the kinetic data with the Mayr–Patz eq 3 yields the nucleophilicity *N* = 16.38 (*s*_N_ = 0.60) of **3** in MeCN. Following the same approach for **4a** yields *N*(**4a**) = 17.28 (*s*_N_ = 0.65), in excellent agreement with the results obtained for these two systems in selected catalytic transformations.^23^

**Figure 2:**
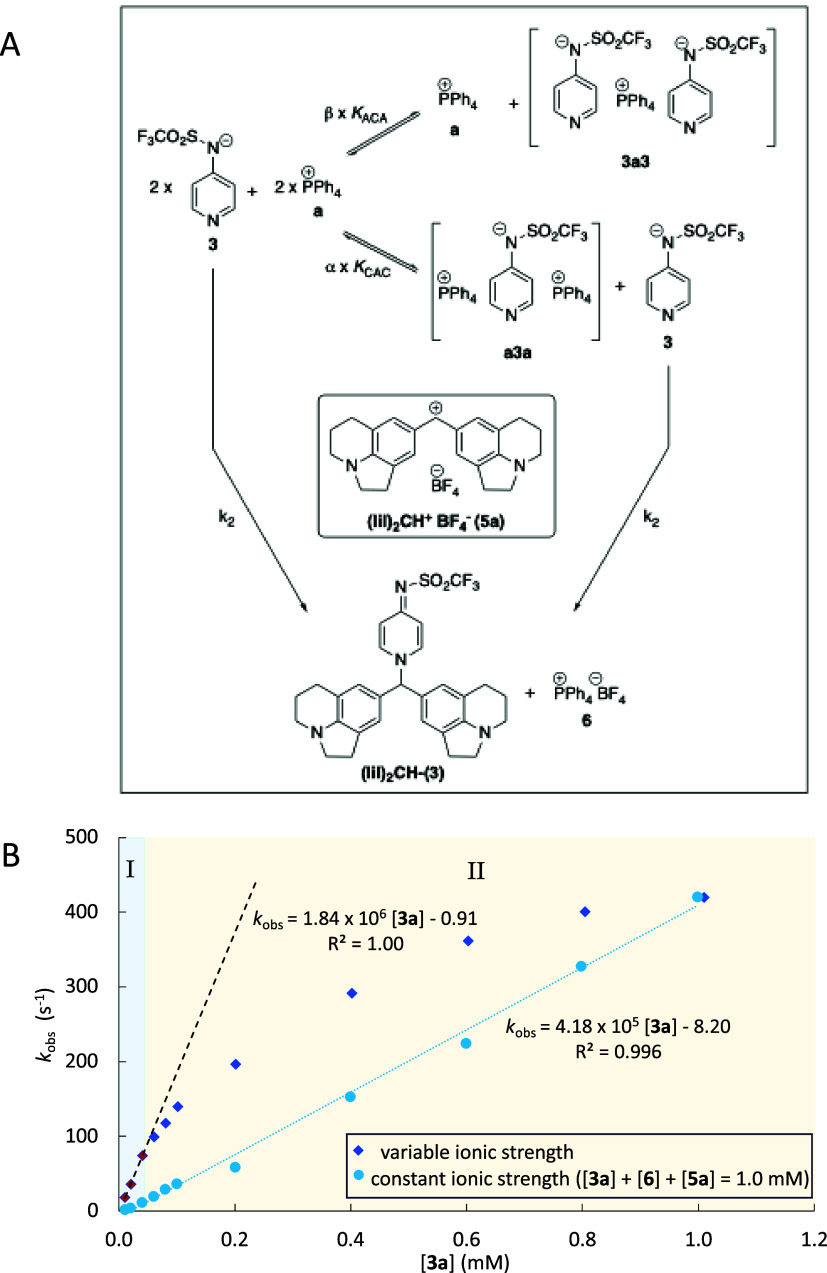
(A) Benzhydrylium ion reaction applied for the quantification of the nucleophilicity of **3**. (B) Correlation of *k*_obs_ for the reaction of **5a** with **3a** for salt concentrations [**3a**] from 0.01 to 1.0 mM in DCM at 20 °C (blue diamonds) and in the presence of additive PPh_4_BF_4_ (**6**) (turquoise dots).

The kinetics of reactions of **3a** with reference electrophiles **5** in DCM solution, however, showed a more complex dependence of *k*_obs_ on [**3a**] in the concentration range from 0.01 to 1.0 mM (red and blue diamonds in Figure 2). In analogy to the conductivity measurements, an initial region I with linear *k*_obs_ vs [**3a**]_0_ relation was observed (Figure 2, blue background, experimental values marked in red). At higher [**3a**] values, this is followed by nonlinear region II (blue diamonds on a beige background in Figure 2), where the observed rate constants deviate negatively from the linear correlation extrapolated from region I. The degree of deviation reflects the fraction of anion **3** captured in the (presumably) unreactive sandwich cation **a3a** and the (presumably) less reactive sandwich anion **3a3**.

Analysis of the kinetic data in region I (0.01–0.03 mM) is straightforward, as conductivity measurements in combination with the sandwich association models indicate almost complete (>97%) dissociation into separate ions **3** and **a**, that is, [**3**] = [**3a**]_0_. Application of eq 2 then yields *k*_2_(**5a**) = 1.84 × 10^6^ M^–1^ s^–1^, as indicated by the dashed line in Figure 2.

In synthetic applications, the concentration of ion pair catalysts is usually 1.0 mM or higher, which is far into the nonlinear region II. Increasing ion concentrations may impact the reaction rates not only through shifting the association equilibrium toward a higher fraction of ionic aggregates but also through nonspecific polarity effects. To assess the influence of the high salt concentration on the solvent polarity, we determined Reichardt’s *E*_T_(30) values in DCM solutions with increasing concentrations of pyridinamide salt **3a** and additive Ph_4_P^+^BF_4_^–^ (**6**). As shown in Figure 2, this additive combines the common unreactive ions in the reaction mixture, that is, the BF_4_^–^ of the benzhydrylium salts and the unreactive Ph_4_P^+^ countercation of the pyridinamide salts. We observed insignificant changes of the *E*_T_(30) values even at total salt concentrations of up to 6.0 mM (see the SI).

We conclude, therefore, that addition of **6** to a reaction mixture of **3a** and **5a**–**c** does not change the overall polarity of the solvent system and affects only the position of the ion pairing equilibrium shown in Figure 2, where higher concentrations of Ph_4_P^+^ (=**a**) give rise to an increase of [**a3a**]. To further investigate the effect of the Ph_4_P^+^BF_4_^–^ (**6**) additive, the ion volumes of selected **3a** + **6** mixtures were determined by DOSY measurements. The DOSY experiments show that the volumes of the cation and anion determined for **3a** + **6** mixtures at an ionic strength of *I* = 1.0 mM are in the same region as the volumes obtained for a pure **3a** solution at [**3a**] = 1.0 mM (for details, see the SI).^40^ The kinetics of the reaction of **3a** + **5a** was subsequently studied at a constant ionic strength (*I*) of *I* = 1.0 mM, as this represents the highest concentration of **3a** in this study. At [**3a**] < 1.0 mM, the ionic strength of the DCM solution was adjusted by addition of **6** such that in each kinetic measurement, the condition [**3a**] + [**6**] + [**5a**] = 1.0 mM is fulfilled. By maintaining *I* = 1.0 mM, the rate constants k_obs_ for **3a** + **5a** reactions in DCM correlated linearly with [**3a**] in the entire concentration range from 0.01 to 1.0 mM (turquoise points in Figure 2). When we account for the fact that variable fractions of anion **3** are caught in unreactive sandwich cation **a3a** (and to a smaller extent also in anion sandwich **3a3**) and also consider the effect of additive **6** on [Ph_4_P^+^], we obtain *k*_2_(**5a**) = 5.42 × 10^5^ M^–1^ s^–1^ for the reaction of **3** with **5a**, which is by a factor of 3.5 lower than *k*_2_ obtained in the low-concentration (LC) region (Table 2). Analogous kinetic measurements at I = 1.0 mM were performed for reactions of **3a** with more reactive benzhydryl salts **5b** and **5c** (Table 2).

**Table 2:** Second-Order Rate Constants *k*_2_ of the Reactions of DMAP (**1**), TCAP (**2**), and Pyridinamide Salt **3a** with Reference Electrophiles **5a**, **5b**, and **5c** in DCM (at 20 °C)

	*k*_2_ [M^–1^ s^–1^]		
cat	**5a**	**5b**	**5c**
**1**^a^	6.45 × 10^3^	9.84 × 10^3^	4.96 × 10^4^
**2**^b^	1.42 × 10^4^	3.11 × 10^4^	1.28 × 10^5^
**3**^c^	1.84 × 10^6^ (LC)	4.23 × 10^6^ (LC)	1.98 × 10^7^ (LC)
**3**^d^	5.42 × 10^5^ (mix)	1.25 × 10^6^ (mix)	4.64 × 10^6^ (mix)
**4**^e^	1.69 × 10^6^ (mix)	4.19 × 10^6^ (mix)	1.15 × 10^7^ (mix)

a Second-order rate constants *k*_2_ from ref (10a).

b This work, see the Supporting Information for details of the kinetic experiments.

c Determined at [**3**] < 0.03 mM, that is, in the low-concentration (LC) region I (Figure 2), by assuming [**3**] = [**3a**]_0_.

d Determined over a concentration range [**3a**] = 0.1 to 0.3 mM at constant ionic strength *I* = 1.0 mM (kept by addition of salt **6**) by assuming a mixed sandwich association model (see the SI for details).

e Determined over a concentration range [**4a**] = 0.04 to 0.1 mM at constant ionic strength *I* = 1.0 mM (kept by addition of salt **6**) by assuming a mixed sandwich association model.

In DCM as the solvent, we note a moderate increase in the bimolecular rate constants *k*_2_ when going from DMAP (**1**) to TCAP (**2**) but a significantly larger increase of the *k*_2_ values for **3a** (Table 2). The *k*_2_ values for the reaction of **3a** with **5a**–**c** in DCM in the low-concentration (LC) region I (as defined in Figure 2) exceed those for **2** by approximately 2 orders of magnitude. Analyzing the LC kinetic data by the Mayr–Patz equation in eq 3 gives *N* = 17.78 (*s*_N_ = 0.81) for **3a**. Rate constants *k*_2_ for reactions of **3** with all three benzhydryl cations **5a**–**c** decrease slightly (by a factor of 3.5–4.5) under conditions of constant ion strength (*I* = 1.0 mM). The resulting *N*-parameter for **3** is, however, hardly changed at *N* = 17.88 (*s*_N_ = 0.73). Following the same mode of analysis for **4a** under reaction conditions where *I* = 1.0 mM, we find that anion **4** exceeds the nucleophilicity of **3** by a factor of 3.0 ± 0.5 in its reaction with benzhydryl cations **5a**–**c**, which is also reflected in the respective nucleophilicity parameter of *N*(**4**) = 19.63 (*s*_N_ = 0.65).

**Figure 3:**
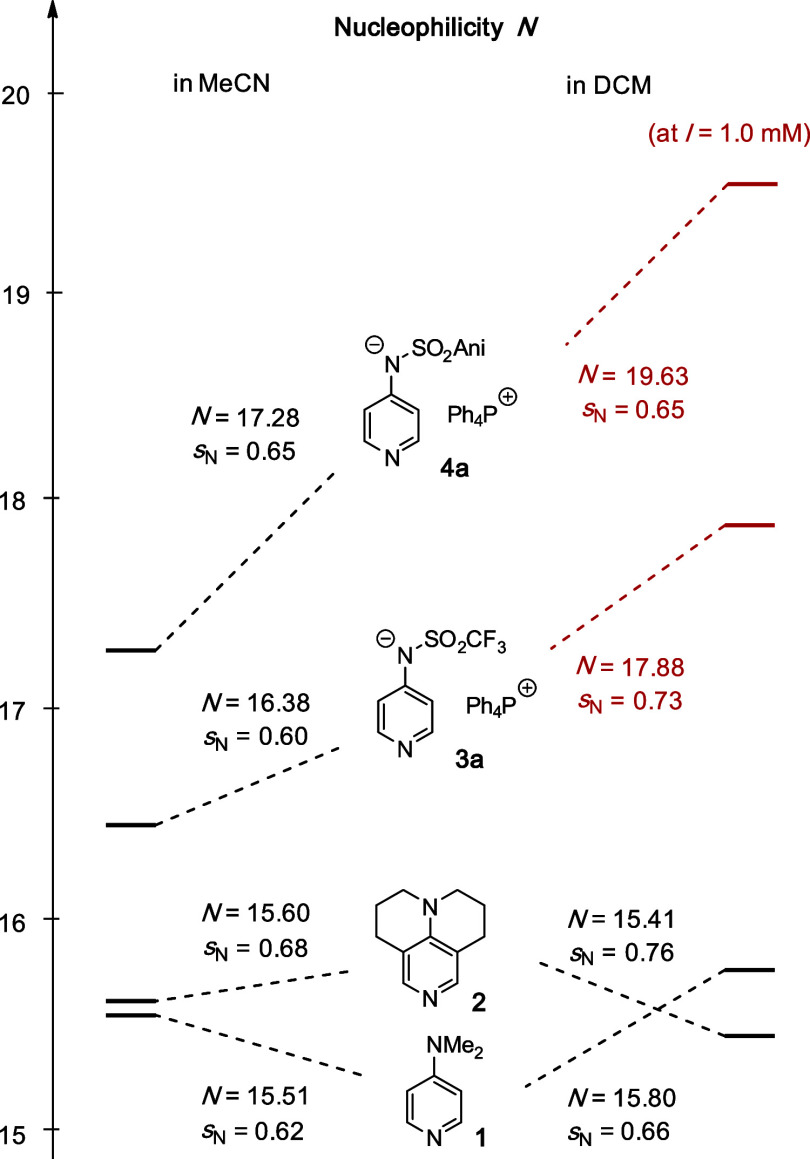
Mayr nucleophilicities *N* (and *s*_N_) of DMAP (**1**), TCAP (**2**), and pyridinamide anions **3** and **4** (Ani = *p*-methoxyphenyl) in MeCN and DCM.

These measurements thus establish **3a** and **4a** as potent and highly nucleophilic pyridine derivatives in solvents of low polarity (Figure 3). That **4a** is more nucleophilic than **3a** is in full agreement with the results for selected organocatalytic transformations performed in CDCl_3_ as the solvent.^23^ The combined methodology developed here thus allows for a quantitative assessment of catalyst nucleophilicity at synthetically relevant concentrations.

## Conclusions

Pyridinamide salts **3a** and **4a** exceed the nucleophilic reactivity of the highly reactive neutral Lewis base TCAP (**2**) by up to 2 orders of magnitude in DCM. Employing a combination of conductivity and DOSY measurements, we have deciphered an asymmetric association behavior of both pyridinamide ion pairs in the low polarity solvent DCM, which includes both cationic and anionic sandwich complexes but not the commonly assumed and unreactive 1:1 ion pair. Without the combination of conductivity and DOSY measurements, this result could not have been achieved since conductivity alone does not give insight into the type of charged species that are being measured. The reactivity of the supernucleophilic anions **3** and **4** was quantified with the newly developed ionic strength-controlled benzhydrylium ion method, which facilitates the comparison of **3a** and **4a** with neutral nucleophilic catalysts, such as DMAP or TCAP. In DCM, we were able to evaluate kinetic data not only at low salt concentrations but also at synthetically relevant higher concentrations by keeping the ionic strength constant throughout the measurement to prevent the interference of ion association. The direct comparison of *k*_2_ values for reactions with cationic reference electrophiles reveals reactivity values of pyridinamide anions **3** and **4** (at high concentration) that are 39 and 90 times higher than that of TCAP (**2**). The superior reactivity of pyridinamide anions **3** and **4** has recently been observed in catalytic reactions with isocyanates and Michael acceptors as electrophiles.^23,24^ This indicates that the higher nucleophilicity of **3** and **4** in comparison to neutral nucleophilic catalysts might be comparably effective for reactions with neutral electrophiles in low polarity media (alkanes, THF, and Et_2_O). The asymmetric ion association described here opens the general avenue for employing highly reactive free anions to activate so far inaccessible substrates in catalytic transformations.

## Supporting Information Available

Additional experimental and computational details, analysis procedures, and methods, including step by step descriptions (PDF)
