# Supplementary material for: Highly Nucleophilic Pyridinamide Anions in Apolar Organic Solvents due to Asymmetric Ion Pair Association
Source: J Am Chem Soc. 2025 Jan 24;147(6):5043–50. doi: 10.1021/jacs.4c14825 (PMC11826998; doi:10.1021/jacs.4c14825)
Supplement: Supplementary file 1 — ja4c14825_si_001.pdf [file ja4c14825_si_001.pdf]

Supporting Information

Highly Nucleophilic Pyridinamide Anions in Apolar Organic Solvents due to Asymmetric Ion Pair Association

Veronika Burger[a], Maximilian Franta[b], Armin R. Ofial*[a], Ruth M. Gschwind*[b], and Hendrik Zipse[*a]

[a]Department of Chemistry, Ludwig-Maximilians-Universität München, Butenandtstr. 5-13, 81377 München, Germany.

[b]Institute for Organic Chemistry, University Regensburg, Universitätsstr. 31, 93053 Regensburg, Germany.

TABLE OF CONTENTS

1. Additional Figures and Correlations 4

2. Synthesis of Compounds 5

3. Conductometric Measurements 7
3.1 Sample Preparation and Data Acquirement 7
3.2 Data analysis – 1:1 Association Model 7
3.3 Double Ion Pair Association Model 10
3.4 Sandwich Association Model 10
3.5 Mixed Sandwich Association (Model 4) 16
3.6 Workflow Summary 22
3.7 Conductivity Data in MeCN 23
3.8 Conductivity Data in DCM 26

4. DOSY NMR Spectroscopy 30

5. Characterization of Additive PPh_4BF_4 (6) 44

6. Nucleophilicity Data 47
6.1 Mayr's Benzhydrylium Method – Results in MeCN and DCM at *c* = 0.01–0.03 mM 47
6.2 Cationic Sandwich Association Extension 51
6.3 Mixed Sandwich Association Extension 53
6.4 Nucleophilicity Measurement Data 58
6.4.1 Nucleophilicity of TCAP in DCM at 20 °C 58
6.4.2 Nucleophilicity of 3a in MeCN at 20 °C 60
6.4.3 Nucleophilicity of 4a in MeCN at 20 °C 61
6.4.4 Nucleophilicity of 3a in DCM at 20 °C 62
6.4.5 Nucleophilicity of 3a in DCM at *I* = 1.0 mM at 20 °C (with 1:1 Model for Additive 6) 63
6.4.6 Nucleophilicity of 3a in DCM at *I* = 1.0 mM at 20 °C (with Cationic Sandwich Model for Additive 6) 64
6.4.7 Nucleophilicity of 3a in DCM at *I* = 1.0 mM at 20 °C (with Mixed Sandwich Model Extension) 65
6.4.8 Nucleophilicity of 4a in DCM at *I* = 1.0 mM at 20 °C (with Cationic Sandwich Model for Additive 6) 66
6.4.9 Nucleophilicity of 4a in DCM at *I* = 1.0 mM at 20 °C (with Mixed Sandwich Model Extension) 67

7. Kinetics of Wide Range Measurement 68
7.1 Wide Range Data at 20°C 68
7.2 Wide Range Data of 3a at *I* = 1.0 mM at 20 °C (with 1:1 Model for Additive 6) 70
7.3 Wide Range Data at *I* = 1.0 mM at 20 °C (with Cationic Sandwich Model for Additive 6) 70
7.4 Wide Range Data at *I* = 1.0 mM at 20 °C (with Mixed Sandwich Model Extension) 71

8. Crystallographic Data 73

9. NMR Spectra of Newly Synthesized Compounds 75

10. DOSY Fits 79

11. Computational Study 97
11.1 System 3a – Computational Information 98
11.2 System 4a – Computational Information 103
11.3 System PPh_4BF_4 (6) – Computational Information 106
11.4 Sandwich Systems – Computational Information 108
11.5 XYZ-Coordinates of Most Stable Compounds 110

References 128

General Information

All reagents were purchased from Sigma Aldrich, TCI, or Acros and used without further purification unless otherwise noted. Solvents were obtained from Acros Organics, Sigma Aldrich, or Merck and purified by simple distillation in a rotary evaporator, unless otherwise specified.

All air- and moisture-sensitive reactions were performed under a nitrogen atmosphere and the glassware and magnetic stirrers were dried in a dry oven at 110 °C overnight.

CH_2Cl_2 for nucleophilicity measurements was stirred over concentrated H_2SO_4 for two weeks before extraction with water (1 x 1.0 L), $NaHCO_3$ (1 x 1.0 L), and again water (1 x 1.0 L). CaH_2 was added as a drying agent, and the solvent was freshly distilled over CaH_2 prior to use.

Melting points were acquired using Büchi Melting Point M-560 devices and are uncorrected.

Nuclear magnetic resonance (NMR) spectra were recorded on a Bruker 400 MHz or INOVA 400 and 600 MHz machines. The following abbreviations were used in the analysis of NMR spectra: s = singlet, d = doublet, t = triplet, q = quartet, m = multiplet, br s = broad singlet. NMR signals were assigned based on 2D spectra (COSY, HSQC, HMBC, NOESY) experiment analysis. Chemical shifts are given in ppm. The internal reference was set to the residual solvent signals (CD_2Cl_2, $CDCl_3$, DMSO-d_6). The ^{13}C NMR spectra (101 or 151 MHz) were recorded under broadband proton-decoupling. ^{19}F NMR spectra were referenced using the solvent signal.[1] The spectra were imported and processed in the program MestreNova (version 14.1.1).

Infrared (IR) spectra were measured on a Perkin Elmer Spectrum BX-59343 instrument with a Smith Detection DuraSamplIR II Diamond ATR sensor for liquids or neat for solids. Intensities are described as vs = very strong, m = medium, w = weak, br = broad.

High-resolution mass spectra (HRMS) were recorded on a Thermo Finnigan LTQ FT Ultra Fourier Transform Ion Cyclotron Resonance mass spectrometer with electrospray ionization (ESI) for sample ionization. For EI (70 eV) measurements a Thermo Finnigan of the MAT 95 type with a direct exposure probe (DEP) was used.

Crystal structures were recorded using an Oxford Diffraction XCalibur with Sapphire CCD-detector and a molybdenum-K_α-source (λ = 0.71073) with a concentric circle kappa-device. The structures were resolved using the program SHELXS or SIR97 and refined with SHELXS.

1. Additional Figures and Correlations

Details on data evaluation of conductivity data can be found in Chapter 3.

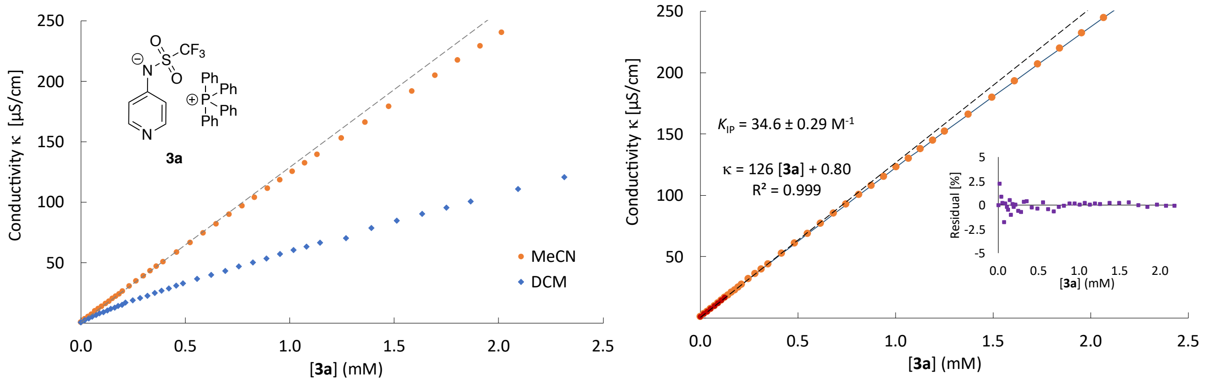

Figure S1. Left: conductivity of **3a** in MeCN (orange points) and in DCM (blue points) at 20 °C with extrapolated conductivity profile of fully dissociated salt (dotted grey line); right: conductivity of **3a** in MeCN (orange points) and fit (black line) of the experimental data according to the 1:1 association model at 20 °C with extrapolated conductivity profile of fully dissociated salt (dotted grey line). Conductivity measurements were performed for concentrations ranging from 0.02 – 2.5 mM but only values in the range 0.02 to 1.0 mM are interpreted as 1.0 mM appears to represent the threshold onset of ion pair formation from the free ions.

The data displayed in Figure S1 in the manuscript can be found in Table S1.

Table S1. List of measured conductivity values (without solvent background conductivity of 0.8 μS cm^{-1}), DOSY measurement results (given as the hydrodynamic volume) and kinetic results for ion pair **3a** in DCM.

Conductivity		DOSY NMR			Wide Range[b]	
[3a] (mM)	κ (μS/cm)	[3a] (mM)	Vol Anion (Å³)	Vol Cation (Å³)	[3a] (mM)	k_{obs} (s^{-1})
0.00	0.00	–	–	–	–	–
–	–	0.005	193	367	–	–
–	–	0.01	203	342	0.01	18.0
0.02	1.44	–	–	–	0.02	36.4
0.04	2.72	–	–	–	0.04	74.1
0.05	4.32	0.05	383	500	0.06	99.2
0.07	5.76	–	–	–	0.08	118
0.09	6.80	0.10	405	515	0.10	140
0.11	8.00	–	–	–	–	–
0.12	9.12	–	–	–	–	–
0.14	10.2	–	–	–	–	–
0.18	12.4	–	–	–	–	–
0.21	14.6	0.20	413	537	0.20	197
0.25	16.2	–	–	–	–	–
0.28	18.4	–	–	–	–	–
0.31	20.3	–	–	–	–	–
0.35	22.2	–	–	–	–	–
0.38	24.0	–	–	–	–	–
0.42	25.6	0.40	466	587	0.40	292
0.48	28.8	–	–	–	–	–
0.55	32.0	–	–	–	–	–
0.62	35.2	0.60	477	598	0.60	362
0.68	38.4	–	–	–	–	–
0.75	41.6	–	–	–	–	–
0.82	44.3	0.80	497	603	0.80	400
0.88	47.2	–	–	–	–	–
0.94	49.8	–	–	–	–	–
1.01	52.3	1.00	505[a]	642[a]	1.01	420

[a] averaged value based on entry 9-12 in Table S16, Chapter 4; [b] "wide range" is the description of kinetic measurements performed according to the Mayr's benzhydrylium method over a concentration range from c = 0.01 – 1.0 mM.

2. Synthesis of Compounds

1,1,1-Trifluoro-*N*-(pyridin-4(1*H*)-ylidene)methanesulfonamide

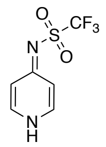

4-Aminopyridine (2.37 g, 25.2 mmol, 1.0 eq) was dissolved in dry pyridine (30.0 mL) under nitrogen atmosphere. Triethylamine (8.00 mL, 57.4 mmol, 2.3 eq) was added and stirred for 10 min. The reaction mixture was cooled to 0 °C and trifluoromethanesulfonyl chloride (5.00 g, 29.7 mmol, 1.2 eq) was added. After stirring for 10 min at 0 °C, the mixture was refluxed for 3.5 h under nitrogen atmosphere before the solvent was removed. The crude product was suspended in H_2O, refluxed for 20 min and filtered while hot. This process was repeated with acetone and MTBE, yielding pyridinamide **3** (3.55 g, 15.7 mmol, 62%) as a light brown solid.

^{1}H NMR (400 MHz, DMSO-*d6*): δ [ppm] = 13.60 (s, 1H), 8.28 (d, *J* = 7.3 Hz, 2H), 7.27 (d, *J* = 7.3 Hz, 2H).

^{13}C NMR (101 MHz, DMSO-*d6*): δ [ppm] = 163.7, 140.2, 120.7 (q, *J* = 325.6 Hz), 116.9.

^{19}F NMR (377 MHz, DMSO-*d6*): δ [ppm] = -77.69.

HRMS (ESI): calc. for $C_6H_5F_3N_2O_2S^+$ [M$^+$]: 226.0018, found 226.0019.

4-Methoxy-*N*-(pyridin-4(1*H*)-ylidene)benzenesulfonamide

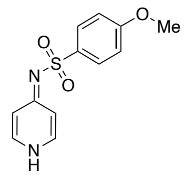

4-Aminopyridine (2.03 g, 21.6 mmol, 1.0 eq) and NEt_3 (8.50 mL, 61.0 mmol, 2.8 eq) were dissolved in pyridine (22 mL). The mixture was cooled to 0 °C and 4-methoxybenzenesulfonyl chloride (5.35 g, 25.9 mmol, 1.2 eq) was added and stirred for 20 min. The reaction mixture was refluxed for 3 h before being cooled down to rt. The solvent was removed, and the resulting precipitate was repeatedly refluxed in H_2O, acetone, and MTBE. The final product was dried *in vacuo*. Pyridinamide **4** (4.78 g, 18.1 mmol, 84%) was obtained as an off-white solid.

^{1}H NMR (400 MHz, DMSO-*d6*): δ [ppm] = 12.1 (s, 1H), 8.04 (d, *J* = 6.5 Hz, 2H), 7.74 (d, *J* = 8.9 Hz, 1H), 7.03 (d, *J* = 8.9 Hz, 1H), 6.95 – 6.89 (m, 2H), 3.79 (s, 3H).

^{13}C NMR (101 MHz, DMSO-*d6*): δ [ppm] = 161.7, 151.1, 141.7, 134.4, 128.3, 114.1, 113.8, 55.5.

HRMS (ESI): calc. for $C_{12}H_{12}N_2O_3S^+$ [M$^+$]: 264.0563, found 264.0567.

Tetraphenylphosphonium pyridin-4-yl((trifluoromethyl)sulfonyl)amide (**3a**)

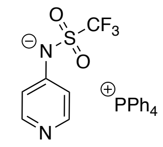

3a

Pyridinamide **3** (402 mg, 1.78 mmol, 1.0 eq) was added to a solution of NaOH (78.2 mg, 1.96 mmol, 1.1 eq) in H_2O (5.0 mL). Tetraphenyl bromide (746 mg, 1.78 mmol, 1.0 eq) was added, and the reaction mixture was stirred at rt. for 1 h. DCM (5.0 mL) was added, and the two phases were separated. The aqueous phase was extracted with DCM (3 x 15 mL). The collected organic phase was dried over $MgSO_4$, filtered and the solvent was evaporated. The crude product was crystallized from DCM overlayered with toluene. Pyridinamide ion pair **3a** (787 mg, 1.39 mmol, 78%) was obtained in form of colorless needles.

^{1}H NMR (400 MHz CDCl$_3$): δ [ppm] = 8.05 – 8.00 (m, 2H), 7.88 – 7.81 (m, 4H), 7.71 (td, *J* = 7.9, 3.6 Hz, 4H), 7.55 (ddd, *J* = 13.0, 8.4, 1.3 Hz, 4H), 7.02 – 6.97 (m, 2H).

^{13}C NMR (101 MHz, CDCl$_3$): δ [ppm] = 156.1, 149.5, 135.9 (d, *J* = 3.1 Hz), 134.5 (d, *J* = 10.3 Hz), 130.9 (d, *J* = 12.9 Hz), 122.2 (q, *J* = 328.6 Hz), 118.0 (d, *J* = 6.4 Hz), 117.1.

^{19}F NMR (377 MHz, CDCl$_3$): δ [ppm] = -76.8.

^{31}P NMR (162 MHz, CDCl$_3$): δ [ppm] = 23.19.

IR (ATR): ν (cm^{-1}) = 3060 (br), 1588 (m), 1484 (w), 1438 (m), 1317 (s), 1288 (s), 1198 (vs), 1155 (s), 1108 (m), 1000 (m), 832 (w), 723 (m).

Elemental Analysis: $C_{30}H_{24}F_3N_2O_2PS$ (564.56 g/mol): calc. (%) C, 63.82; H, 4.29; N, 4.96; S, 5.68; Found (%): C, 63.79; H, 4.32; N, 5.00; S, 5.45.

HRMS (ESI): calc. for $C_6H_4F_3N_2O_2S^-$ [A⁻]: 224.9951; found 224.9948; calc. for $C_{24}H_{20}P^+$ [C⁺]: 339.1297; found: 339.1289.

m. p: 183-185 °C.

Tetraphenylphosphonium ((4-methoxyphenyl)sulfonyl)(pyridin-4-yl)amide (**4a**)

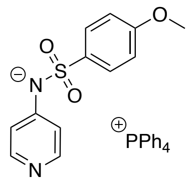

4a

Pyridinamide **4** (501 mg, 1.90 mmol, 1.0 eq) was added to a solution of NaOH (78.9 mg, 1.97 mmol, 1.1 eq) in H_2O (5.0 mL). Tetraphenylphosphonium bromide (795 mg, 1.90 mmol, 1.0 eq) was dissolved in DCM and added dropwise over 30 min through a dropping funnel. The reaction mixture was stirred at rt. for 2 h. More DCM (5.0 mL) was added, and the two phases were separated. The aqueous phase was extracted with DCM (3 x 25 mL). The collected organic phase was dried over $MgSO_4$, filtered and the solvent was evaporated. The crude product was crystallized from DCM overlayered with toluene and heptane (0.5 mL) while stored in the fridge. Ion Pair **4a** (782 mg, 1.30 mmol, 69%) was obtained in form of colorless crystals.

^{1}H NMR (600 MHz, $CDCl_3$): δ [ppm] = 7.90 – 7.79 (m, 1H), 7.77 – 7.69 (m, 1H), 7.61 – 7.53 (m, 1H), 6.79 – 6.72 (m, 4H), 3.74 (s, 3H).

^{13}C NMR (151 MHz, $CDCl_3$): δ [ppm] = 160.5, 157.9, 148.8, 138.8, 138.8, 135.9 (d, *J* = 3.1 Hz), 134.5 (d, *J* = 10.3 Hz), 130.9 (d, *J* = 12.9 Hz), 128.7, 117.5 (d, *J* = 89.5 Hz), 116.3, 113.2, 55.4.

^{31}P NMR (162 MHz, $CDCl_3$): δ [ppm] = 23.03.

Elemental Analysis: $C_{36}H_{31}N_2O_3PS$ (602.69 g/mol): calc. (%) C, 71.74; H, 5.18; N, 4.65; S, 5.32; Found (%): C, 71.35; H, 5.24; N, 4.62; S, 5.77.

Tetraphenylphosphonium tetrafluoroborate (**6**)

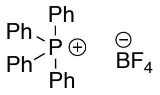

6

Tetraphenylphosphonium bromide (1.05 g, 2.50 mmol, 1.0 eq) was dissolved in water and sodium tetrafluoroborate (276 mg, 2.50 mmol, 1.0 eq) was added in portions. The resulting cloudy suspension was stirred for 2 h at rt. DCM (10 mL) was added, and the two phases were separated. The aqueous phase was extracted with DCM (3 x 20 mL). The collected organic phase was dried over $MgSO_4$, filtered and the solvent was evaporated. The crude product was dissolved in DCM and precipitated by adding toluene. Product **6** (940 mg, 2.21 mmol, 88%) was obtained as colorless needles.

^{1}H NMR (400 MHz CD_2Cl_2): δ [ppm] = 7.98 – 7.87 (m, 4 H), 7.81 – 7.72 (m, 8 H), 7.66 – 7.56 (m, 8 H).

^{13}C NMR (101 MHz, CD_2Cl_2): δ [ppm] = 36.2 (d, *J* = 3.3 Hz), 134.8 (d, *J* = 10.4 Hz), 131.0 (d, *J* = 13.0 Hz).

^{19}F NMR (377 MHz, CD_2Cl_2): δ [ppm] = -153.43.

^{31}P NMR (162 MHz, CD_2Cl_2): δ [ppm] = 23.12.

IR (ATR): ν (cm^{-1}) = 1586 (w), 1436 (m), 1108 (m), 1052 (s), 750 (w), 689 (s).

Elemental Analysis: $C_{24}H_{20}BF_4P$ (426.20 g/mol): calc. (%) C, 67.64; H, 4.73; Found (%): C, 67.58; H, 4.62.

HRMS (ESI): calc. for BF_4^- [A⁻]: 87.0035; found: 87.0034; calc. for $C_{24}H_{20}P^+$ [C⁺]: 339.1297; found: 339.1292.

m. p: 350-352 °C.

3. Conductometric Measurements

3.1 Sample Preparation and Data Acquirement

For the measurement, an up to 0.02 M solution of the respective catalyst is prepared in a 10 mL volumetric flask and is given portion-wise to the pure solvent in a measuring cell at 20 °C. Conductivity measurements were done using a WTW conductometer with a Pt electrode LTA 1/NS in MeCN and DCM. Calibration was done as described in ref. [2]. Temperature control (20.0 ± 0.1 °C) was achieved by using a circulating bath cryostat. The conductivity is measured in volts [V] and depicted in "ME-REDLab Data Acquisition V1.1", developed by Dr. B. Kempf 2010, where the conductivity is plotted against time. After adding a portion of salt stock solution, one must wait until the conductivity value reaches a stable plateau (min 30 sec up to 100 sec) from which the average conductivity value is read off. In Excel this value [V] is then converted into [µS/cm] by multiplying it with the cell parameter of the used electrode (z = 160). The cell parameter was obtained by calibrating the conductometric set up with aqueous KCl solutions (0.007 M, 0.0145 M, and 0.0375 M; commercial conductivity standard solutions purchased from Alfa Aesar) referring to the conductivity of 1273 µS/cm for an 0.01 M solution of aq. KCl at 20 °C.[3]

3.2 Data analysis – 1:1 Association Model

The conductivity of a substance is defined as its ability to conduct electricity and depends on the number of charge carriers in solution. Therefore, it can be expressed as a molar quantity where κ is the measured conductivity, Λ_m is the molar conductivity and c is the measured concentration of electrolyte.

$$\kappa = \Lambda_m c \tag{S1}$$

Analysis of the conductivity profile shown in Figure S2 can most easily be approached assuming the formation of an 1:1 ion pair system with concentration [IP] from diffusively free anions A and cations C. For this situation the association constant K_{IP} is given by eq. S2.

$$K_{IP} = \frac{[IP]}{[A][C]} \tag{S2}$$

The combination of eq. S2 with eq. S3 for the ion pair concentration yields eq. S4, which relates the concentration of free ions [A] to the total salt concentration $[IP]_{tot}$ and the association constant K_{IP}.

$$[IP] = [IP]_{tot} - [A] \tag{S3}$$

$$[A] = [C] = \frac{-1 + \sqrt{1 + 4K_{IP}[IP]_{tot}}}{2K_{IP}} \tag{S4}$$

The experimentally determined conductivity κ depends on the specific molar conductivity Λ_m and the ion concentration [A] as expressed in eq. S5, whose combination with eq. S4 the combination of which then yields eq. S6.

$$\kappa = \Lambda_m[A] \tag{S5}$$

$$\kappa = \Lambda_m \times \left\{\frac{-1 + \sqrt{1 + 4K_{IP}[IP]_{tot}}}{2K_{IP}}\right\} + BG \tag{S6}$$

The value for the specific molar conductivity Λ_m for the combined ions of salt **3a** is derived from the extrapolated linear regression line of the red-marked conductivity values (first three points). The background conductivity (BG) included here is the conductivity of the used solvent (BG = 0.8 µS cm^{-1}).

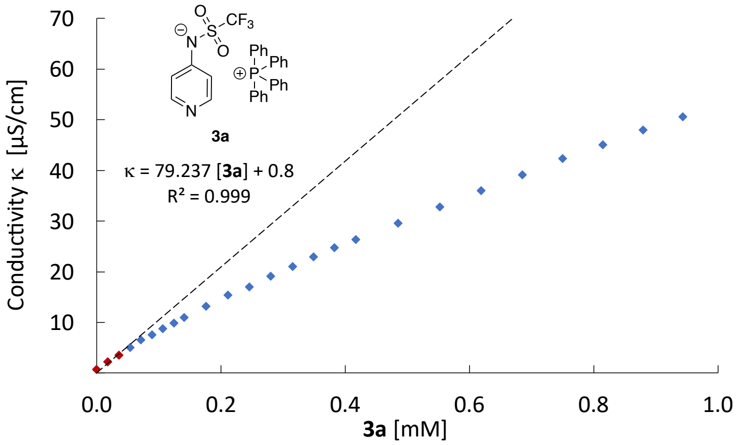

Figure S2. Concentration-dependent conductivity profile for catalyst **3a** in DCM with Λ_m = 79.2 S cm^2 mol^{-1} and solvent background BG = 0.8 µS cm^{-1}at 20 °C.

Eq. S6 was used in the fitting program "ProFit7" (version 7.0.18) to obtain the ion pairing constant K_{IP} from the conductivity profiles measured in DCM and MeCN. The specific molar conductivity Λ_m was extrapolated using the first three data points in DCM and the first eight data points in MeCN. The results are collected in Table S2.

Table S2. Association constants K_{IP} of ion catalyst **3a** in MeCN and DCM according to eq. S6 at 20 °C.

	K_{IP} (M^{-1})	
**Ion Pair**	**MeCN** [45.6][a]	**DCM** [40.7][a]
**3a**	34.6 ± 0.3	828 ± 8.27

[a] $E_T(30)$ [kcal mol^{-1}] solvent polarity parameters taken from Reichardt[4].

In a second approach the complex pathway simulation program, COPASI[5], was used to numerically analyze the obtained conductivity data. After starting COPASI, a biochemical model was chosen and the association of a single anion **A** reacting with a single cation **C** into the ion pair **IP** was added as reaction (see Scheme S1).

$$A \quad + \quad C \quad \xrightleftharpoons{K_{IP}} \quad IP$$

Scheme S1. Equation for numerical simulations of 1:1 ion association for salt **3a**.

The reaction was set to be reversible, which is also indicated by the equal sign "=" in the reaction equation. In the "Symbol Definition" section the starting compound (A + C) is listed with rate constant k_1 as well as the product (IP) with rate constant k_2, defining the ion pairing constant as $k_1/k_2 = K_{IP}$.

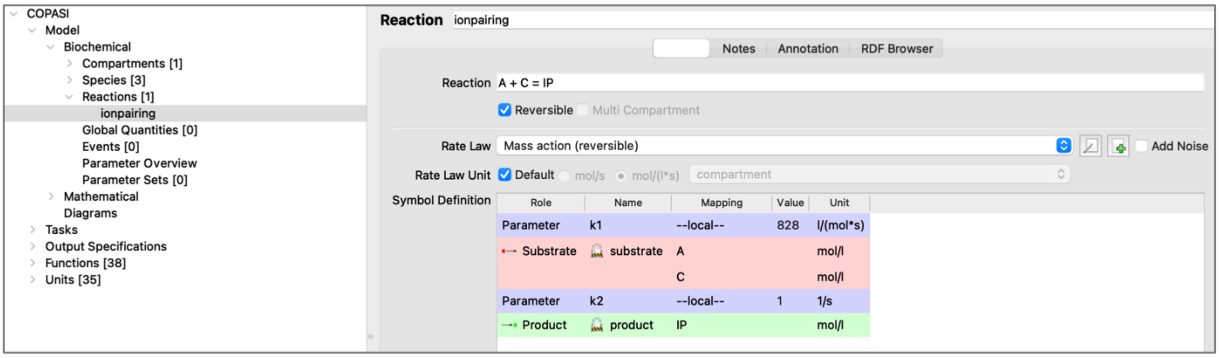

To find the ion pairing constant K_{IP} that best fits our experimental data in the section "Reactions" the reaction constant k_2 was set to be 1, while the rate constant k_1 was changed until the best fit between the simulated and experimental conductivity curve was found.

In the section "Species" the individual concentration for all compounds was added. This would be zero for the 1:1 ion pair **IP** and the first measured total salt concentration of the anion **A** and for the cation **C** (in this example 1.78 x 10^{-5} M).

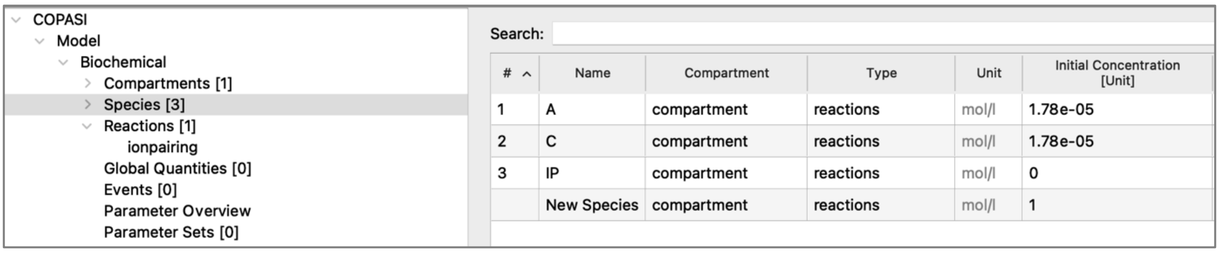

Next, we moved on to the "Tasks" menu and selected the "Steady-State" option there. Clicking the "Run" button at the bottom of the window, COPASI ran through a steady state analysis and then reports the results as follows:

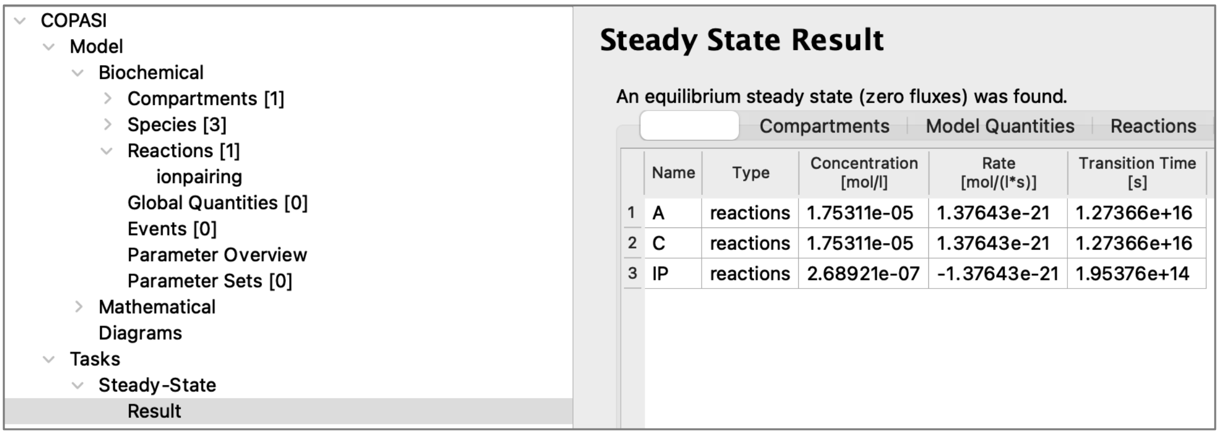

The obtained values for [**A**], [**C**], and [**IP**] were copied into an Excel analysis sheet, that contains all relevant data. The obtained concentrations for those three species were then converted into conductivity values according to eq. S5. Therefore, the solvent conductivity background of 0.8 µS cm^{-1} was subtracted from the measured conductivity values. The molar conductivity Λ_m can be divided into the limited molar conductivity λ_i of each single ionic species *i* in solution according to eq. S7.

$$\Lambda_m = \lambda_A + \lambda_C \qquad \text{(S7)}$$

Since the concentration of both ionic species **A** and **C** is always identical in this association model, the individual conductivity contributions are not of critical importance. For now, we assumed that the single anion **A** and the single cation **C** contribute equally to the total molar conductivity Λ_m, that was extracted from region I of the conductivity profile. The 1:1 ion pair **IP** was assigned zero contribution to the molar conductivity Λ_m. The conductivity value of each species is summed up and the final value is compared to the experimental value. This step is repeated until the simulated and experimental conductivity value are close to identical.

This procedure can be tremendously sped up by the use of a Phyton script that automatically performs the "steady state" function for any given numbers of concentrations.[6] The "root mean square error" (RMSE) is kept as a control measure and k_1 is optimized until the minimum RMSE for the chosen concentration range is found (which, in some cases, includes up to 5 decimal places). With the assistance of the Python script, we can examine all measured conductivity values right from the start to find the association constant K_{IP}.

The 1:1 association model is the simplest model that can be applied and is usually the first model that is tested for the analysis of the association of a newly designed salt.

By comparing the solutions for the association constant K_{IP} found with the integrated method and the numerical simulations performed with COPASI no difference could be observed (see Figure S3).

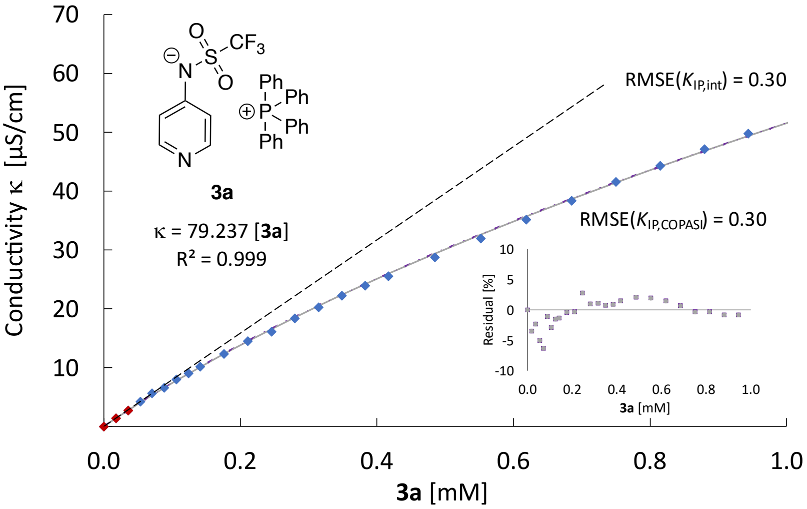

Figure S3. Comparing results of integrated method (purple dotted-dashed line) and numerical simulation according to 1:1 ion association model (grey line) for conductivity data of salt **3a**.

The RMSE values do not differentiate form one another as well as the percentage residue between the experimental data and simulated conductivity values. Therefore, we conclude that the two methods can be used interchangeably for the determination of the ion association constant K_{IP}.

3.3 Double Ion Pair Association Model

DOSY measurements revealed a deviation from the previously assumed 1:1 association model. Instead, we presume an aggregate formation where two cation with one anion form a triple cation complex, leaving one anion free. Here, four charged ions associate into two charged species. To compare this "sandwich" association type with the classical 1:1 model on equal footing, the existing model was modified to involve four ions combining into two separate charge-neutral ion pairs (see Scheme S2).

$$2 * A + 2 * C \xrightleftharpoons{K_{IP}} 2 * IP$$

Scheme S2. Equation for numerical simulations of double 1:1 ion association for salt **3a**.

The above detailed procedure was followed to determine and optimize the ion pairing constant K_{IP}. The final result was found to be $K_{IP} = 6.86 \times 10^5\ M^{-2}$ for pyridinamide ion pair **3a** in DCM.

3.4 Sandwich Association Model

With DOSY measurements, it was determined that instead of the previously assumed simple 1:1 association of anion and cation, a "sandwich cation" aggregate form first. This new insight necessitates a reconsideration of the previously applied association model. Since a mathematical description of this new association model has not yet been achieved, the software COPASI was utilized for the numerical analysis of this newly derived model. The association of two single anions **A** and cations **C** into the sandwich cation **CAC** and one free **A** (see Scheme S3) was numerically simulated using COPASI.

$$2 * A + 2 * C \xrightleftharpoons{K_{CAC}} A + CAC$$

Scheme S3. Equation for numerical simulations of newly derived cationic sandwich association model for salt **3a**.

The reaction was selected to be reversible. The sandwich association constant is defined as $k_1/k_2 = K_{CAC}$ with to $k_2 = 1$ being a fixed value.

3.4.1 Model 1

The procedure to find the best fitting sandwich association constant K_{CAC} is identical to the one described for the 1:1 association model. However, when converting the obtained concentration of each species, anion **A**, cation **C**, and sandwich-cation **CAC**, into conductivity values, the contribution of each species towards the molar conductivity is critical. Through literature search, we found the limited molar conductivity λ_{NBu4} of tetrabutylammonium cation (NBu_4^+, **b**) to be 45.5 S cm^2 mol^{-1},[7] which is also part of our pyridinamide ion pair library in combination with anion **3**, giving pyridinamide ion pair **3b** (see Scheme S4).

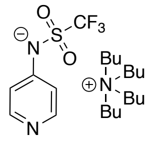

3b

Scheme S4. Structure of pyridinamide ion pair **3b**.

By recording the conductivity profile **3b** in DCM, its molar conductivity was identified to be 57.9 S cm^2 mol^{-1} (see Figure S4, red-marked initial three points for extrapolation of Λ_m).

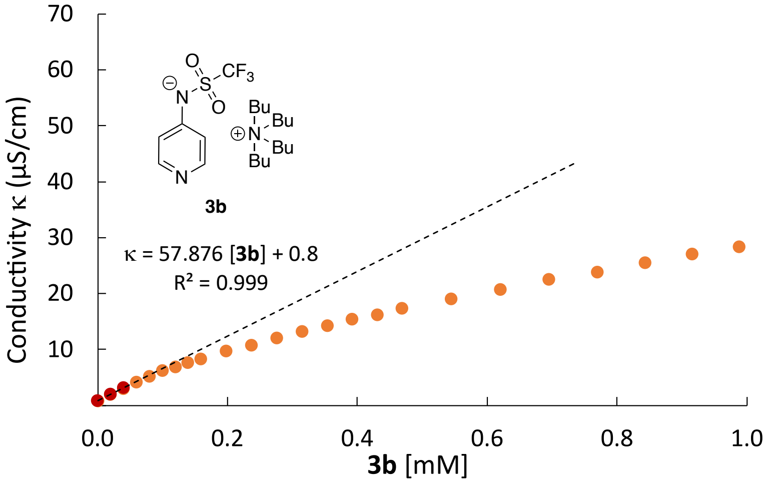

Figure S4. Concentration-dependent conductivity profile for catalyst **3b** in DCM with Λ_m = 57.9 S cm^2 mol^{-1} without solvent background at 20 °C.

By subtracting the limited molar conductivity λ_{NBu4} of NBu_4^+ form the molar conductivity Λ_m of salt **3b**, the limited molar conductivity λ_{an3} of anion **3** is determined to be λ_{an3} = 11.4 S cm^2 mol^{-1}. Since the molar conductivity of salt **3a** is 79.2 S cm^2 mol^{-1} in DCM, it can be concluded that the limited molar conductivity of cation **a** is λ_{PPh4} = 67.8 S cm^2 mol^{-1}. This translates to a contribution of 14% for the anion **A** and 86% for the cation **C** towards the total molar conductivity Λ_m of salt **3a**. Next, it is necessary to evaluate the contribution of the sandwich cation to the overall molar conductivity Λ_m of 79.2 S cm^2 mol^{-1}.

According to literature, it is common to assume a specific quotient for the limited molar conductivity of the sandwich-cation (λ_{CAC}/Λ_m). Most often the quotient is assumed to be 1/3[8–10], 2/3[11], or $(1/3)^{(1/3)}$ = 0.693[12,13]. The quotient λ_{CAC}/Λ_m = (1/3) was used for different salts, like lithium and tributylammonium thiocyanates[9] or 1-ethyl-3-methylimidazolium tetrafluoroborate and 1-butyl-3-methylimidazolium tetrafluoro borate[10], in various solvents. The quotient λ_{CAC}/Λ_m = $(1/3)^{(1/3)}$ was mainly used to describe the fluoroalkanoates and triethylamine chlorides in polar solvents as MeCN. Meanwhile, the quotient λ_{CAC}/Λ_m = 2/3 has been used for the analysis of tetraphenyl boron salts in THF, which is another spheric ion in an nonpolar solvent.[11] Therefore, it serves as the best reference for our system **3a**. So, for the future analysis of equilibria with sandwich cation formations in DCM, the quotient λ_{CAC}/Λ_m = δ_{CAC} = 2/3 will be used. Here, we introduce δ_i as *scaling factor* for the conversion of Λ_m into the respective λ_i. The final result for the sandwich association constant with 14% anion contribution, 86% cation

contribution and 2/3 sandwich cation contribution would be K_{CAC} = 2.62 x 10^6 M^{-2} for pyridinamide ion pair **3a** in DCM with an RMSE = 0.62 (see Table S3).

Table S3. Association constant K_{CAC} for pyridinamide ion pair **3a** according to model 1 with the respective limited ionic conductivities λ_i.

λ_A (S cm² mol⁻¹)	λ_C (S cm² mol⁻¹)	λ_{CAC} (S cm² mol⁻¹)	K_{CAC} (M^{-2})	RMSE
0.14 x Λ_m	0.86 x Λ_m	(2/3) x Λ_m	2.62 x 10^6	0.62

3.4.2 Model 2

a) Cationic Sandwich Association (Model 2a)

The recent DOSY result, however, made us question if conventional conductivity measurements are able to measure solutions of high enough dilution to guarantee fully dissociated ions. Therefore, we decided on a second approach toward the determination of the sandwich association constant based on the conductivity measurements, where the ratio between the anion and cation contribution to the molar conductivity Λ_m is treated as a second variable parameter. Since the model always starts from fully dissociated ions the sum of the scaling factors $\delta_A + \delta_C$ cannot exceed 1. The contribution of the sandwich cation was still set to be $2/3\Lambda_m$. Different ratios for δ_A/δ_C were tested and the full analysis to find K_{CAC} was done. Then the RMSE value for this sandwich association constant was compared (see Table S4).

Table S4. List of optimization steps for limited molar ionic conductivities λ_i with the corresponding association constants K_{CAC} for pyridinamide ion pair **3a** according to model 1.

λ_A (S cm² mol⁻¹)	λ_C (S cm² mol⁻¹)	λ_{CAC} (S cm² mol⁻¹)	K_{CAC} (M^{-2})	RMSE
0.14 x Λ_m	0.86 x Λ_m	(2/3) x Λ_m	2.62 x 10^6	0.620
0.30 x Λ_m	0.70 x Λ_m		4.0 x 10^6 – 5.0 x 10^6	<0.35
0.33 x Λ_m	0.67 x Λ_m		5.20 x 10^6	0.210
0.35 x Λ_m	0.65 x Λ_m		5.70 x 10^6	0.183
0.36 x Λ_m	0.64 x Λ_m		5.97 x 10^6	0.175
0.37 x Λ_m	0.63 x Λ_m		6.27 x 10^6	0.173
0.38 x Λ_m	0.62 x Λ_m		6.57 x 10^6	0.176
0.40 x Λ_m	0.60 x Λ_m		7.31 x 10^6	0.200

The best fit was found for K_{CAC} = 6.27 x 10^6 M^{-2} with a RMSE = 0.17, which is the final result for the sandwich association constant of pyridinamide ion pair **3a**.

Both equilibrium constants were used to determine the percentage speciation (see Table S5 and S6).

Table S5. Speciation of pyridinamide ion pair **3a** in 1:1 model in DCM with K_{IP} = 6.86 x 10^5 M^{-2} and their percentage.

[3a] / (M)	[A] / (M)	[C] / (M)	[IP] / (M)	[A]%	[C]%	[IP]%
0.00	0.00	0.00	0.00	–	–	–
1.78 x 10^{-5}	1.75 x 10^{-5}	1.75 x 10^{-5}	2.55 x 10^{-7}	0.99	0.99	0.01
3.55 x 10^{-5}	3.45 x 10^{-5}	3.45 x 10^{-5}	9.87 x 10^{-7}	0.97	0.97	0.03
5.40 x 10^{-5}	5.18 x 10^{-5}	5.18 x 10^{-5}	2.22 x 10^{-6}	0.96	0.96	0.04
7.09 x 10^{-5}	6.72 x 10^{-5}	6.72 x 10^{-5}	3.74 x 10^{-6}	0.95	0.95	0.05
8.86 x 10^{-5}	8.29 x 10^{-5}	8.29 x 10^{-5}	5.69 x 10^{-6}	0.94	0.94	0.06
1.06 x 10^{-4}	9.80 x 10^{-5}	9.80 x 10^{-5}	7.96 x 10^{-6}	0.92	0.92	0.08
1.24 x 10^{-4}	1.13 x 10^{-4}	1.13 x 10^{-4}	1.06 x 10^{-5}	0.91	0.91	0.09
1.41 x 10^{-4}	1.28 x 10^{-4}	1.28 x 10^{-4}	1.35 x 10^{-5}	0.90	0.90	0.10
1.76 x 10^{-4}	1.56 x 10^{-4}	1.56 x 10^{-4}	2.01 x 10^{-5}	0.89	0.89	0.11
2.11 x 10^{-4}	1.83 x 10^{-4}	1.83 x 10^{-4}	2.78 x 10^{-5}	0.87	0.87	0.13
2.46 x 10^{-4}	2.10 x 10^{-4}	2.10 x 10^{-4}	3.64 x 10^{-5}	0.85	0.85	0.15

2.80 x 10^{-4}	2.34 x 10^{-4}	2.34 x 10^{-4}	4.55 x 10^{-5}	0.84	0.84	0.16
3.15 x 10^{-4}	2.59 x 10^{-4}	2.59 x 10^{-4}	5.57 x 10^{-5}	0.82	0.82	0.18
3.49 x 10^{-4}	2.83 x 10^{-4}	2.83 x 10^{-4}	6.62 x 10^{-5}	0.81	0.81	0.19
3.83 x 10^{-4}	3.06 x 10^{-4}	3.06 x 10^{-4}	7.74 x 10^{-5}	0.80	0.80	0.20
4.17 x 10^{-4}	3.28 x 10^{-4}	3.28 x 10^{-4}	8.91 x 10^{-5}	0.79	0.79	0.21
4.85 x 10^{-4}	3.71 x 10^{-4}	3.71 x 10^{-4}	1.14 x 10^{-4}	0.76	0.76	0.24
5.52 x 10^{-4}	4.12 x 10^{-4}	4.12 x 10^{-4}	1.40 x 10^{-4}	0.75	0.75	0.25
6.19 x 10^{-4}	4.51 x 10^{-4}	4.51 x 10^{-4}	1.68 x 10^{-4}	0.73	0.73	0.27
6.85 x 10^{-4}	4.88 x 10^{-4}	4.88 x 10^{-4}	1.97 x 10^{-4}	0.71	0.71	0.29
7.50 x 10^{-4}	5.23 x 10^{-4}	5.23 x 10^{-4}	2.27 x 10^{-4}	0.70	0.70	0.30
8.15 x 10^{-4}	5.58 x 10^{-4}	5.58 x 10^{-4}	2.57 x 10^{-4}	0.68	0.68	0.32
8.80 x 10^{-4}	5.91 x 10^{-4}	5.91 x 10^{-4}	2.89 x 10^{-4}	0.67	0.67	0.33
9.44 x 10^{-4}	6.23 x 10^{-4}	6.23 x 10^{-4}	3.21 x 10^{-4}	0.66	0.66	0.34
1.01 x 10^{-3}	6.55 x 10^{-4}	6.55 x 10^{-4}	3.55 x 10^{-4}	0.65	0.65	0.35

Table S6. Speciation of pyridinamide ion pair **3a** in sandwich association model in DCM with K_{CAC} = 6.27x 10^6 M^{-2} and their percentage.

[3a] / (M)	[A] / (M)	[C] / (M)	[CAC] / (M)	[A]%	[C]%	[CAC]%
0.00	0.00	0.00	0.00	–	–	–
1.78 x 10^{-5}	1.78 x 10^{-5}	1.77 x 10^{-5}	3.50 x 10^{-8}	1.00	1.00	0.00
3.55 x 10^{-5}	3.52 x 10^{-5}	3.50 x 10^{-5}	2.70 x 10^{-7}	0.99	0.98	0.01
5.40 x 10^{-5}	5.31 x 10^{-5}	5.22 x 10^{-5}	9.07 x 10^{-7}	0.98	0.97	0.02
7.09 x 10^{-5}	6.90 x 10^{-5}	6.70 x 10^{-5}	1.94 x 10^{-7}	0.97	0.95	0.03
8.86 x 10^{-5}	8.51 x 10^{-5}	8.15 x 10^{-5}	3.54 x 10^{-6}	0.96	0.92	0.04
1.06 x 10^{-4}	1.00 x 10^{-4}	9.47 x 10^{-5}	5.64 x 10^{-6}	0.95	0.89	0.05
1.24 x 10^{-4}	1.16 x 10^{-4}	1.07 x 10^{-4}	8.35 x 10^{-6}	0.93	0.87	0.07
1.41 x 10^{-4}	1.30 x 10^{-4}	1.18 x 10^{-4}	1.14 x 10^{-5}	0.92	0.84	0.08
1.76 x 10^{-4}	1.57 x 10^{-4}	1.38 x 10^{-4}	1.88 x 10^{-5}	0.89	0.79	0.11
2.11 x 10^{-4}	1.83 x 10^{-4}	1.55 x 10^{-4}	2.78 x 10^{-5}	0.87	0.74	0.13
2.46 x 10^{-4}	2.08 x 10^{-4}	1.70 x 10^{-4}	3.79 x 10^{-5}	0.85	0.69	0.15
2.80 x 10^{-4}	2.31 x 10^{-4}	1.83 x 10^{-4}	4.85 x 10^{-5}	0.83	0.65	0.17
3.15 x 10^{-4}	2.55 x 10^{-4}	1.94 x 10^{-4}	6.03 x 10^{-5}	0.81	0.62	0.19
3.49 x 10^{-4}	2.77 x 10^{-4}	2.04 x 10^{-4}	7.24 x 10^{-5}	0.79	0.59	0.21
3.83 x 10^{-4}	2.98 x 10^{-4}	2.13 x 10^{-4}	8.49 x 10^{-5}	0.78	0.56	0.22
4.17 x 10^{-4}	3.19 x 10^{-4}	2.21 x 10^{-4}	9.79 x 10^{-5}	0.77	0.53	0.23
4.85 x 10^{-4}	3.60 x 10^{-4}	2.35 x 10^{-4}	1.25 x 10^{-4}	0.74	0.48	0.26
5.52 x 10^{-4}	3.99 x 10^{-4}	2.47 x 10^{-4}	1.53 x 10^{-4}	0.72	0.45	0.28
6.19 x 10^{-4}	4.38 x 10^{-4}	2.57 x 10^{-4}	1.81 x 10^{-4}	0.71	0.41	0.29
6.85 x 10^{-4}	4.75 x 10^{-4}	2.65 x 10^{-4}	2.10 x 10^{-4}	0.69	0.39	0.31
7.50 x 10^{-4}	5.11 x 10^{-4}	2.73 x 10^{-4}	2.39 x 10^{-4}	0.68	0.36	0.32
8.15 x 10^{-4}	5.47 x 10^{-4}	2.79 x 10^{-4}	2.68 x 10^{-4}	0.67	0.34	0.33
8.80 x 10^{-4}	5.83 x 10^{-4}	2.85 x 10^{-4}	2.97 x 10^{-4}	0.66	0.32	0.34
9.44 x 10^{-4}	6.17 x 10^{-4}	2.91 x 10^{-4}	3.27 x 10^{-4}	0.65	0.31	0.35
1.01 x 10^{-3}	6.53 x 10^{-4}	2.95 x 10^{-4}	3.57 x 10^{-4}	0.65	0.30	0.35

In both association models the anion and cation are mainly dissociated at low concentrations. While in the 1:1 model the concentration of anion and cation are always identical, in the sandwich association the concentration of free cation [C] is faster declining than the concentration of free anion [A] due to the formation of the sandwich cation. In both association model the concentration of free anion [A] is 65% and available to react as nucleophilic catalyst in solution.

The ion pair **4a** and additive **6** were analyzed the same way as pyridinamide salt **3a** with the two-parameter approach (model 2) for the sandwich association constant K_{CAC}. The results can be found in Table S7.

Table S7. Association constants K_{IP} and K_{CAC} according to the respective biochemical models of ion catalysts **3a**, **4a**, and additive **6** at 20 °C in DCM.

Ion Pair	K_{IP} (M^{-2})	K_{CAC} (M^{-2})
**3a**	6.86 x 10^5	6.27 x 10^6
**4a**	8.79 x 10^5	6.71 x 10^6
**6**	1.01 x 10^6	7.28 x 10^6

b) Anion Sandwich Association (Model 2b)

Aside from the previously discussed cationic sandwich association, the contrasting anionic sandwich association leading to a free cation **C** and a sandwich anion **ACA**, which consists of two anions **A** and one central cation **C**, is equally thinkable. For the analysis of this association type, we applied the same numerical model as discussed earlier using COPASI (see Scheme S5).

$$2 * A \; + \; 2 * C \xrightleftharpoons{K_{ACA}} C \; + \; ACA$$

Scheme S5. Equation for numerical simulations of anionic sandwich association model for salt **3a**.

The anion sandwich association constant is defined as $k_1/k_2 = K_{ACA}$ with to $k_2 = 1$ being a fixed value. The contribution of the sandwich anion was also set to be 2/3*Λ_m. Different ratios for $\lambda_A + \lambda_C$ were tested, and numerical simulation were performed until the best association constant K_{ACA} was found. The RMSE value for the anion sandwich association constant K_{ACA} was used as a quality control measure.

Following the above-described guideline for model 2 resulted in exactly the same anion sandwich association constant as the cationic sandwich association with $K_{ACA} = K_{CAC} = 6.27 \times 10^6$ M^{-2}. This was to be expected since the individual limited ionic conductivities λ_i of the overall molar conductivity Λ_m are treated as variables here, while the limited molar conductivity λ_i of the respective sandwich ion is fixed to be 2/3*Λ_m. Therefore, the molar ionic conductivity contributions interchanged by moving from the cationic sandwich association to the anionic sandwich association (see Table S8).

Table S8. Association constants K_{CAC} and K_{ACA} for pyridinamide ion pair **3a** according to model 2 with the respective limited ionic conductivities λ_i.

Sandwich Type	λ_A (S cm^2 mol^{-1})	λ_C (S cm^2 mol^{-1})	$\lambda_{i=CAC,ACA}$ (S cm^2 mol^{-1})	K (M^{-2})	RMSE
**Cationic**	0.37 x Λ_m	0.63 x Λ_m	(2/3) x Λ_m	6.27 x 10^6	0.17
**Anionic**	0.63 x Λ_m	0.37 x Λ_m	(2/3) x Λ_m	6.27 x 10^6	0.17

To conclude, based on the conductivity data, we cannot determine with certainty which type of sandwich ion association is favored.

3.4.3 Model 3

a) Cation Sandwich Association (Model 3a)

Further development of the conductivity model led to the following changes. We specifically focused on how we determine the contribution made towards the molar specific conductivity Λ_m by each of the charged species. Fuoss et. al. stated that the limited molar ionic conductivity λ of each ion is proportional to their volume.[14,15] Therefore, we applied this approach by using the calculated cavity volumes of each anion, cation, and ion pair to calculate the percentage share of the anion volume ***A*** and cation volume ***C*** (see eq. S8), which will then be used as scaling factor δ_i to convert Λ_m into the respective λ_i.

$$\delta_A = \frac{\mathrm{vol(A)}}{\mathrm{vol(IP)}} \tag{S8a}$$

$$\delta_C = \frac{\mathrm{vol(C)}}{\mathrm{vol(IP)}} \tag{S8b}$$

Here, the calculated volumes of anion **3** (215 Å^3), cation **a** (362 Å^3), and contact ion pair **3a** (570 Å^3), which are based on the van der Waals cavities employed in the SMD continuum solvation model at the SMD(DCM)/B3LYP-D3/6-31+G(d) level of theory are used (see Table S19 for calculated volumes of all ionic species). Since there is no known way to determine the limited molar ionic conductivity λ_{CAC} for the cation sandwich in DCM, we decided to treat it as another variable instead of assigning a set value to it. This refined approach gives us the following results for the cationic sandwich association (see Table S9). All scaling factors δ_i are limited to two decimals when used in the excel sheet to transform concentration into conductivity values.

Table S9. Association constant K_{CAC} for salt **3a**, **4a**, and **6** according to model 3a with the respective limited molar ionic conductivities λ_i and the scaling factors δ_i.

Ion Pair	Λ_m (S cm^2 mol^{-1})	λ_A (S cm^2 mol^{-1})	λ_C (S cm^2 mol^{-1})	δ_A /δ_C /δ_{CAC} ratio	K_{CAC} (M^{-2})	RMSE
**3a**	79.2	29.3	49.9	37/63/67	6.38 x 10^6	0.17
**4a**	73.6	32.4	41.2	44/56/54	6.50 x 10^6	0.12
**6**	95.2	15.2	80.0	16/84/80	7.05 x 10^6	0.16

Comparing the results from model 2 with model 3 show only small deviations between the obtained sandwich association constants K_{CAC}.

b) Anion Sandwich Association (Model 3b)

The analysis of the conductivity data according to model 3 for the anion sandwich association gives the same value for the anionic sandwich association constant K_{ACA} as for the cationic sandwich association with $K_{ACA} = K_{CAC} = 6.38 \times 10^6$ M^{-2} for ion pair **3a** and $K_{ACA} = K_{CAC} = 6.50 \times 10^6$ M^{-2} for ion pair **4a**. The results are summarized in Table S10.

Table S10. Association constant K_{ACA} for salt **3a** and **4a** according to model 3b with the respective limited molar ionic conductivities λ_i and the scaling factors δ_i.

Ion Pair	Λ_m (S cm^2 mol^{-1})	λ_A (S cm^2 mol^{-1})	λ_C (S cm^2 mol^{-1})	δ_A /δ_C /δ_{ACA} ratio	K_{ACA} (M^{-2})	RMSE
**3a**	79.2	29.3	49.9	37/63/41	6.38 x 10^6	0.17
**4a**	73.6	32.4	41.2	44/56/42	6.50 x 10^6	0.12

Thus, both sandwich association types are not distinguishable based on conductivity data alone. Therefore, we need to look at the DOSY measurements to gain an understanding of which association type is a better fit for each respective ion pair.

3.5 Mixed Sandwich Association (Model 4)

So far, we have separately analyzed the conductivity data for both cationic and anionic sandwich association. However, the comparison between the simulated DOSY volumes based on the numerical simulation of conductivity data for both association types and the experimentally determined DOSY volumes revealed significant deviations (see Figure S5).

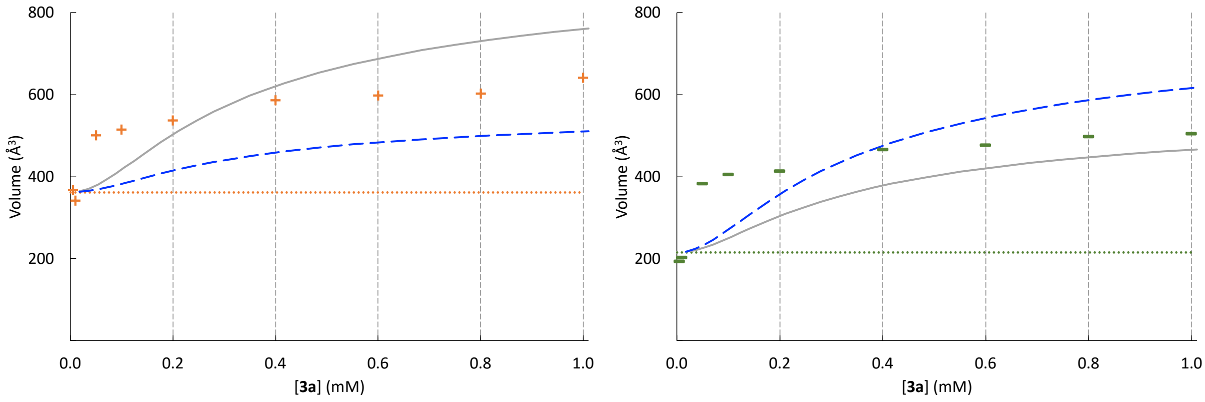

Figure S5. Concentration-dependent volumes of cations **a** (orange "+" symbols) and anions **3** (green "–" symbols) of compound **3a** in CD_2Cl_2 as calculated from DOSY experiments (with data from Table S16) with the respective free ion volumes (dotted line; cation in orange; anion in green), the calculated trend for the cationic (grey line) and anionic (blue dashed line) sandwich model.

To minimize this deviation, we extended our earlier described conductivity model 3. Since the resulting association constant was identical for both the cationic and anionic sandwich associations under model 3, the idea was to combine both sandwich association types into one model, which we will refer to as the *mixed association model* moving forward.

$$2 * A \;+\; 2 * C \;\underset{}{\overset{K_{CAC}}{\rightleftharpoons}}\; A \;+\; CAC$$

$$2 * A \;+\; 2 * C \;\underset{}{\overset{K_{ACA}}{\rightleftharpoons}}\; C \;+\; ACA$$

Scheme S6. Cation sandwich association and anion sandwich association model for pyridinamide ion pair **3a**.

To find the right balance between the cation sandwich association and the anion sandwich association each association constant was assigned a *scaling factor*, which would be multiplied with the respective sandwich association constant whereas, where α would be a fixed value decreasing from 1 in steps of 0.1 (see eq. S9)

$$K_{CAC} \text{ x } \alpha \qquad \text{(S9a)}$$

$$K_{ACA} \text{ x } \beta \qquad \text{(S9b)}$$

For each step the best β value with the smallest RMSE for the conductivity data was determined by employing numerical simulations. The optimization of the scaling factor was limited to two relevant decimals.

Table S11. List of association constants based on the optimized scaling factors α and β with the calculated anion and cation volumes (Å^3) and the respective percentual residual between the reference ion volume and calculated ion volume for pyridinamide ion pair **3a**.

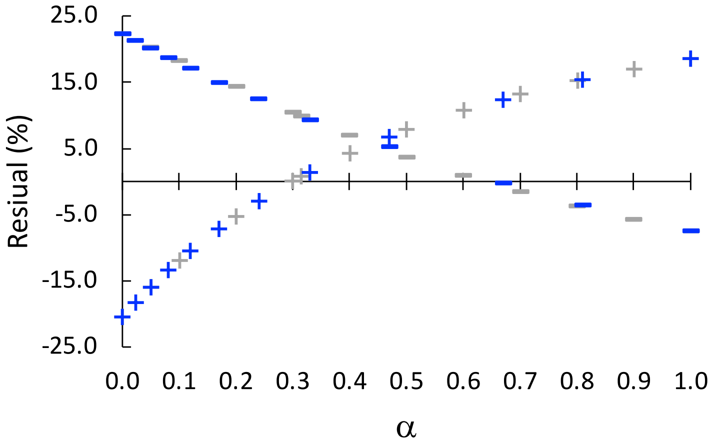

K_{CAC} (M^{-2})	K_{ACA} (M^{-2})	α	β	RMSE	V_{an} (Å^3)	V_{cat} (Å^3)	Residual (%) Anion	Residual (%) Cation
**Reference**					505	642		
6.38 x 10^6	0.00	1.00	0.00	0.17	467	761	-7.51	18.64
5.74 x 10^6	1.47 x 10^5	0.90	0.023	0.18	476	751	-5.72	17.05
5.10 x 10^6	3.19 x 10^5	0.80	0.05	0.22	486	739	-3.73	15.23
4.47 x 10^6	5.17 x 10^5	0.70	0.081	0.28	497	726	-1.60	13.18
3.83 x 10^6	7.66 x 10^5	0.60	0.12	0.34	509	711	0.84	10.74
3.19 x 10^6	1.08 x 10^6	0.50	0.17	0.4	523	692	3.58	7.84
2.55 x 10^6	1.53 x 10^6	0.40	0.24	0.45	540	669	6.94	4.30
2.01 x 10^6	2.01 x 10^6	0.315	0.315	0.46	554	647	9.84	0.75
1.91 x 10^6	2.11 x 10^6	0.30	0.33	0.46	557	642	10.38	0.03
1.28 x 10^6	3.00 x 10^6	0.20	0.47	0.42	577	608	14.38	-5.24
6.38 x 10^5	4.27 x 10^6	0.10	0.67	0.3	597	565	18.26	-11.93
3.19 x 10^5	5.17 x 10^6	0.05	0.81	0.22	607	540	20.24	-15.91
0.00	6.38 x 10^6	0.00	1.00	0.17	617	511	22.28	-20.38
1.47 x 10^5	5.74 x 10^6	0.023	0.90	0.18	612	524	21.26	-18.28
3.19 x 10^5	5.10 x 10^6	0.05	0.80	0.22	606	540	20.07	-15.93
5.17 x 10^5	4.47 x 10^6	0.081	0.70	0.28	599	556	18.68	-13.39
7.66 x 10^5	3.83 x 10^6	0.12	0.60	0.34	591	575	17.01	-10.44
1.08 x 10^6	3.19 x 10^6	0.17	0.50	0.4	580	596	14.98	-7.08
1.53 x 10^6	2.55 x 10^6	0.24	0.40	0.45	568	623	12.49	-2.97
2.11 x 10^6	1.91 x 10^6	0.33	0.30	0.46	552	651	9.29	1.46
3.00 x 10^6	1.28 x 10^6	0.47	0.20	0.42	531	685	5.25	6.71
4.27 x 10^6	6.38 x 10^5	0.67	0.10	0.3	503	721	-0.26	12.32
5.17 x 10^6	3.19 x 10^5	0.81	0.05	0.22	486	740	-3.65	15.37

This routine was repeated with β as the fixed factor and α as the factor to be optimized, ensuring compliance between both approaches, as shown in Figure S6.

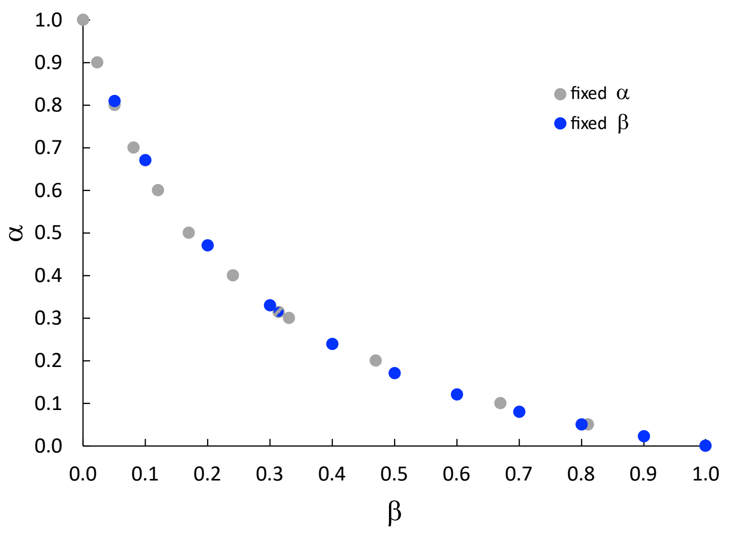

Figure S6. Graphical representation of the ratio of the optimized scaling factors α and β with α being the fixed value for the optimization of β (grey dots) and in reverse β being the fixed value for the optimization of α (blue dots) for pyridinamide ion pair **3a**.

The two data sets are almost identical with small deviations especially in the outer regions where one factor is significantly larger than the other. Therefore, the performance of each optimization step based on both scaling factors α and β is crucial since each chosen fix point offers a unique point of view onto the ideal sandwich association mixture. The zenith is found at α = β = 0.315 with K_{CAC} = K_{ACA} = 2.01 x 10^6 M^{-2}.

The next step involved incorporating experimental DOSY data by using the experimental DOSY volumes of ion pair **3a** as a reference value (with V_{an} = 505 Å^3 and V_{cat} = 642 Å^3). Numerical simulations provided concentrations used to calculate simulated DOSY volumes (see eq. S10).

$$\mathrm{vol_{cat}} = \frac{[\mathrm{C}]}{[\mathrm{IP}]_{\mathrm{tot}}} \times 362 + \left(\frac{2 \times [\mathrm{CAC}]}{[\mathrm{IP}]_{\mathrm{tot}}}\right) \times 925 + \left(\frac{[\mathrm{ACA}]}{[\mathrm{IP}]_{\mathrm{tot}}}\right) \times 782 \qquad \text{(S10a)}$$

$$\mathrm{vol_{an}} = \frac{[\mathrm{A}]}{[\mathrm{IP}]_{\mathrm{tot}}} \times 215 + \left(\frac{[\mathrm{CAC}]}{[\mathrm{IP}]_{\mathrm{tot}}}\right) \times 925 + \left(\frac{2 \times [\mathrm{ACA}]}{[\mathrm{IP}]_{\mathrm{tot}}}\right) \times 782 \qquad \text{(S10b)}$$

Subsequently, the percentual residual between experimental and simulated ion volumes determined the optimal mix of cationic and anionic sandwich association, showing that α = β = 0.44/0.21 for ion pair **3a** was optimal (see Table S12). The previous optimization steps of α and β indicates in what region further optimization steps need to be taken e.g. for ion pair **3a** additional optimization was done starting from α/β = 0.47/0.20.

Table S12. List of association constants based on the optimized scaling factors α and β with the calculated anion and cation volumes (Å³) and the respective percentual residual between the reference ion volume and calculated ion volume with focus on the area around the smallest percentual residue for both ion volumes for **3a**.

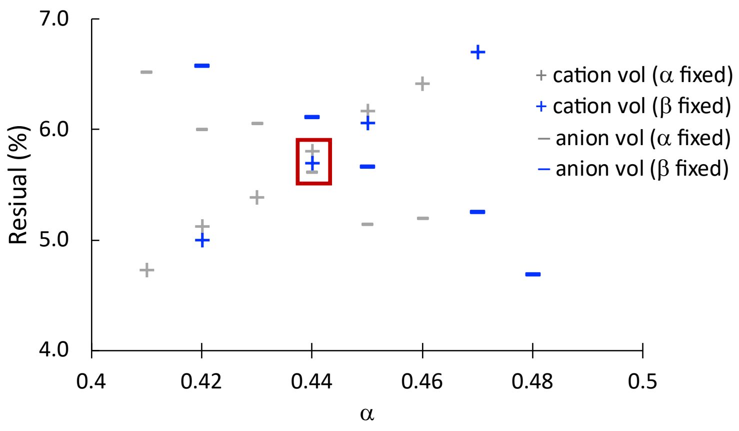

K_{CAC} (M^{-2})	K_{ACA} (M^{-2})	α	β	RMSE	V_{an} (Å³)	V_{cat} (Å³)	Residual (%) Anion	Residual (%) Cation
**Reference**					505	642		
2.93 x 10^6	1.28 x 10^6	0.46	0.20	0.42	531	683	5.20	6.42
2.87 x 10^6	1.28 x 10^6	0.45	0.20	0.43	531	681	5.15	6.17
2.81 x 10^6	1.34 x 10^6	**0.44**	**0.21**	0.43	533	679	**5.61**	**5.80**
2.74 x 10^6	1.40 x 10^6	0.43	0.22	0.44	535	676	6.06	5.39
2.68 x 10^6	1.40 x 10^6	0.42	0.22	0.44	535	675	6.01	5.12
2.62 x 10^6	1.47 x 10^6	0.41	0.23	0.44	538	672	6.52	4.73
3.06 x 10^6	1.21 x 10^6	0.48	0.19	0.41	528	687	4.68	7.08
3.00 x 10^6	1.28 x 10^6	0.47	0.20	0.42	531	685	5.25	6.71
2.87 x 10^6	1.34 x 10^6	0.45	0.21	0.43	533	681	5.66	6.06
2.81 x 10^6	1.40 x 10^6	**0.44**	**0.22**	0.43	536	678	**6.11**	**5.70**
2.68 x 10^6	1.47 x 10^6	0.42	0.23	0.44	538	674	6.57	5.00

Comparing the simulated DOSY data based on Model 3 for the cationic and anionic sandwich association with the newly introduced mixed model demonstrated a significant improved overlap with experimental data than any other association model (see Figure S7).

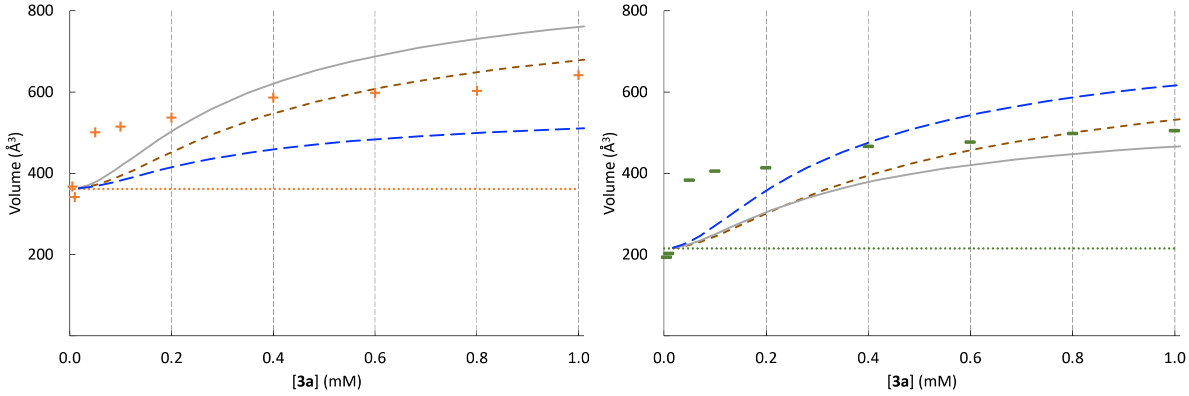

Figure S7. On the left side of Figure S7 we show: a) the DOSY cation volumes (orange "+" symbols); b) the free cation volume (orange dotted line); c) the average cation volume for the cationic sandwich model (grey line); d) the average cation volume for the anionic sandwich model (blue dashed line); e) the average cation volume for the mixed sandwich association model (brown short-dash line). On the right side of Figure S7 we show: a) the DOSY anion volumes (green "–" symbols); b) the free anion volume (green dotted line); c) the average anion volume for

the cationic sandwich model (grey line); d) the average anion volume for the anionic sandwich model (blue dashed line); e) the average anion volume for the mixed sandwich association model (brown short-dash line).

This procedure was repeated for pyridinamide ion pair **4a**. The results of the mixed model analysis are summarized in Table S13.

Table S13. Specific molar conductivity Λ_m, the scaling factors δ_i and association constants K_{CAC} and K_{ACA} with the respective scaling factors α and β for pyridinamide ion pair **3a** and **4a**.

Ion Pair	Λ_m (S cm^2 mol^{-1})	δ_A /δ_C /δ_{CAC}/δ_{ACA} (%)	α x K_{CAC} (M^{-2})	β x K_{ACA} (M^{-2})	α/β (%)	RMSE
**3a**	79.2	37/63/67/41	2.81 x 10^6	1.34 x 10^6	44/21	0.43
**4a**	73.6	44/56/54/42	7.80 x 10^5	3.97 x 10^6	12/61	0.35

The resulting association constants of the mixed model analysis for pyridinamide ion pair **3a** and **4a** were used to determine the percentage speciation (see Table S14 and S15).

Table S14. Speciation of pyridinamide ion pair **3a** in model 4 with α x K_{CAC} = 2.81 x 10^6 M^{-2} and β x K_{ACA} = 1.34 x 10^6 M^{-2} (α/β = 0.44/0.21), and the percentage of each involved charged species.

[3a]	[A] (M)	[C] (M)	[CAC] (M)	[ACA] (M)	[A]%	[C]%	[CAC]%	[ACA]%
0.00	0.00	0.00	0.00	0.00	–	–	–	–
1.78 x 10^{-5}	1.78 x 10^{-5}	1.78 x 10^{-5}	1.58 x 10^{-8}	7.51 x 10^{-9}	1.00	1.00	0.00	0.00
3.55 x 10^{-5}	3.53 x 10^{-5}	3.52 x 10^{-5}	1.23 x 10^{-7}	5.86 x 10^{-8}	0.99	0.99	0.00	0.00
5.40 x 10^{-5}	5.32 x 10^{-5}	5.30 x 10^{-5}	4.19 x 10^{-7}	2.01 x 10^{-7}	0.98	0.98	0.01	0.00
7.09 x 10^{-5}	6.91 x 10^{-5}	6.86 x 10^{-5}	9.15 x 10^{-7}	4.39 x 10^{-7}	0.97	0.97	0.01	0.01
8.86 x 10^{-5}	8.53 x 10^{-5}	8.44 x 10^{-5}	1.71 x 10^{-6}	8.22 x 10^{-7}	0.96	0.95	0.02	0.01
1.06 x 10^{-4}	1.01 x 10^{-4}	9.91 x 10^{-5}	2.77 x 10^{-6}	1.34 x 10^{-6}	0.95	0.93	0.03	0.01
1.24 x 10^{-4}	1.16 x 10^{-4}	1.14 x 10^{-4}	4.19 x 10^{-6}	2.04 x 10^{-6}	0.93	0.92	0.03	0.02
1.41 x 10^{-4}	1.29 x 10^{-4}	1.27 x 10^{-4}	5.82 x 10^{-6}	2.84 x 10^{-6}	0.92	0.90	0.04	0.02
1.76 x 10^{-4}	1.56 x 10^{-4}	1.51 x 10^{-4}	1.00 x 10^{-5}	4.93 x 10^{-6}	0.89	0.86	0.06	0.03
2.11 x 10^{-4}	1.81 x 10^{-4}	1.73 x 10^{-4}	1.52 x 10^{-5}	7.57 x 10^{-6}	0.86	0.82	0.07	0.04
2.46 x 10^{-4}	2.03 x 10^{-4}	1.93 x 10^{-4}	2.12 x 10^{-5}	1.07 x 10^{-5}	0.83	0.78	0.09	0.04
2.80 x 10^{-4}	2.24 x 10^{-4}	2.10 x 10^{-4}	2.78 x 10^{-5}	1.41 x 10^{-5}	0.80	0.75	0.10	0.05
3.15 x 10^{-4}	2.44 x 10^{-4}	2.27 x 10^{-4}	3.52 x 10^{-5}	1.80 x 10^{-5}	0.77	0.72	0.11	0.06
3.49 x 10^{-4}	2.62 x 10^{-4}	2.41 x 10^{-4}	4.28 x 10^{-5}	2.22 x 10^{-5}	0.75	0.69	0.12	0.06
3.83 x 10^{-4}	2.79 x 10^{-4}	2.55 x 10^{-4}	5.09 x 10^{-5}	2.66 x 10^{-5}	0.73	0.67	0.13	0.07
4.17 x 10^{-4}	2.95 x 10^{-4}	2.67 x 10^{-4}	5.93 x 10^{-5}	3.12 x 10^{-5}	0.71	0.64	0.14	0.07
4.85 x 10^{-4}	3.26 x 10^{-4}	2.90 x 10^{-4}	7.69 x 10^{-5}	4.12 x 10^{-5}	0.67	0.60	0.16	0.08
5.52 x 10^{-4}	3.53 x 10^{-4}	3.10 x 10^{-4}	9.52 x 10^{-5}	5.18 x 10^{-5}	0.64	0.56	0.17	0.09
6.19 x 10^{-4}	3.79 x 10^{-4}	3.28 x 10^{-4}	1.14 x 10^{-4}	6.30 x 10^{-5}	0.61	0.53	0.18	0.10
6.85 x 10^{-4}	4.02 x 10^{-4}	3.44 x 10^{-4}	1.33 x 10^{-4}	7.46 x 10^{-5}	0.59	0.50	0.19	0.11
7.50 x 10^{-4}	4.24 x 10^{-4}	3.58 x 10^{-4}	1.53 x 10^{-4}	8.64 x 10^{-5}	0.57	0.48	0.20	0.12
8.15 x 10^{-4}	4.45 x 10^{-4}	3.71 x 10^{-4}	1.73 x 10^{-4}	9.86 x 10^{-5}	0.55	0.46	0.21	0.12
8.80 x 10^{-4}	4.65 x 10^{-4}	3.84 x 10^{-4}	1.92 x 10^{-4}	1.11 x 10^{-4}	0.53	0.44	0.22	0.13
9.44 x 10^{-4}	4.84 x 10^{-4}	3.95 x 10^{-4}	2.12 x 10^{-4}	1.24 x 10^{-4}	0.51	0.42	0.22	0.13
1.01 x 10^{-3}	5.02 x 10^{-4}	4.06 x 10^{-4}	2.33 x 10^{-4}	1.37 x 10^{-4}	0.50	0.40	0.23	0.14

Table S15. Speciation of pyridinamide ion pair **4a** in model 4 with α x K_{CAC} = 7.80 x 10^5 M^{-2} and β x K_{ACA} = 3.97 x 10^6 M^{-2} (α/β = 0.12/0.61), and the percentage of each involved charged species.

[4a] (M)	[A] (M)	[C] (M)	[CAC] (M)	[ACA] (M)	[A]%	[C]%	[CAC]%	[ACA]%
0.00	0.00	0.00	0.00	0.00	–	–	–	–

1.96×10^{-5}	1.95×10^{-5}	1.96×10^{-5}	5.83×10^{-9}	2.96×10^{-8}	1.00	1.00	0.00	0.00
3.91×10^{-5}	3.86×10^{-5}	3.88×10^{-5}	4.53×10^{-8}	2.29×10^{-7}	0.99	0.99	0.00	0.01
5.86×10^{-5}	5.70×10^{-5}	5.76×10^{-5}	1.47×10^{-7}	7.42×10^{-7}	0.97	0.98	0.00	0.01
7.81×10^{-5}	7.44×10^{-5}	7.58×10^{-5}	3.33×10^{-7}	1.67×10^{-6}	0.95	0.97	0.00	0.02
9.75×10^{-5}	9.08×10^{-5}	9.32×10^{-5}	6.15×10^{-7}	3.05×10^{-6}	0.93	0.96	0.01	0.03
1.17×10^{-4}	1.06×10^{-4}	1.10×10^{-4}	1.00×10^{-6}	4.92×10^{-6}	0.91	0.94	0.01	0.04
1.36×10^{-4}	1.20×10^{-4}	1.26×10^{-4}	1.48×10^{-6}	7.21×10^{-6}	0.88	0.93	0.01	0.05
1.56×10^{-4}	1.34×10^{-4}	1.42×10^{-4}	2.10×10^{-6}	1.01×10^{-5}	0.86	0.91	0.01	0.06
1.76×10^{-4}	1.46×10^{-4}	1.57×10^{-4}	2.82×10^{-6}	1.34×10^{-5}	0.83	0.89	0.02	0.08
1.94×10^{-4}	1.57×10^{-4}	1.70×10^{-4}	3.55×10^{-6}	1.67×10^{-5}	0.81	0.88	0.02	0.09
2.13×10^{-4}	1.68×10^{-4}	1.84×10^{-4}	4.41×10^{-6}	2.05×10^{-5}	0.79	0.86	0.02	0.10
2.32×10^{-4}	1.77×10^{-4}	1.97×10^{-4}	5.36×10^{-6}	2.46×10^{-5}	0.76	0.85	0.02	0.11
2.71×10^{-4}	1.96×10^{-4}	2.22×10^{-4}	7.54×10^{-6}	3.38×10^{-5}	0.72	0.82	0.03	0.12
3.09×10^{-4}	2.12×10^{-4}	2.45×10^{-4}	9.95×10^{-6}	4.37×10^{-5}	0.69	0.79	0.03	0.14
3.47×10^{-4}	2.26×10^{-4}	2.68×10^{-4}	1.26×10^{-5}	5.42×10^{-5}	0.65	0.77	0.04	0.16
3.84×10^{-4}	2.38×10^{-4}	2.88×10^{-4}	1.54×10^{-5}	6.50×10^{-5}	0.62	0.75	0.04	0.17
4.22×10^{-4}	2.50×10^{-4}	3.08×10^{-4}	1.85×10^{-5}	7.66×10^{-5}	0.59	0.73	0.04	0.18
4.59×10^{-4}	2.61×10^{-4}	3.27×10^{-4}	2.18×10^{-5}	8.83×10^{-5}	0.57	0.71	0.05	0.19
4.97×10^{-4}	2.71×10^{-4}	3.46×10^{-4}	2.53×10^{-5}	1.01×10^{-4}	0.54	0.70	0.05	0.20
5.34×10^{-4}	2.80×10^{-4}	3.64×10^{-4}	2.88×10^{-5}	1.13×10^{-4}	0.52	0.68	0.05	0.21
5.71×10^{-4}	2.88×10^{-4}	3.81×10^{-4}	3.25×10^{-5}	1.25×10^{-4}	0.50	0.67	0.06	0.22
6.08×10^{-4}	2.96×10^{-4}	3.97×10^{-4}	3.64×10^{-5}	1.38×10^{-4}	0.49	0.65	0.06	0.23
6.81×10^{-4}	3.10×10^{-4}	4.29×10^{-4}	4.44×10^{-5}	1.63×10^{-4}	0.45	0.63	0.07	0.24
7.54×10^{-4}	3.22×10^{-4}	4.59×10^{-4}	5.29×10^{-5}	1.89×10^{-4}	0.43	0.61	0.07	0.25
8.26×10^{-4}	3.34×10^{-4}	4.87×10^{-4}	6.18×10^{-5}	2.15×10^{-4}	0.40	0.59	0.07	0.26
8.98×10^{-4}	3.44×10^{-4}	5.14×10^{-4}	7.10×10^{-5}	2.42×10^{-4}	0.38	0.57	0.08	0.27
9.69×10^{-4}	3.53×10^{-4}	5.40×10^{-4}	8.05×10^{-5}	2.68×10^{-4}	0.36	0.56	0.08	0.28
1.04×10^{-3}	3.62×10^{-4}	5.66×10^{-4}	9.03×10^{-5}	2.94×10^{-4}	0.35	0.54	0.09	0.28

For both pyridinamide ion pair mainly dissociated anion and cation were shown at low concentrations.

At 1.0 mM salt concentration, the association of pyridinamide ion pair **3a** still showed 50% of free anion in solution which acted as reactive nucleophile. Meanwhile, pyridinamide ion pair **4a** only has 35% free anion at 1.0 mM since here the anionic sandwich association is more pronounced. This, however, was no concern in the nucleophilicity measurement since DOSY NMR tests revealed a shift in ion volumes for mixtures of **4a** with additive **6** (for details see Chapter 4).

3.6 Workflow Summary

The above detailed workflow for the determination of ion pairing constant K_{IP} and sandwich association constant K_{CAC}/K_{ACA}, and the composition of mixed model for pyridinamide ion pairs based on conductivity measurements in DCM, can be summarized in the following steps:

Determination of ion pairing constant K_{IP} for salt **3a**

a) Measure conductivity for **3a**.
b) Determine the molar conductivity Λ_m by linear extrapolation of the first three data points.
c) Use eq. S6 or the respective biochemical model (for details see Scheme S1 or S2) concentrations for all compound involved in the model, copy the concentrations into Excel to convert them into conductivity values by assigning both ion 50% contribution towards Λ_m.
d) Compare sum of theoretical conductivity with experimental values (without solvent background conductivity).
e) Use the RMSE value as a quality control measure.
f) Adjust k_1 in $K_{CAC} = k_1/k_2$ until the minimum RMSE is found, to obtain K_{IP}.

Determination of cationic and anionic sandwich association constant K_{CAC} or K_{ACA} for salt **3a** (Model 3)

g) Measure conductivity for **3a**.
h) Determine the molar conductivity Λ_m by linear extrapolation of the first three data points.
i) Set the limited molar ionic conductivity for the anion λ_A and cation λ_C to the calculated values of the scaling factor δ_i based on eq. S8. The sum of $\delta_A+\delta_C$ cannot exceed 1.
j) Treat $\delta_{CAC} = \lambda_{CAC}/\Lambda_m$(**3a**) as a second variable. Select at starting value e.g. $\delta_{CAC} = 0.67$ and start the optimization process for K_{CAC}.
k) Use the respective sandwich association model (Model 3) to obtain concentrations for all species involved, copy them into Excel to convert them into conductivity values using eq. S5.
l) Compare sum of theoretical conductivity with experimental values (without solvent background conductivity).
m) Use the RMSE values as a quality control measure.
n) Adjust k_1 in $K_{CAC} = k_1/k_2$ until minimum RMSE is found, to obtain the final K_{CAC}.
o) Adjust the molar ionic conductivity for the sandwich ion λ_{CAC} to find the percentual distribution that fits the conductivity data the best. For each new percentual distribution, repeat step k)-n) until the global minimum RMSE is found.

For analysis of the additive **6** follow these steps:

p) Measure conductivity for **6**.
q) Follow step i) – o) as described above to obtain K_{CAC} for additive **6**.

Determination of composition of cationic and anionic sandwich association for salt **3a** (Model 4)

After determination of the cationic sandwich association constant K_{CAC} and the anionic sandwich association K_{ACA} the mix of both association types that fits the experimental data best is ascertained.

r) Assign each association constant a *scaling factor*: K_{CAC} x α and K_{ACA} x β.
s) Set α to a fixed value starting at 1.0 and going down in steps of 0.1. Optimize β to achieve the smallest RMSE value for the conductivity data using numerical simulations. Limit each factor to two relevant decimals.
t) Repeat step s) vice versa for factor β.
u) Determine the composition of cationic and anionic sandwich association in reference to the measured DOSY volume at 1.0 mM by calculating the percentual residual between experimental and simulated ion volumes.
v) Take further optimization steps until finding the α/β ratio with the smallest percentual residual for both cation and anion volume.

3.7 Conductivity Data in MeCN

Raw data of concentration dependent conductivity profile of **3a** in MeCN at 20 °C

[**3a**]/M	Conductivity κ (μS/cm)
0.00	0.80
1.77 x 10^{-5}	2.96
3.54 x 10^{-5}	5.20
5.30 x 10^{-5}	7.44
7.06 x 10^{-5}	9.84
8.82 x 10^{-5}	11.8
1.06 x 10^{-4}	14.1
1.23 x 10^{-4}	16.3
1.41 x 10^{-4}	18.3
1.58 x 10^{-4}	20.8
1.75 x 10^{-4}	22.7
1.93 x 10^{-4}	25.0
2.10 x 10^{-4}	27.0
2.45 x 10^{-4}	31.5
2.79 x 10^{-4}	35.8
3.13 x 10^{-4}	39.7
3.47 x 10^{-4}	43.8
4.15 x 10^{-4}	52.5
4.83 x 10^{-4}	60.8
5.50 x 10^{-4}	68.5
6.16 x 10^{-4}	77.0
6.82 x 10^{-4}	85.2
7.47 x 10^{-4}	92.6
8.12 x 10^{-4}	100
8.76 x 10^{-4}	108
9.40 x 10^{-4}	115
1.00 x 10^{-3}	123
1.07 x 10^{-3}	130
1.13 x 10^{-3}	138
1.19 x 10^{-3}	145
1.25 x 10^{-3}	152
1.37 x 10^{-3}	166
1.49 x 10^{-3}	180
1.61 x 10^{-3}	193
1.73 x 10^{-3}	207
1.84 x 10^{-3}	220
1.96 x 10^{-3}	232
2.07 x 10^{-3}	245
2.18 x 10^{-3}	257

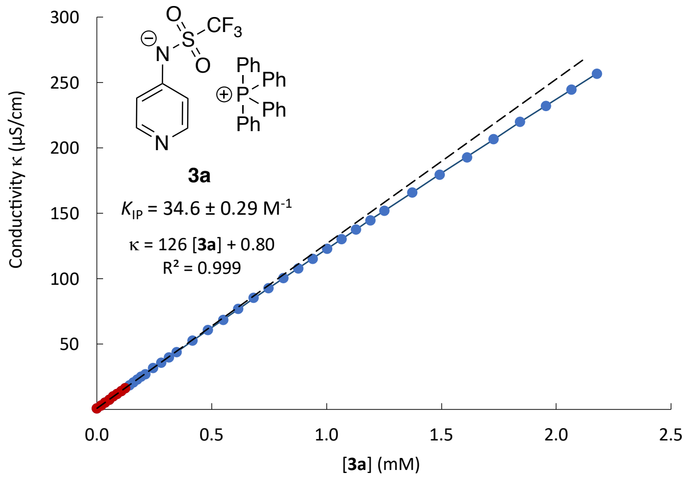

Raw data of concentration dependent conductivity data of **4a** in MeCN at 20 °C

[**4a**]/M	Conductivity κ (μS/cm)
0.00	0.80
1.85 x 10^{-5}	2.88
3.86 x 10^{-5}	5.04
5.78 x 10^{-5}	7.12
7.70 x 10^{-5}	9.28
9.62 x 10^{-5}	11.4
1.15 x 10^{-4}	13.6
1.34 x 10^{-4}	15.6
1.53 x 10^{-4}	17.5
1.72 x 10^{-4}	19.7
1.91 x 10^{-4}	21.8
2.10 x 10^{-4}	23.7
2.29 x 10^{-4}	25.8
2.67 x 10^{-4}	29.9
3.04 x 10^{-4}	33.9
3.42 x 10^{-4}	37.9
3.79 x 10^{-4}	42.2
4.53 x 10^{-4}	49.6
5.27 x 10^{-4}	57.1
5.99 x 10^{-4}	65.0
6.72 x 10^{-4}	72.6
7.44 x 10^{-4}	80.0
8.15 x 10^{-4}	87.4
8.85 x 10^{-4}	94.6
9.56 x 10^{-4}	102
1.03 x 10^{-3}	109
1.09 x 10^{-3}	116
1.23 x 10^{-3}	129
1.37 x 10^{-3}	143
1.50 x 10^{-3}	156
1.63 x 10^{-3}	168
1.76 x 10^{-3}	181
1.88 x 10^{-3}	193
2.01 x 10^{-3}	205
2.13 x 10^{-3}	217
2.25 x 10^{-3}	229
2.37 x 10^{-3}	240
2.49 x 10^{-3}	251
2.61 x 10^{-3}	262
2.72 x 10^{-3}	273
2.84 x 10^{-3}	283
2.95 x 10^{-3}	294

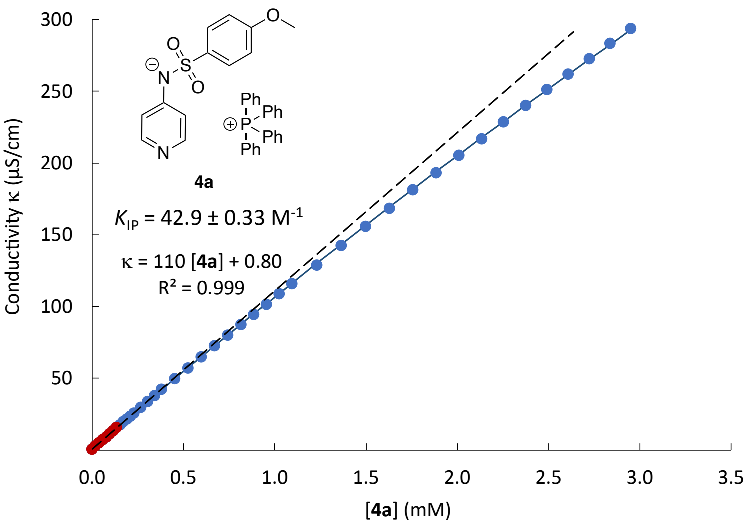

Raw data of concentration dependent conductivity profile of **6** in MeCN at 20 °C

[**6**]/M	Conductivity κ (μS/cm)
0.00	0.80
1.98 x 10^{-5}	3.52
3.96 x 10^{-5}	7.04
5.93 x 10^{-5}	9.76
7.90 x 10^{-5}	13.9
9.86 x 10^{-5}	17.0
1.18 x 10^{-4}	20.3
1.38 x 10^{-4}	23.6
1.57 x 10^{-4}	26.7
1.77 x 10^{-4}	29.8
1.96 x 10^{-4}	33.0
2.16 x 10^{-4}	36.0
2.35 x 10^{-4}	39.3
2.74 x 10^{-4}	45.4
3.12 x 10^{-4}	51.5
3.50 x 10^{-4}	57.8
3.89 x 10^{-4}	63.8
4.65 x 10^{-4}	76.0
5.40 x 10^{-4}	87.2
6.15 x 10^{-4}	98.8
6.89 x 10^{-4}	111
7.62 x 10^{-4}	122
8.35 x 10^{-4}	133
9.08 x 10^{-4}	144
9.80 x 10^{-4}	155
1.05 x 10^{-3}	166
1.12 x 10^{-3}	176
1.19 x 10^{-3}	184
1.26 x 10^{-3}	196
1.33 x 10^{-3}	207
1.40 x 10^{-3}	217
1.54 x 10^{-3}	237
1.67 x 10^{-3}	257
1.80 x 10^{-3}	275

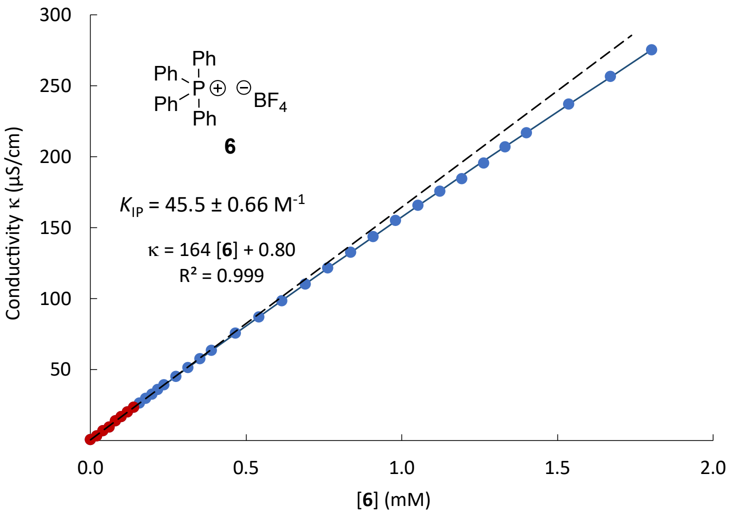

3.8 Conductivity Data in DCM

Raw data of concentration dependent conductivity profile of **3a** in DCM at 20 °C

[**3a**]/M	Conductivity κ (μS/cm)
0.00	0.80
1.78×10^{-5}	2.24
3.55×10^{-5}	3.60
5.40×10^{-5}	5.12
7.09×10^{-5}	6.48
8.86×10^{-5}	7.44
1.06×10^{-4}	8.80
1.24×10^{-4}	9.92
1.41×10^{-4}	11.0
1.76×10^{-4}	13.2
2.11×10^{-4}	15.4
2.46×10^{-4}	17.0
2.80×10^{-4}	19.2
3.15×10^{-4}	21.1
3.49×10^{-4}	23.0
3.83×10^{-4}	24.8
4.17×10^{-4}	26.4
4.85×10^{-4}	29.6
5.52×10^{-4}	32.8
6.19×10^{-4}	36.0
6.85×10^{-4}	39.2
7.50×10^{-4}	42.4
8.15×10^{-4}	45.1
8.80×10^{-4}	48.0
9.44×10^{-4}	50.6
1.01×10^{-3}	53.1

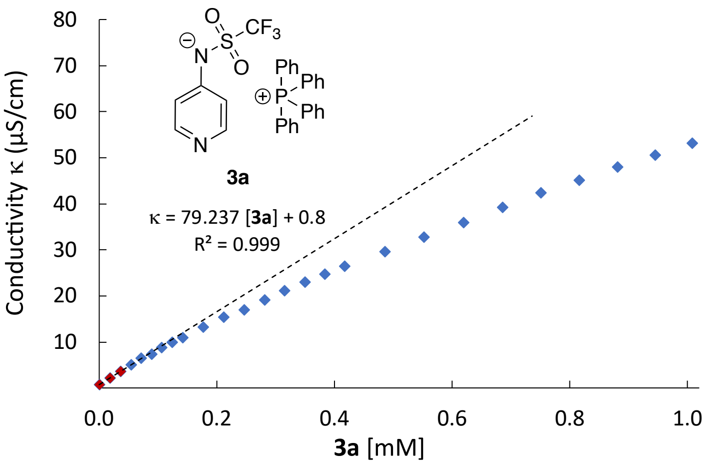

Raw data of concentration dependent conductivity profile of **3b** in DCM at 20 °C

[**3b**]/M	Conductivity κ (μS/cm)
0.00	0.80
2.00 x 10^{-5}	2.00
3.99 x 10^{-5}	3.09
5.98 x 10^{-5}	4.24
7.97 x 10^{-5}	5.28
9.95 x 10^{-5}	6.24
1.19 x 10^{-4}	6.96
1.39 x 10^{-4}	7.68
1.59 x 10^{-4}	8.40
1.98 x 10^{-4}	9.76
2.37 x 10^{-4}	10.9
2.76 x 10^{-4}	12.1
3.15 x 10^{-4}	13.3
3.54 x 10^{-4}	14.3
3.92 x 10^{-4}	15.4
4.30 x 10^{-4}	16.3
4.69 x 10^{-4}	17.4
5.45 x 10^{-4}	19.1
6.20 x 10^{-4}	20.8
6.95 x 10^{-4}	22.6
7.69 x 10^{-4}	23.9
8.43 x 10^{-4}	25.5
9.16 x 10^{-4}	27.1
9.88 x 10^{-4}	28.5
1.06 x 10^{-3}	29.8

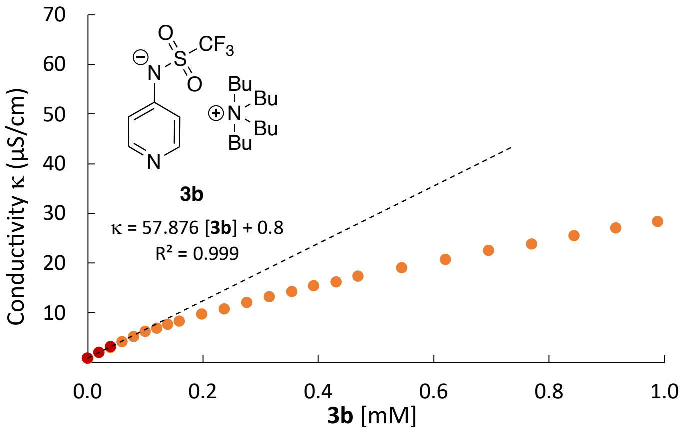

Raw data of concentration dependent conductivity profile of **4a** in DCM at 20 °C

[**4a**]/M	Conductivity κ (μS/cm)
0.00	0.80
1.96×10^{-5}	2.24
3.91×10^{-5}	3.68
5.86×10^{-5}	5.04
7.81×10^{-5}	6.40
9.75×10^{-5}	7.52
1.17×10^{-4}	8.96
1.36×10^{-4}	9.92
1.56×10^{-4}	11.0
1.76×10^{-4}	12.2
1.94×10^{-4}	13.1
2.13×10^{-4}	14.2
2.32×10^{-4}	15.3
2.71×10^{-4}	17.0
3.09×10^{-4}	19.0
3.47×10^{-4}	20.8
3.84×10^{-4}	22.6
4.22×10^{-4}	24.2
4.59×10^{-4}	25.9
4.97×10^{-4}	27.5
5.34×10^{-4}	29.2
5.71×10^{-4}	30.7
6.08×10^{-4}	32.5
6.81×10^{-4}	35.4
7.54×10^{-4}	38.2
8.26×10^{-4}	41.1
8.98×10^{-4}	43.7
9.69×10^{-4}	46.6
1.04×10^{-3}	49.1

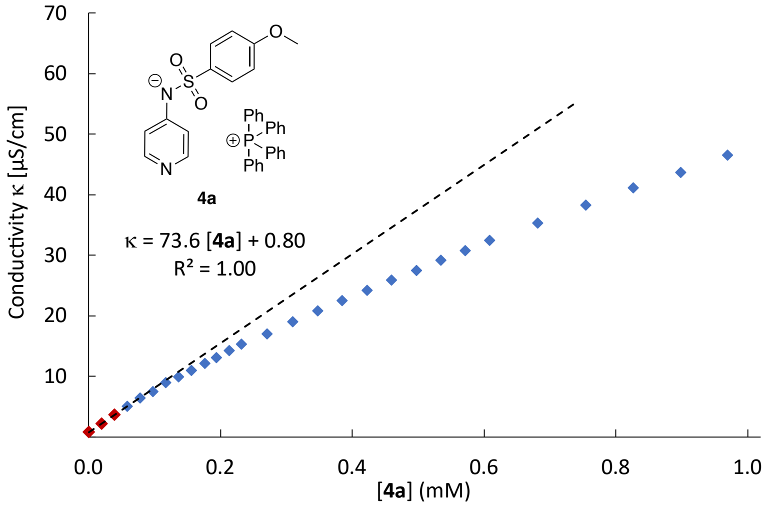

Raw data of concentration dependent conductivity profile of **6** in DCM at 20 °C

[**6**]/M	Conductivity κ (μS/cm)
0.00	0.80
1.93×10^{-5}	2.64
3.86×10^{-5}	4.48
5.79×10^{-5}	6.24
7.71×10^{-5}	7.84
9.63×10^{-5}	9.36
1.15×10^{-4}	10.6
1.35×10^{-4}	12.3
1.54×10^{-4}	13.8
1.92×10^{-4}	16.4
2.30×10^{-4}	19.0
2.67×10^{-4}	21.4
3.05×10^{-4}	23.9
3.42×10^{-4}	26.1
3.80×10^{-4}	28.4
4.17×10^{-4}	30.5
4.54×10^{-4}	32.6
5.27×10^{-4}	36.6
6.00×10^{-4}	40.6
6.73×10^{-4}	44.1
7.45×10^{-4}	47.9
8.16×10^{-4}	51.4
8.87×10^{-4}	55.0
9.57×10^{-4}	58.2
1.03×10^{-3}	61.7

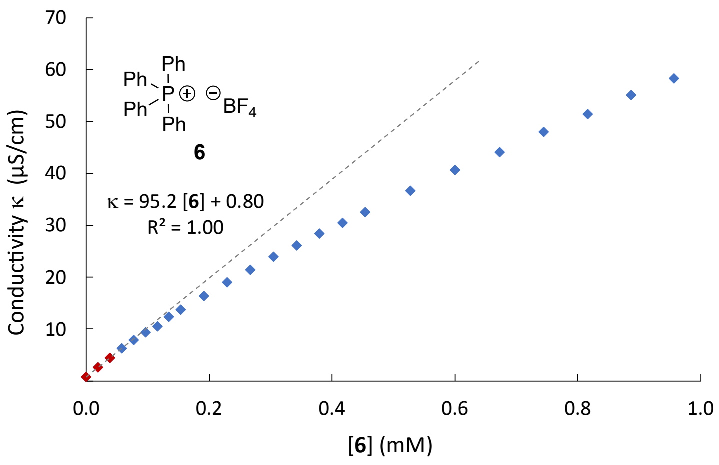

4. DOSY NMR Spectroscopy

Diffusion ordered NMR spectroscopy (DOSY) experiments were performed using a Bruker® Avance III 600 MHz operating at 600.25 MHz for protons, equipped with a 5-mm high-resolution TCI cryoprobe and with pulsed gradient units, capable of producing magnetic field pulsed gradients in the z-direction of 0.64 $T.m^{-1}$. All measurements were performed at 298 K. Temperature was certified by internal NMR calibration samples from Bruker®. NMR Data was processed, evaluated, and plotted with TopSpin 3.2 software and with a python script. Further analysis of the measurements was performed with Microsoft Excel (Version 16.0.10359.20023 64 Bit) and a python script.

Sample preparation for diffusion-ordered spectroscopy

For the sample preparation 1.0 mL stock solutions with a concentration of 1/5 mM were prepared of ion pair **3a** in freshly distilled deuterated solvents (CD_2Cl_2, CD_3CN). These stock solutions were then diluted to the respective concentrations. Thereafter the stock solution was put in an ultrasonic bath for one minute before it was used. Before the addition of the stock solutions, the NMR tubes were evacuated and flushed with Argon three times. Then the stock solution was added to the NMR tube under Argon flow. Tetramethyl silane (TMS) was added as a reference by withdrawing 0.5 mL from the atmosphere of the TMS bottle, just above the surface of the liquid. Afterwards, the NMR tube was sealed and again put in an ultrasonic bath for one minute before use.

Diffusion ordered spectroscopy (DOSY)

All DOSY measurements were performed with the convection suppressing DSTE (double stimulated echo) pulse sequence developed by Jerschow and Müller in a pseudo 2D mode.[16] Therefore, TMS was added to the sample and used as reference for the 1H chemical shifts and for Hydrodynamic radius as well as the viscosity of the solvent. For the measurement a set of 4 dummy scans and 32 scans was used for samples with concentration ≥ 0.5 mM of the ion pair. A set of 4 dummy scans and 64 scans were used for samples with concentrations ≥ 0.1 mM of the ion pair. A set of 4 dummy scans and 256 scans was used for samples with less than 0.05 mM of the ion pair. A relaxation delay of 3.5 s was used for all samples. The diffusion time delay was set to 40 ms and the gradient pulse lengths (p30, SINE.100 pulse shape) were optimized for each species to give a sigmoidal signal decay for varying gradient strengths between 5% and 95%. Optimal pulse lengths of 1.12 – 1.3 ms were used at 298 K for TMS and ion pairs. For each species, 32 spectra with linear varying gradient strength from 5% to 95% were measured. Thereby, no line broadening occurred for increased gradient strengths. For integration the corresponding signals of **3a** were used except if there was an overlap with another signal (see Figure S8).

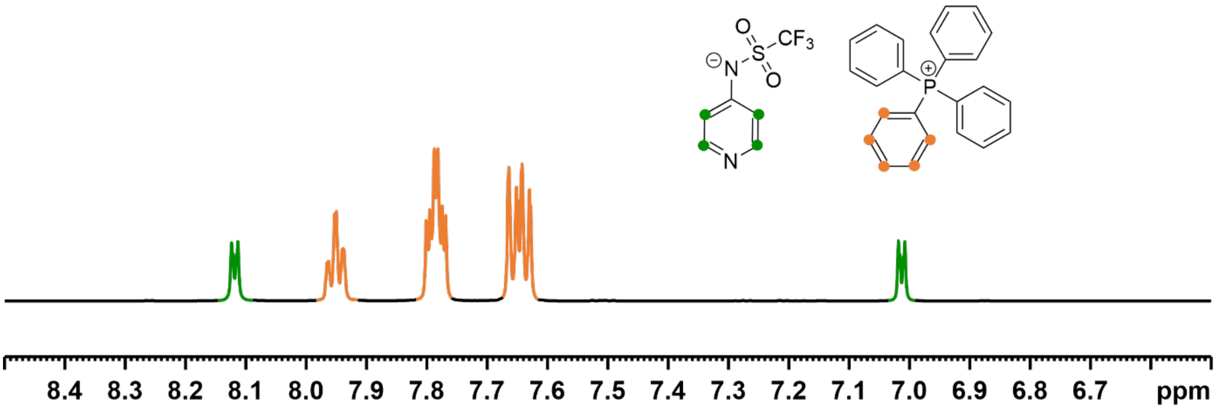

Figure S8. Signals used for DOSY evaluation of **3a**. Anion signals are marked in green, cation signals are marked in orange. All corresponding signals were used for DOSY evaluation and averaged for each ion.

In general, the transformation of diffusion coefficients into hydrodynamic radii via the Stejskal-Tanner equation is applicable for neutral molecules. In the case of ions, the additional electrostatic interactions between counter ions should be included and are expected to reduce the mobility of the ions at higher concentrations. In this manuscript extremely low concentrations of ions between 0.005 mM and 1.0 mM

are applied, therefore the electrostatic interactions should be negligible. Indeed, DOSY/Steijskal-Tanner/Stokes-Einstein approach for measurements at 0.005 mM were in good agreement with the calculated volumes of the free ions. Furthermore, upon concentration increase the DOSY data do not show any direct correlation to the concentration but a concentration dependence which in combination with the conductivity data and the nucleophilicity data is only consistent with the sandwich formation (see main text). In addition, for the interpretation mainly the relative values of cations and anions i.e. the offset is important, and this shouldn't be affected by the electrostatic interactions. Therefore, the signal intensities of these groups in the DOSY spectra were classically analyzed as a function of the gradient strength by the in Bruker TopSpin 3.2 included software T1/T2 relaxation package by employing the Stejskal-Tanner equation.[17] Based on the obtained translational diffusion coefficients, the hydrodynamic radii of the analytes r_H were estimated following the Stokes-Einstein equation (S11), with D_i = self-diffusion coefficient k_B = Boltzmann constant, T = temperature, η = viscosity of the sample, c = correcting factor, F = shape factor:[18]

$$D_i = \frac{k_B T}{F c \pi \eta r_H} \quad \text{(S11)}$$

The shape factor F was set to 1 for a spherical shape. The semi-empirical modification by Chen (S12) was used to calculate the correction factor c. Therefore, a from literature known value for the radius of the corresponding solvent was used ($r_{CD_2Cl_2}$ = 2.46 Å[19], r_{CD_3CN} = 2.86 Å[20]).[21]

$$c_{Chen} = \frac{6F}{1 + 0.695\,(\frac{r_{solv}}{r_{ref}})^{2.234}} \quad \text{(S12)}$$

Viscosity calibration of the derived D_i values was performed with literature known values for the radii of TMS (r_{ref} = 2.96 Å, calculated from hard-sphere increments[20]) and the experimentally determined diffusion coefficient D_{ref} of TMS, which is determined individually for each sample.

$$\eta\ [kg/ms] = \frac{k_B T\ (1 + 0.695\left(\frac{r_{solv}}{r_{ref}}\right)^{2.234})}{6\pi D_{ref}\ r_{ref}} \quad \text{(S13)}$$

After including all correction and calibration equations in the Stokes equation (S11), the equation was rearranged for the hydrodynamic radii r_H (S14). The corresponding volumes V_A were calculated with the assumption of a spherical shape.

$$D = \frac{k_B T\ (1 + 0.695\left(\frac{r_{solv}}{r_{ref}}\right)^{2.234})}{6\pi \eta r_H} \quad \text{(S14)}$$

The experimental self-diffusion coefficients D_i, the viscosity corrected hydrodynamic radii r_H and the resulting volumes V_A of all samples are depicted in Table S16 and Table S17. TMS was used as viscosity reference in each sample separately with the variation in its experimental diffusion coefficients reflecting the different viscosities depending on the ion pair concentration (see Table S16-18). The average D_i values were derived by using all baseline separated signals that were referring to the same species. As stated above the hydrodynamic values and the volumes are viscosity corrected and therefore the only values, which can be directly compared with each other.

Table S16. Experimental self-diffusion coefficients D_i, viscosity corrected hydrodynamic radii r_H and resulting volumes V_A of ion pair **3a** in varying concentrations. TMS was used as viscosity reference for the experimental self-diffusion coefficients D_i to allow for a comparison of hydrodynamic radii r_H and resulting volumes V_A. The corresponding self-diffusion coefficients D_i of TMS are given for each sample. Entry 1-17: SW = 22 Hz, O1P = 10.0 ppm, gradient strength 5-95% linear. Samples were measured at room temperature.

Sample	solvent	Species inside sample	Concentration (mM)	Diffusion coefficient D_i ($m^2.s^{-1}$)	Standard deviation D_i ($m^2.s^{-1}$)	Hydrodynamic radius r_H (Å)	Volume V_A (Å^3)	R^2
		**3a** Anion	0.005	1.81×10^{-9}	5.52×10^{-11}	3.59	192	0.992
**1**	CD_2Cl_2	**3a** Cation	0.005	1.33×10^{-9}	1.30×10^{-11}	4.44	367	0.997
		TMS		2.47×10^{-9}				
		**3a** Anion	0.01	1.66×10^{-9}	2.56×10^{-11}	3.64	203	0.998
**2**	CD_2Cl_2	**3a** Cation	0.01	1.29×10^{-9}	8.22×10^{-12}	4.34	342	0.998
		TMS		2.31×10^{-9}				
		**3a** Anion	0.05	1.32×10^{-9}	1.21×10^{-11}	4.51	383	0.999
**3**	CD_2Cl_2	**3a** Cation	0.05	1.18×10^{-9}	3.69×10^{-12}	4.93	500	0.999
		TMS		2.49×10^{-9}				
		**3a** Anion	0.1	1.29×10^{-9}	7.59×10^{-11}	4.59	405	0.999
**4**	CD_2Cl_2	**3a** Cation	0.1	1.16×10^{-9}	2.44×10^{-12}	4.97	515	0.999
		TMS		2.49×10^{-9}				
		**3a** Anion	0.2	1.26×10^{-9}	6.54×10^{-12}	4.62	413	0.999
**5**	CD_2Cl_2	**3a** Cation	0.2	1.13×10^{-9}	1.22×10^{-12}	5.04	537	0.999
		TMS		2.46×10^{-9}				
		**3a** Anion	0.4	1.21×10^{-9}	5.13×10^{-12}	4.81	466	0.999
**6**	CD_2Cl_2	**3a** Cation	0.4	1.10×10^{-9}	1.08×10^{-12}	5.19	587	0.999
		TMS		2.49×10^{-9}				
		**3a** Anion	0.6	1.19×10^{-9}	4.48×10^{-12}	4.84	477	0.999
**7**	CD_2Cl_2	**3a** Cation	0.6	1.08×10^{-9}	2.16×10^{-12}	5.23	598	0.999
		TMS		2.47×10^{-9}				
		**3a** Anion	0.8	1.17×10^{-9}	5.75×10^{-12}	4.92	497	0.999
**8**	CD_2Cl_2	**3a** Cation	0.8	1.08×10^{-9}	2.50×10^{-12}	5.24	603	0.999
		TMS		2.48×10^{-9}				
		**3a** Anion	1.0	1.20×10^{-9}	4.51×10^{-12}	4.85	477	0.999
**9**	CD_2Cl_2	**3a** Cation	1.0	1.07×10^{-9}	5.09×10^{-12}	5.32	629	0.999
		TMS		2.49×10^{-9}				
		**3a** Anion	1.0	1.18×10^{-9}	5.22×10^{-12}	4.98	517	0.999
**10**	CD_2Cl_2	**3a** Cation	1.0	1.07×10^{-9}	1.38×10^{-12}	5.36	645	0.999
		TMS		2.52×10^{-9}				
		**3a** Anion	1.0	1.19×10^{-9}	5.39×10^{-12}	4.92	497	0.999
**11**	CD_2Cl_2	**3a** Cation	1.0	1.07×10^{-9}	3.89×10^{-12}	5.36	643	0.999
		TMS		2.51×10^{-9}				
		**3a** Anion	1.0	1.18×10^{-9}	4.96×10^{-12}	5.01	527	0.999
**12**	CD_2Cl_2	**3a** Cation	1.0	1.08×10^{-9}	4.53×10^{-12}	5.37	649	0.999
		TMS		2.55×10^{-9}				
		**3a** Anion	2.0	1.31×10^{-9}	7.98×10^{-11}	4.85	478	0.998
**13**	CD_2Cl_2	**3a** Cation	2.0	1.14×10^{-9}	3.74×10^{-11}	5.42	667	0.997
		TMS		2.72×10^{-9}				
		**3a** Anion	3.0	1.11×10^{-9}	1.83×10^{-11}	5.19	587	0.999
**14**	CD_2Cl_2	**3a** Cation	3.0	1.01×10^{-9}	9.89×10^{-12}	5.61	739	0.999
		TMS		2.52×10^{-9}				
		**3a** Anion	5.0	1.06×10^{-9}	6.93×10^{-10}	5.47	687	0.999
**15**	CD_2Cl_2	**3a** Cation	5.0	1.02×10^{-9}	9.42×10^{-12}	5.52	703	0.999
		TMS		2.47×10^{-9}				
		**3a** Anion	0.5	1.89×10^{-9}	1.03×10^{-13}	4.33	340	0.998
**16**	CD_3CN	**3a** Cation	0.5	1.69×10^{-9}	2.05×10^{-10}	4.79	461	0.999
		TMS		3.23×10^{-9}				
		**3a** Anion	5.0	2.09×10^{-9}	9.12×10^{-11}	4.21	313	0.999
**17**	CD_3CN	**3a** Cation	5.0	1.51×10^{-9}	6.09×10^{-12}	5.48	689	0.999
		TMS		3.43×10^{-9}				

Table S17. Experimental self-diffusion coefficients D_i, viscosity corrected hydrodynamic radii r_H and resulting volumes V_A of ion pair **4a** in varying concentrations. TMS was used as viscosity reference for the experimental self-diffusion coefficients D_i to allow for a comparison of hydrodynamic radii r_H and resulting volumes V_A. The corresponding self-diffusion coefficients D_i of TMS are given for each sample. Entry 18-26: SW = 22 Hz, O1P = 10.0 ppm, gradient strength 5-95% linear. Samples were measured at room temperature.

Sample	solvent	Species inside sample	Concentration (mM)	Diffusion coefficient D_i ($m^2.s^{-1}$)	Standard deviation D_i ($m^2.s^{-1}$)	Hydrodynamic radius r_H (Å)	Volume V_A (Å^3)	R^2
18	CD_2Cl_2	**4a** Anion	0.005	1.41×10^{-9}	1.79×10^{-10}	4.13	296	0.996
		**4a** Cation	0.005	1.22×10^{-9}	1.27×10^{-11}	4.59	404	0.998
		TMS		2.36×10^{-9}				
19	CD_2Cl_2	**4a** Anion	0.01	1.37×10^{-9}	1.62×10^{-9}	4.49	379	0.987
		**4a** Cation	0.01	1.24×10^{-9}	8.24×10^{-11}	4.87	482	0.998
		TMS		2.58×10^{-9}				
20	CD_2Cl_2	**4a** Anion	0.05	1.17×10^{-9}	1.14×10^{-10}	5.00	525	0.998
		**4a** Cation	0.05	1.17×10^{-9}	3.43×10^{-12}	5.02	529	0.999
		TMS		2.53×10^{-9}				
21	CD_2Cl_2	**4a** Anion	0.1	1.09×10^{-9}	1.18×10^{-11}	5.29	619	0.999
		**4a** Cation	0.1	1.14×10^{-9}	3.03×10^{-12}	5.09	553	0.999
		TMS		2.52×10^{-9}				
22	CD_2Cl_2	**4a** Anion	0.2	1.06×10^{-9}	3.94×10^{-12}	5.44	672	0.999
		**4a** Cation	0.2	1.11×10^{-9}	2.75×10^{-12}	5.24	602	0.999
		TMS		2.53×10^{-9}				
23	CD_2Cl_2	**4a** Anion	0.4	1.02×10^{-9}	4.74×10^{-12}	5.49	692	0.999
		**4a** Cation	0.4	1.08×10^{-9}	2.87×10^{-12}	5.28	615	0.999
		TMS		2.48×10^{-9}				
24	CD_2Cl_2	**4a** Anion	0.6	1.00×10^{-9}	2.99×10^{-1}	5.58	729	0.999
		**4a** Cation	0.6	1.05×10^{-9}	2.64×10^{-12}	5.38	653	0.999
		TMS		2.49×10^{-9}				
25	CD_2Cl_2	**4a** Anion	0.8	9.90×10^{-10}	2.11×10^{-12}	5.71	778	0.999
		**4a** Cation	0.8	1.04×10^{-9}	3.87×10^{-12}	5.50	698	0.999
		TMS		2.52×10^{-9}				
26	CD_2Cl_2	**4a** Anion	1.0	9.84×10^{-10}	4.43×10^{-12}	5.67	765	0.999
		**4a** Cation	1.0	1.03×10^{-9}	2.92×10^{-11}	5.46	682	0.994
		TMS		2.49×10^{-9}				

Table S18. Experimental self-diffusion coefficients D_i, viscosity corrected hydrodynamic radii r_H and resulting volumes V_A of ion pair **3a**/**4a** while fixing the overall ion concentrations at 1.0 mM. PPh_4BF_4 was chosen as additive (Add) to keep the ion concentration at 1.0 mM. The ratio of **3a**/Add is given in percentage. TMS was used as viscosity reference for the experimental self-diffusion coefficients D_i to allow for a comparison of hydrodynamic radii r_H and resulting volumes V_A. The corresponding self-diffusion coefficients D_i of TMS are given for each sample. Entry 27-32: SW = 22 Hz, O1P = 10.0 ppm, gradient strength 5-95% linear. Samples were measured at room temperature.

Sample	solvent	Species inside sample	Concentration (mM)	3a/Add ratio	Diffusion coefficient D_i ($m^2.s^{-1}$)	Standard deviation D_i ($m^2.s^{-1}$)	Hydrodynamic radius r_H (Å)	Volume V_A (Å^3)	R^2
27	CD_2Cl_2	**3a** Anion	1.0	20% **3a** 80% Add	1.16×10^{-9}	1.78×10^{-11}	4.98	519	0.999
		**3a** Cation	1.0		1.07×10^{-9}	1.09×10^{-11}	5.32	631	0.999
		TMS			2.49×10^{-9}				
28	CD_2Cl_2	**3a** Anion	1.0	50% **3a** 50% Add	1.21×10^{-9}	3.35×10^{-11}	5.03	528	0.999
		**3a** Cation	1.0		1.09×10^{-9}	1.23×10^{-11}	5.44	673	0.999
		TMS			2.62×10^{-9}				
29	CD_2Cl_2	**3a** Anion	1.0	80% **3a** 20% Add	1.13×10^{-9}	3.16×10^{-12}	5.03	535	0.999
		**3a** Cation	1.0		1.04×10^{-9}	6.97×10^{-12}	5.39	656	0.998
		TMS			2.46×10^{-9}				
30	CD_2Cl_2	**4a** Anion	1.0	20% **4a** 80% Add	1.15×10^{-10}	7.39×10^{-11}	5.16	576	0.998
		**4a** Cation	1.0		1.07×10^{-9}	3.81×10^{-12}	5.50	698	0.999
		TMS			2.59×10^{-9}				
31	CD_2Cl_2	**4a** Anion	1.0	50% **4a** 50% Add	1.01×10^{-9}	1.20×10^{-10}	5.57	725	0.999
		**4a** Cation	1.0		1.06×10^{-9}	2.20×10^{-12}	5.36	646	0.999
		TMS			2.50×10^{-9}				
32	CD_2Cl_2	**4a** Anion	1.0	80% **4a** 20% Add	1.00×10^{-9}	2.27×10^{-12}	5.71	780	0.999
		**4a** Cation	1.0		1.06×10^{-9}	1.51×10^{-12}	5.46	680	0.999
		TMS			2.56×10^{-9}				

Cationic Sandwich Association Model

The resulting DOSY volumes for **3a** of Table S16 were plotted against the used concentration for both cation and anion (see Figure S9). First, for the lowest concentration 0.005 mM and 0.01 mM we were able to determine volumes close to the ones which were calculated for the free ions (cation: 362 Å^3 and anion: 215 Å^3, for calculation see Table S19). In a 1:1 association model, as the concentration is increased, the volumes of both ions should rise and gradually converge until they are identical at higher concentrations. Thereby, the anion volume would have to grow more rapidly with higher concentrations than the cation volume to result in an equal volume, as expected for a 1:1 aggregation.

Despite a clear increase of the volumes for both the cation **a** as well as of the anion **3** can at concentration higher than 0.01 mM, a large offset between the cation **a** and the anion **3** volume is observed. Notably, the cation **a** volume increases more than the anion **3** volume leading to an increasing offset between the two, which is the reverse of the expected behavior for a 1:1 model. These observations are in contrast to the initially assumed 1:1 association model the volumes of anion and cation should converge and approach 570 Å^3 for a monomeric ion pair **3a**. Additionally, in the concentration range of 0.4-1.0 mM a stagnation of the cation volume can be seen at a volume that even significantly exceeds the calculated volume for the 1:1 monomeric ion pair **3a** (649 Å^3 versus 570 Å^3). All these observations led to the assumption of another species that has to be involved.

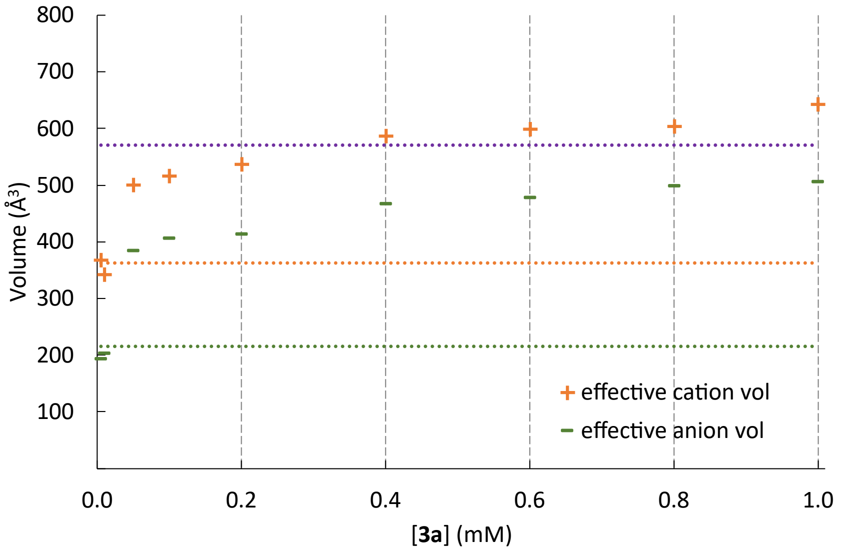

Figure S9. Concentration-dependent volumes of cation **a** (orange "+" symbols) and anion **3** (green "–" symbols) of compound **3a** in CD_2Cl_2 as calculated from DOSY experiments (with data from Table S16) with the respective calculated free ion volumes (dotted line; cation **a** in orange; anion **3** in green), and the calculated volumes of the 1:1 ion pair **3a** (purple dotted line).

To explain this deviation, we established a "sandwich association" model. In this model two cations **a** and one anion **3** form a "sandwich-cation" **a3a** (calculated volume of 925 Å^3, for calculation see Table S19) with an additional free anion **3** (calculated volume of 215 Å^3, for calculation see below). With this sandwich cation the increasing offset between cation **a** and anion **3** upon ion pair formation can be explained. The significantly smaller increase of the volume of anion **3** is in agreement with the additional free anion reducing the averaged DOSY volume. In addition, the significantly higher volumes of 642 Å^3 at higher concentrations can be explained by the formation of CAC cations which are included in the average volume of all cation species derived by DOSY.

In addition, the theoretical volumes of both free ions, the 1:1 monomeric ion pair **3a** as well as the sandwich model were calculated. These calculations were performed based on the van der Waals cavity used in the SMD solvation model at the B3LYP-D3/6-31+G(d) level of theory (see Table S19).

Table S19. Calculated volumes of all proposed species based on the van der Waals cavity used in the SMD solvation model at the B3LYP-D3/6-31+G(d) level of theory.

Species	Volume (Å^3)
Anion **3**	215
Anion **4**	285
Cation **a**	362
1:1 ion pair **3a**	570
Cation sandwich **a3a**	925
Anion sandwich **3a3**	782
1:1 ion pair **4a**	641
Cation sandwich **a4a**	995
Anion sandwich **4a4**	920

Based on these values we set up a two variable equation system which only considers an equilibrium between free ions (**3**/**a**) and the sandwich model **a3a**+**3** (S15). Using the calculated volumes of the free ions and the average measured volume of the cation in the concentration range between 0.4-1.0 mM leads to a ratio of 68:32 of free ions to sandwich cation/free anion.

$$\mathrm{vol_{cat}} = \frac{[\mathrm{C}]}{[\mathrm{IP}]_{\mathrm{tot}}} \times 362 + \left(\frac{2 \times [\mathrm{CAC}]}{[\mathrm{IP}]_{\mathrm{tot}}}\right) \times 925 \quad \text{(S15a)}$$

$$\mathrm{vol_{an}} = \frac{[\mathrm{A}]}{[\mathrm{IP}]_{\mathrm{tot}}} \times 215 + \left(\frac{[\mathrm{CAC}]}{[\mathrm{IP}]_{\mathrm{tot}}}\right) \times 925 \quad \text{(S15b)}$$

Besides this assumption, we also set up a three variable equation system which considers an equilibrium between free ions (**3**/**a**), monomeric 1:1 ion-pair **3a** and the sandwich model **a3a**+**3** (S16). Due to this system being a three variable two equation system, a clear ratio cannot be determined only based on DOSY. Nevertheless, various solutions are possible for this model (e. g. 20% free cation, 34% sandwich cation, 46% 1:1 ion pair). Hence, both models are fitting to the observed volumes while the 3 point-model system is slightly closer to the observed volumes.

$$(2 * (362 * x + 2 * 925 * y + 570 * z))/3 = 642 \quad \text{(S16a)}$$

$$(2 * (215 * x + 925 * y + 215 * y + 570 * z))/3 = 505 \quad \text{(S16b)}$$

The population of the free anion and free cation at a given overall salt concentration was calculated with the aid of the conductivity data (for details see SI chapter 3). Based on the calculated populations of the respective free ion, the theoretical DOSY volumes were simulated (see Figure S10).

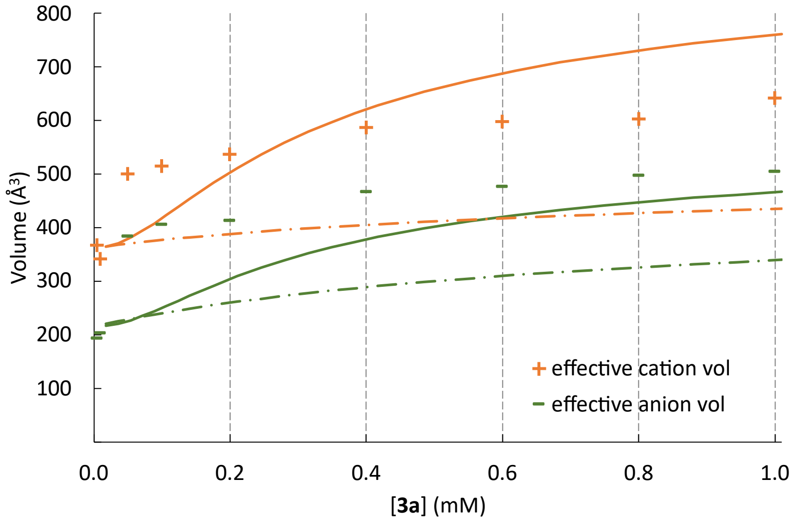

Figure S10. Concentration-dependent volumes of cation **a** (orange “+“ symbols) and anion **3** (green “–“ symbols) of compound **3a** in CD_2Cl_2 as calculated from DOSY experiments with the simulated trends for the cationic sandwich association model (orange line for cation **a**, green line for anion **3**), and the 1:1 ion association model (orange dotted-dashed line for cation **a**, green dotted-dashed line for anion **3**).

For both ions, the calculated trend for a 1:1 ion aggregation model leads to far lower volumes than observed by the DOSY model with discrepancies of up to 250 Å^3 (see Figure S10). In contrast, by the addition of the sandwich cation the calculated curves fit far better to the observed trends in volume (see Figure S10,12-14). Hence, the volumes cannot be explained by a single 1:1 aggregation but other species like in the sandwich model have to be considered.

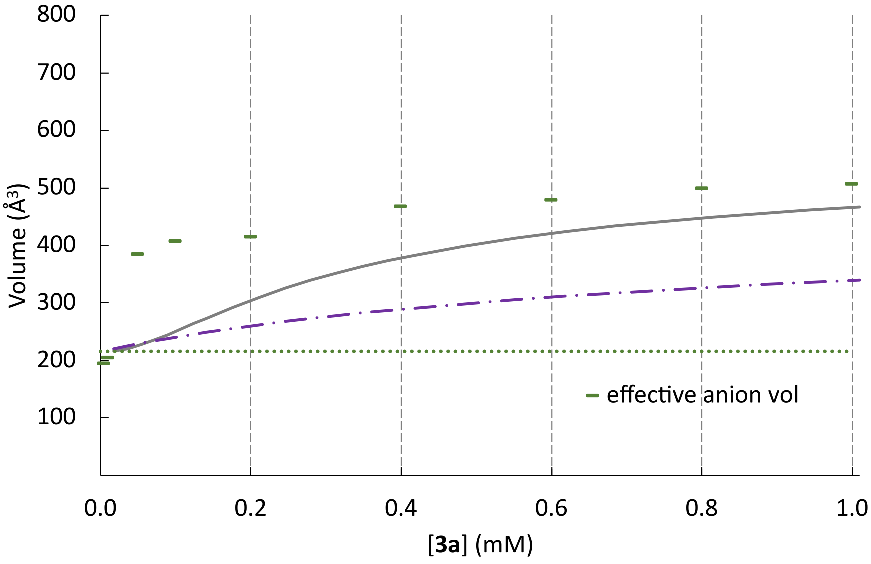

Figure S11. Concentration-dependent volumes of anion **3** (green “–“ symbols) of compound **3a** in CD_2Cl_2 as calculated from DOSY experiments with the free anion volume (green dotted line), the simulated trends for the 1:1 ion association model (purple dotted-dashed line), and cationic sandwich association model (grey line).

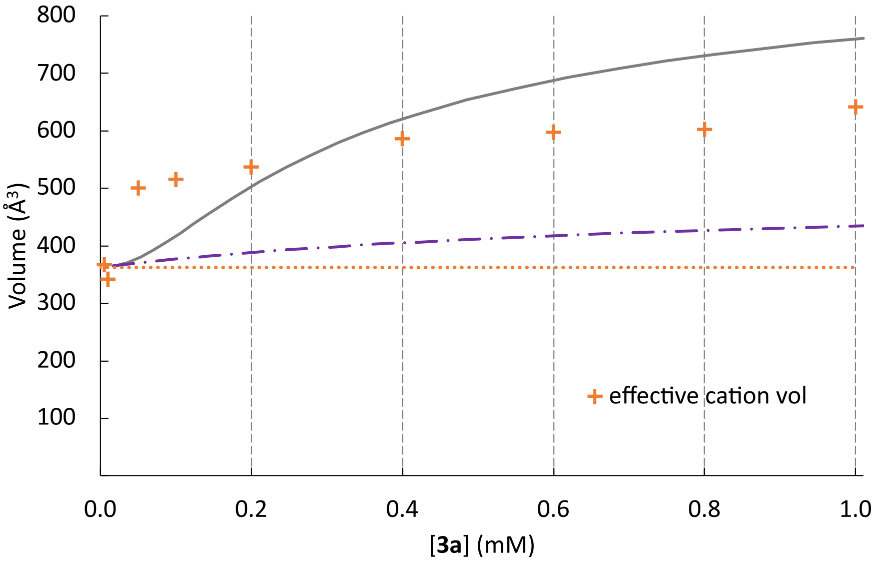

Figure S12. Concentration-dependent volumes of cation **a** (orange "+" symbols) of compound **3a** in CD_2Cl_2 as calculated from DOSY experiments with the free cation volume (orange dotted line), the simulated trends for the 1:1 ion association model (purple dotted-dashed line), and cationic sandwich association model (grey line).

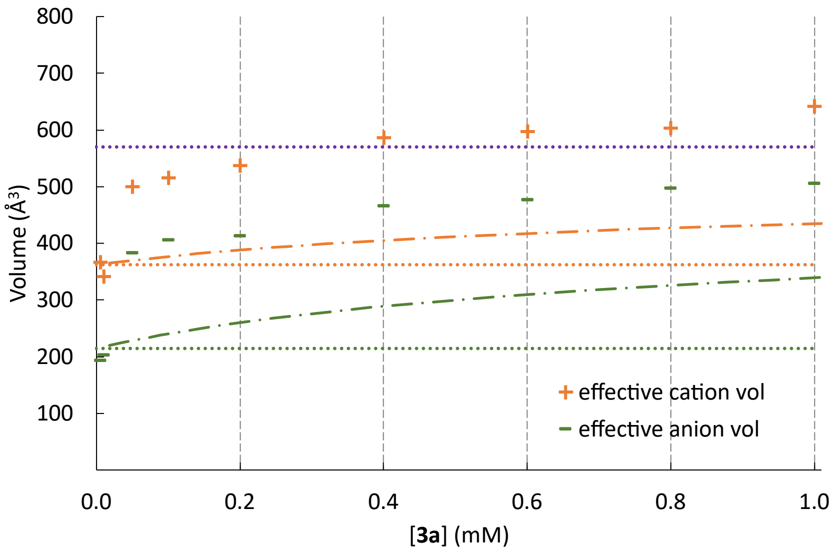

Figure S13. Concentration-dependent volumes of cation **a** (orange "+" symbols) and anion **3** (green "–" symbols) of compound **3a** in CD_2Cl_2 as calculated from DOSY experiments (with data from Table S16) with the respective calculated free ion volumes (dotted line; cation **a** in orange; anion **3** in green), the calculated volumes of the 1:1 ion pair **3a** (purple dotted line), the simulated trend for the 1:1 ion association model (orange and green dotted-dashed line).

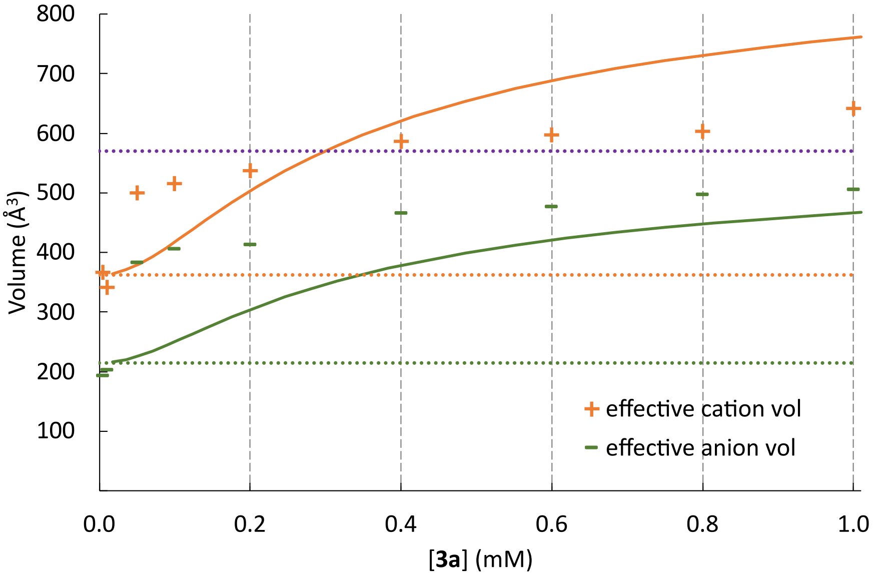

Figure S14. Concentration-dependent volumes of cation **a** (orange "+" symbols) and anion **3** (green "–" symbols) of compound **3a** in CD_2Cl_2 as calculated from DOSY experiments (with data from Table S16) with the respective calculated free ion volumes (dotted line; cation **a** in orange; anion **3** in green), the calculated volumes of the 1:1 ion pair **3a** (purple dotted line), the simulated trend for the cationic sandwich association model (orange and green line).

Anionic Sandwich Association Model

When plotting the DOSY volumes for **4a** against the concentration a different trend is observed, indicating the presence of another species. Here, contrary to **3a** the volume of anion **4** is increasing significantly more than the volume of cation **a** with increasing concentration until the volume of anion **4** surpasses the volume of cation **a**. At a concentration of 0.005 mM, the volumes determined for anion **4** (296 Å^3) and cation **a** (403 Å^3) are close to the calculated volumes of the free ions (anion: 285 Å^3 and cation: 360 Å^3, see Table S19). As the concentration increases, both the volumes of anion **4** and cation **a** rises, but the anion volume increases more rapidly than the cation volume. This leads to a crossing point at 0.05 mM were both volumes are nearly identical before the anion volume surpasses the volume of the cation **a** (**4**: 765 Å^3; **a**: 682 Å^3). Both the anion and cation volumes exceed the calculated volume of 1:1 ion pair **4a** (641 Å^3) which is depicted in Figure S15. These results cannot be explained by a 1:1 ion association nor by the cationic sandwich association. Thus, for salt **4a** a different type of aggregate has to be formed.

Considering the formation of the sandwich cation for salt **3a**, a similar higher aggregate in form of an sandwich anion is suggested for salt **4a** consisting of two anions **4** and one cation **a** in the sandwich anion complex **4a4** with a free cation **a**. The anionic sandwich association model explains the increase of the anion volume exceeding the cation volume and the resulting crossing point due to the formation of the sandwich anion, which increases the average DOSY volume of anion **4**. Additionally, the model explains the cation volume exceeding the calculated volume of the 1:1 ion pair **4a** as the respective DOSY volumes display an average of all cation **a** species in solution.

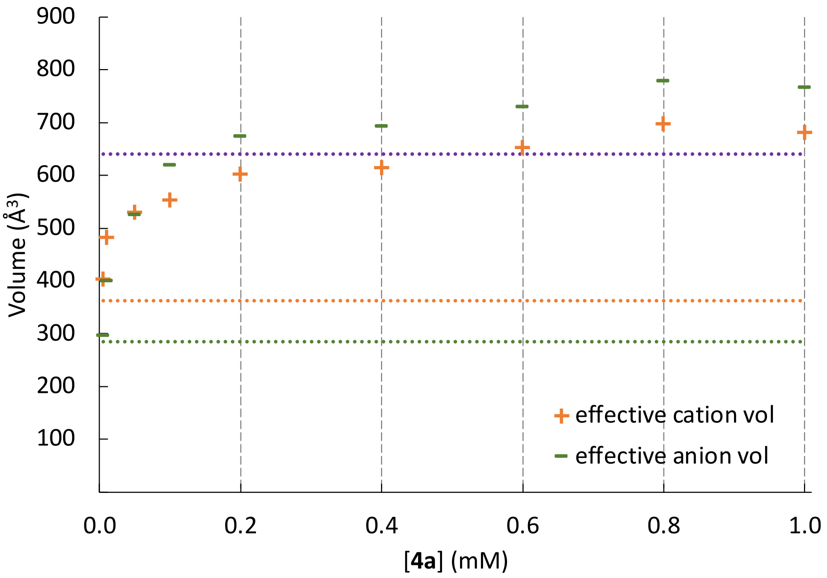

Figure S15. Concentration-dependent volumes of cation **a** (orange "+" symbols) and anion **4** (green "–" symbols) of compound **4a** in CD_2Cl_2 as calculated from DOSY experiments (with data from Table S16) with the respective calculated free ion volumes (dotted line; cation **a** in orange; anion **4** in green), and the calculated volumes of the 1:1 ion pair **4a** (purple dotted line).

Additional experiments with a fixed cation concentration at 1.0 mM were performed. Three different ion pair **4a** concentrations (0.2 mM, 0.5 mM, 0.8 mM) were investigated using additive **6** to maintain the overall ion concentration *I* at *I* = 1.0 mM (see Table S9, Entries 27-29). The anionic sandwich association model dictates that the anion volume should decrease with increasing proportion of additive **6** since the formation of the sandwich-anion **4a4** is less likely to occur with increasing cation concentration. And indeed, the anion volume decreases significantly (from 780 $Å^3$ to 576 $Å^3$) as the additive portion increases. Meanwhile, the cation volume remains relatively unchanged (680 $Å^3$ to 698 $Å^3$), leading to another crossing point where the cation volume exceeds that of the anion (see Figure S16). Thus, these findings support the anionic sandwich association model for ion pair **4a**.

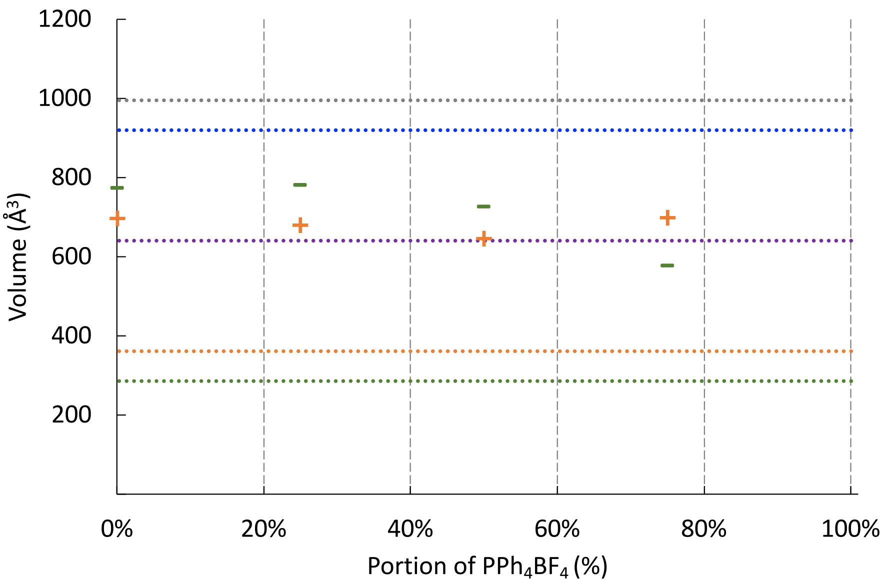

Figure S16. Concentration-dependent volumes of cation **a** (orange "+" symbols) and anion **4** (green "–" symbols) of **4a**/additive **6** mixtures with increasing amounts of **6** at *I* = 1.0 mM in CD_2Cl_2 as calculated from DOSY experiments with the respective calculated free ion volumes (dotted line; cation **a** in orange; anion **4** in green), and the calculated volumes of the 1:1 ion pair **4a** (purple dotted line), the sandwich cation **a4a** (grey dotted line), and the sandwich anion **4a4** (blue dotted line).

Furthermore, numerical simulations comparing the cationic sandwich association, the anionic sandwich association, the 1:1 ion association, and the mixed sandwich association were conducted and compared to the DOSY data (see Figure S17-19). For ion pair **4a**, the anionic sandwich association model and the mixed association model showed the best fit to the experimental data, while the cationic sandwich association model and the 1:1 ion association model showed significantly larger deviations. This further supports the presence of the sandwich anion **4a4** in solution. To conclude, the mixed sandwich association model where both types of sandwich complexes coexist with the anionic sandwich association being the predominate association pattern, gives the best fit between simulation and experimental data.

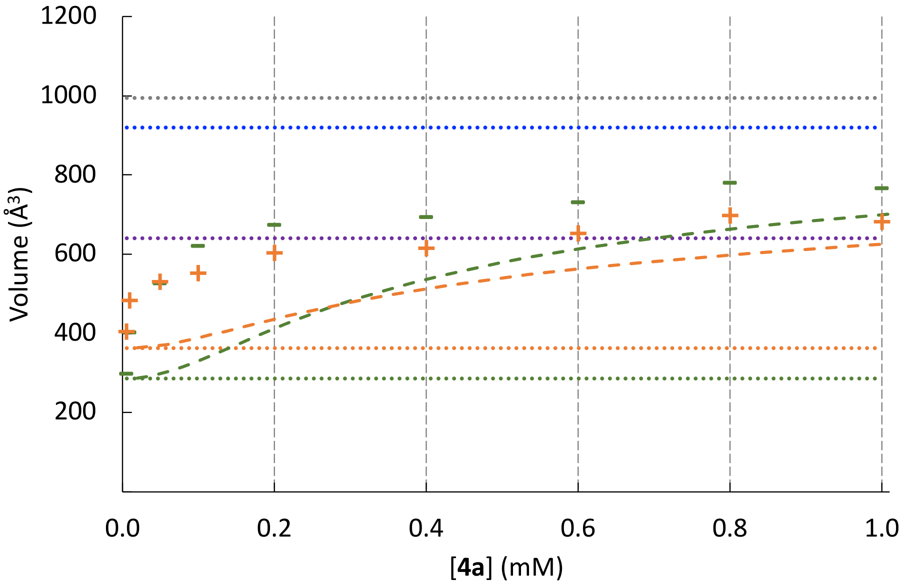

Figure S17. Concentration-dependent volumes of cation **a** (orange "+" symbols) and anion **4** (green "–" symbols) of salt **4a** in CD_2Cl_2 as calculated from DOSY experiments with the respective calculated free ion volumes (dotted line; cation **a** in orange; anion **4** in green), and the calculated volumes of the 1:1 ion pair **4a** (purple dotted line), the sandwich cation **a4a** (grey dotted line), the sandwich anion **4a4**(blue dotted line), and the simulated trend for the mixed sandwich association model (short-dashed line, cation trend in orange, anion trend in green).

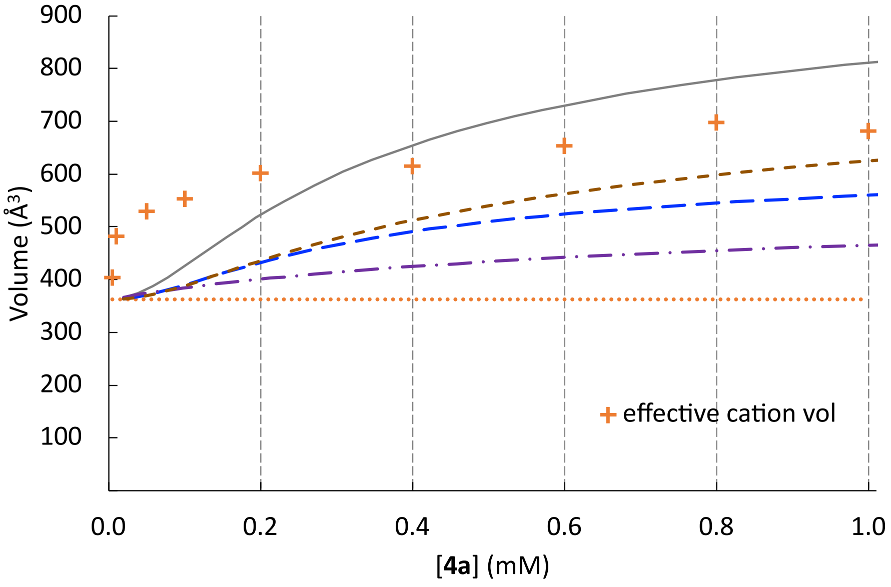

Figure S18. Concentration-dependent volumes of cation **a** (orange "+" symbols) of salt **4a** in CD_2Cl_2 as calculated from DOSY experiments with the free cation volume (orange dotted line), the simulated trends for the 1:1 ion association model (purple dotted-dashed line), the cationic (grey line) and anionic (blue dashed line) sandwich association model, and the mixed sandwich association model (brown short-dashed line).

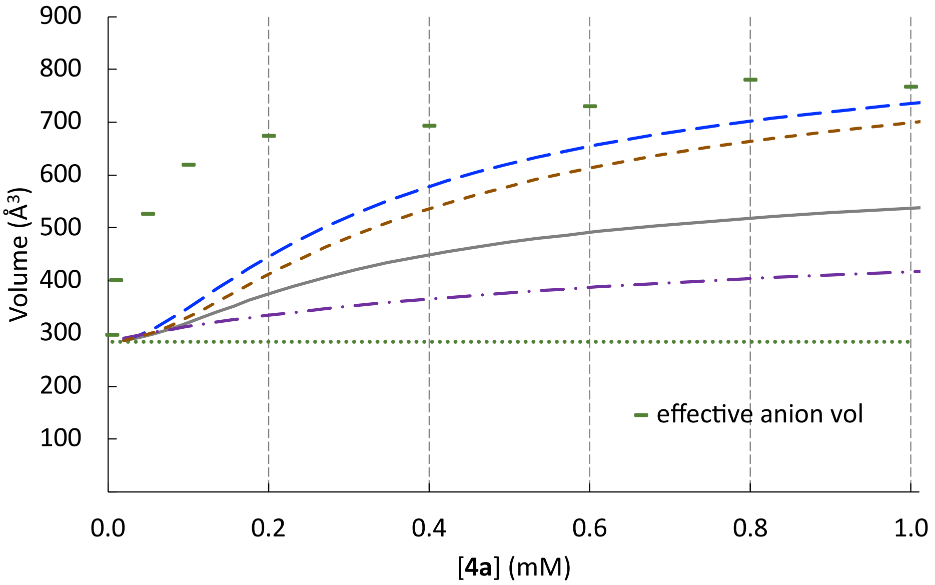

Figure S19. Concentration-dependent volumes of anion **4** (green "–" symbols) of salt **4a** in CD_2Cl_2 as calculated from DOSY experiments with the free anion volume (green dotted line), the simulated trends for the 1:1 ion association model (purple dotted-dashed line), the cationic (grey line) and anionic (blue dashed line) sandwich association model, and the mixed sandwich association model (brown short-dashed line).

Importance of the Ultrasonic Bath during NMR Sample Preparation

During initial investigations the NMR samples were prepared without using an ultrasonic bath. DOSY measurements of these samples showed high volumes for both cation and anion even at very low concentrations of **3a** (see Figure S20, Table S20). For a concentration of 0.001 mM volumes similar to the calculated free ions were determined for both cation **a** (392 Å^3) and anion **3** (239 Å^3). However, starting from 0.00166 mM a sudden increase of the cation volume to 528 Å^3 was observed. In addition, the derived volumes in a concentration range of 0.2 – 1.0 mM were varying significantly, especially for anion **3**.

Considering that the samples were prepared by diluting a stock solution starting from a concentration of 1.0 – 5.0 mM **3a**, we assumed that some ion-pair aggregates formed at this concentration remain even after dilution. Hence, the resulting volume was depending on the starting concentration of the stock solutions, explaining the variance in the concentration range of 0.2 – 1.0 mM as well as the high volumes at low concentrations. Indeed, repeating the measurements with a sample preparation that includes an ultrasonic bath during dilution solved both problems (see Figure S10). Thus, for a valid interpretation of the DOSY results for these pyridinamide ion-pairs the usage of an ultrasonic bath for the preparation of samples is essential.

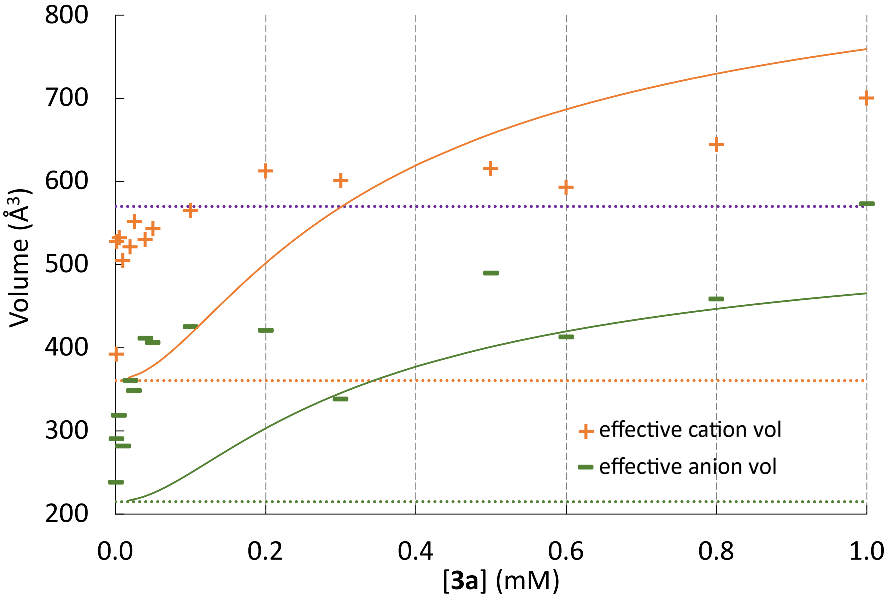

Figure S20. Concentration-dependent volumes of cation **a** (orange "+" symbols) and anion **3** (green "–" symbols) of compound **3a** in CD_2Cl_2 as calculated from DOSY experiments (with data from Table S20) with the respective calculated free ion volumes (dotted line; cation **a** in orange; anion **3** in green), the calculated volumes of the 1:1 ion pair **3a** (purple dotted line), and the simulated trend for the cationic sandwich association model (orange and green line).

Table S20. Experimental self-diffusion coefficients Di, viscosity corrected hydrodynamic radii rH and resulting volumes VA of ion pair **3a** when preparing the samples without an ultrasonic bath. TMS was used as viscosity reference for the experimental self-diffusion coefficients Di to allow for a comparison of hydrodynamic radii rH and resulting volumes VA. The corresponding self-diffusion coefficients Di of TMS are given for each sample. Entry 1-20: SW = 22 Hz, O1P = 10.0 ppm, gradient strength 5-95% linear. Samples were measured at room temperature.

**Sample**	**solvent**	**Species inside sample**	**Concentration (mM)**	**Diffusion coefficient D_i ($m^2.s^{-1}$)**	**Standard deviation D_i ($m^2.s^{-1}$)**	**Hydrodynamic radius r_H (Å)**	**Volume V_A (Å³)**	**R^2**
**1**	CD_2Cl_2	**3a** Anion<br>**3a** Cation<br>TMS	0.001<br>0.001	1.65 x 10^{-9}<br>1.31 x 10^{-9}<br>2.49 x 10^{-9}	2.08E-11<br>4.33E-11	3.85<br>4.54	239<br>392	0.997<br>0.999
**2**	CD_2Cl_2	**3a** Anion<br>**3a** Cation<br>TMS	0.00166<br>0.00166	1.53 x 10^{-9}<br>1.17 x 10^{-9}<br>2.54 x 10^{-9}	1.94E-11<br>4.13E-11	4.11<br>5.01	290<br>528	0.995<br>0.999
**3**	CD_2Cl_2	**3a** Anion<br>**3a** Cation<br>TMS	0.005<br>0.005	1.47 x 10^{-9}<br>1.17 x 10^{-9}<br>2.55 x 10^{-9}	6.37E-11<br>1.86E-11	4.24<br>5.03	319<br>532	0.995<br>0.999
**4**	CD_2Cl_2	**3a** Anion<br>**3a** Cation<br>TMS	0.01<br>0.01	1.56 x 10^{-9}<br>1.20 x 10^{-9}<br>2.55 x 10^{-9}	1.76E-11<br>3.63E-11	4.07<br>4.94	282<br>505	0.996<br>0.999
**5**	CD_2Cl_2	**3a** Anion<br>**3a** Cation<br>TMS	0.02<br>0.02	1.37 x 10^{-9}<br>1.17 x 10^{-9}<br>3.50 x 10^{-9}	1.49E-12<br>1.06E-11	4.41<br>4.99	360<br>521	0.993<br>0.999
**6**	CD_2Cl_2	**3a** Anion<br>**3a** Cation<br>TMS	0.025<br>0.025	1.41 x 10^{-9}<br>1.15 x 10^{-9}<br>2.53 x 10^{-9}	8.62E-11<br>7.14E-12	4.37<br>5.09	349<br>552	0.988<br>0.998
7	CD_2Cl_2	**3a** Anion<br>**3a** Cation<br>TMS	0.04<br>0.04	1.28 x 10^{-9}<br>1.14 x 10^{-9}<br>2.48 x 10^{-9}	1.80E-11<br>5.93E-12	4.61<br>5.02	411<br>530	0.995<br>0.999
8	CD_2Cl_2	**3a** Anion<br>**3a** Cation<br>TMS	0.05<br>0.05	1.27 x 10^{-9}<br>1.13 x 10^{-9}<br>2.46 x 10^{-9}	1.22E-10<br>9.88E-12	4.59<br>5.06	406<br>543	0.999<br>0.999
9	CD_2Cl_2	**3a** Anion<br>**3a** Cation<br>TMS	0.1<br>0.1	1.29 x 10^{-9}<br>1.14 x 10^{-9}<br>2.54 x 10^{-9}	1.09E-10<br>2.85E-11	4.66<br>5.13	425<br>565	0.999<br>0.999
10	CD_2Cl_2	**3a** Anion	0.2	1.30 x 10^{-9}	7.57E-11	4.65	421	0.999

		**3a** Cation<br>TMS	0.2	1.11 x 10^{-9}<br>2.56 x 10^{-9}	1.47E-11	5.27	613	0.999
11	CD_2Cl_2	**3a** Anion<br>**3a** Cation<br>TMS	0.3<br>0.3	1.45 x 10^{-9}<br>1.13 x 10^{-9}<br>2.59 x 10^{-9}	5.53E-11<br>2.13E-11	4.32<br>5.24	338<br>601	0.998<br>0.999
12	CD_2Cl_2	**3a** Anion<br>**3a** Cation<br>TMS	0.5<br>0.5	1.21 x 10^{-9}<br>1.09 x 10^{-9}<br>2.53 x 10^{-9}	5.40E-12<br>1.12E-11	4.89<br>5.28	490<br>616	0.999<br>0.999
13	CD_2Cl_2	**3a** Anion<br>**3a** Cation<br>TMS	0.6<br>0.6	1.30 x 10^{-9}<br>1.11 x 10^{-9}<br>2.53 x 10^{-9}	7.70E-11<br>2.60E-11	4.62<br>5.21	413<br>593	0.999<br>0.999
14	CD_2Cl_2	**3a** Anion<br>**3a** Cation<br>TMS	0.8<br>0.8	1.24 x 10^{-9}<br>1.07 x 10^{-9}<br>2.52 x 10^{-9}	3.98E-11<br>1.05E-11	4.78<br>5.36	459<br>645	0.999<br>0.999
15	CD_2Cl_2	**3a** Anion<br>**3a** Cation<br>TMS	1.0<br>1.0	1.13 x 10^{-9}<br>1.04 x 10^{-9}<br>2.53 x 10^{-9}	8.36E-11<br>2.71E-11	5.15<br>5.51	573<br>700	0.999<br>0.999

5. Characterization of Additive PPh_4BF_4 (6)

The “wide range” measurement for **3a** were performed with the total ion concentration in solution being kept constant by the addition of an additive that remains structurally as close as possible to catalyst **3a**. Therefore, tetraphenylphosphonium tetrafluoroborate (PPh_4BF_4, **6**) was synthesized by salt metathesis from tetraphenylphosphonium bromide with sodium tetrafluoroborate in water. After extraction with DCM, the final product was obtained by precipitation from DCM with toluene with 88% yield.

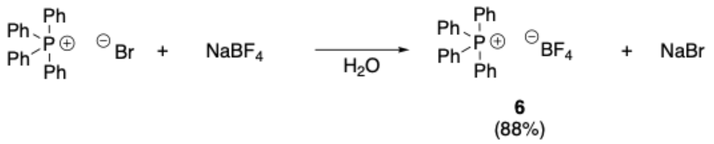

Scheme S7. Synthesis of tetraphenylphosphonium tetrafluoroborate PPh_4BF_4 (**6**).

Additive **6** was examined with conductivity in MeCN and DCM and the results are shown in Figure S21. In both solvents, additive **6** shows a slightly higher conductivity than ion pair catalyst **3a**.

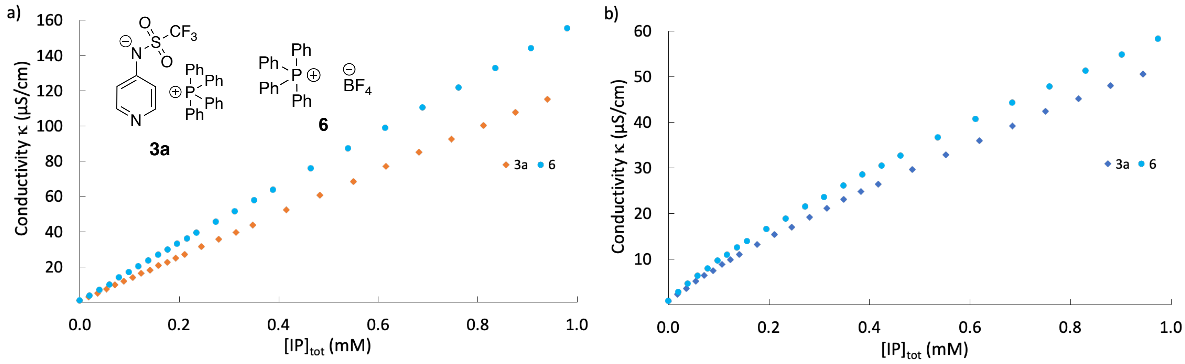

Figure S21. a) Concentration-dependent conductivity profile for additive **6** and catalyst **3a** in MeCN at 20 °C, b) Concentration-dependent conductivity profile for additive **6** and catalyst **3a** in DCM at 20 °C.

The conductivity profile of **6** was used to determine the corresponding association constants *K* (Table S21) which were obtained by employing eq. S6, the double ion pairing model as well as the sandwich association model 3a (see Chapter 3). For the determination of the sandwich association constant K_{CAC}, it is again crucial to assess the contribution of the single ion towards the molar conductivity Λ_m, gained by linear extrapolation of the initial three data points.

Table S21. Association constants K_{IP} in different solvents according to eq. S6, double K_{IP}, and K_{CAC} according to model 3a of pyridinamide ion pairs **3a**, **4a**, and additive **6** and at 20 °C.

**Ion Pair**	**K_{IP} (M^{-1})** MeCN [45.6][a]	DCM [40.7][a]	**K_{IP} (M^{-2})** DCM [40.7][a]	**K_{CAC} (M^{-2})** DCM [40.7][a]
**3a**	34.6	828	6.86 x 10^5	6.38 x 10^6
**4a**	42.9	938	8.79 x 10^5	6.50 x 10^6
**6**	45.5	1004	1.01 x 10^6	7.05 x 10^6

[a] $E_T(30)$ [kcal mol^{-1}] solvent polarity parameters taken from Reichardt[4].

Next, additive **6** was employed in the wide range measurement of **3a** in DCM. Except for the addition of **6** as a second stock solution, the method was not changed further. In a first step, the overall ionic concentration was set to be 1.0 mM for all eleven data point of this measurement (Figure S22, turquoise diamonds). Now, instead of the previously measured non-linear correlation between $[IP]_{tot}$ and k_{obs} a linear correlation is observed.

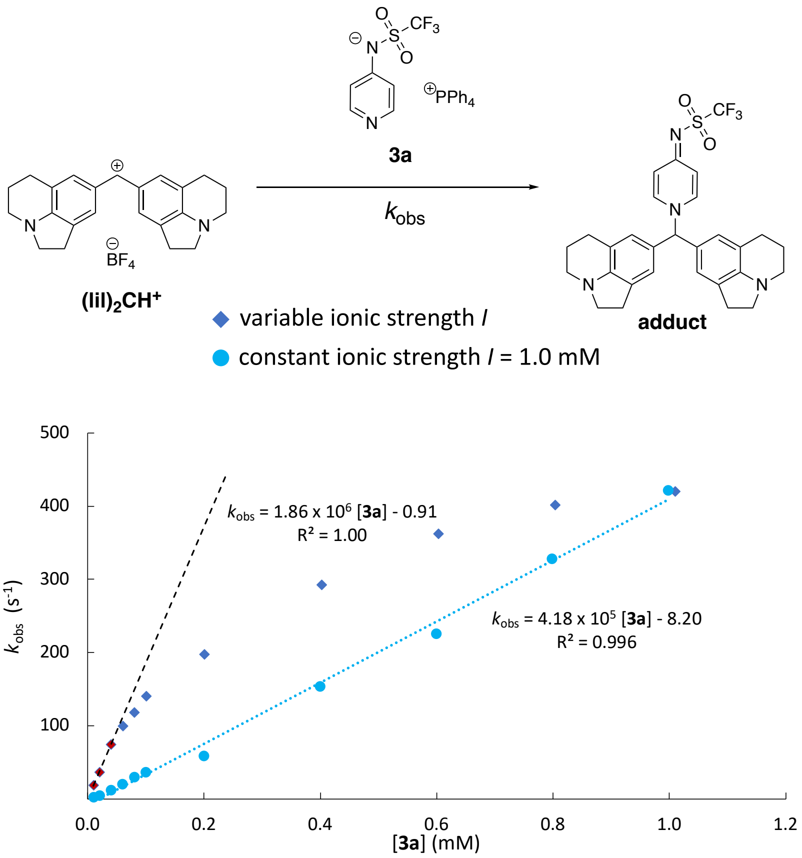

Figure S22. Plot of k_{obs} vs. concentration of catalysts [**3a**] over a concentration range from 0.01–1.0 mM in DCM at 20°C (blue diamonds), and with the additive PPh_4BF_4 (**6**) with a total ionic strength I = 1.0 mM (turquoise points).

Now, there are two ways to interpret this data: A) The association equilibrium of **3a** is pushed to the product side by the addition of additive **6** while the solvent polarity remains unchanged. B) The solvent polarity is changed by the addition of additive **6**, indicating that the second-order reaction rate k_2 and the association constant K_{IP} is depending on the concentration of salt **6**. Which one of the assumptions holds true will be checked with DOSY measurement as well as $E_T(30)$ value measurements of DCM at different salt concentrations.

First, the solvent polarity was determined by using the Reichardt‘s Dye Betaine $E_T(30)$. The $E_T(30)$ dye was dissolved in dry DCM and mixed with stock solutions of the respective salt. The UV/Vis spectrum was measured. The $E_T(30)$ values of the mixture were calculated according to eq. S17 in which h is the Planck‘s constant, c is the speed of light, and N_A is the Avogadro‘s constant.

$$E_T = \frac{hcN_A}{\lambda_{max}} = \frac{28951}{\lambda_{max}} \quad (S17)$$

A gradual increase in the $E_T(30)$ values can be observed for DCM with increasing concentrations of **3a** and **6**, respectively. The results are listed in Table S22.

Table S22. Structure of Betaine $E_T(30)$ with a list of measured λ_{max} of Betaine respective with ion pair **3a** and additive **6** in DCM.

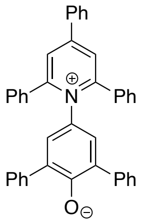

Betaine $E_T(30)$

Salt (mM)	λ_{max} (nm) with 3a	$E_T(30)$ (kcal mol^{-1})	λ_{max} (nm) with 6	$E_T(30)$ (kcal mol^{-1})
0.1	698	41.0	698	41.0
0.5	698	41.0	698	41.0
1.0	697	41.0	700	40.9
2.0	696	41.1	700	40.9
3.0	698	41.1	699	40.9
4.0	694	41.2	694	41.2
5.0	694	41.2	–	–
6.0	696	41.1	–	–

As a second measure, DOSY measurements were utilized to determine the association state at an overall salt concentration of 1.0 mM with an increasing portion of ion pair **3a** in solution (Table S23).

Table S23. Viscosity corrected hydrodynamic radii r_H and resulting volumes of ion pair **3a** while fixing the overall ion concentrations at 1.0 mM. PPh_4BF_4 was chosen as additive (Add) to keep the ion concentration at 1.0 mM. The ratio of **3a**/Add is given in percentage. TMS was used as viscosity reference for the experimental self-diffusion coefficients D_i to allow for a comparison of hydrodynamic radii r_H and resulting volumes V_A (see Table S8). Entry 21-23: SW = 22 Hz, O1P = 10.0 ppm, gradient strength 5-95% linear. Samples were measured at room temperature.

	[3a] (mM)	Radius (Å)	Volume (Å^3)
**Cation**	1.0	5.51	642[a]
**Anion**	1.0	5.15	505[a]
	0.2	5.32	631
**Cation**	0.5	5.44	673
	0.8	5.39	656
	0.2	4.98	519
**Anion**	0.5	5.03	528
	0.8	5.03	535

[a] averaged value based on entry 9-12 in Table S16, Chapter 4.

To summarize, the measured volumes of the anion at an overall salt concentration of 1.0 mM are located in the same region as the anion volume of pure **3a** at 1.0 mM. So, by keeping a constant salt concentration throughout the measurement through the addition of additive **6**, the association of the respective pyridinamide ion pair can be “frozen” at a certain association state.

6. Nucleophilicity Data

The rates of the reactions of ion pair catalysts **3a** and **4a** as well as of **TCAP** with the reference electrophiles were measured photometrically on a stopped-flow spectrophotometer. The temperature was controlled with a circulating bath cryostat. The reactions were carried out under pseudo-first-order conditions (ion pair = nucleophile, excess compound) and monitored at the absorption maximum of benzhydrylium ion in the respective solvent as described previously by the H. Mayr group.[22] First-order rate constants k_{obs} (s^{-1}) were derived by fitting the absorbance decay according to the mono-exponential function $A_t = A_0 \exp(-k_{obs}\, t) + C$ (see Figure S23).

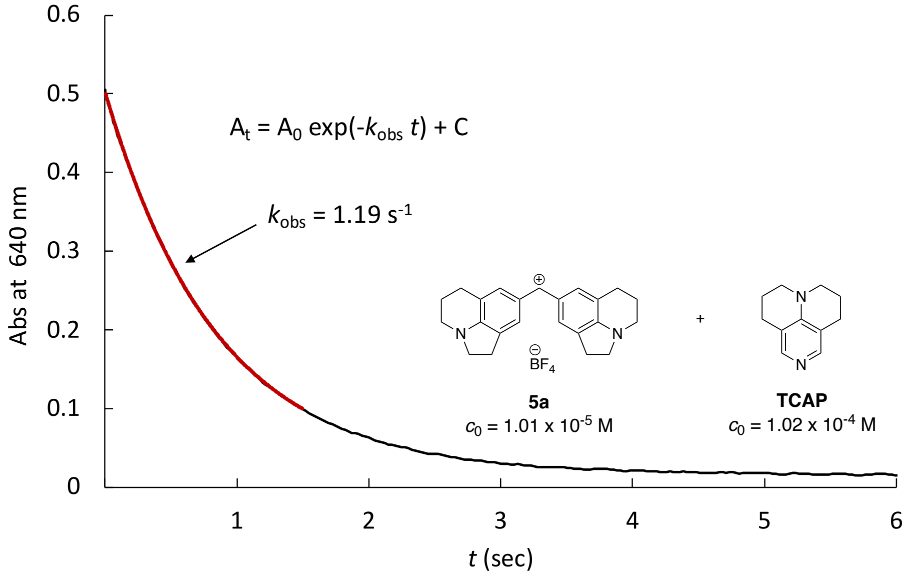

Figure S23. Absorbance decay of benzhydrylium salt **5a** reacting with the nucleophile TCAP (**2**) in DCM at 20°C, fitted by a mono-exponential decay function resulting in $k_{obs} = 1.19\ s^{-1}$.

Second-order rate constants k_2 ($L\ mol^{-1}\ s^{-1}$) were obtained from the slope of the linear plots of k_{obs} (s^{-1}) vs. [Nu] since $k_{obs} = k_2[Nu]$. The subsequent characterization is based on the Mayr-Patz eq. (S18) where the second-order rate constants ($\log k_2^{20\,°C}$) for the reaction of the nucleophile with the electrophile is expressed as a function of the nucleophilicity parameter N, the nucleophile-associated sensitivity parameter s_N, and the electrophilicity parameter E. Both, N and s_N parameter are solvent dependent.[22–24]

$$\log k_2^{20\,°C} = s_N(N + E) \quad \text{(S18)}$$

6.1 Mayr‘s Benzhydrylium Method – Results in MeCN and DCM at *c* = 0.01–0.03 mM

Mayr’s benzhydrylium method is applicable for dissociated ion pairs, therefore, the data gathered in MeCN can be easily analyzed according to the standard procedure. Measurements done in DCM require more consideration for the analysis since ion association affects the measurements. Salt **3a** will be used as reference system to discuss any new model and data analysis procedures. To circumvent the effects of association, initial measurements in DCM were done between 0.01 – 0.03 mM where salt **3a** is assumed to be fully dissociated into the free anion **3** and cation **a**, with anion **3** being the only nucleophilic species in solution. The total amount of the fully dissociated **3a** in solution is annotated as $[\mathbf{3}]_{tot}$, meaning that the weighed amount of **3a** = amount of free anion **3** in solution. Kinetic data obtained using Mayr‘s benzhydrylium method are summarized in Table S24.

Table S24. Second-order rate constants for the reactions of **3a** with $(lil)_2CHBF_4$ (**5a**), $(jul)_2CHBF_4$ (**5b**), and $(ind)_2CHBF_4$ (**5c**) determined in the concentration range $[\mathbf{3}]_{tot}$ = 0.01 – 0.03 mM in DCM (blue diamonds) and MeCN (orange dots) at 20 °C and the corresponding *N*- and s_N-parameters.

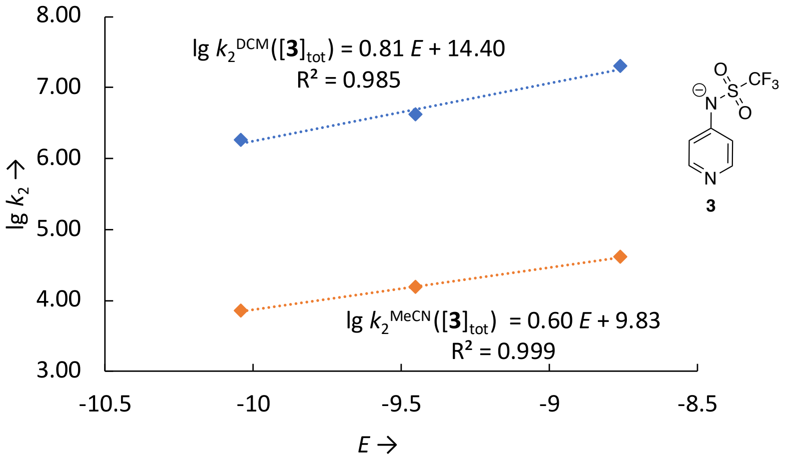

**Solvent**	**Nucleo-phile**	***k*₂ (M^{-1} s^{-1})**			***N*/*s*ₙ**
		**5a**	**5b**	**5c**	
MeCN	$[\mathbf{3}]_{tot}$	7.16 x 10^3	1.53 x 10^4	4.13 x 10^4	16.38/0.60
DCM	$[\mathbf{3}]_{tot}$	1.82 x 10^6	4.23 x 10^6	1.98 x 10^7	17.75/0.81

In MeCN, the correlation between k_{obs} for **3** + **5** reactions and the total concentration of **3a** in solution are linear (see Table S24). This is due to the sufficient polarity of MeCN to dissociate salts into the corresponding free anions and cations. However, this stabilizing influence also results in a considerably lower nucleophilicity of **3** when compared to its measurement in DCM. In DCM, the nucleophilic anion **3** experiences less solvation, rendering it more readily available to react with electrophiles **5**. Consequently, the second-order rate constant k_2 for **3** + **5** reactions are almost three orders of magnitude higher in DCM than in MeCN.

This analysis was made under the assumption that **3a** is mainly, if not fully, dissociated in DCM at concentrations below 0.03 mM. A way to check this assumption is using numerical simulations according to the cationic sandwich association model (see Chapter 3.4) to determine how much free anion **3** is present in solution. We know the total salt concentration of **3a** at each data point, so this concentration is given as the initial concentration of the anion and cation concentration in COPASI. In the section "reaction", the rate constant k_1 is set to the determined association constant K_{CAC} while the rate constant k_2 is fixed to 1. Then the function "Steady State" is used to get the concentration for each species at the given concentration. This process is repeated for each salt concentration of **3a**. Now the first-order rate constant k_{obs} can be plotted against the anion concentration [**3**] to get the second-order rate constant k_2 for the nucleophilic anion **3** in solution. This leads to the listed results in Table S25.

Table S25. Second-order rate constants for the reactions of **3a** in the full dissociation model (where [**3**] = [**3a**]$_{tot}$, blue diamonds) and the sandwich association model (purple dots) with benzhydrylium electrophile salts $(lil)_2CHBF_4$ (**5a**), $(jul)_2CHBF_4$ (**5b**), and $(ind)_2CHBF_4$ (**5c**) between 0.01 – 0.03 mM in DCM at 20 °C and the corresponding *N*- and s_N-parameters.

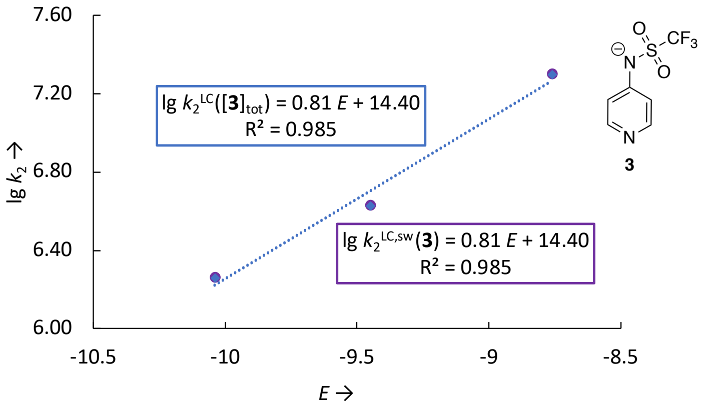

Conditions	Superscript	Nucleophile	k_2 (M^{-1} s^{-1})			*N*/s_N
			5a	5b	5c	
I = 0.01-0.03 mM	LC	[**3**]$_{tot}$	1.84 x 10^6[a]	4.23 x 10^6	1.98 x 10^7	17.78/0.81
Sandwich model	LC,sw	[**3**]	1.84 x 10^6	4.28 x 10^6	2.00 x 10^7	17.78/0.81

[a] the reaction of **3a** with **5a** was measured twice, resulting in k_2^{LC} = 1.82 x 10^6 M^{-1} s^{-1} (*I* = 0.01-0.03 mM) and k_2^{LC} = 1.86 x 10^6 M^{-1} s^{-1} (*I* = 0.01-0.04 mM, initial three data points of wide range measurement) giving an average k_2^{LC} = 1.84 x 10^6 M^{-1} s^{-1}.

At low salt concentrations, where [**3a**] = 0.01 – 0.03 mM in DCM (superscript LC), the second-order rate constant k_2^{LC} were obtained by assuming that the total salt concentration [**3**]$_{tot}$ = the free anion concentration [**3**]. Additionally, second-order rate constant $k_2^{LC,sw}$ were determined by using the free anion concentration [**3**] obtained with numerical simulations. The resulting rate constant k_2^{LC} and $k_2^{LC,sw}$ revealed only minor differences and identical *N* and s_N parameters. We conclude that at low concentrations salt **3a** is mainly dissociated, and the assumption of [**3**] = [**3**]$_{tot}$ is valid.

By applying the cationic sandwich model (model 3a) to the kinetic data of the wide range measurement performed in DCM over a wider concentration range (0.01 – 1.0 mM), the concentration of free anion **3** was determined and plotted against the pseudo-first order rate constant k_{obs}. The resulting correlation between k_{obs} and anion **3** concentration was expected to be linear and following the black dashed trendline (see Figure S24).

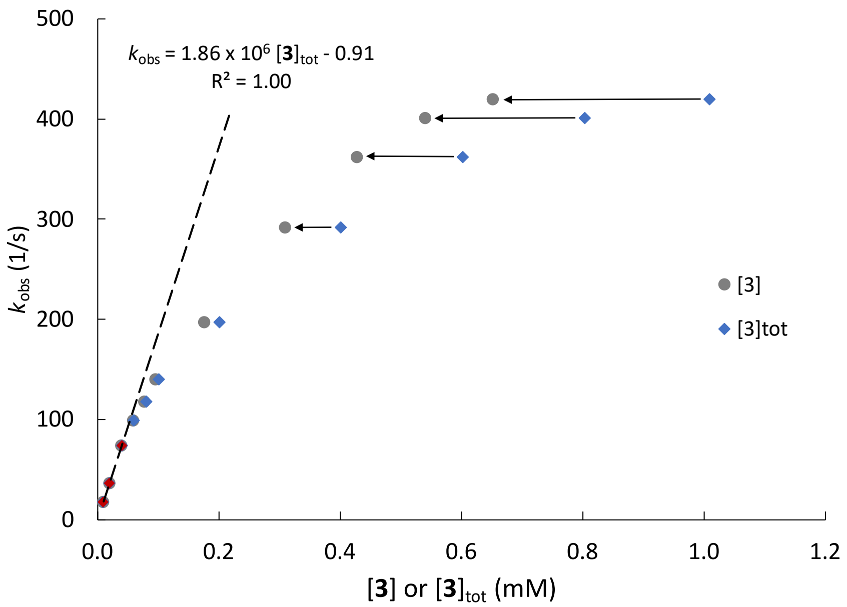

Figure S24. Plot of k_{obs} vs. concentration of catalysts **3a** over a concentration range from 0.01–1.0 mM in DCM at 20°C (blue diamonds), and vs. concentration of anion **3** over a concentration range from 0.01–1.0 mM in DCM at 20°C (grey dots).

Instead, the result is a steeper, but still non-linear correlation indicating that the association of **3a** is not the only process that influences the kinetic measurements. Assuming that the change in overall ionic strength when going from 0.01 mM to 1.0 mM salt concentrations may be responsible for this non-linear behavior, rate measurements were repeated in the presence of $PPh_4^+BF_4^-$ (**6**). The additive was added to maintain a constant ionic strength of 1.0 mM over all pyridinamide salt concentrations. The choice of salt **6** combines cation **a** of **3a** with the BF_4^- counter-anion of the benzhydrylium electrophile salts. Addition of **6** increases the concentration of phosphonium cation **a** and thus the concentration of sandwich cation **a3a**, resulting in a linear correlation of the first-order rate constant k_{obs} and the nucleophile concentration (see Figure S25, turquoise points).

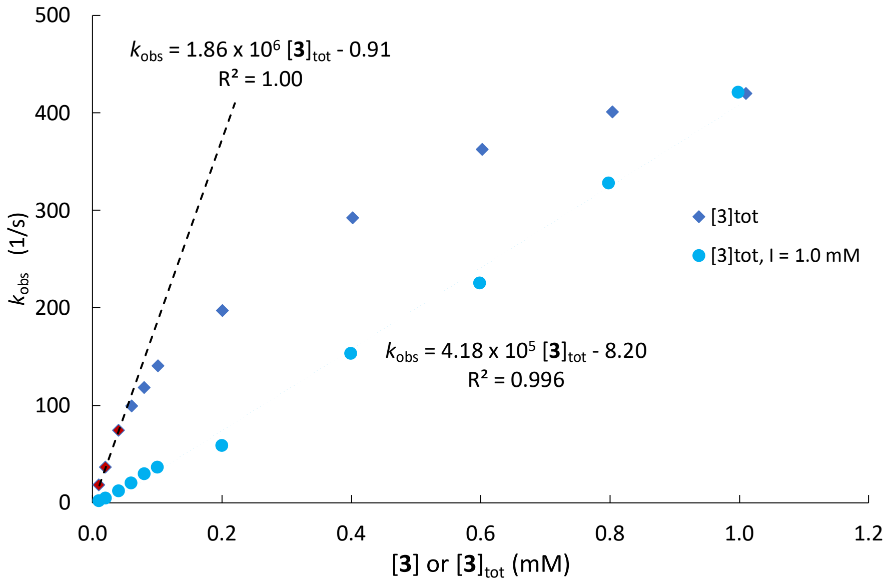

Figure S25. Plot of k_{obs} vs. concentration of catalysts **3a** over a concentration range from 0.01–1.0 mM in DCM at 20°C (blue diamonds), and with additive PPh_4BF_4 (**6**) with a $[IP]_{tot}$ = 1.0 mM (turquoise points).

6.2 Cationic Sandwich Association Extension

The measurements done with the addition of additive **6** can be analyzed in multiple ways. The first way would be to assume that the amount of weighted salt **3a** equals the amount of nucleophile in solution. Here, we would simply use the standard Mayr's benzhydrylium method to obtain the second-order rate constant k_2 and subsequently gain the N parameter and s_N parameter (see Table S26).

From the combination of conductivity and DOSY measurements, we know that the salt **3a** associates into triple ionic adducts, so-called sandwich cations (see Chapter 4). Therefore, the weighted amount of salt **3a** is most likely not equivalent to the concentration of the reactive nucleophile in solution. We assume here that the sandwich complex does not react as a nucleophile, but rather only the free anion **3**.

Again, we use the earlier established association model 3a for the cationic sandwich association with $K_{CAC} = k_1/k_2 = 6.38 \times 10^6\ M^{-2}$. Except now, we have to add one more reaction to the biochemical model for the sandwich association of **3a** in COPASI to incorporate the influence of additive **6**. The additive can either associate according to the 1:1 model or according to the sandwich model (see eq. S19)

$$2 * C + 2 * B \xrightleftharpoons{K_{IP}(add)} 2 * CB \qquad \text{(S19a)}$$

$$2 * C + 2 * B \xrightleftharpoons{K_{CAC}(add)} B + CBC \qquad \text{(S19b)}$$

Depending on the chosen association type, either eq. S19a or eq. S19b was added to the biochemical model as "additive association" with **C** = PPh_4^+, **B** = BF_4^-, and **CD** = PPh_4BF_4 (**6**) or the sandwich adduct **CBC** = $PPh_4BF_4PPh_4$.

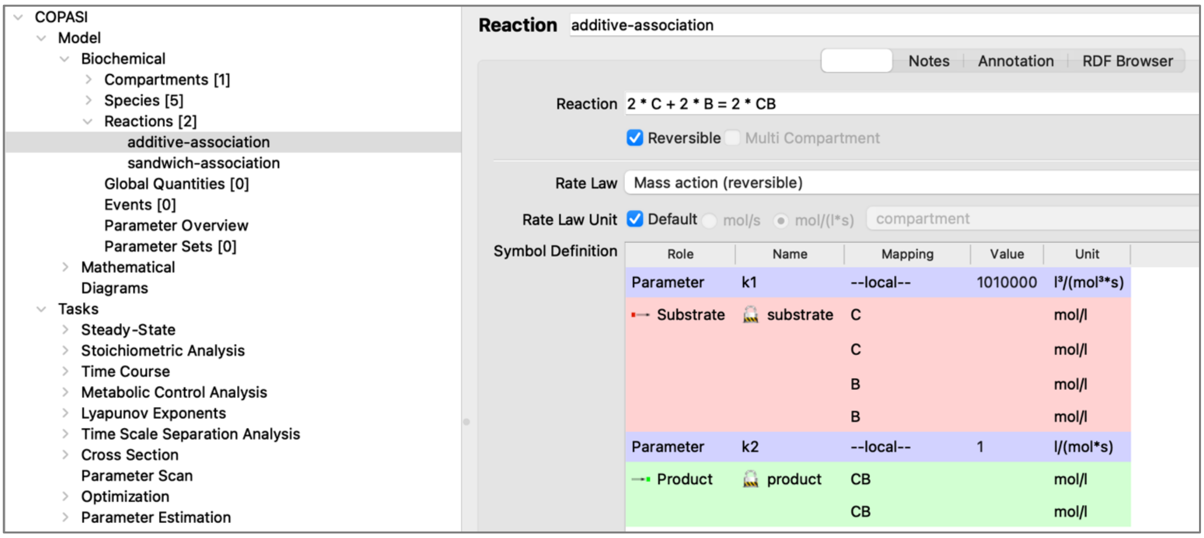

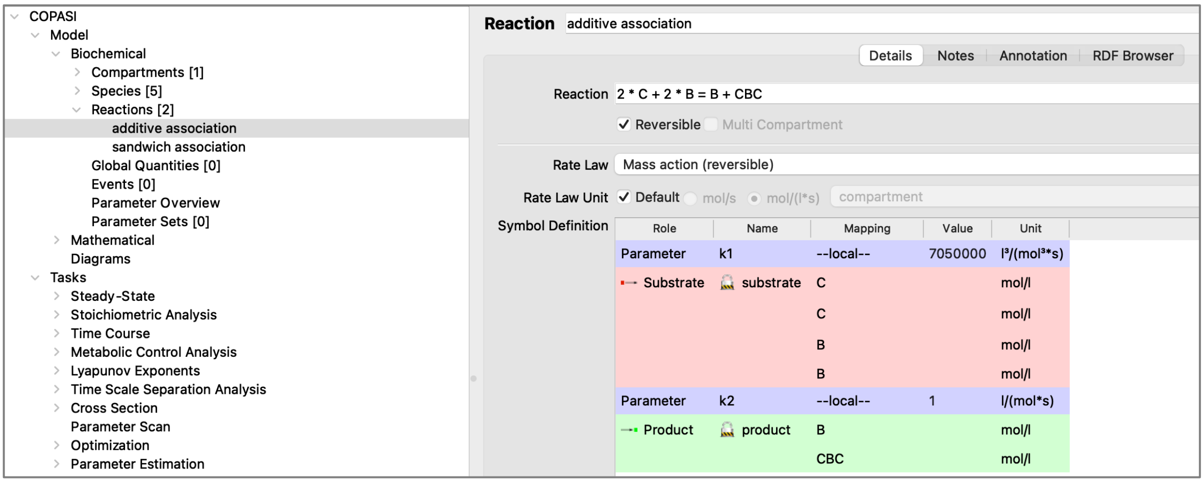

The additive association constant was determined using the same procedure as for the sandwich association constant K_{CAC} of compound **3a** and is defined as either K_{IP}(**6**) = k_1/k_2 = 1.01 x 10^6 M^{-2} or K_{CAC}(**6**) = k_1/k_2 = 7.05 x 10^6 M^{-2}.

Next, in the section “Species” the individual concentration for all compounds were added. As a starting point for this model, we assume that both salts are completely dissociated. Therefore, [**A**] = concentration of salt **3a**, [**C**] = concentration of salt **3a** + concentration of additive **6**, and [**B**] = concentration of additive **6** + concentration of the respective benzhydrylium salt **5**.

The function “Steady State” was used to obtain the concentrations for each species for the given association constants. This process was repeated for each salt concentration of **3a**. Subsequently, the concentration of the single anion **3** can be plotted against k_{obs} to get the second-order rate constant k_2 for the reactive nucleophilic anion **3** in solution. All three resulting sets of reaction rates k_{obs} are depicted in Figure S26.

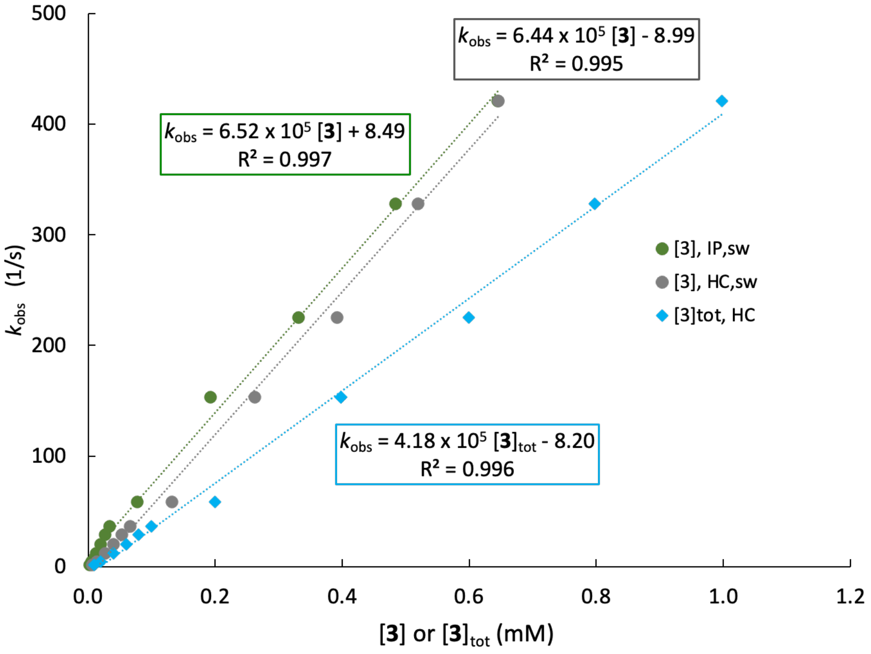

Figure S26. Plot of k_{obs} vs. concentration of catalysts **3a** with additive **6** (turquoise diamonds), concentration of anion [**3**] obtained with model “HC,IP” (green dots) and concentration of anion [**3**] obtained with model “HC,sw” (grey dots) over a concentration range from 0.01–1.0 mM in DCM at 20°C with constant ionic strength I = 1.0 mM.

To distinguish the second-order rate constants k_2 obtained with all model variations, the following superscripts were introduced: “LC” = [**3**]$_{tot}$ with I = variable (blue); “LC,sw” = [**3**] with I = variable (purple);

"HC" = [**3**]$_{tot}$ with *I* = 1.0 mM (turquoise); "HC,IP" = [**3**] with *I* = 1.0 mM (green); "HC,sw" = [**3**] with *I* = 1.0 mM (grey, see Table S26).

Table S26. Second-order rate constants obtained with all five possible analysis methods for salt **3a** with benzhydrylium electrophiles salts $(lil)_2CHBF_4$ (**5a**), $(jul)_2CHBF_4$ (**5b**), and $(ind)_2CHBF_4$ (**5c**) between 0.01 – 0.03 mM in DCM at 20 °C and the corresponding *N*- and s_N-parameters.

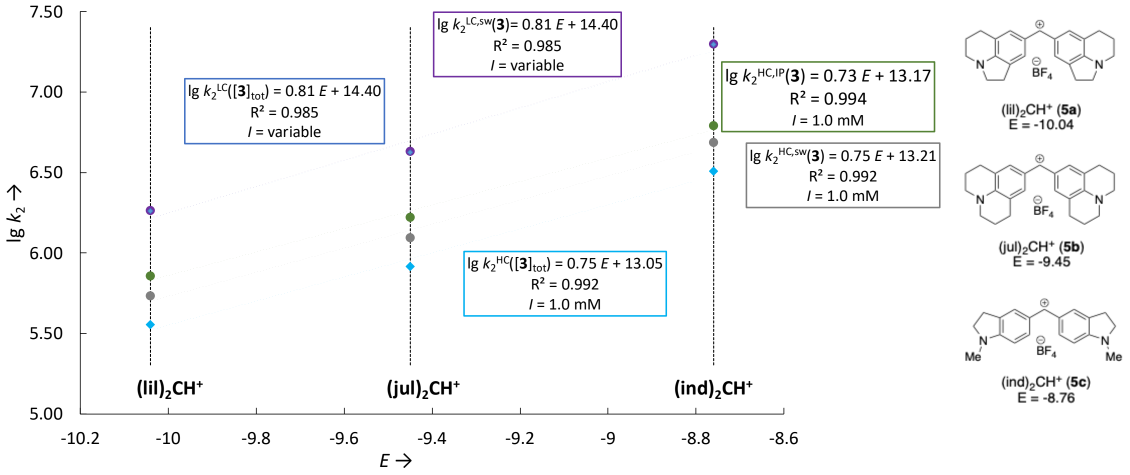

Conditions	Superscript	Nucleophile	k_2 (M^{-1} s^{-1})			N/s_N
			5a	5b	5c	
I = 0.01-0.03 mM	LC	**[3]**$_{tot}$	1.84×10^{6}[a]	4.23×10^6	1.98×10^7	17.78/0.81
Sandwich model	LC,sw	**3**	1.84×10^6	4.28×10^6	2.00×10^7	17.78/0.81
I = 1.0 mM	HC	**[3]**$_{tot}$	3.58×10^5	8.28×10^5	3.23×10^6	17.40/0.75
**6** –> 1:1 association	HC,IP	**3**	7.23×10^5	1.67×10^6	7.17×10^6	18.04/0.73
**6** –> sw associaton	HC,sw	**3**	5.43×10^5	1.25×10^6	4.87×10^6	17.61/0.75

[a] the reaction of **3a** with **5a** was measured twice, resulting in $k_2^{LC} = 1.82 \times 10^6$ M^{-1} s^{-1} (*I* = 0.01-0.03 mM) and $k_2^{LC} = 1.86 \times 10^6$ M^{-1} s^{-1} (*I* = 0.01-0.04 mM, initial three data points of wide range measurement) giving an average $k_2^{LC} = 1.84 \times 10^6$ M^{-1} s^{-1}.

To summarize, at low concentrations between 0.01 – 0.03 mM while the ionic strength *I* in solution is variable, the second-order rates k_2^{LC} and $k_2^{LC,sw}$ are almost identical, which results in identical *N* and s_N parameters. With the addition of additive **6**, the main issue for the determination of the nucleophilicity of anion **3** at higher concentrations being the ongoing association of **3a** was circumvented. Depending on the chosen model for the subsequent analysis, the resulting second-order rate constants k_2 differ from one another. So is $k_2^{HC,IP}$ 1.3 times faster than $k_2^{HC,sw}$, while both are 2.0 and 1.5 times faster than k_2^{HC}, respectively. Overall, the nucleophile-specific sensitivity s_N falls within a narrow window (0.81 vs. 0.73), implying that the relative reactivity of anion **3** is not significantly influenced by the addition of additive **6**.

6.3 Mixed Sandwich Association Extension

Previously, we applied the cationic sandwich model (model 3a) to determine the concentration of free nucleophilic anion **3** in kinetic measurements performed in DCM at higher concentrations, expecting a linear correlation between k_{obs} and [**3**] (see Figure S23). Instead, a steeper but still non-linear correlation was observed. By applying the mixed sandwich association model (model 4) to the same kinetic data, the resulting correlation between k_{obs} and [**3**] is still non-linear, but closer to the ideal theoretical correlation than the correlation based on model 3a (see Figure S27).

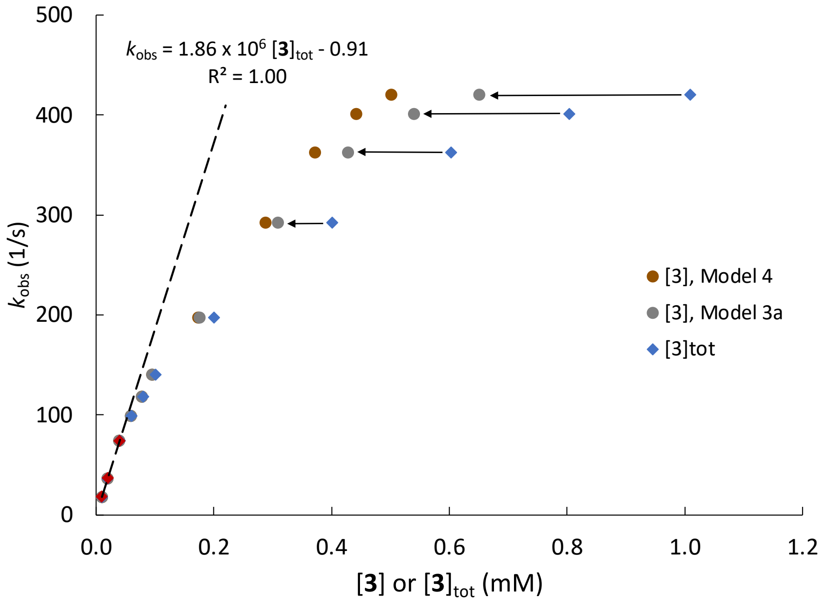

Figure S27. Plot of k_{obs} vs. concentration of catalysts **3a** over a concentration range from 0.01–1.0 mM in DCM at 20°C (blue diamonds), vs. concentration of anion **3** over a concentration range from 0.01–1.0 mM in DCM at 20°C based on the sandwich association model 3a (grey dots), and vs. concentration of anion **3** over a concentration range from 0.01–1.0 mM in DCM at 20°C based on the mixed sandwich association model 4 (brown dots).

The mixed model comes closer to the theoretical ideal case of the predicted linear correlation between k_{obs} and [**3**], indicating a step forward in the development of a comprehensive association model for pyridinamide ion pairs as **3a**.

Next, the mixed sandwich association model (model 4) was used to analyze the ionic strength-controlled kinetic data. Therefore, model 4 was extended by adding eq. S19b to account for the additive association whilst taking both cationic and anionic sandwich association into account in analyzing the nucleophilicity data (see Scheme S8). Moving forward, it was assumed that additive **6** associates according to the cationic sandwich association model.

$$2 * A + 2 * C \overset{\alpha \times K_{CAC}}{\rightleftharpoons} A + CAC$$

$$2 * A + 2 * C \overset{\beta \times K_{ACA}}{\rightleftharpoons} C + ACA$$

$$2 * C + 2 * B \overset{K_{CAC}(add)}{\rightleftharpoons} B + CBC$$

Scheme S8. Mixed model with additive association extension for the analysis of nucleophilicity data.

For numerical simulations, the same procedure was followed as described earlier for the cationic sandwich association extension. For ion pair **3a**, the following association constants K_{CAC}(**3a**) = k_1/k_2 = 2.81 x 10^6 M^{-2}, and K_{ACA}(**3a**) = k_1/k_2 = 1.34 x 10^6 M^{-2}, were applied with K_{CAC}(**6**) = 7.05 x 10^6 M^{-2} still being the same value as before for measurements done with constant ionic strength I = 1.0 mM.

Numerical simulation according to the mixed sandwich association extension gives the concentration of the free anion **3** as well as the concentration of the sandwich anion **3a3**, which could potentially act as another nucleophile. To discuss the different options, we look at the correlation between k_{obs} and the respective nucleophile concentration (see Figure S28).

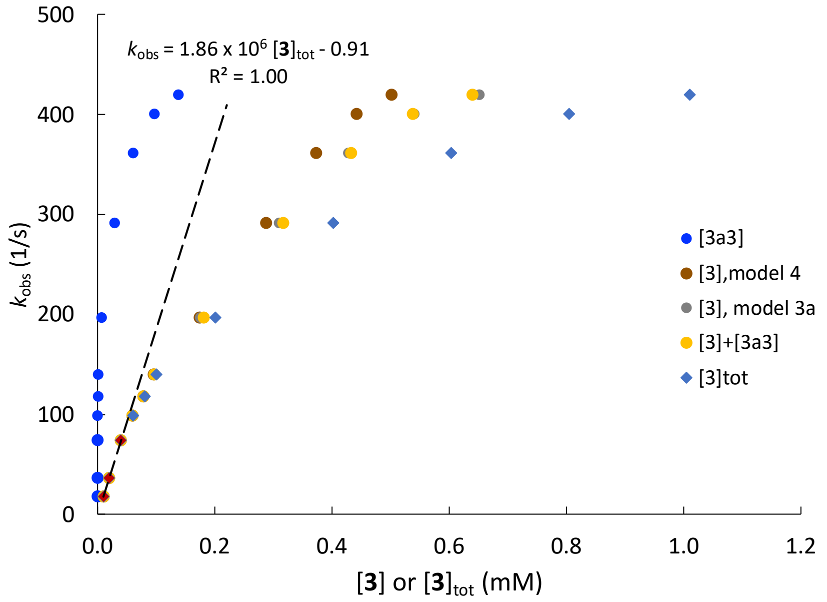

Figure S28. Plot of k_{obs} vs. concentration of catalysts **3a** over a concentration range from 0.01–1.0 mM in DCM at 20°C (blue diamonds), k_{obs} vs. anion [**3**] over a concentration range from 0.01–1.0 mM in DCM at 20°C based on the sandwich association model 3a (grey dots), k_{obs} vs. anion [**3**] + anion sandwich [**3a3**] over a concentration range from 0.01–1.0 mM in DCM at 20°C based on the mixed sandwich association model 4 (yellow dots), k_{obs} vs. anion [**3**] over a concentration range from 0.01–1.0 mM in DCM at 20°C based on the mixed sandwich association model 4 (brown dots), and k_{obs} vs. anion sandwich [**3a3**] over a concentration range from 0.01–1.0 mM in DCM at 20°C based on the mixed sandwich association model 4 (blue dots).

The anionic sandwich complex **3a3** is unlikely to be the more reactive nucleophile due to its reduced reactivity caused by complexation. Simultaneously, its concentration is far too low to plausible be the main reacting nucleophile. In the best-case scenario both the free anion **3** and the anion sandwich **3a3** react equally as nucleophiles (yellow correlation). This would give a correlation that is virtually overlapping with the correlation obtained with numerical simulation according to the cationic sandwich association model 3a (grey dots). As already established, the correlation for k_{obs} vs. [**3**] obtained by applying the mixed sandwich association model 4 (brown dots) is closest to the predicted ideal linear correlation. Moving forward, we will focus on two possible nucleophiles in solution being either the free anion [**3**] or the combination of [**3**] + [**3a3**].

Looking at the second-order rate constant obtained by plotting k_{obs} against [**3**] based on model 3a or model 4 and k_{obs} against [**3**]+[**3a3**], it can be determined that the deviations are clearly visible, but all are in a reasonable window (see Figure S29).

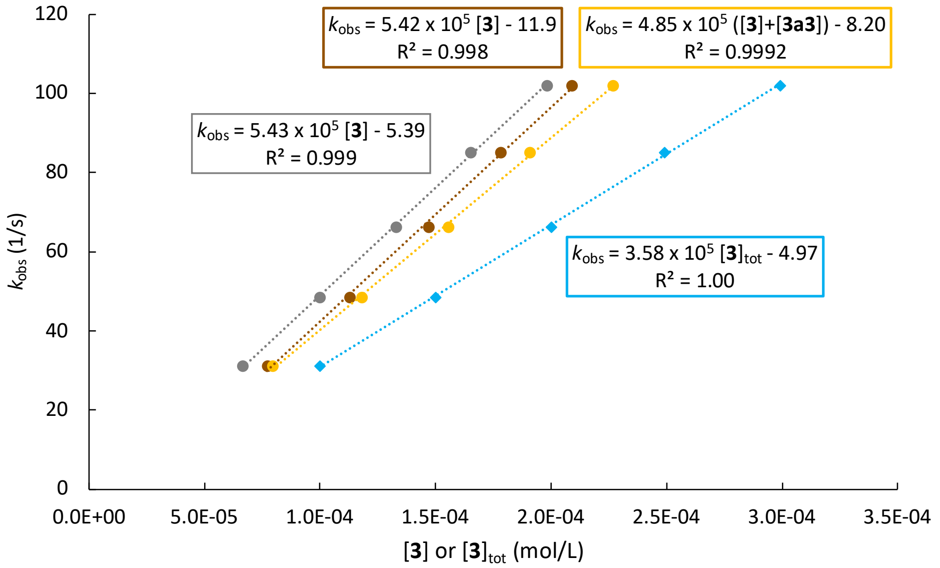

Figure S29. Plot of k_{obs} vs. concentration of catalysts **3a** with additive **6** (turquoise diamonds), concentration of anion [3]+[3a3] obtained with model 4 ("HC,mix", yellow dots), concentration of anion [**3**] obtained with model 4 ("HC,mix", brown dots), and concentration of anion [**3**] obtained with model ("HC,sw", grey dots) over a concentration range from 0.01–1.0 mM in DCM at 20°C with constant ionic strength I = 1.0 mM.

The new superscript "HC,mix" = $[\mathbf{3}]_{tot}$ with I = 1.0 mM (brown) was introduced to distinguish the second-order rate constants k_2 and the resulting N- and s_N-parameters based on the derived mixed sandwich association extension. While the differences in the second-order rate constant are still noticeable, the resulting N- and s_N-parameter are almost overlapping. Therefore, while the analysis of the kinetic data with due regard to the effects of ion association is relevant to obtain a reliable second-order rate constant, for practicability, it is within a justifiable window of deviation to use the overall ion pair concentration $[\mathbf{3}]_{tot}$ and follow the established Mayr method for the data evaluation of the ionic strength-controlled measurements. In Table S27, all second-order rate constants k_2 and the resulting N- and s_N-parameter are summarized for all seven executed ways of evaluation for pyridinamide ion pair **3a**.

Table S27. Second-order rate constants obtained with all seven possible analysis methods for salt **3a** with benzhydrylium electrophiles salts (lil)$_2$CHBF$_4$ (**5a**), (jul)$_2$CHBF$_4$ (**5b**), and (ind)$_2$CHBF$_4$ (**5c**) between 0.01 – 0.03 mM in DCM at 20 °C and the corresponding N- and s_N-parameters.

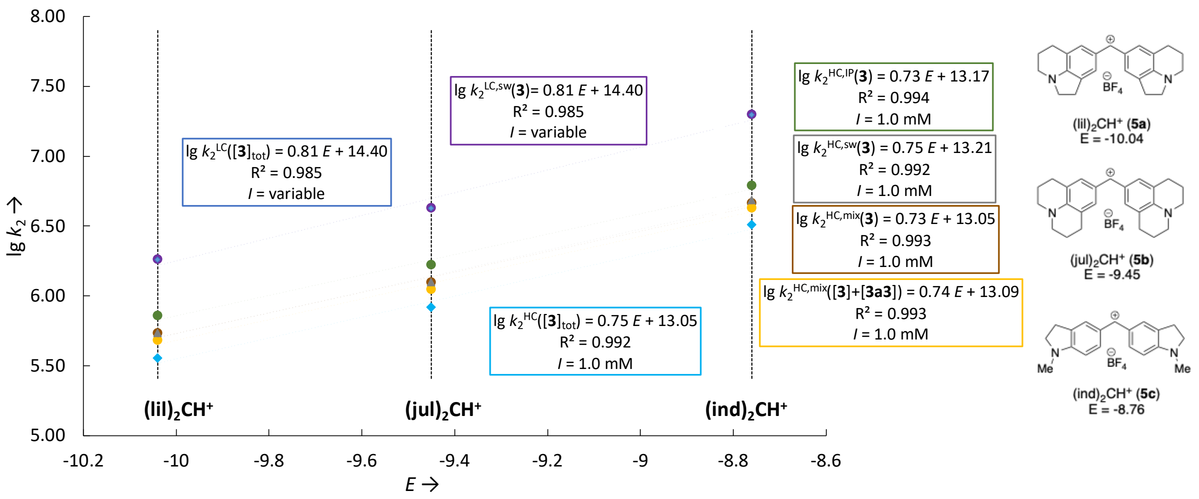

Conditions	Superscript	Nucleophile	k_2 (M^{-1} s^{-1})			N/s_N
			5a	5b	5c	
I = 0.01-0.03 mM	LC	$[\mathbf{3}]_{tot}$	1.84 x 10^6[a]	4.23 x 10^6	1.98 x 10^7	17.78/0.81
Sandwich model	LC,sw	**3**	1.84 x 10^6	4.28 x 10^6	2.00 x 10^7	17.78/0.81

I = 1.0 mM	HC	**[3]$_{tot}$**	3.58 x 10^5	8.28 x 10^5	3.23 x 10^6	17.40/0.75
**6** –> 1:1 association	HC,IP	**3**	7.23 x 10^5	1.67 x 10^6	7.17 x 10^6	18.04/0.73
**6** –> sw associaton	HC,sw	**3**	5.43 x 10^5	1.25 x 10^6	4.87 x 10^6	17.61/0.75
**6** –> sw associaton	HC,mix	**[3]+[3a3]**	4.85 x 10^5	1.12 x 10^6	4.26 x 10^6	17.69/0.74
**6** –> sw associaton	HC,mix	**3**	5.42 x 10^5	1.25 x 10^6	4.64 x 10^6	17.88/0.73

[a] the reaction of **3a** with **5a** was measured twice, resulting in k_2^{LC} = 1.82 x 10^6 M^{-1} s^{-1} (I = 0.01-0.03 mM) and k_2^{LC} = 1.86 x 10^6 M^{-1} s^{-1} (I = 0.01-0.04 mM, initial three data points of wide range measurement) giving an average k_2^{LC} = 1.84 x 10^6 M^{-1} s^{-1}.

In future applications of the mixed sandwich association model, the free anion **3** is treated as the only active nucleophile in solution. The sandwich anion will not be considered in the analysis.

6.4 Nucleophilicity Measurement Data

6.4.1 Nucleophilicity of TCAP in DCM at 20 °C

Reaction of **TCAP** with $(lil)_2CH^+BF_4^-$ (stopped-flow, λ = 640 nm)

$[(lil)_2CH^+BF_4^-]$ (mol L^{-1})	[TCAP] (mol L^{-1})	k_{obs} (s^{-1})
1.01×10^{-5}	1.02×10^{-4}	1.19
	1.53×10^{-4}	1.92
	2.04×10^{-4}	2.62
	2.55×10^{-4}	3.32
	3.06×10^{-4}	4.10

$k_2 = 1.42 \times 10^4$ L mol^{-1} s^{-1}

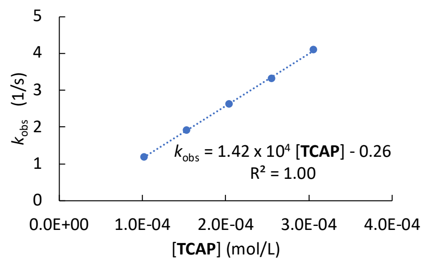

Reaction of **TCAP** with $(jul)_2CH^+BF_4^-$ (stopped-flow, λ = 643 nm)

$[(jul)_2CH^+BF_4^-]$ (mol L^{-1})	[TCAP] (mol L^{-1})	k_{obs} (s^{-1})
9.85×10^{-6}	1.02×10^{-4}	2.71
	1.53×10^{-4}	4.41
	2.04×10^{-4}	6.04
	2.55×10^{-4}	7.39
	3.06×10^{-4}	9.15

$k_2 = 3.11 \times 10^4$ L mol^{-1} s^{-1}

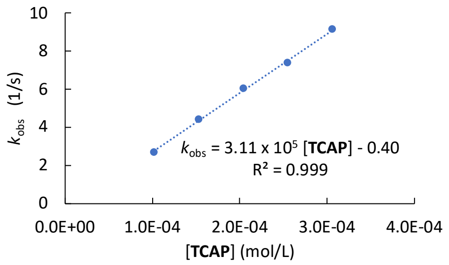

Reaction of **TCAP** with $(ind)_2CH^+BF_4^-$ (stopped-flow, λ = 626 nm)

$[(ind)_2CH^+BF_4^-]$ (mol L^{-1})	[TCAP] (M^{-1})	k_{obs} (s^{-1})
9.90×10^{-6}	1.02×10^{-4}	11.4
	1.53×10^{-4}	18.9
	2.04×10^{-4}	25.2
	2.55×10^{-4}	31.8
	3.06×10^{-4}	37.1

$k_2 = 1.28 \times 10^5$ L mol^{-1} s^{-1}

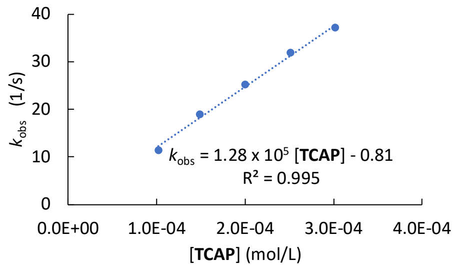

Reaction of **TCAP** with $(pyr)_2CH^+BF_4^-$ (stopped-flow, λ = 620 nm)

$[(pyr)_2CH^+BF_4^-]$ (mol L^{-1})	[TCAP] (mol L^{-1})	k_{obs} (s^{-1})
1.01×10^{-5}	1.02×10^{-4}	61.9
	1.53×10^{-4}	102
	2.04×10^{-4}	137
	2.55×10^{-4}	178
	3.06×10^{-4}	210

$k_2 = 7.41 \times 10^5$ L mol^{-1} s^{-1}

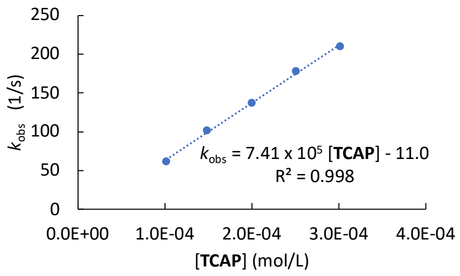

Reaction of **TCAP** with $(dma)_2CH^+BF_4^-$ (stopped-flow, λ = 613 nm)

$[(dma)_2CH^+BF_4^-]$ (mol L^{-1})	[TCAP] (mol L^{-1})	k_{obs} (s^{-1})
	1.02×10^{-4}	197
	1.53×10^{-4}	323
9.90×10^{-6}	2.04×10^{-4}	461
	2.55×10^{-4}	576
	3.06×10^{-4}	719

k_2 = 2.58 x 10^6 L mol^{-1} s^{-1}

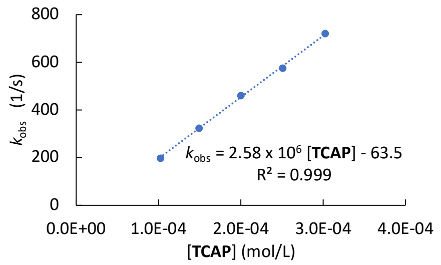

Determination of *N* and s_N parameter for **TCAP** in DCM

Electrophile	*E*	k_2 (M^{-1} s^{-1})
Lil	-10.04	1.42×10^4
Jul	-9.45	3.11×10^4
Ind	-8.76	1.28×10^5
Pyr	-7.69	7.41×10^5
Dma	-7.02	2.58×10^6

***N* = 15.41** **s_N = 0.76**

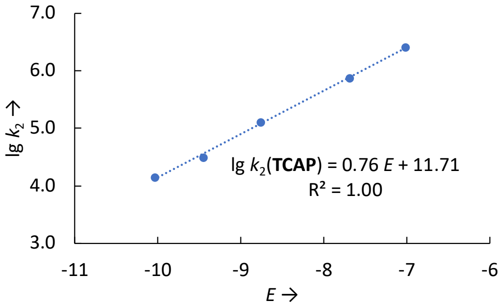

6.4.2 Nucleophilicity of 3a in MeCN at 20 °C

Reaction of **3a** with $(lil)_2CH^+BF_4^-$ (stopped-flow, λ = 632 nm)

[5a] (mol L^{-1})	[3a] (mol L^{-1})	k_{obs} (s^{-1})
	1.00×10^{-5}	0.34
	1.50×10^{-5}	0.38
1.11×10^{-6}	2.00×10^{-5}	0.41
	2.51×10^{-5}	0.45
	3.01×10^{-5}	–

$k_2 = 7.16 \times 10^3$ L mol^{-1} s^{-1}

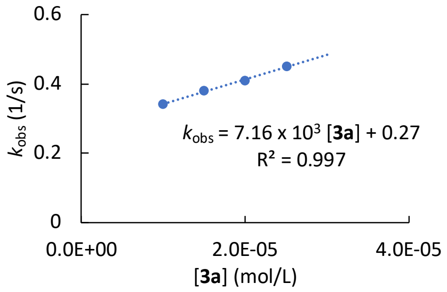

Reaction of **3a** with $(jul)_2CH^+BF_4^-$ (stopped-flow, λ = 635 nm)

[5b] (mol L^{-1})	[3a] (mol L^{-1})	k_{obs} (s^{-1})
	1.00×10^{-5}	–
	1.50×10^{-5}	0.86
1.00×10^{-6}	2.00×10^{-5}	0.92
	2.51×10^{-5}	1.00
	3.01×10^{-5}	1.09

$k_2 = 1.53 \times 10^4$ L mol^{-1} s^{-1}

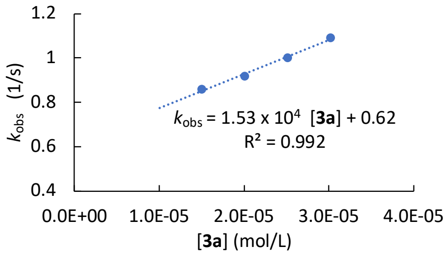

Reaction of **3a** with $(ind)_2CH^+BF_4^-$ (stopped-flow, λ = 616 nm)

[5c] (mol L^{-1})	[3a] (mol L^{-1})	k_{obs} (s^{-1})
	1.00×10^{-5}	–
	1.50×10^{-5}	0.86
9.27×10^{-7}	2.00×10^{-5}	1.08
	2.51×10^{-5}	1.30
	3.01×10^{-5}	1.48

$k_2 = 4.13 \times 10^4$ L mol^{-1} s^{-1}

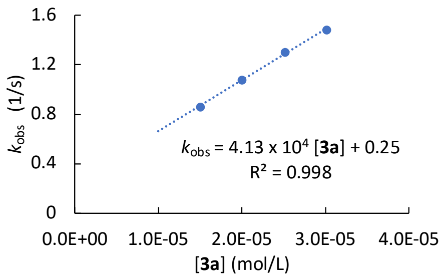

Determination of N and s_N parameter for **3a** in MeCN

Electrophile	E	k_2 (M^{-1} s^{-1})
**5a**	-10.04	7.16×10^3
**5b**	-9.45	1.53×10^4
**5c**	-8.76	4.13×10^4

***N* = 16.38** ***s*$_N$ = 0.60**

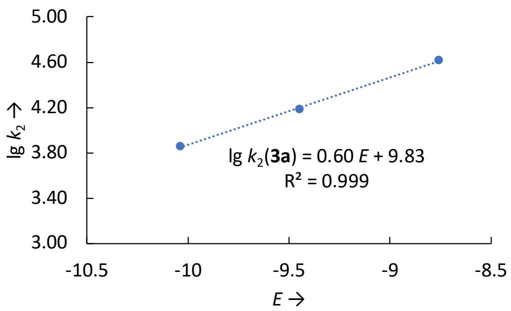

6.4.3 Nucleophilicity of **4a** in MeCN at 20 °C

Reaction of **4a** with $(lil)_2CH^+BF_4^-$ (stopped-flow, λ = 632 nm)

[**5a**] (mol L^{-1})	[**4a**] (mol L^{-1})	k_{obs} (s^{-1})
	9.89 x 10^{-6}	0.33
	1.53 x 10^{-5}	0.73
9.69 x 10^{-7}	1.98 x 10^{-5}	0.97
	2.52 x 10^{-5}	1.12
	2.97 x 10^{-5}	1.40

k_2 = 5.11 x 10^4 L mol^{-1} s^{-1}

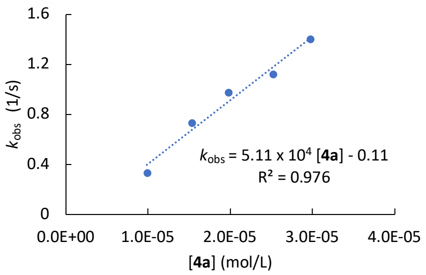

Reaction of **4a** with $(jul)_2CH^+BF_4^-$ (stopped-flow, λ = 635 nm)

[**5b**] (mol L^{-1})	[**4a**] (mol L^{-1})	k_{obs} (s^{-1})
	9.89 x 10^{-6}	0.58
	1.53 x 10^{-5}	1.49
9.89 x 10^{-7}	1.98 x 10^{-5}	2.09
	2.52 x 10^{-5}	–
	2.97 x 10^{-5}	3.32

k_2 = 1.36 x 10^5 L mol^{-1} s^{-1}

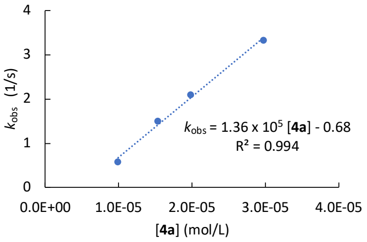

Reaction of **4a** with $(ind)_2CH^+BF_4^-$ (stopped-flow, λ = 616 nm)

[**5c**] (mol L^{-1})	[**4a**] (mol L^{-1})	k_{obs} (s^{-1})
	9.89 x 10^{-6}	1.81
	1.53 x 10^{-5}	3.59
1.06 x 10^{-6}	1.98 x 10^{-5}	5.16
	2.52 x 10^{-5}	–
	2.97 x 10^{-5}	8.66

k_2 = 3.47 x 10^5 L mol^{-1} s^{-1}

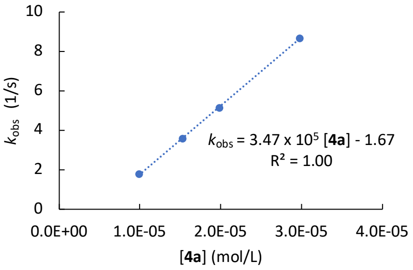

Determination of N and s_N parameter for **4a** in MeCN

Electrophile	E	k_2 (M^{-1} s^{-1})
**5a**	-10.04	5.11 x 10^4
**5b**	-9.45	1.36 x 10^5
**5c**	-8.76	3.47 x 10^5

***N* = 17.28** **s_N = 0.65**

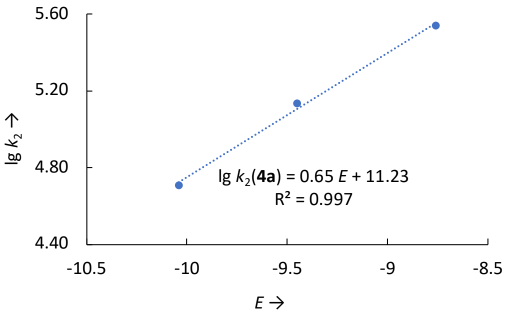

6.4.4 Nucleophilicity of 3a in DCM at 20 °C

Equilibrium constants for numerical simulation: K_{CAC} = 6.38 x 10^6 M^{-2} for **3a**.

Reaction of salt **3a** with $(lil)_2CH^+BF_4^-$ (stopped-flow, λ = 640 nm)

[5a] (mol L^{-1})	$[3]_{tot}$ (mol L^{-1})	[3] (mol L^{-1})	k_{obs} (s^{-1})
	9.98 x 10^{-6}	9.97 x 10^{-6}	22.2
	1.50 x 10^{-5}	1.50 x 10^{-5}	34.1
8.63 x 10^{-7}	2.00 x 10^{-5}	2.00 x 10^{-5}	43.7
	2.50 x 10^{-5}	2.49 x 10^{-5}	52.2
	2.99 x 10^{-5}	2.97 x 10^{-5}	58.6

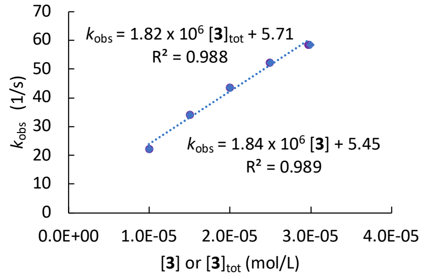

k_2^{LC} = 1.82 x 10^6 L mol^{-1} s^{-1} for $[3]_{tot}$ **$k_2^{LC,sw}$ = 1.84 x 10^6 L mol^{-1} s^{-1}** for [3]

Reaction of **3a** with $(jul)_2CH^+BF_4^-$ (stopped-flow, λ = 643 nm)

[5b] (mol L^{-1})	$[3]_{tot}$ (mol L^{-1})	[3] (mol L^{-1})	k_{obs} (s^{-1})
	9.95 x 10^{-6}	9.94 x 10^{-6}	33.2
	1.49 x 10^{-5}	1.49 x 10^{-5}	63.3
8.82 x 10^{-7}	1.99 x 10^{-5}	1.99 x 10^{-5}	84.2
	2.49 x 10^{-5}	2.48 x 10^{-5}	102
	2.98 x 10^{-5}	2.96 x 10^{-5}	119

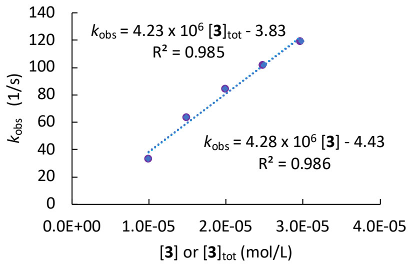

k_2^{LC} = 4.23 x 10^6 L mol^{-1} s^{-1} for $[3]_{tot}$ **$k_2^{LC,sw}$ = 4.28 x 10^6 L mol^{-1} s^{-1}** for [3]

Reaction of **3a** with $(ind)_2CH^+BF_4^-$ (stopped-flow, λ = 626 nm)

[5c] (mol L^{-1})	$[3]_{tot}$ (mol L^{-1})	[3] (mol L^{-1})	k_{obs} (s^{-1})
	9.95 x 10^{-6}	9.94 x 10^{-6}	110
	1.49 x 10^{-5}	1.49 x 10^{-5}	222
9.87 x 10^{-7}	1.99 x 10^{-5}	1.99 x 10^{-5}	338
	2.49 x 10^{-5}	2.48 x 10^{-5}	426
	2.98 x 10^{-5}	2.96 x 10^{-5}	501

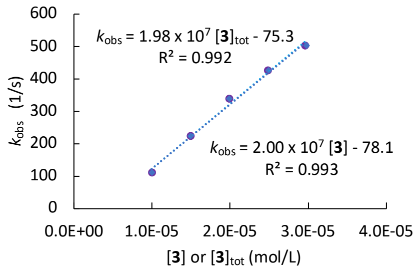

k_2^{LC} = 1.98 x 10^7 L mol^{-1} s^{-1} for $[3]_{tot}$ **$k_2^{LC,sw}$ = 2.00 x 10^7 L mol^{-1} s^{-1}** for [3]

Determination of *N* and s_N parameter for **3a** in DCM

Electrophile	*E*	k_2^{LC} (M^{-1} s^{-1}) for $[3]_{tot}$	$k_2^{LC,sw}$ (M^{-1} s^{-1}) for [3]
**5a**	-10.04	1.82 x 10^6	1.84 x 10^6
**5b**	-9.45	4.23 x 10^6	4.28 x 10^6
**5c**	-8.76	1.98 x 10^7	2.00 x 10^7

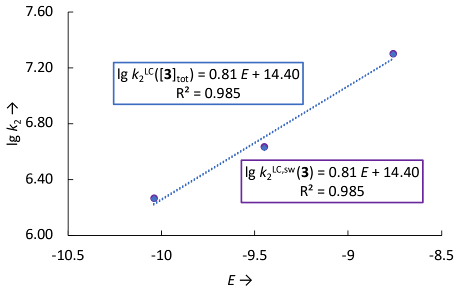

***N* = 17.78** **s_N = 0.81** for $[3]_{tot}$

***N* = 17.78** **s_N = 0.81** for [3]

6.4.5 Nucleophilicity of 3a in DCM at I = 1.0 mM at 20 °C (with 1:1 Model for Additive 6)

Equilibrium constants for numerical simulation: K_{CAC} = 6.38 x 10^6 M^{-2} for **3a**, K_{IP} = 1.01 x 10^7 M^{-2} for **6**.

Reaction of **3a + 6** with (lil)$_2$CH$^+$BF$_4^-$ (stopped-flow, λ = 640 nm)

**[5a] (mol L^{-1})**	**[3]$_{tot}$ (mol L^{-1})**	**[3] (mol L^{-1})**	**[6] (mol L^{-1})**	k_{obs} **(s^{-1})**
	9.98 x 10^{-5}	3.47 x 10^{-5}	8.91 x 10^{-4}	31.1
	1.50 x 10^{-4}	5.63 x 10^{-5}	8.34 x 10^{-4}	48.5
9.27 x 10^{-6}	2.00 x 10^{-4}	8.68 x 10^{-5}	7.87 x 10^{-4}	66.2
	2.49 x 10^{-4}	1.05 x 10^{-4}	7.41 x 10^{-4}	85.0
	2.99 x 10^{-4}	1.33 x 10^{-4}	6.94 x 10^{-4}	102

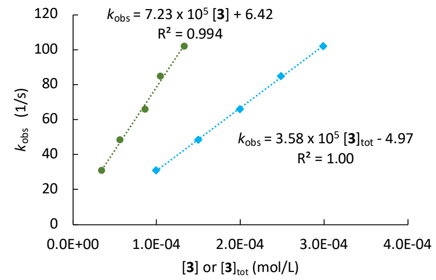

k_2^{HC} **= 3.58 x 10^5 L mol^{-1} s^{-1}** for [3a] $k_2^{HC,IP}$ **= 7.23 x 10^5 L mol^{-1} s^{-1}** for [3]

Reaction of **3a + 6** with (jul)$_2$CH$^+$BF$_4^-$ (stopped-flow, λ = 643 nm)

**[5b] (mol L^{-1})**	**[3]$_{tot}$ (mol L^{-1})**	**[3] (mol L^{-1})**	**[6] (mol L^{-1})**	k_{obs} **(s^{-1})**
	9.98 x 10^{-5}	3.47 x 10^{-5}	8.91 x 10^{-4}	62.1
	1.50 x 10^{-4}	5.63 x 10^{-5}	8.34 x 10^{-4}	101
9.40 x 10^{-6}	2.00 x 10^{-4}	8.68 x 10^{-5}	7.87 x 10^{-4}	141
	2.49 x 10^{-4}	1.05 x 10^{-4}	7.41 x 10^{-4}	183
	2.99 x 10^{-4}	1.33 x 10^{-4}	6.94 x 10^{-4}	227

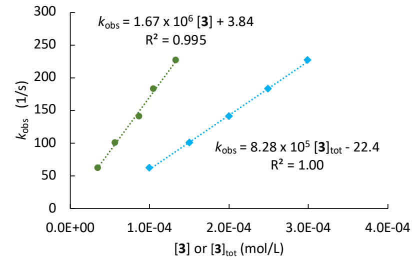

k_2^{HC} **= 8.28 x 10^5 L mol^{-1} s^{-1}** for [3a] $k_2^{HC,IP}$ **= 1.67 x 10^6 L mol^{-1} s^{-1}** for [3]

Reaction of **3a + 6** with (ind)$_2$CH$^+$BF$_4^-$ (stopped-flow, λ = 626 nm)

**[5c] (mol L^{-1})**	**[3]$_{tot}$ (mol L^{-1})**	**[3] (mol L^{-1})**	**[6] (mol L^{-1})**	k_{obs} **(s^{-1})**
	9.98 x 10^{-5}	3.47 x 10^{-5}	8.91 x 10^{-4}	244
	1.50 x 10^{-4}	5.63 x 10^{-5}	8.34 x 10^{-4}	400
9.99 x 10^{-6}	2.00 x 10^{-4}	8.68 x 10^{-5}	7.87 x 10^{-4}	568
	2.49 x 10^{-4}	1.05 x 10^{-4}	7.41 x 10^{-4}	–
	2.99 x 10^{-4}	1.33 x 10^{-4}	6.94 x 10^{-4}	–

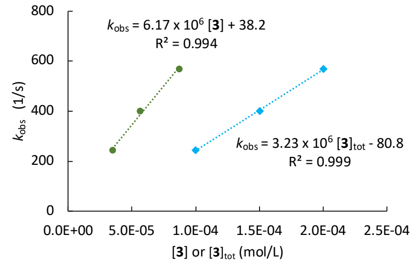

k_2^{HC} **= 3.23 x 10^6 L mol^{-1} s^{-1}** for [3a] $k_2^{HC,IP}$ **= 7.17 x 10^6 L mol^{-1} s^{-1}** for [3]

Determination of N and s_N parameter for **3a** in DCM at constant ionic strength of 1.0 mM

**Electrophile**	E	k_2^{HC} **(M^{-1} s^{-1})** for [3]$_{tot}$	$k_2^{HC,IP}$ **(M^{-1} s^{-1})** for [3]
**5a**	-10.04	3.58 x 10^5	7.23 x 10^5
**5b**	-9.45	8.28 x 10^5	1.67 x 10^6
**5c**	-8.76	3.23 x 10^6	7.17 x 10^6

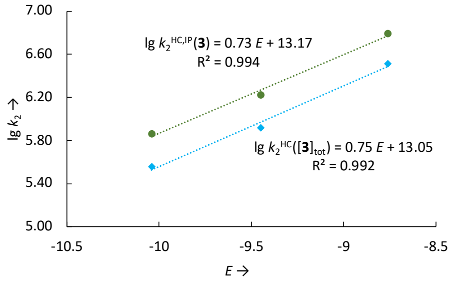

N **= 17.40** s_N **= 0.75** for [3]$_{tot}$

N **= 18.04** s_N **= 0.73** for [3]

6.4.6 Nucleophilicity of 3a in DCM at *I* = 1.0 mM at 20 °C (with Cationic Sandwich Model for Additive 6)

Equilibrium constants for numerical simulation: K_{CAC} = 6.38 x 10^6 M^{-2} for **3a**, K_{CAC} = 7.05 x 10^6 M^{-2} for **6**.

Reaction of **3a + 6** with $(lil)_2CH^+BF_4^-$ (stopped-flow, λ = 640 nm)

[5a] (mol L^{-1})	$[3]_{tot}$ (mol L^{-1})	[3] (mol L^{-1})	[6] (mol L^{-1})	k_{obs} (s^{-1})
9.27 x 10^{-6}	9.98 x 10^{-5}	6.65 x 10^{-5}	8.91 x 10^{-4}	31.1
	1.50 x 10^{-4}	9.98 x 10^{-5}	8.34 x 10^{-4}	48.5
	2.00 x 10^{-4}	1.33 x 10^{-4}	7.87 x 10^{-4}	66.2
	2.49 x 10^{-4}	1.65 x 10^{-4}	7.41 x 10^{-4}	85.0
	2.99 x 10^{-4}	1.98 x 10^{-4}	6.94 x 10^{-4}	102

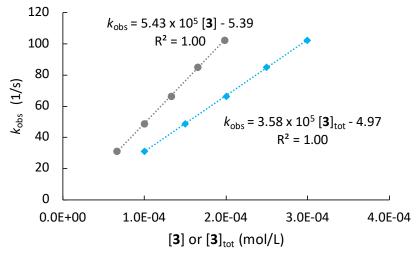

k_2^{HC} **= 3.58 x 10^5 L mol^{-1} s^{-1}** for $[3]_{tot}$ $k_2^{HC,sw}$ **= 5.43 x 10^5 L mol^{-1} s^{-1}** for [3]

Reaction of **3a + 6** with $(jul)_2CH^+BF_4^-$ (stopped-flow, λ = 643 nm)

[5b] (mol L^{-1})	$[3]_{tot}$ (mol L^{-1})	[3] (mol L^{-1})	[6] (mol L^{-1})	k_{obs} (s^{-1})
9.40 x 10^{-6}	9.98 x 10^{-5}	6.65 x 10^{-5}	8.91 x 10^{-4}	62.1
	1.50 x 10^{-4}	9.98 x 10^{-5}	8.34 x 10^{-4}	101
	2.00 x 10^{-4}	1.33 x 10^{-4}	7.87 x 10^{-4}	141
	2.49 x 10^{-4}	1.65 x 10^{-4}	7.41 x 10^{-4}	183
	2.99 x 10^{-4}	1.98 x 10^{-4}	6.94 x 10^{-4}	227

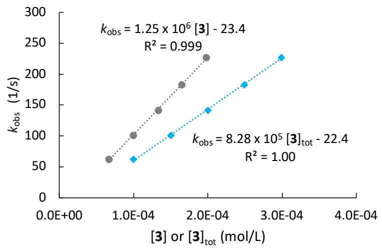

k_2^{HC} **= 8.28 x 10^5 L mol^{-1} s^{-1}** for $[3]_{tot}$ $k_2^{HC,sw}$ **= 1.25 x 10^6 L mol^{-1} s^{-1}** for [3]

Reaction of **3a + 6** with $(ind)_2CH^+BF_4^-$ (stopped-flow, λ = 626 nm)

[5c] (mol L^{-1})	$[3]_{tot}$ (mol L^{-1})	[3] (mol L^{-1})	[6] (mol L^{-1})	k_{obs} (s^{-1})
9.99 x 10^{-6}	9.98 x 10^{-5}	6.65 x 10^{-5}	8.91 x 10^{-4}	244
	1.50 x 10^{-4}	9.98 x 10^{-5}	8.34 x 10^{-4}	400
	2.00 x 10^{-4}	1.33 x 10^{-4}	7.87 x 10^{-4}	568
	2.49 x 10^{-4}	1.65 x 10^{-4}	7.41 x 10^{-4}	–
	2.99 x 10^{-4}	1.98 x 10^{-4}	6.94 x 10^{-4}	–

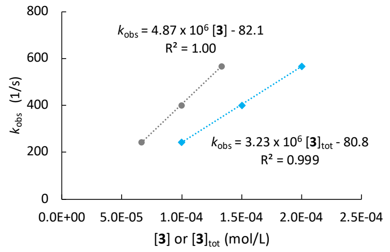

k_2^{HC} **= 3.23 x 10^6 L mol^{-1} s^{-1}** for $[3]_{tot}$ $k_2^{HC,sw}$ **= 4.87 x 10^6 L mol^{-1} s^{-1}** for [3]

Determination of *N* and s_N parameter for **3a** in DCM at constant ionic strength of 1.0 mM

Electrophile	*E*	k_2^{HC} (M^{-1} s^{-1}) for $[3]_{tot}$	$k_2^{HC,sw}$ (M^{-1} s^{-1}) for [3]
**5a**	-10.04	3.58 x 10^5	5.43 x 10^5
**5b**	-9.45	8.28 x 10^5	1.25 x 10^6
**5c**	-8.76	3.23 x 10^6	4.87 x 10^6

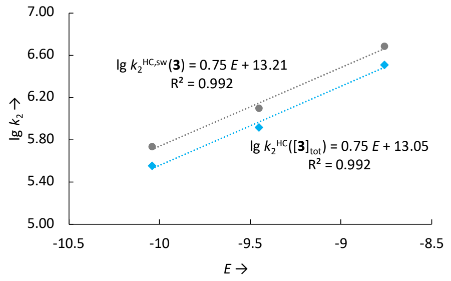

***N* = 17.40** s_N **= 0.75** for $[3]_{tot}$

***N* = 17.61** s_N **= 0.75** for [3]

6.4.7 Nucleophilicity of 3a in DCM at *I* = 1.0 mM at 20 °C (with Mixed Sandwich Model Extension)

Equilibrium constants for numerical simulation: $\alpha \times K_{CAC} = 2.81 \times 10^6$ M^{-2} and $\beta \times K_{ACA} = 1.34 \times 10^6$ M^{-2} for **3a** (α/β = 0.44/0.21), $K_{CAC} = 7.05 \times 10^6$ M^{-2} for **6**.

Reaction of **3a + 6** with $(lil)_2CH^+BF_4^-$ (stopped-flow, λ = 640 nm)

**[5a] (mol L^{-1})**	**$[3]_{tot}$ (mol L^{-1})**	**[3] (mol L^{-1})**	**[6] (mol L^{-1})**	**k_{obs} (s^{-1})**
	9.98×10^{-5}	7.72×10^{-5}	8.91×10^{-4}	31.1
	1.50×10^{-4}	1.13×10^{-4}	8.34×10^{-4}	48.5
9.27×10^{-6}	2.00×10^{-4}	1.47×10^{-4}	7.87×10^{-4}	66.2
	2.49×10^{-4}	1.78×10^{-4}	7.41×10^{-4}	85.0
	2.99×10^{-4}	2.09×10^{-4}	6.94×10^{-4}	102

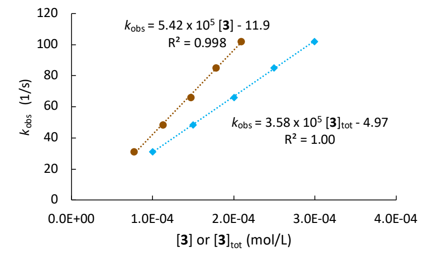

k_2^{HC} = 3.58 x 10^5 L mol^{-1} s^{-1} for $[3]_{tot}$ **$k_2^{HC,mix}$ = 5.42 x 10^5 L mol^{-1} s^{-1}** for [3]

Reaction of **3a + 6** with $(jul)_2CH^+BF_4^-$ (stopped-flow, λ = 643 nm)

**[5b] (mol L^{-1})**	**$[3]_{tot}$ (mol L^{-1})**	**[3] (mol L^{-1})**	**[6] (mol L^{-1})**	**k_{obs} (s^{-1})**
	9.98×10^{-5}	7.72×10^{-5}	8.91×10^{-4}	62.1
	1.50×10^{-4}	1.13×10^{-4}	8.34×10^{-4}	101
9.40×10^{-6}	2.00×10^{-4}	1.47×10^{-4}	7.87×10^{-4}	141
	2.49×10^{-4}	1.78×10^{-4}	7.41×10^{-4}	183
	2.99×10^{-4}	2.09×10^{-4}	6.94×10^{-4}	227

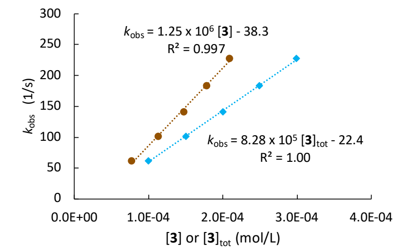

k_2^{HC} = 8.28 x 10^5 L mol^{-1} s^{-1} for $[3]_{tot}$ **$k_2^{HC,mix}$ = 1.25 x 10^6 L mol^{-1} s^{-1}** for [3]

Reaction of **3a + 6** with $(ind)_2CH^+BF_4^-$ (stopped-flow, λ = 626 nm)

**[5c] (mol L^{-1})**	**$[3]_{tot}$ (mol L^{-1})**	**[3] (mol L^{-1})**	**[6] (mol L^{-1})**	**k_{obs} (s^{-1})**
	9.98×10^{-5}	7.72×10^{-5}	8.91×10^{-4}	244
	1.50×10^{-4}	1.13×10^{-4}	8.34×10^{-4}	400
9.99×10^{-6}	2.00×10^{-4}	1.47×10^{-4}	7.87×10^{-4}	568
	2.49×10^{-4}	1.78×10^{-4}	7.41×10^{-4}	–
	2.99×10^{-4}	2.09×10^{-4}	6.94×10^{-4}	–

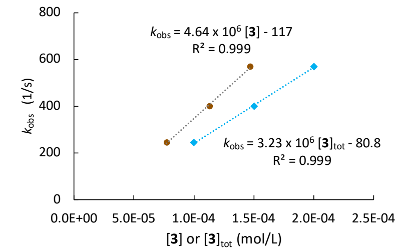

k_2^{HC} = 3.23 x 10^6 L mol^{-1} s^{-1} for $[3]_{tot}$ **$k_2^{HC,mix}$ = 4.64 x 10^6 L mol^{-1} s^{-1}** for [3]

Determination of *N* and s_N parameter for **3a** in DCM at constant ionic strength of 1.0 mM

**Electrophile**	***E***	**k_2^{HC} (M^{-1} s^{-1}) for $[3]_{tot}$**	**$k_2^{HC,mix}$ (M^{-1} s^{-1}) for [3]**
**5a**	-10.04	3.58×10^5	5.42×10^5
**5b**	-9.45	8.28×10^5	1.25×10^6
**5c**	-8.76	3.23×10^6	4.64×10^6

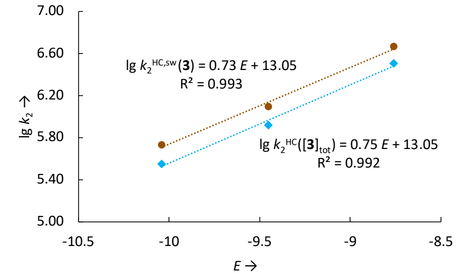

***N* = 17.40** **s_N = 0.75** for $[3]_{tot}$

***N* = 17.88** **s_N = 0.73** for [3]

6.4.8 Nucleophilicity of 4a in DCM at I = 1.0 mM at 20 °C (with Cationic Sandwich Model for Additive 6)

Equilibrium constants for numerical simulation: K_{CAC} = 6.50 x 10^6 M^{-2} for **4a**, K_{CAC} = 7.05 x 10^6 M^{-2} for **6**.

Reaction of **4a + 6** with $(lil)_2CH^+BF_4^-$ (stopped-flow, λ = 640 nm)

[5a] (mol L^{-1}))	$[4]_{tot}$ (mol L^{-1})	[4] (mol L^{-1})	[6] (mol L^{-1})	k_{obs} (s^{-1})
	3.96 x 10^{-5}	2.62 x 10^{-5}	9.54 x 10^{-4}	29.0
	5.93 x 10^{-5}	3.91 x 10^{-5}	9.41 x 10^{-4}	51.6
4.21 x 10^{-6}	7.91 x 10^{-5}	5.22 x 10^{-5}	9.14 x 10^{-4}	76.6
	9.89 x 10^{-5}	6.52 x 10^{-5}	9.00 x 10^{-4}	99.3
	1.19 x 10^{-4}	7.82 x 10^{-5}	8.87 x 10^{-4}	127

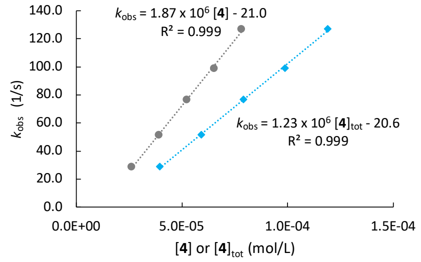

k_2^{HC} **= 1.23 x 10^6 L mol^{-1} s^{-1}** for $[4]_{tot}$ $k_2^{HC,sw}$ **= 1.87 x 10^6 L mol^{-1} s^{-1}** for [4]

Reaction of **4a + 6** with $(jul)_2CH^+BF_4^-$ (stopped-flow, λ = 643 nm)

[5b] (mol L^{-1})	$[4]_{tot}$ (mol L^{-1})	[4] (mol L^{-1})	[6] (mol L^{-1})	k_{obs} (s^{-1})
	3.96 x 10^{-5}	2.62 x 10^{-5}	9.54 x 10^{-4}	59.0
	5.93 x 10^{-5}	3.91 x 10^{-5}	9.41 x 10^{-4}	119
4.06 x 10^{-6}	7.91 x 10^{-5}	5.22 x 10^{-5}	9.14 x 10^{-4}	188
	9.89 x 10^{-5}	6.52 x 10^{-5}	9.00 x 10^{-4}	235
	1.19 x 10^{-4}	7.82 x 10^{-5}	8.87 x 10^{-4}	303

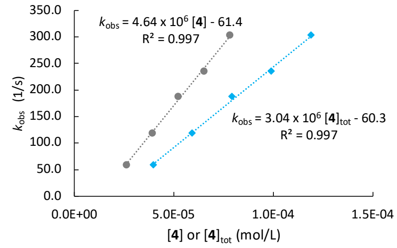

k_2^{HC} **= 3.04 x 10^6 L mol^{-1} s^{-1}** for $[4]_{tot}$ $k_2^{HC,sw}$ **= 4.64 x 10^6 L mol^{-1} s^{-1}** for [4]

Reaction of **4a + 6** with $(ind)_2CH^+BF_4^-$ (stopped-flow, λ = 626 nm)

[5c] (mol L^{-1})	$[4]_{tot}$ (mol L^{-1})	[4] (mol L^{-1})	[6] (mol L^{-1})	k_{obs} (s^{-1})
	3.96 x 10^{-5}	2.62 x 10^{-5}	9.54 x 10^{-4}	188
	5.93 x 10^{-5}	3.91 x 10^{-5}	9.41 x 10^{-4}	378
4.14 x 10^{-6}	7.91 x 10^{-5}	5.22 x 10^{-5}	9.14 x 10^{-4}	588
	9.89 x 10^{-5}	6.52 x 10^{-5}	9.00 x 10^{-4}	–
	1.19 x 10^{-4}	7.82 x 10^{-5}	8.87 x 10^{-4}	846

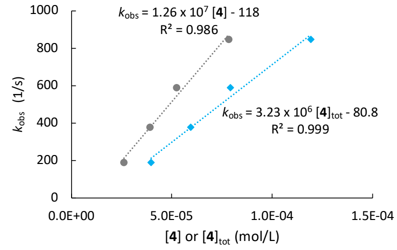

k_2^{HC} **= 8.27 x 10^6 L mol^{-1} s^{-1}** for $[4]_{tot}$ $k_2^{HC,sw}$ **= 1.26 x 10^7 L mol^{-1} s^{-1}** for [4]

Determination of N and s_N parameter for **4a** in DCM at constant ionic strength of 1.0 mM

Electrophile	E	k_2^{HC}(M^{-1} s^{-1}) for $[4]_{tot}$	$k_2^{HC,sw}$ (M^{-1} s^{-1}) for [4]
**5a**	-10.04	1.23 x 10^6	1.87 x 10^6
**5b**	-9.45	3.04 x 10^6	4.64 x 10^6
**5c**	-8.76	8.27 x 10^6	1.26 x 10^7

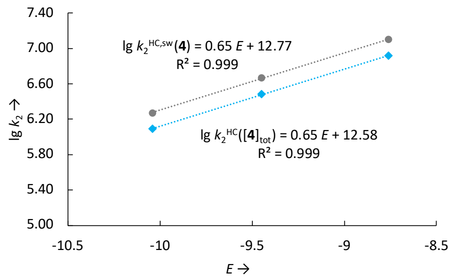

***N* = 19.35** s_N **= 0.65** for $[4]_{tot}$

***N* = 19.66** s_N **= 0.65** for [4]

6.4.9 Nucleophilicity of 4a in DCM at *I* = 1.0 mM at 20 °C (with Mixed Sandwich Model Extension)

Equilibrium constants for numerical simulation: $\alpha \times K_{CAC} = 7.80 \times 10^5$ M^{-2} and $\beta \times K_{ACA} = 3.97 \times 10^6$ M^{-2} for **4a** ($\alpha/\beta = 0.12/0.61$), $K_{CAC} = 7.05 \times 10^6$ M^{-2} for **6**.

Reaction of **4a** + **6** with $(lil)_2CH^+BF_4^-$ (stopped-flow, λ = 640 nm)

[5a] (mol L^{-1})	$[4]_{tot}$ (mol L^{-1})	[4] (mol L^{-1})	[6] (mol L^{-1})	k_{obs} (s^{-1})
	3.96 x 10^{-5}	3.47 x 10^{-5}	9.54 x 10^{-4}	29.0
	5.93 x 10^{-5}	5.02 x 10^{-5}	9.41 x 10^{-4}	51.6
4.21 x 10^{-6}	7.91 x 10^{-5}	6.50 x 10^{-5}	9.14 x 10^{-4}	76.6
	9.89 x 10^{-5}	7.90 x 10^{-5}	9.00 x 10^{-4}	99.3
	1.19 x 10^{-4}	9.23 x 10^{-5}	8.87 x 10^{-4}	127

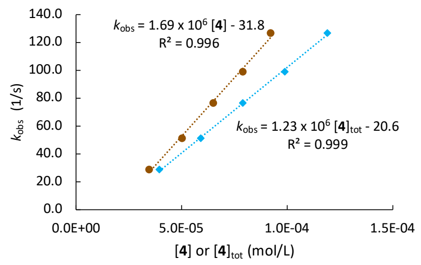

k_2^{HC} = 1.23 x 10^6 L mol^{-1} s^{-1} for $[4]_{tot}$ **$k_2^{HC,mix}$ = 1.69 x 10^6 L mol^{-1} s^{-1}** for [4]

Reaction of **4a** + **6** with $(jul)_2CH^+BF_4^-$ (stopped-flow, λ = 643 nm)

[5b] (mol L^{-1})	$[4]_{tot}$ (mol L^{-1})	[4] (mol L^{-1})	[6] (mol L^{-1})	k_{obs} (s^{-1})
	3.96 x 10^{-5}	3.47 x 10^{-5}	9.54 x 10^{-4}	59.0
	5.93 x 10^{-5}	5.02 x 10^{-5}	9.41 x 10^{-4}	119
4.06 x 10^{-6}	7.91 x 10^{-5}	6.50 x 10^{-5}	9.14 x 10^{-4}	188
	9.89 x 10^{-5}	7.90 x 10^{-5}	9.00 x 10^{-4}	235
	1.19 x 10^{-4}	9.23 x 10^{-5}	8.87 x 10^{-4}	303

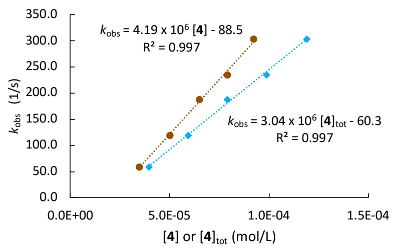

k_2^{HC} = 3.04 x 10^6 L mol^{-1} s^{-1} for $[4]_{tot}$ **$k_2^{HC,mix}$ = 4.19 x 10^6 L mol^{-1} s^{-1}** for [4]

Reaction of **4a** + **6** with $(ind)_2CH^+BF_4^-$ (stopped-flow, λ = 626 nm)

[5c] (mol L^{-1})	$[4]_{tot}$ (mol L^{-1})	[4] (mol L^{-1})	[6] (mol L^{-1})	k_{obs} (s^{-1})
	3.96 x 10^{-5}	3.47 x 10^{-5}	9.54 x 10^{-4}	188
	5.93 x 10^{-5}	5.02 x 10^{-5}	9.41 x 10^{-4}	378
4.14 x 10^{-6}	7.91 x 10^{-5}	6.50 x 10^{-5}	9.14 x 10^{-4}	588
	9.89 x 10^{-5}	7.90 x 10^{-5}	9.00 x 10^{-4}	–
	1.19 x 10^{-4}	9.23 x 10^{-5}	8.87 x 10^{-4}	846

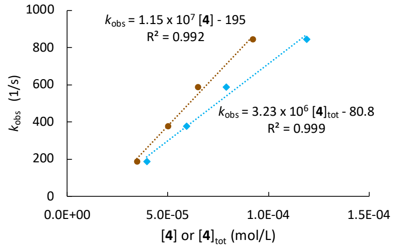

k_2^{HC} = 8.27 x 10^6 L mol^{-1} s^{-1} for $[4]_{tot}$ **$k_2^{HC,mix}$ = 1.15 x 10^7 L mol^{-1} s^{-1}** for [4]

Determination of N and s_N parameter for **4a** in DCM at constant ionic strength of 1.0 mM

Electrophile	*E*	k_2^{HC} (M^{-1} s^{-1}) for $[4]_{tot}$	$k_2^{HC,mix}$ (M^{-1} s^{-1}) for [4]
**5a**	-10.04	1.23 x 10^6	1.69 x 10^6
**5b**	-9.45	3.04 x 10^6	4.19 x 10^6
**5c**	-8.76	8.27 x 10^6	1.15 x 10^7

N = 19.35 **s_N = 0.65** for $[4]_{tot}$

N = 19.63 **s_N = 0.65** for [4]

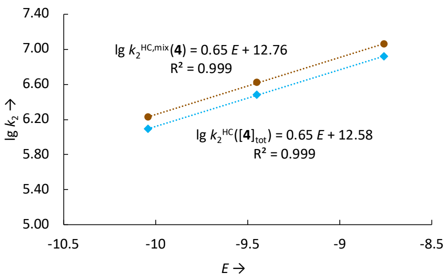

7. Kinetics of Wide Range Measurement

The herein called wide range measurements employ the same principle as the benzhydrylium kinetics except for covering a much larger concentration range (1 x 10^{-5} to 4 x 10^{-3} M). The aim of these measurement was to illustrate the influence of ion pair association on the first-order reaction rate k_{obs} (s^{1}) with increasing concentration. The hereby potentially obtained second order rate constants k_2 (M^{-1} s^{-1}) are not included in the *N* and s_N parameter determination. The benzhydrylium salt $(lil)_2CH^+BF_4^-$ (**5a**) was used as electrophile at λ = 640 nm.

7.1 Wide Range Data at 20°C

Reaction of **3a** with $(lil)_2CH^+BF_4^-$ in MeCN (stopped-flow, λ = 632 nm).

[5a] (mol L^{-1})	[3a] (mol L^{-1})	k_{obs} (s^{-1})
1.05 x 10^{-6}	1.02 x 10^{-5}	0.36
	2.04 x 10^{-5}	0.40
	4.07 x 10^{-5}	0.55
	6.11 x 10^{-5}	0.72
	8.15 x 10^{-5}	0.83
	1.02 x 10^{-4}	0.97
	2.04 x 10^{-4}	1.51
	4.07 x 10^{-4}	2.48
	5.91 x 10^{-4}	3.46
	7.94 x 10^{-4}	4.20
	9.98 x 10^{-4}	5.34
	2.00 x 10^{-3}	10.6
	4.07 x 10^{-3}	22.6

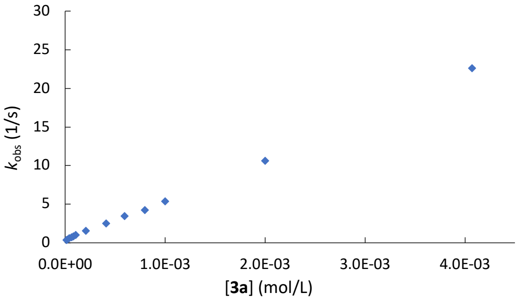

Reaction of **3a** with $(lil)_2CH^+BF_4^-$ in DCM (stopped-flow, λ = 640 nm), equilibrium constants for numerical simulation: K_{CAC} = 6.38 x 10^{6} M^{-2} for **3a** (cationic sandwich model 3a).

[5a] (mol L^{-1})	[3a] (mol L^{-1})	[3] (mol L^{-1})	k_{obs} (s^{-1})
9.62 x 10^{-7}	1.01 x 10^{-5}	1.01 x 10^{-5}	18.0
	2.01 x 10^{-5}	2.01 x 10^{-5}	36.4
	4.02 x 10^{-5}	3.98 x 10^{-5}	74.1
	6.03 x 10^{-5}	5.90 x 10^{-5}	99.2
	8.04 x 10^{-5}	7.76 x 10^{-5}	118
	1.01 x 10^{-4}	9.59 x 10^{-5}	140
	2.01 x 10^{-4}	1.76 x 10^{-4}	197
	4.02 x 10^{-4}	3.09 x 10^{-4}	292
	6.03 x 10^{-4}	4.28 x 10^{-4}	362
	8.04 x 10^{-4}	5.40 x 10^{-4}	401
	1.01 x 10^{-3}	6.52 x 10^{-4}	420

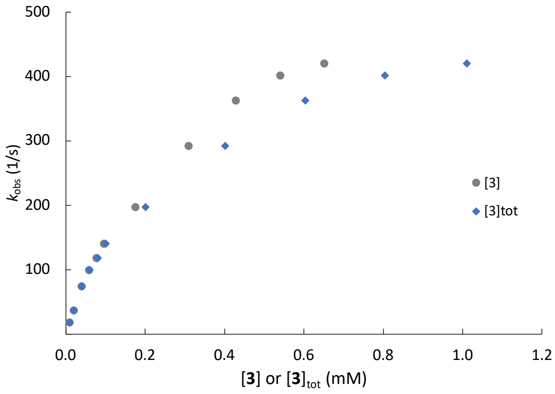

Reaction of **3a** with $(lil)_2CH^+BF_4^-$ in DCM (stopped-flow, λ = 640 nm), equilibrium constants for numerical simulation: $\alpha \times K_{CAC}$ = 2.81 x 10^6 M^{-2} and $\beta \times K_{ACA}$ = 1.34 x 10^6 M^{-2} for **3a** (α/β = 0.44/0.21) (mixed sandwich association model 4).

[5a] (mol L^{-1})	[3a] (mol L^{-1})	[3] (mol L^{-1})	[3a3] (mol L^{-1})	k_{obs} (s^{-1})
9.62 x 10^{-7}	1.01 x 10^{-5}	1.01 x 10^{-5}	1.38 x 10^{-9}	18.0
	2.01 x 10^{-5}	2.01 x 10^{-5}	1.08 x 10^{-8}	36.4
	4.02 x 10^{-5}	3.99 x 10^{-5}	8.46 x 10^{-8}	74.1
	6.03 x 10^{-5}	5.92 x 10^{-5}	2.76 x 10^{-7}	99.2
	8.04 x 10^{-5}	7.78 x 10^{-5}	6.27 x 10^{-7}	118
	1.01 x 10^{-4}	9.62 x 10^{-5}	1.18 x 10^{-6}	140
	2.01 x 10^{-4}	1.74 x 10^{-4}	6.76 x 10^{-6}	197
	4.02 x 10^{-4}	2.88 x 10^{-4}	2.91 x 10^{-5}	292
	6.03 x 10^{-4}	3.73 x 10^{-4}	6.03 x 10^{-5}	362
	8.04 x 10^{-4}	4.42 x 10^{-4}	9.65 x 10^{-5}	401
	1.01 x 10^{-3}	5.02 x 10^{-4}	1.37 x 10^{-4}	420

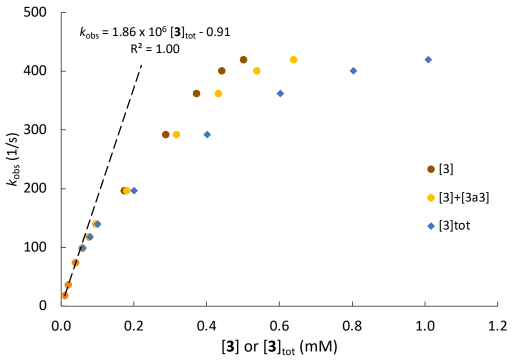

Reaction of **4a** with $(lil)_2CH^+BF_4^-$ in MeCN (stopped-flow, λ = 632 nm)

[5a] (mol L^{-1})	[4a] (mol L^{-1})	k_{obs} (s^{-1})
1.10 x 10^{-6}	1.00 x 10^{-5}	0.22
	2.01 x 10^{-5}	0.82
	4.01 x 10^{-5}	1.56
	6.02 x 10^{-5}	2.27
	8.03 x 10^{-5}	2.95
	1.00 x 10^{-4}	3.75
	2.01 x 10^{-4}	7.38
	4.01 x 10^{-4}	16.0
	6.02 x 10^{-4}	25.5
	8.03 x 10^{-4}	34.4
	1.00 x 10^{-3}	40.9
	2.01 x 10^{-3}	74.9
	4.01 x 10^{-3}	135

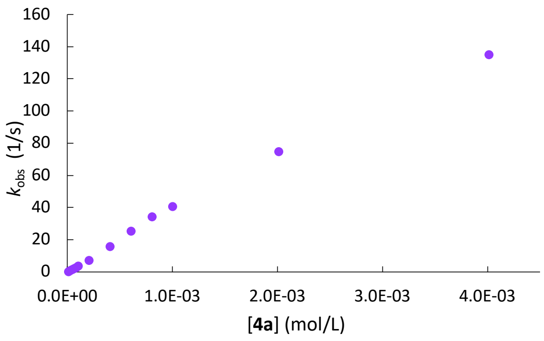

Reaction of **4a** with $(lil)_2CH^+BF_4^-$ in DCM (stopped-flow, λ = 640 nm), equilibrium constants for numerical simulation: K_{CAC} = 6.50 x 10^6 M^{-2} for **4a** (cationic sandwich model 3a).

[5a] (mol L^{-1})	[4a] (mol L^{-1})	[4] (mol L^{-1})	k_{obs} (s^{-1})
9.99 x 10^{-7}	1.01 x 10^{-5}	1.01 x 10^{-5}	50.6
	2.02 x 10^{-5}	2.02 x 10^{-5}	156
	4.05 x 10^{-5}	4.01 x 10^{-5}	291
	6.07 x 10^{-5}	5.94 x 10^{-5}	522
	8.09 x 10^{-5}	7.80 x 10^{-5}	587

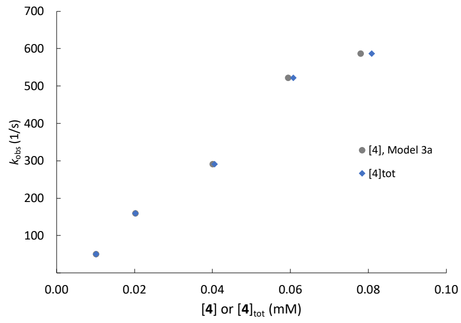

Reaction of **4a** with $(lil)_2CH^+BF_4^-$ in DCM (stopped-flow, λ = 640 nm), equilibrium constants for numerical simulation: α x K_{CAC} = 7.80 x 10^5 M^{-2} and β x K_{ACA} = 3.97 x 10^6 M^{-2} for **4a** (α/β = 0.12/0.61) (mixed sandwich association model 4).

[5a] (mol L^{-1})	[4a] (mol L^{-1})	[4] (mol L^{-1})	[4a4] (mol L^{-1})	k_{obs} (s^{-1})
9.99 x 10^{-7}	1.01 x 10^{-5}	1.01 x 10^{-5}	4.08 x 10^{-9}	50.6
	2.02 x 10^{-5}	2.02 x 10^{-5}	3.24 x 10^{-8}	156
	4.05 x 10^{-5}	4.01 x 10^{-5}	2.54 x 10^{-7}	291
	6.07 x 10^{-5}	5.94 x 10^{-5}	8.20 x 10^{-7}	522
	8.09 x 10^{-5}	7.80 x 10^{-5}	1.84 x 10^{-6}	587
	1.01 x 10^{-4}	9.59 x 10^{-5}	3.35 x 10^{-6}	–

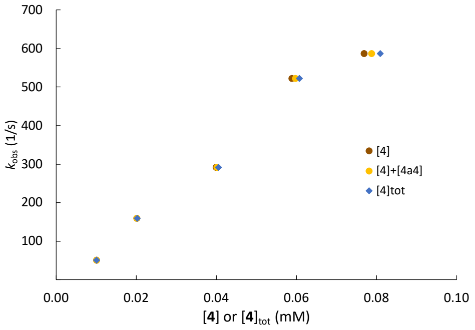

7.2 Wide Range Data of 3a at *I* = 1.0 mM at 20 °C (with 1:1 Model for Additive 6)

Reaction of **3a + 6** with $(lil)_2CH^+BF_4^-$ in DCM (stopped-flow, λ = 640 nm), equilibrium constants for numerical simulation: K_{CAC} = 6.38 x 10^6 M^{-2} for **3a**, K_{IP} = 1.01 x 10^7 M^{-2} for **6**.

[5a] (mol L^{-1})	[3a] (mol L^{-1})	[3] (mol L^{-1})	[6] (mol L^{-1})	k_{obs} (s^{-1})
1.18 x 10^{-6}	9.98 x 10^{-6}	2.98 x 10^{-6}	9.83 x 10^{-4}	1.91
	2.00 x 10^{-5}	6.05 x 10^{-6}	9.83 x 10^{-4}	4.34
	3.99 x 10^{-5}	1.25 x 10^{-5}	9.63 x 10^{-3}	12.0
	5.99 x 10^{-5}	1.93 x 10^{-5}	9.43 x 10^{-4}	20.1
	7.98 x 10^{-5}	2.66 x 10^{-5}	9.24 x 10^{-4}	29.2
	9.98 x 10^{-5}	3.43 x 10^{-5}	9.04 x 10^{-4}	36.5
	2.00 x 10^{-4}	7.77 x 10^{-5}	8.06 x 10^{-4}	58.6
	3.99 x 10^{-4}	1.93 x 10^{-4}	5.99 x 10^{-4}	153
	5.99 x 10^{-4}	3.31 x 10^{-4}	4.03 x 10^{-4}	225
	7.98 x 10^{-4}	4.84 x 10^{-4}	1.97 x 10^{-4}	328
	9.98 x 10^{-4}	6.45 x 10^{-4}	–	421

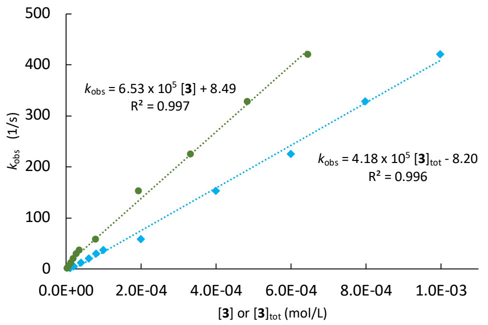

7.3 Wide Range Data at *I* = 1.0 mM at 20 °C (with Cationic Sandwich Model for Additive 6)

Reaction of **3a + 6** with $(lil)_2CH^+BF_4^-$ in DCM (stopped-flow, λ = 640 nm), equilibrium constants for numerical simulation: K_{CAC} = 6.38 x 10^6 M^{-2} for **3a**, K_{CAC} = 7.05 x 10^6 M^{-2} for **6**.

[5a] (mol L^{-1})	[3a] (mol L^{-1})	[3] (mol L^{-1})	[6] (mol L^{-1})	k_{obs} (s^{-1})
1.18 x 10^{-6}	9.98 x 10^{-6}	6.63 x 10^{-6}	9.83 x 10^{-4}	1.91
	2.00 x 10^{-5}	1.33 x 10^{-5}	9.83 x 10^{-4}	4.34
	3.99 x 10^{-5}	2.65 x 10^{-5}	9.63 x 10^{-3}	12.0
	5.99 x 10^{-5}	3.98 x 10^{-5}	9.43 x 10^{-4}	20.1
	7.98 x 10^{-5}	5.30 x 10^{-5}	9.24 x 10^{-4}	29.2
	9.98 x 10^{-5}	6.62 x 10^{-5}	9.04 x 10^{-4}	36.5
	2.00 x 10^{-4}	1.32 x 10^{-4}	8.06 x 10^{-4}	58.6
	3.99 x 10^{-4}	2.62 x 10^{-4}	5.99 x 10^{-4}	153
	5.99 x 10^{-4}	3.92 x 10^{-4}	4.03 x 10^{-4}	225
	7.98 x 10^{-4}	5.19 x 10^{-4}	1.97 x 10^{-4}	328
	9.98 x 10^{-4}	6.46 x 10^{-4}	–	421

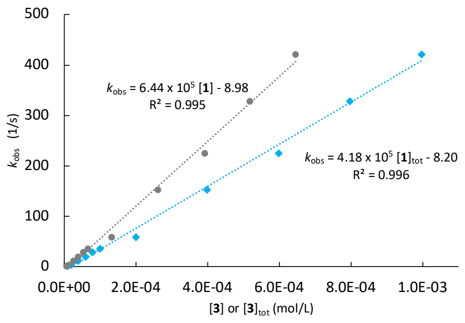

Reaction of **4a + 6** with $(lil)_2CH^+BF_4^-$ in DCM (stopped-flow, λ = 640 nm), equilibrium constants for numerical simulation: K_{CAC} = 6.50 x 10^6 M^{-2} for **4a**, K_{CAC} = 7.05 x 10^6 M^{-2} for **6**.

[5a] (mol L^{-1})	[4a] (mol L^{-1})	[4] (mol L^{-1})	[6] (mol L^{-1})	k_{obs} (s^{-1})
1.15 x 10^{-6}	9.93 x 10^{-6}	6.55 x 10^{-6}	9.90 x 10^{-4}	
	1.99 x 10^{-5}	1.31 x 10^{-5}	9.79 x 10^{-4}	1.09
	3.97 x 10^{-5}	2.62 x 10^{-5}	9.56 x 10^{-3}	27.1
	5.96 x 10^{-5}	3.93 x 10^{-5}	9.45 x 10^{-4}	48.7
	7.94 x 10^{-5}	5.23 x 10^{-5}	9.23 x 10^{-4}	63.4
	9.93 x 10^{-5}	6.54 x 10^{-5}	9.00 x 10^{-4}	100
	1.99 x 10^{-4}	1.31 x 10^{-4}	7.99 x 10^{-4}	306

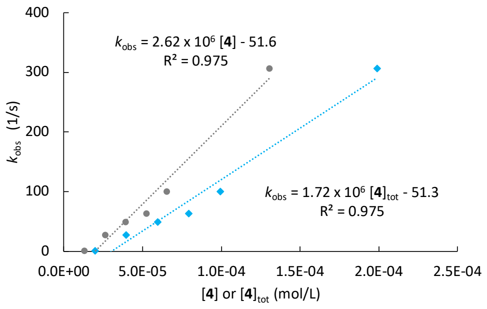

7.4 Wide Range Data at *I* = 1.0 mM at 20 °C (with Mixed Sandwich Model Extension)

Reaction of **3a + 6** with $(lil)_2CH^+BF_4^-$ in DCM (stopped-flow, λ = 640 nm), equilibrium constants for numerical simulation: α x K_{CAC} = 2.81 x 10^6 M^{-2} and β x K_{ACA} = 1.34 x 10^6 M^{-2} for **3a** (α/β = 0.44/0.21), K_{CAC} = 7.05 x 10^6 M^{-2} for **6**.

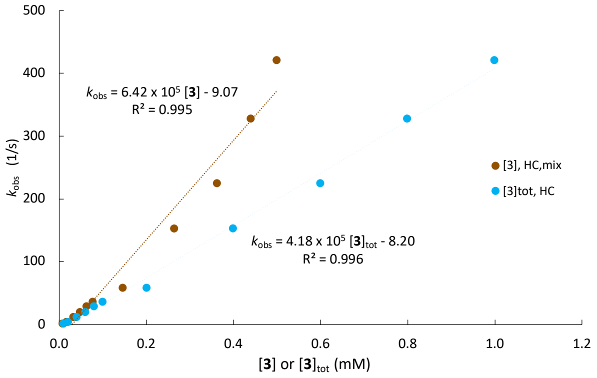

[5a] (mol L^{-1})	[3a] (mol L^{-1})	[3] (mol L^{-1})	[3a3] (mol L^{-1})	[6] (mol L^{-1})	k_{obs} (s^{-1})
1.18 x 10^{-6}	9.98 x 10^{-6}	8.12 x 10^{-6}	2.49 x 10^{-8}	9.83 x 10^{-4}	1.91
	2.00 x 10^{-5}	1.62 x 10^{-5}	9.91 x 10^{-8}	9.83 x 10^{-4}	4.34
	3.99 x 10^{-5}	3.19 x 10^{-5}	3.87 x 10^{-7}	9.63 x 10^{-3}	12.0
	5.99 x 10^{-5}	4.73 x 10^{-5}	8.58 x 10^{-7}	9.43 x 10^{-4}	20.1
	7.98 x 10^{-5}	6.23 x 10^{-5}	1.50 x 10^{-6}	9.24 x 10^{-4}	29.2
	9.98 x 10^{-5}	7.71 x 10^{-5}	2.30 x 10^{-6}	9.04 x 10^{-4}	36.5
	2.00 x 10^{-4}	1.46 x 10^{-4}	8.57 x 10^{-6}	8.06 x 10^{-4}	58.6
	3.99 x 10^{-4}	2.64 x 10^{-4}	2.98 x 10^{-5}	5.99 x 10^{-4}	153
	5.99 x 10^{-4}	3.62 x 10^{-4}	5.97 x 10^{-5}	4.03 x 10^{-4}	225
	7.98 x 10^{-4}	4.39 x 10^{-4}	9.54 x 10^{-5}	1.97 x 10^{-4}	328
	9.98 x 10^{-4}	4.99 x 10^{-4}	1.35 x 10^{-4}	–	421

Reaction of **4a + 6** with $(lil)_2CH^+BF_4^-$ in DCM (stopped-flow, λ = 640 nm), equilibrium constants for numerical simulation: α x K_{CAC} = 7.80 x 10^5 M^{-2} and β x K_{ACA} = 3.97 x 10^6 M^{-2} for **4a** (/β = 0.12/0.61), K_{CAC} = 7.05 x 10^6 M^{-2} for **6**.

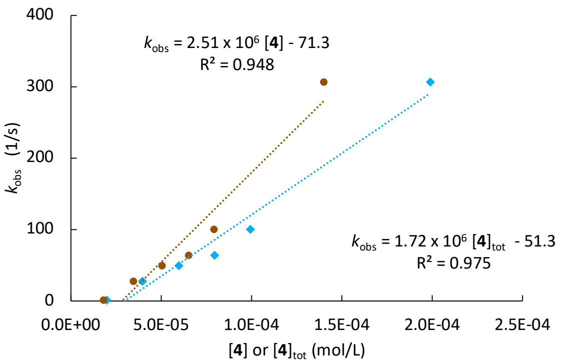

[5a] (mol L^{-1})	[4a] (mol L^{-1})	[4] (mol L^{-1})	[4a4] (mol L^{-1})	[6] (mol L^{-1})	k_{obs} (s^{-1})
1.15 x 10^{-6}	9.93 x 10^{-6}	9.17 x 10^{-6}	9.45 x 10^{-8}	9.90 x 10^{-4}	
	1.99 x 10^{-5}	1.80 x 10^{-5}	3.67 x 10^{-7}	9.79 x 10^{-4}	1.09
	3.97 x 10^{-5}	3.47 x 10^{-5}	1.37 x 10^{-6}	9.56 x 10^{-3}	27.1
	5.96 x 10^{-5}	5.05 x 10^{-5}	2.92 x 10^{-6}	9.45 x 10^{-4}	48.7
	7.94 x 10^{-5}	6.52 x 10^{-5}	4.93 x 10^{-6}	9.23 x 10^{-4}	63.4
	9.93 x 10^{-5}	7.92 x 10^{-5}	7.35 x 10^{-6}	9.00 x 10^{-4}	100
	1.99 x 10^{-4}	1.40 x 10^{-4}	2.41 x 10^{-5}	7.99 x 10^{-4}	306

8. Crystallographic Data

Tetraphenylphosphonium pyridin-4-yl((trifluoromethyl)sulfonyl)amide (**3a**)

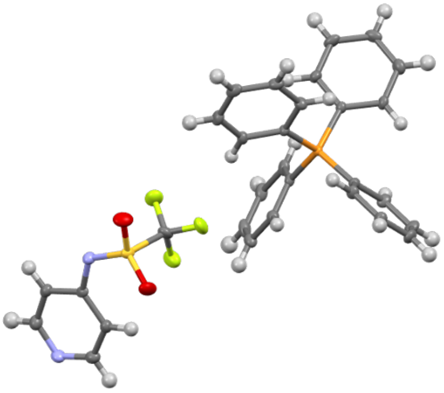

Table S28. Crystallographic details for ion pair catalyst **3a**.

net formula	$C_{30}H_{24}F_3N_2O_2PS$
M_r/g mol^{-1}	564.54
crystal size/mm	0.100 × 0.050 × 0.040
T/K	102.(2)
radiation	MoKα
diffractometer	'Bruker D8 Venture TXS'
crystal system	orthorhombic
space group	'P n a 21'
a/Å	20.5906(10)
b/Å	34.3669(14)
c/Å	14.7169(6)
α/°	90
β/°	90
γ/°	90
V/Å^3	10414.2(8)
Z	16
calc. density/g cm^{-3}	1.440
μ/mm^{-1}	0.240
absorption correction	Multi-Scan
transmission factor range	0.97–0.99
refls. measured	183497
R_{int}	0.0575
mean σ(I)/I	0.0349
θ range	2.306–27.103
observed refls.	20396
x, y (weighting scheme)	0.0737, 5.0008
hydrogen refinement	constr
Flack parameter	−0.024(15)
refls in refinement	22927
parameters	1405
restraints	1
R(F_{obs})	0.0458
R_w(F^2)	0.1260
S	1.040
shift/error$_{max}$	0.001
max electron density/e Å^{-3}	0.894
min electron density/e Å^{-3}	−0.324

Tetraphenylphosphonium tetrafluoroborate (**6**)

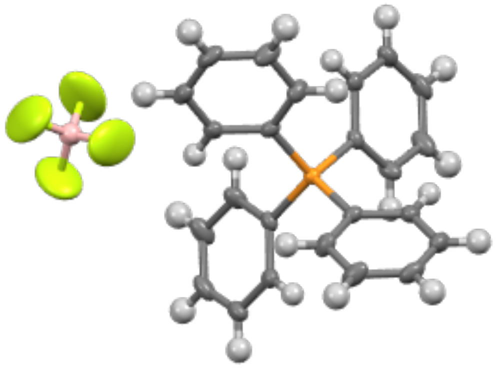

Table S29. Crystallographic details for tetraphenylphosphonium tetrafluoroborate (**6**).

net formula	$C_{24}H_{20}BF_4P$
M_r/g mol^{-1}	426.18
crystal size/mm	0.100 × 0.080 × 0.060
T/K	173.(2)
radiation	MoKα
diffractometer	'Bruker D8 Venture TXS'
crystal system	tetragonal
space group	'I -4'
a/Å	12.0191(4)
b/Å	12.0191(4)
c/Å	6.8981(3)
α/°	90
β/°	90
γ/°	90
V/Å^3	996.49(8)
Z	2
calc. density/g cm^{-3}	1.420
μ/mm^{-1}	0.183
absorption correction	Multi-Scan
transmission factor range	0.97–0.99
refls. measured	8833
R_{int}	0.0318
mean σ(*I*)/*I*	0.0179
θ range	3.390–27.094
observed refls.	1070
x, y (weighting scheme)	0.0470, 0.7488
hydrogen refinement	constr
Flack parameter	−0.04(3)
refls in refinement	1098
parameters	73
restraints	0
$R(F_{obs})$	0.0345
$R_w(F^2)$	0.0919
S	1.106
shift/error$_{max}$	0.001
max electron density/e Å^{-3}	0.432
min electron density/e Å^{-3}	−0.189

The X-ray crystal structure of compound **6** is consistent with previously reported X-ray crystal data by Chen *et al.*[25]

9. NMR Spectra of Newly Synthesized Compounds

Tetraphenylphosphonium pyridin-4-yl((trifluoromethyl)sulfonyl)amide (**3a**)

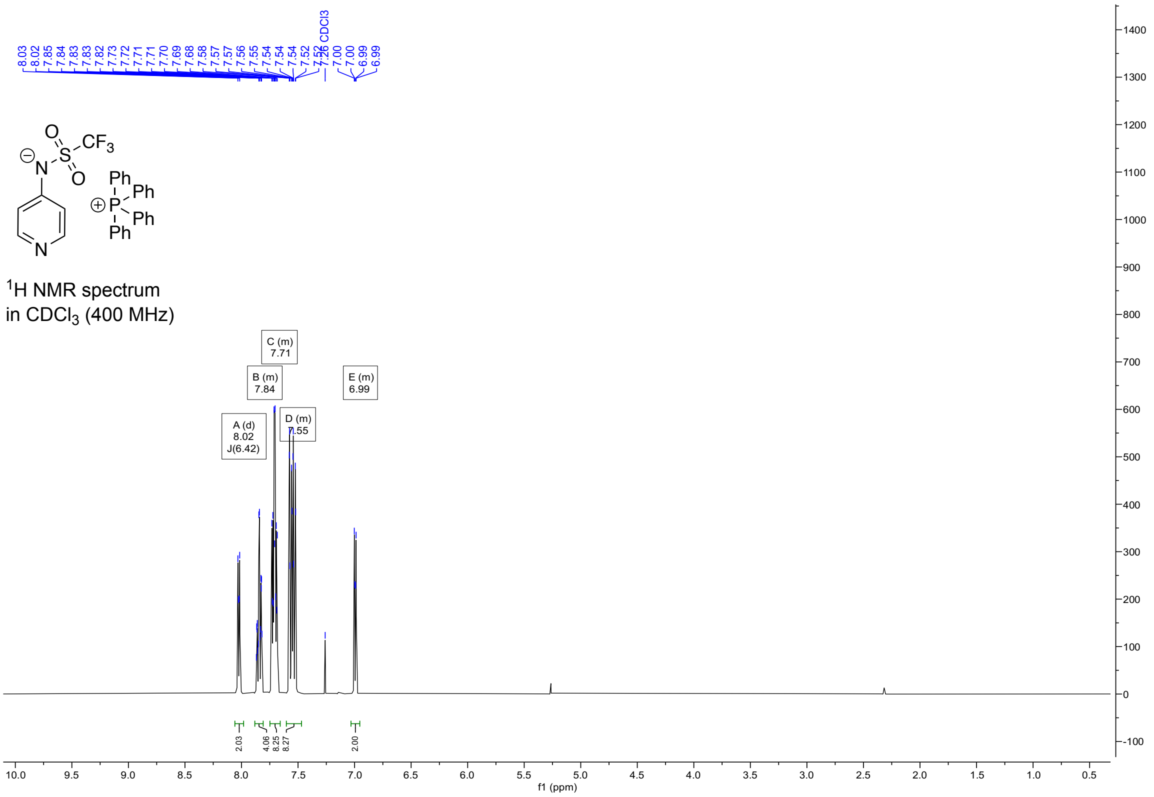

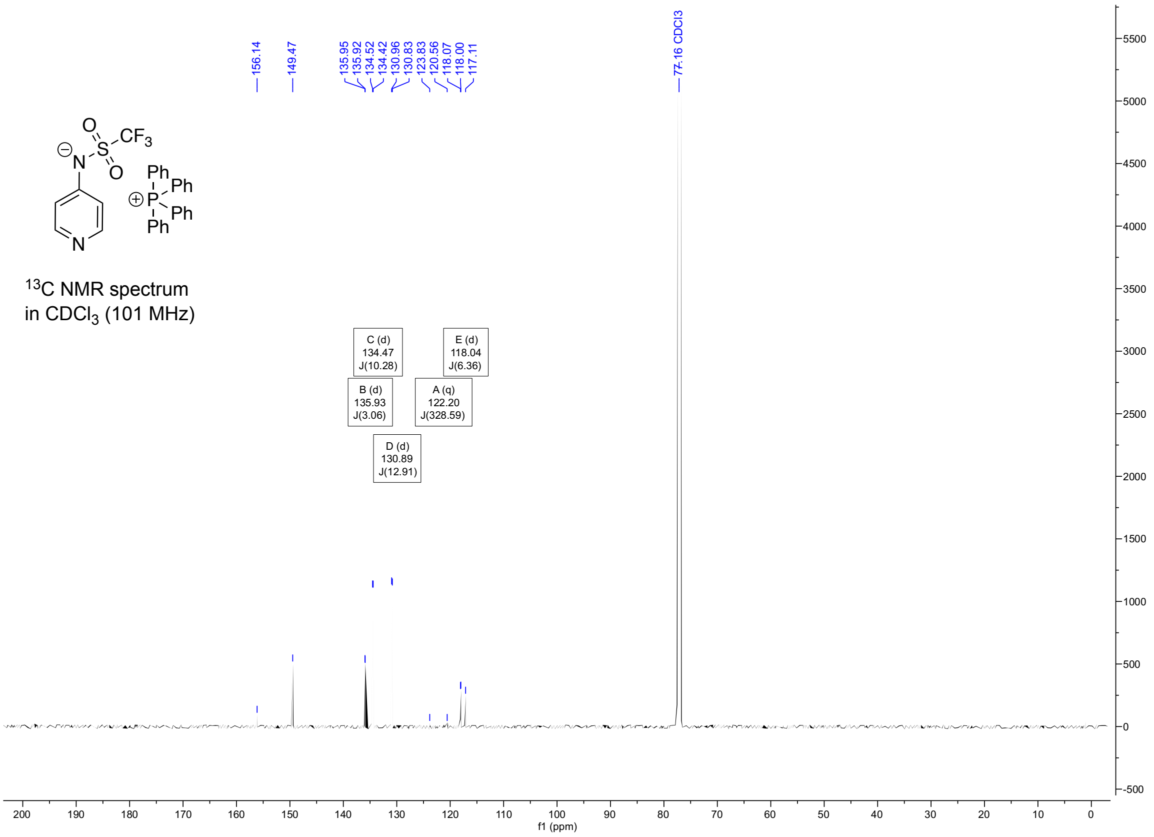

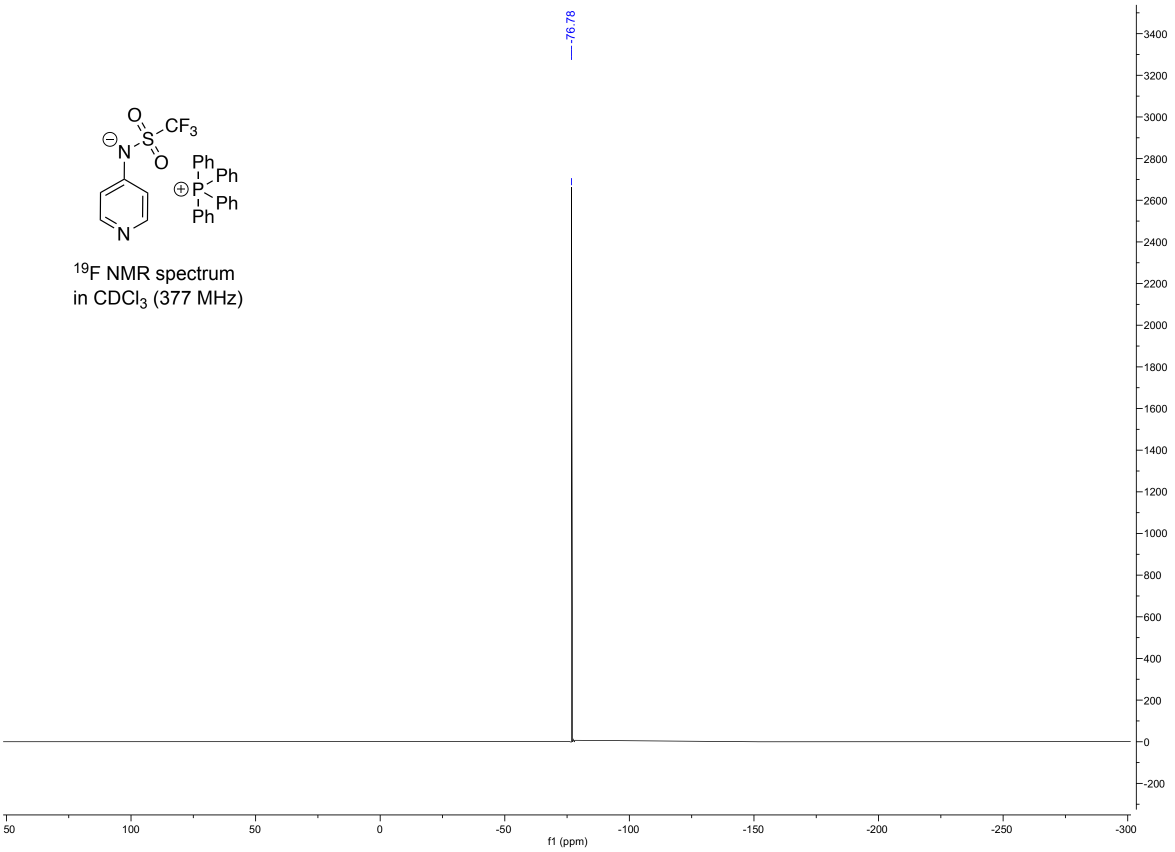

^{19}F NMR spectrum
in $CDCl_3$ (377 MHz)

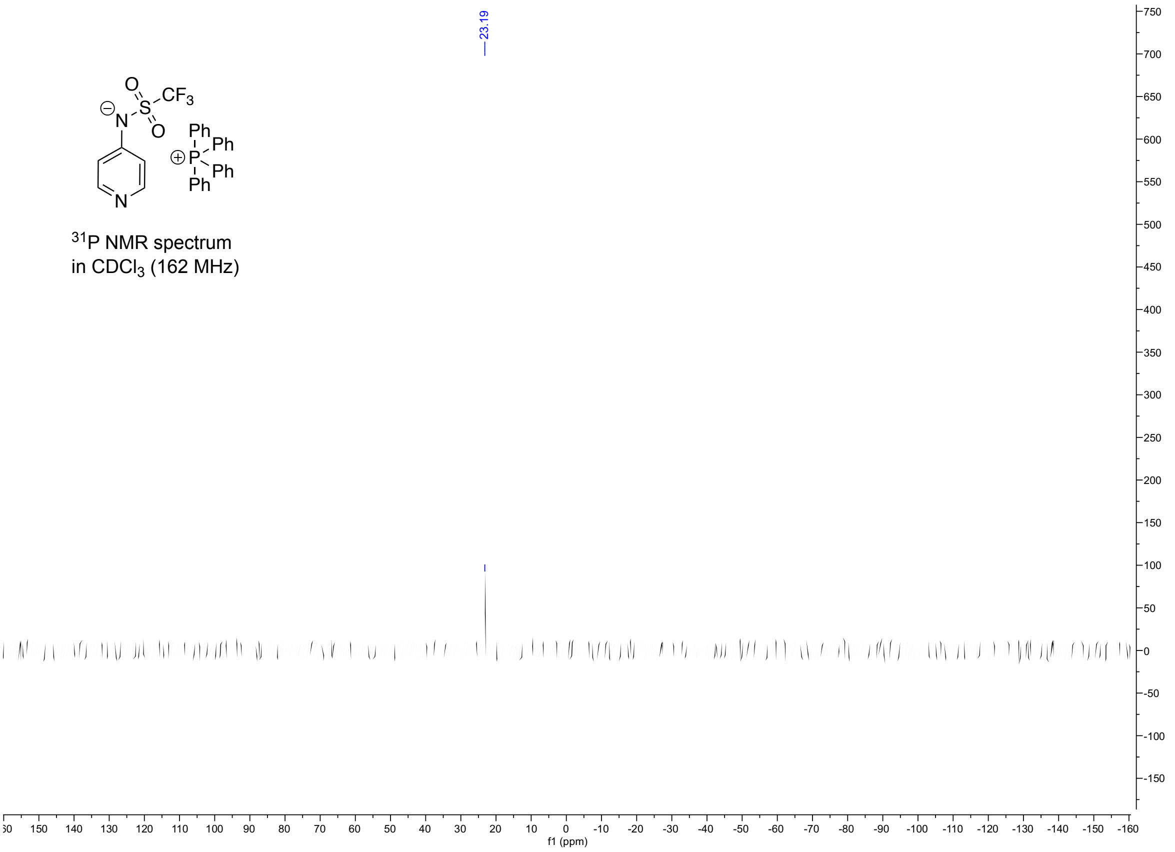

^{31}P NMR spectrum
in $CDCl_3$ (162 MHz)

Tetraphenylphosphonium tetrafluoroborate (**6**)

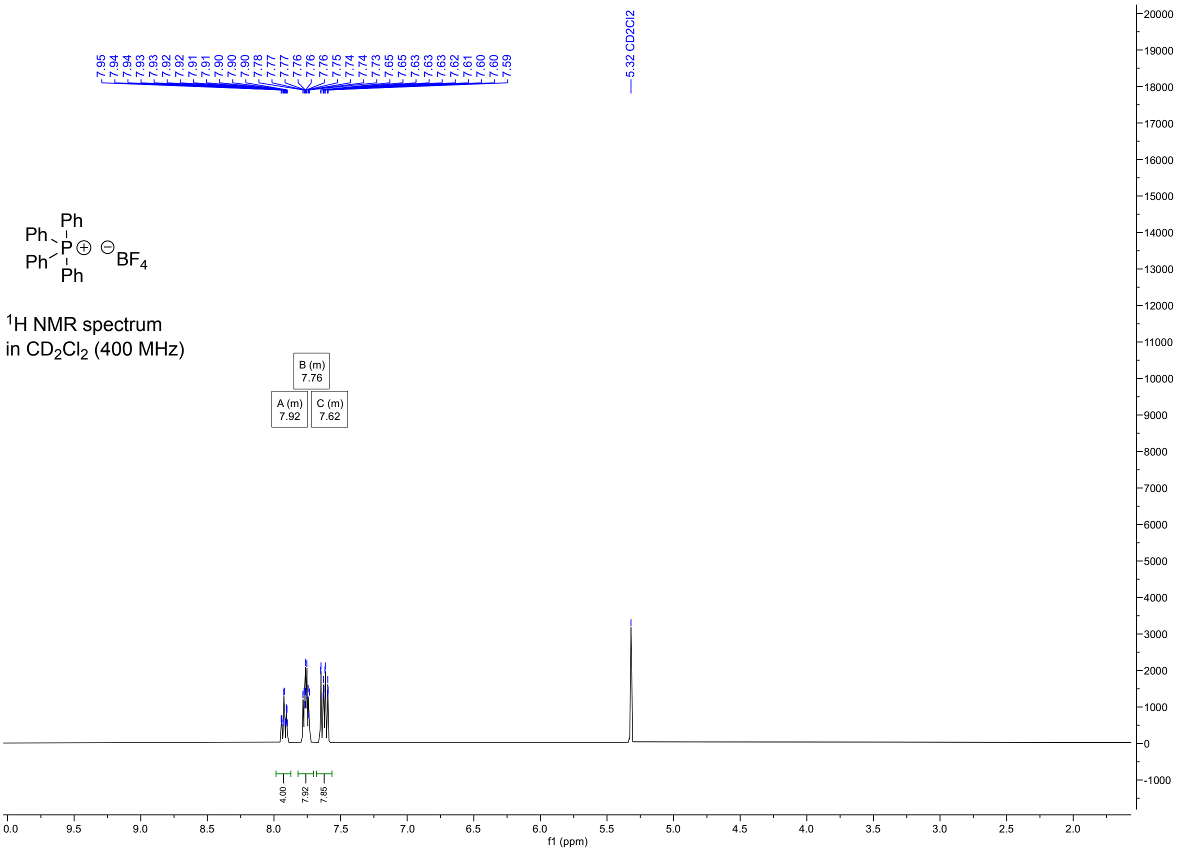

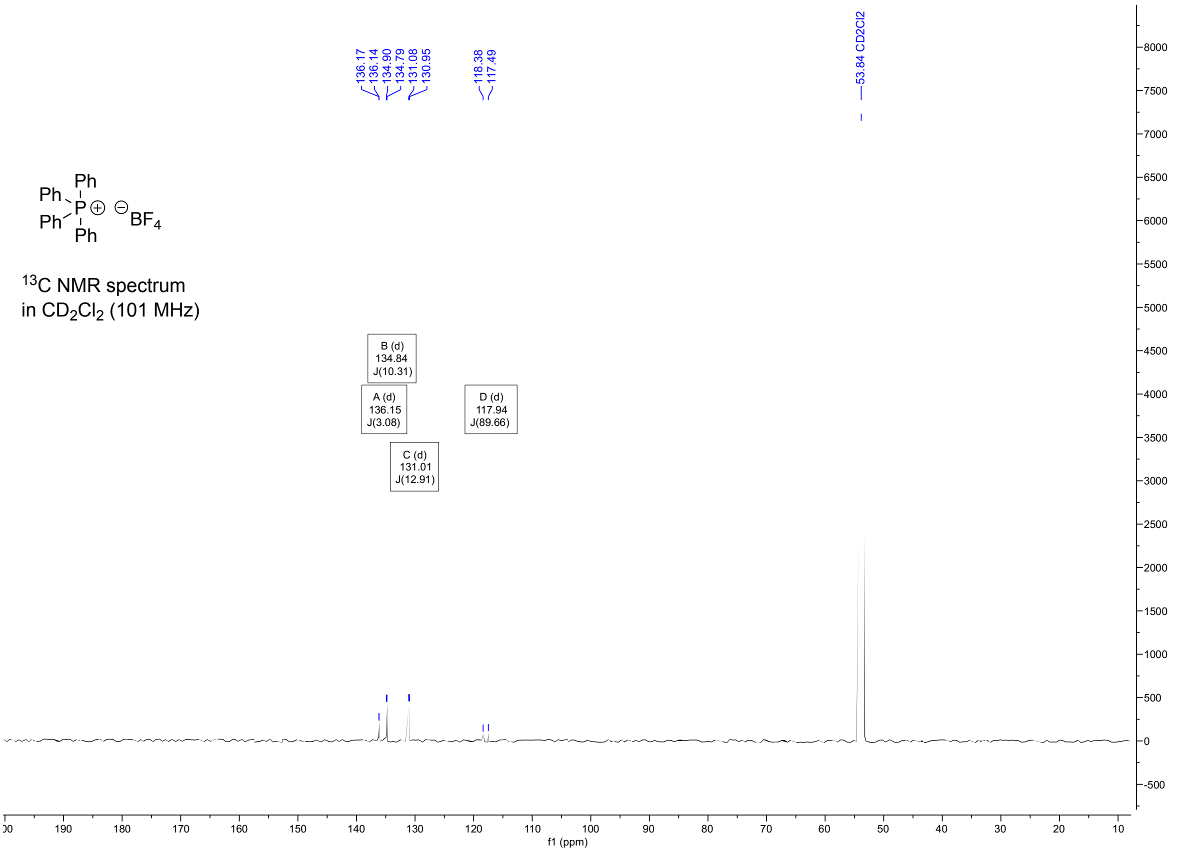

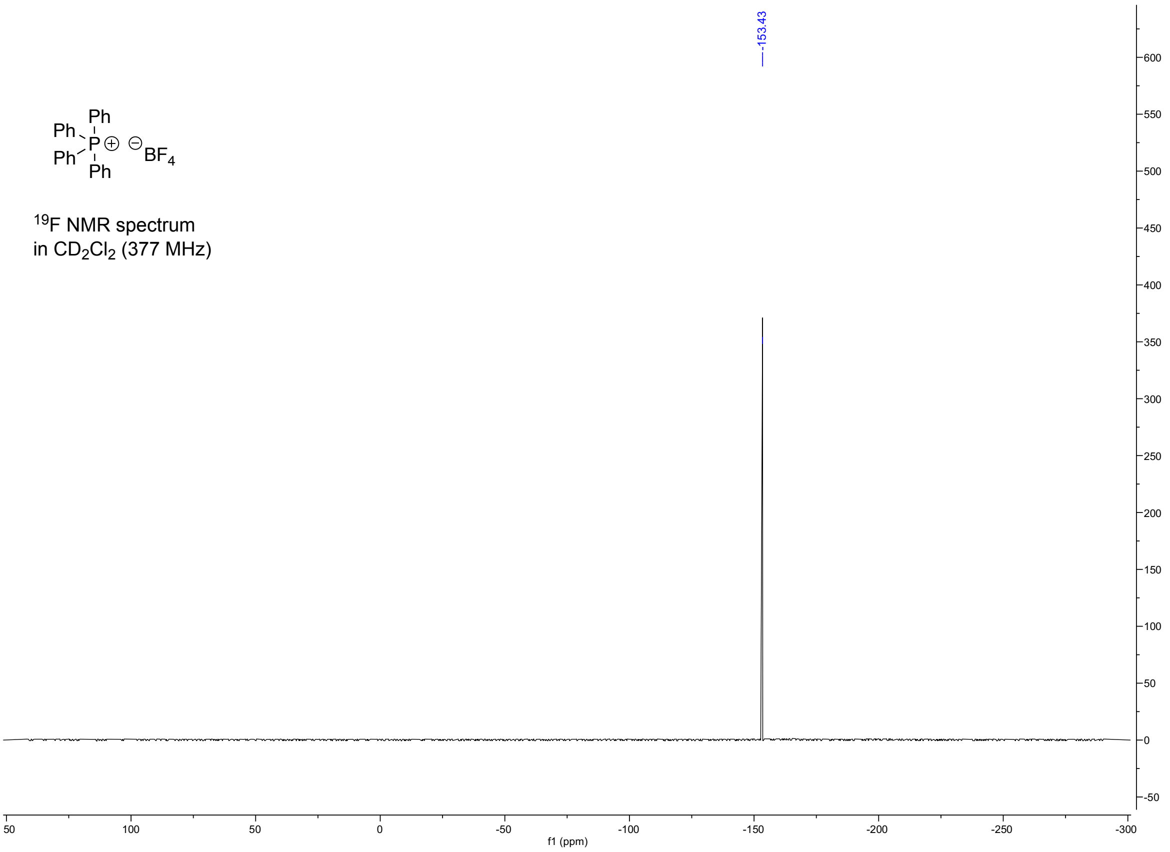
-153.43
Ph
Ph
P
BF4
Ph
Ph
19F NMR spectrum
in CD2Cl2 (377 MHz)
f1 (ppm)

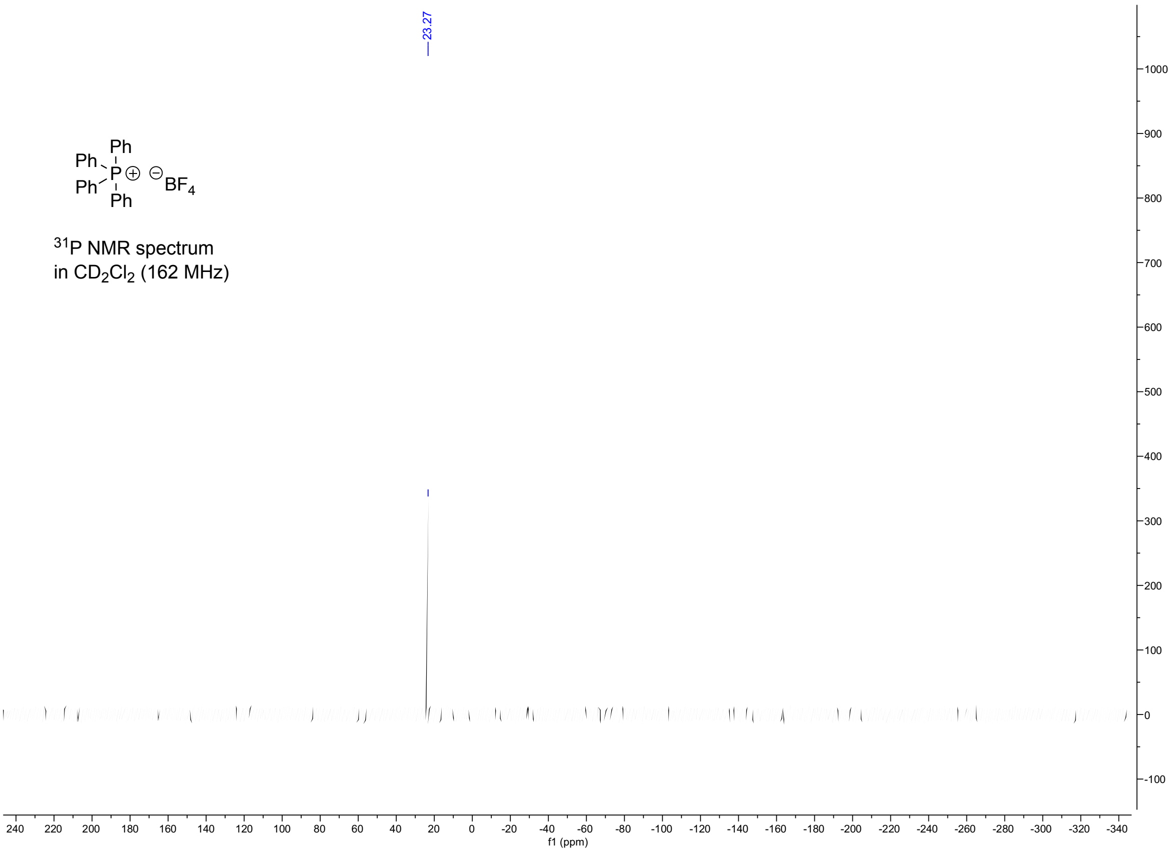
23.27
Ph
Ph
P
BF4
Ph
Ph
31P NMR spectrum
in CD2Cl2 (162 MHz)
f1 (ppm)

10. DOSY Fits

DOSY curve fits for one signal of each ion are displayed in the following. In addition, the DOSY curve fits of TMS, which was used as reference is also given for each sample. Fitting of the data points was conducted according to the Stejskal-Tanner equation[17,26]. The corresponding R^2 of all measurements are given in the tables of chapter 4.

a. Ion Pair 3a

0.005 mM

Anion

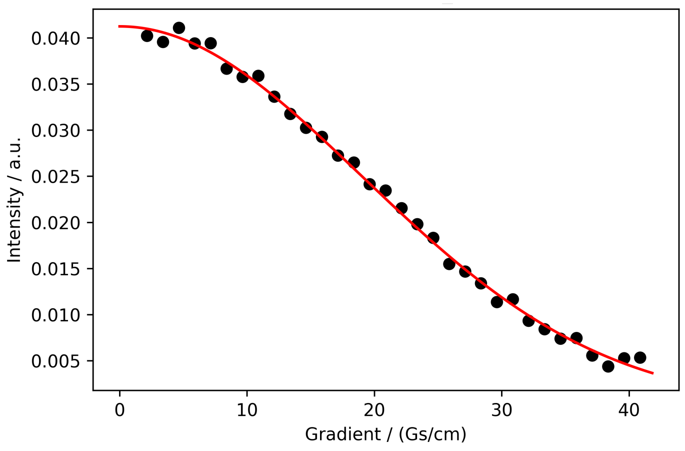

Cation

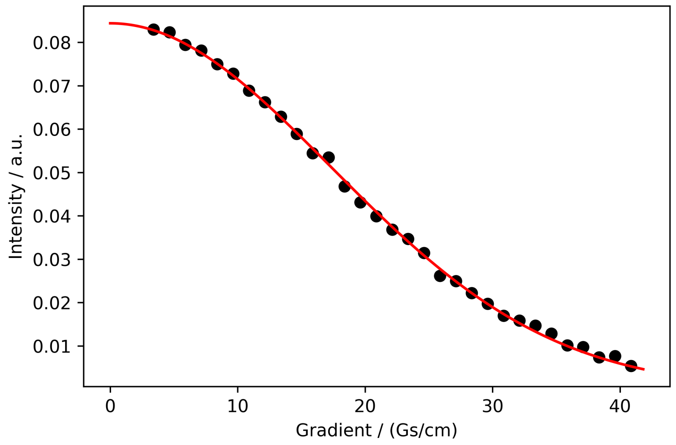

TMS

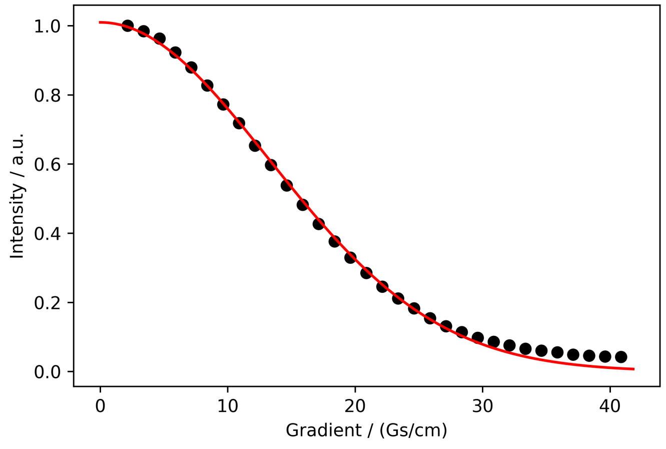

0.01 mM

Anion

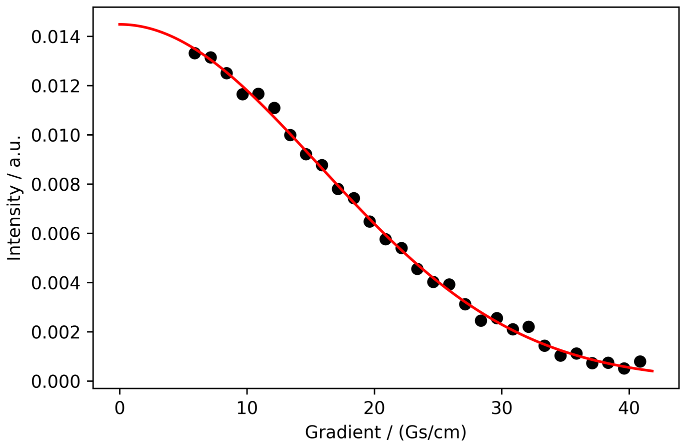

Cation

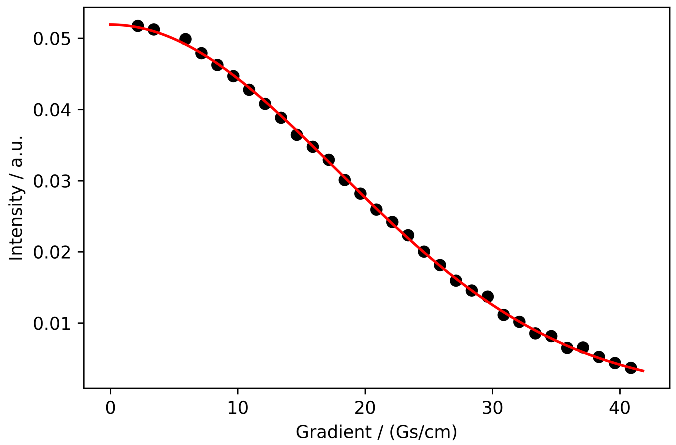

TMS

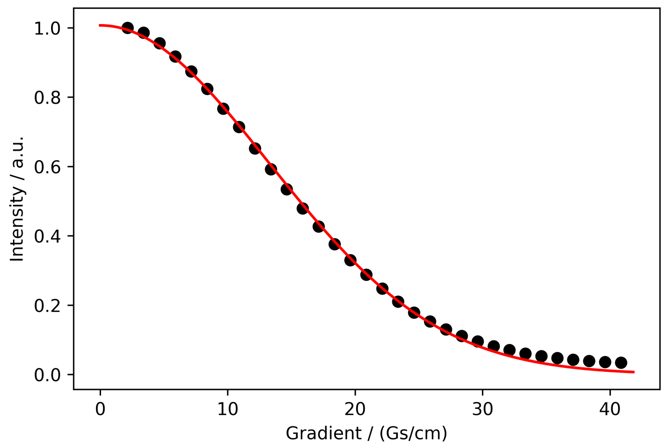

0.05 mM

Anion

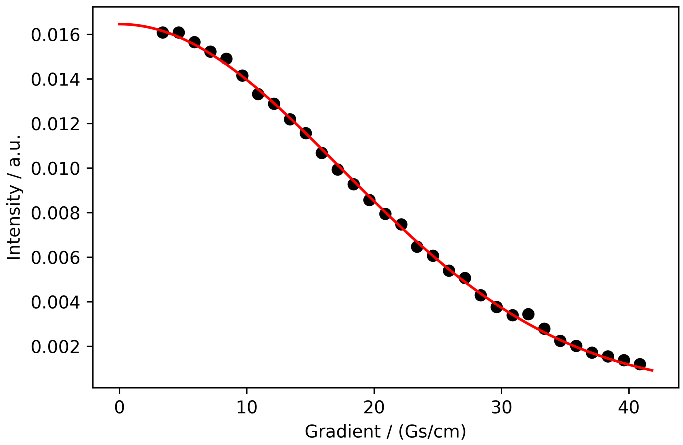

Cation

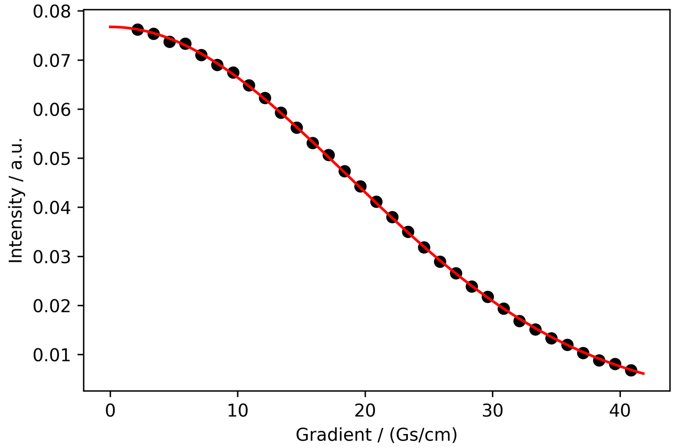

TMS

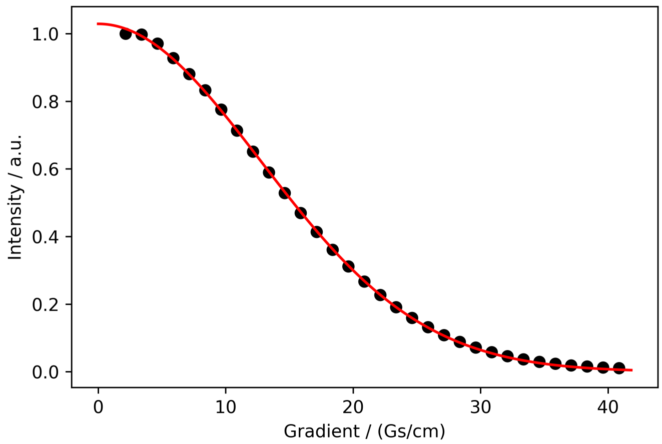

0.1 mM

Anion

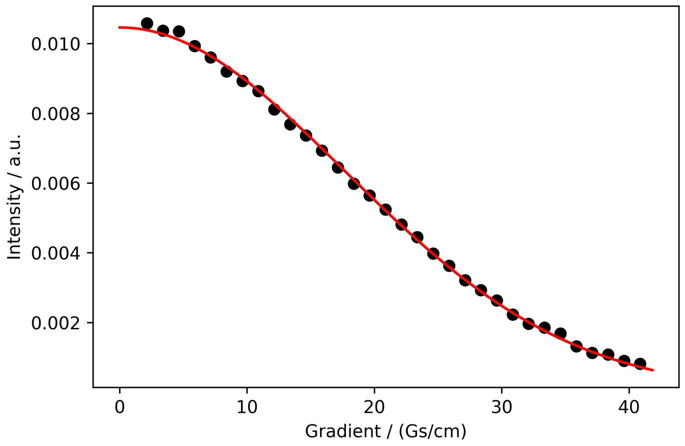

Cation

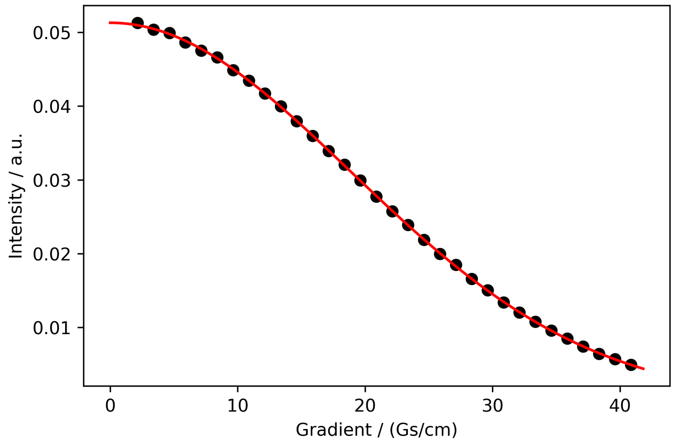

TMS

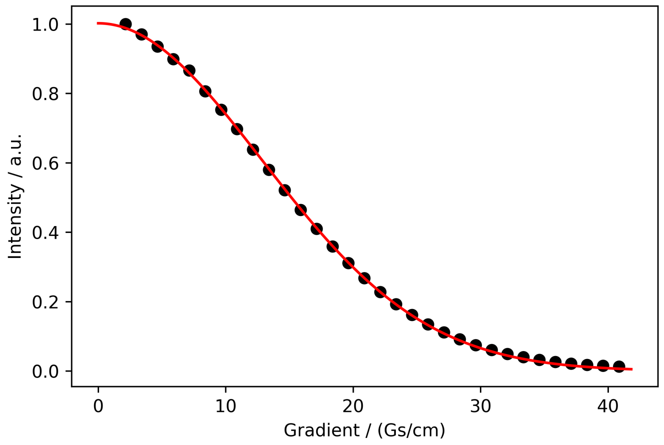

0.2 mM

Anion

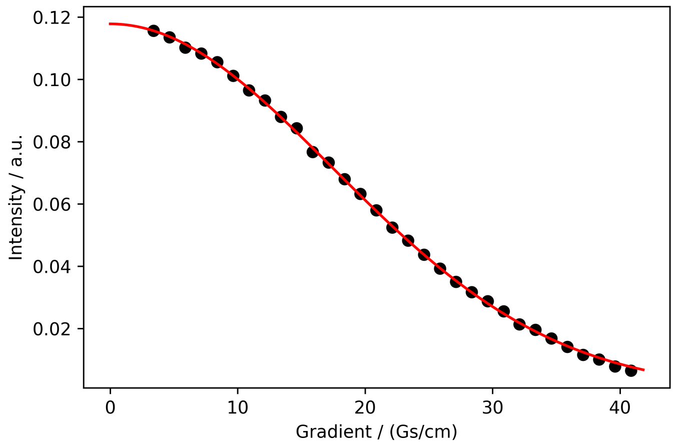

Cation

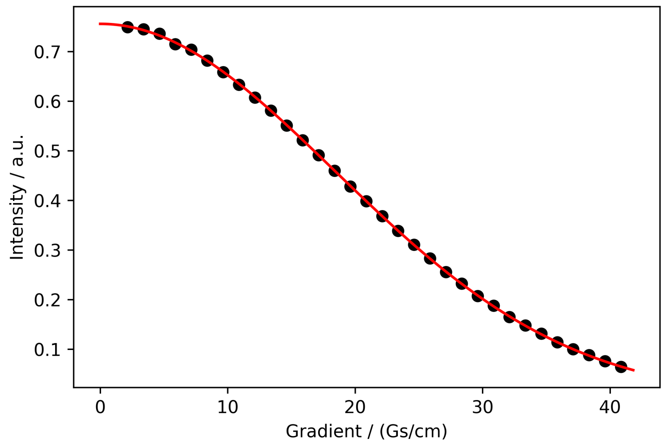

TMS

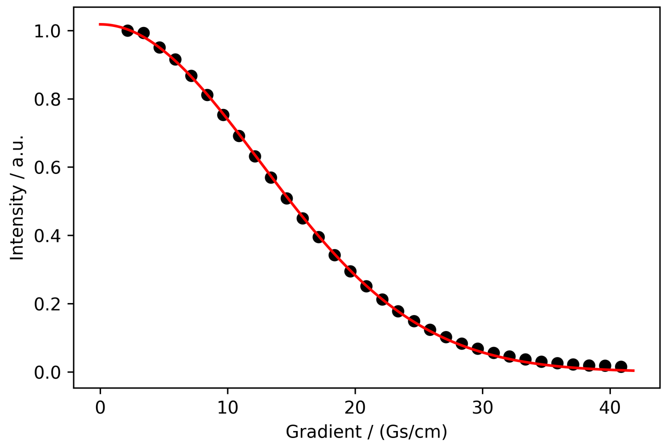

0.4 mM

Anion

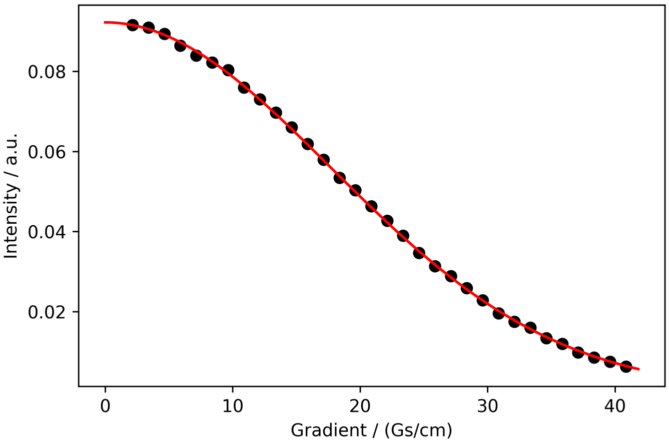

Cation

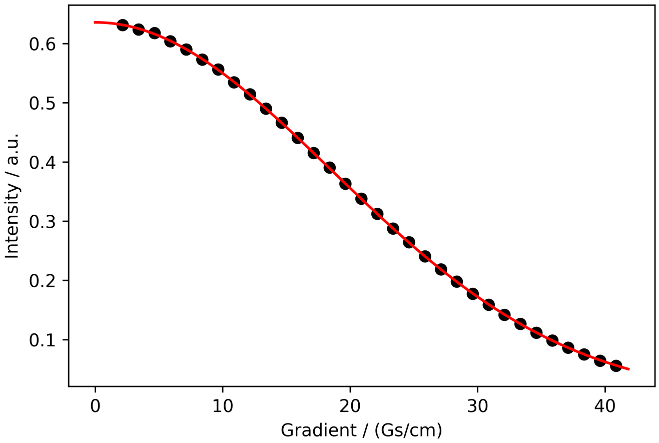

TMS

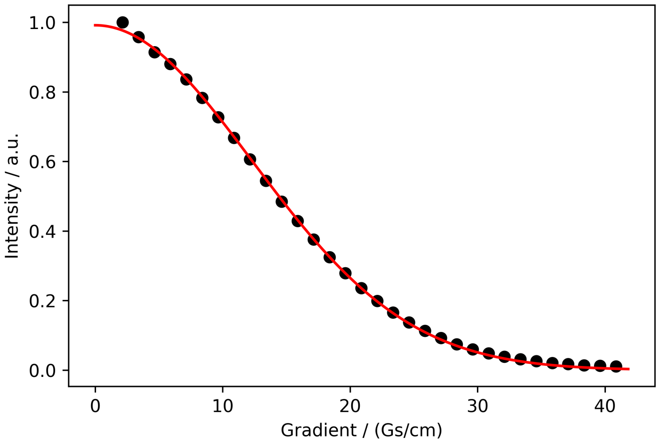

0.6 mM

Anion

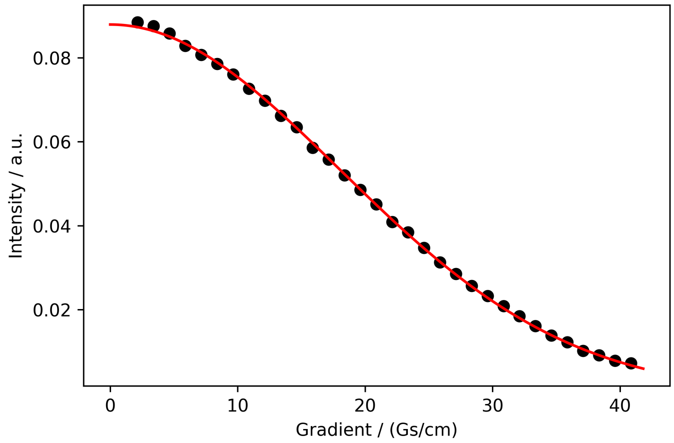

Cation

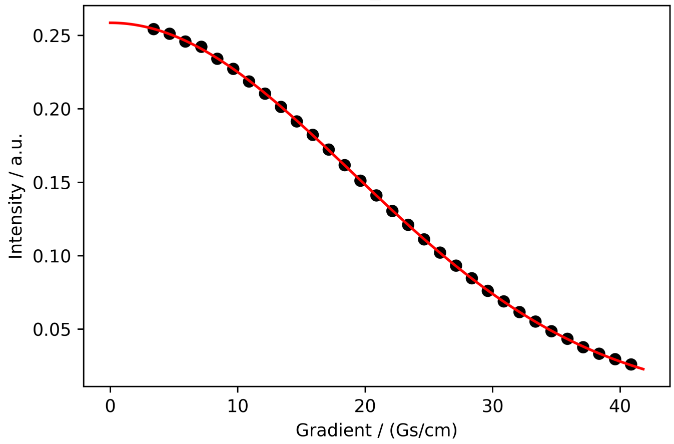

TMS

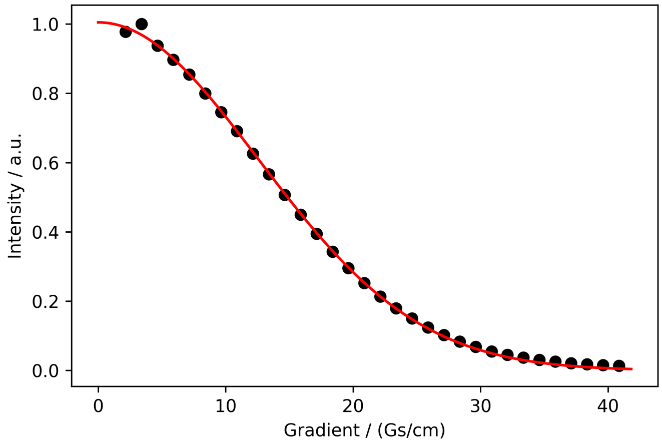

0.8 mM

Anion

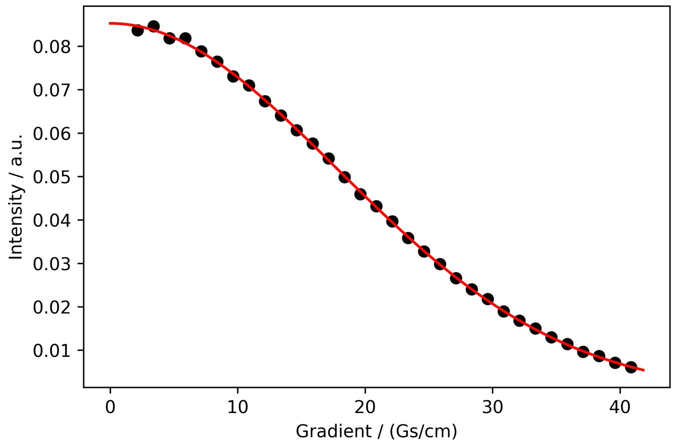

Cation

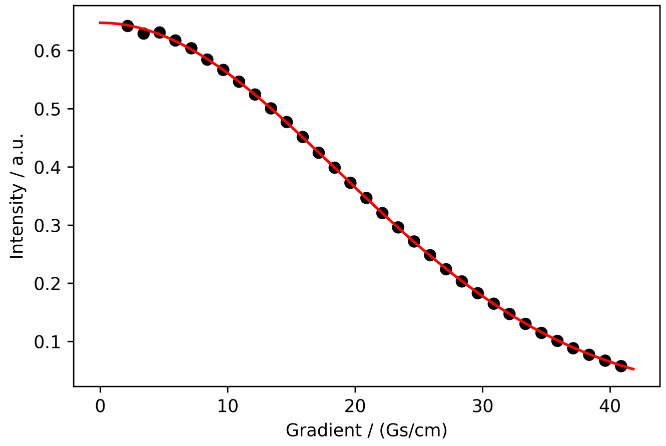

TMS

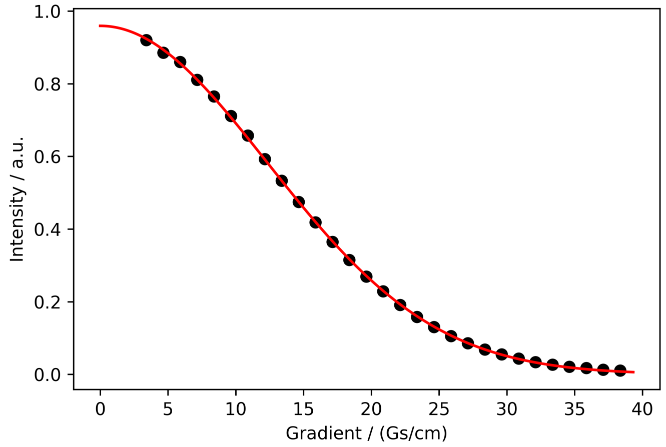

1.0 mM

Anion

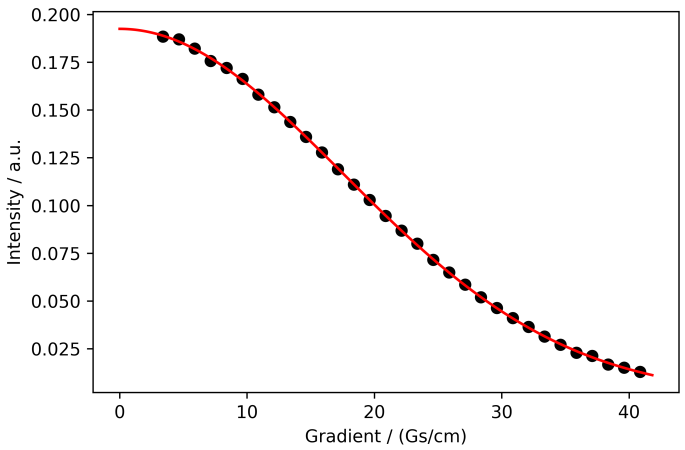

Cation

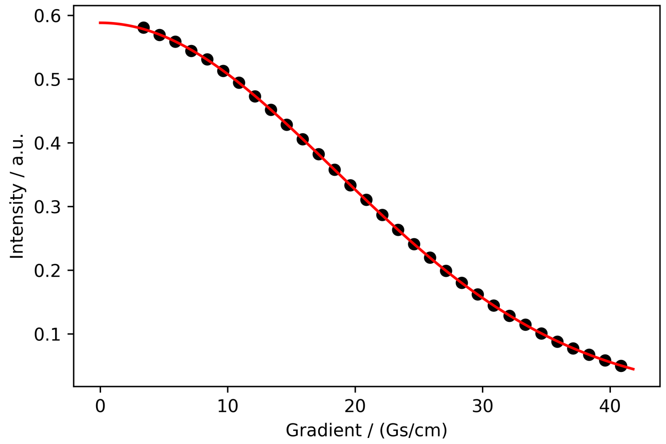

TMS

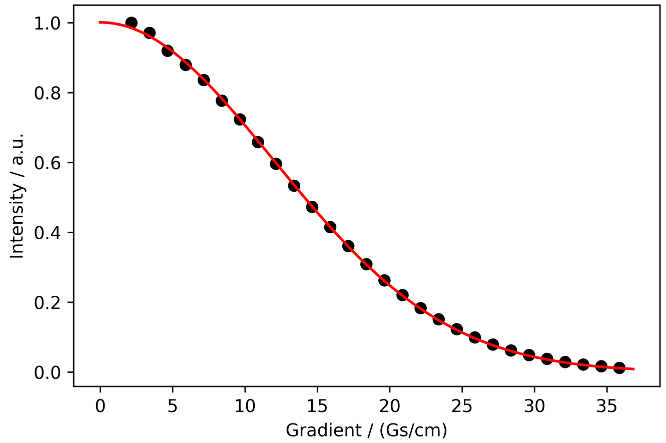

b. Ion Pair 4a

0.005 mM

Anion

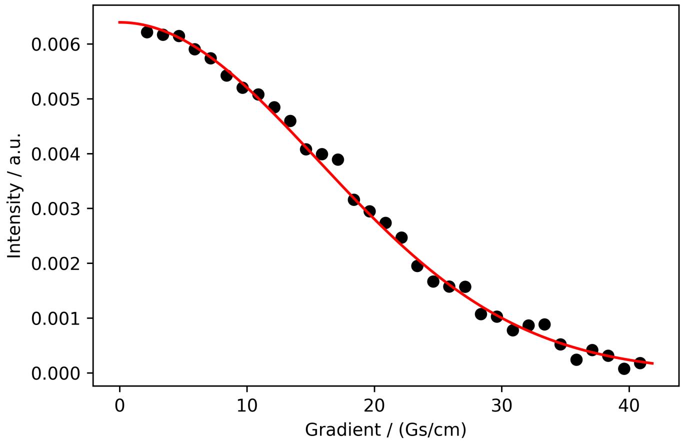

Cation

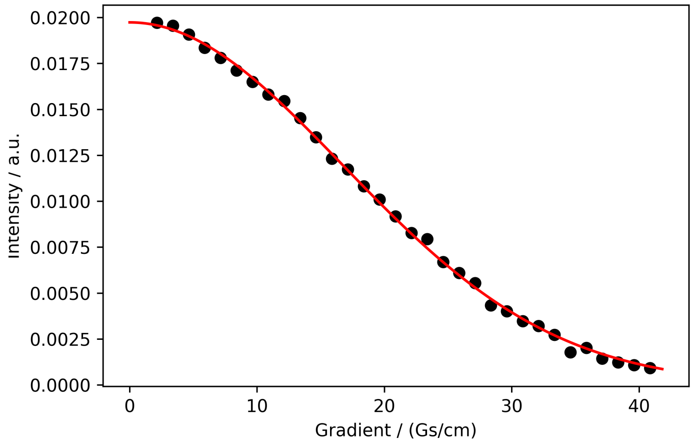

TMS

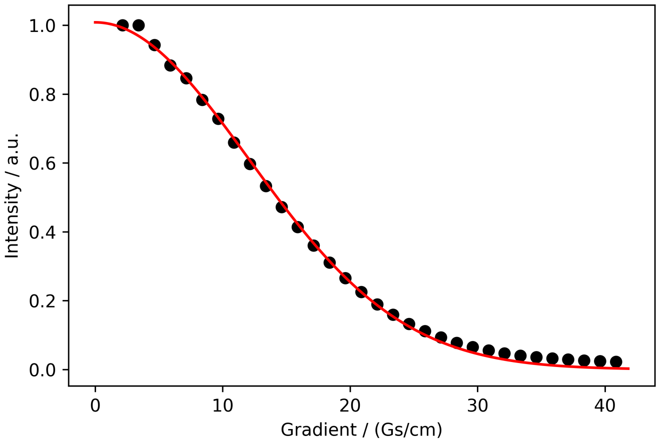

0.01 mM

Anion

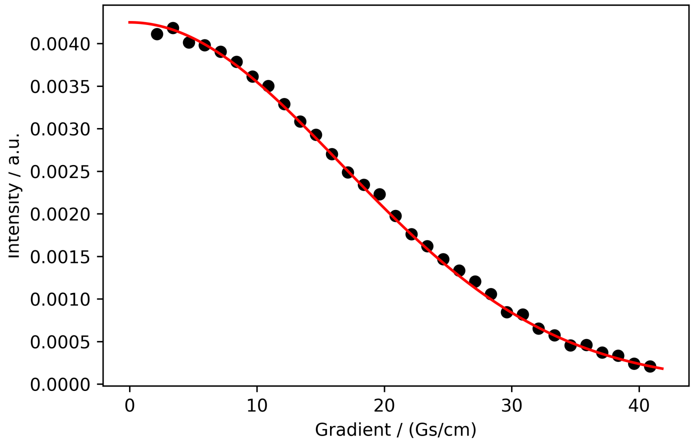

Cation

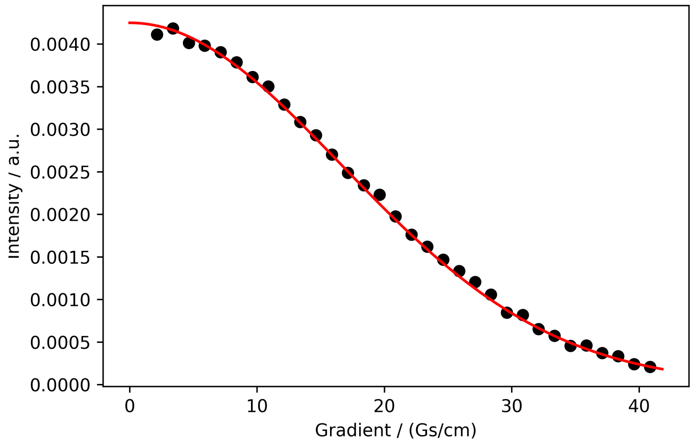

TMS

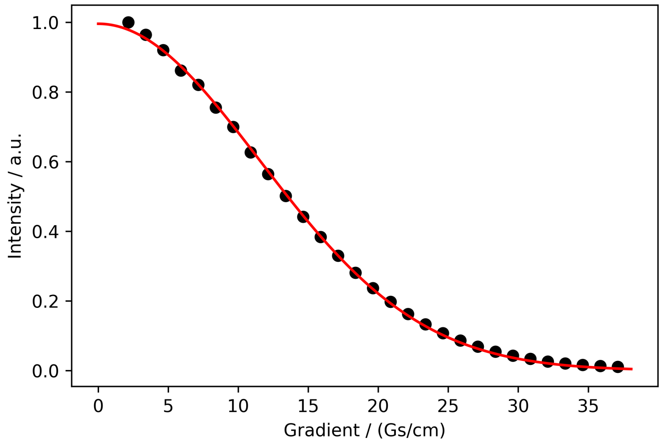

0.05 mM

Anion

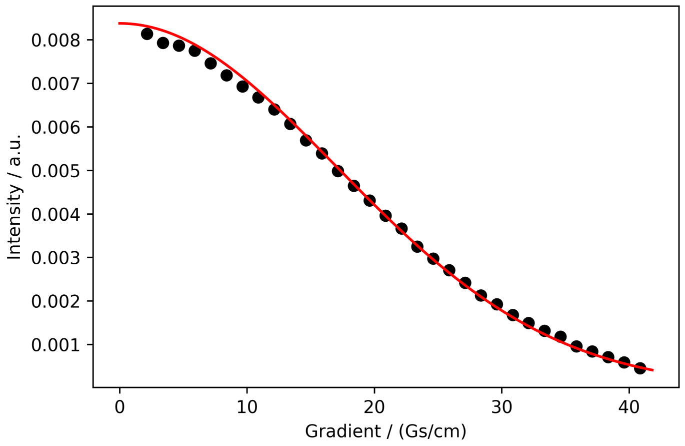

Cation

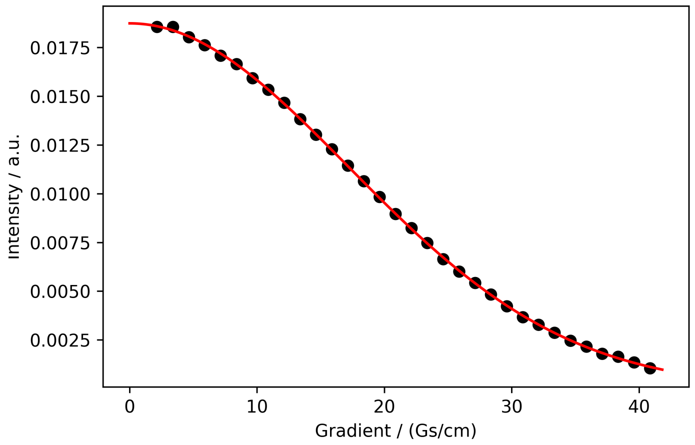

TMS

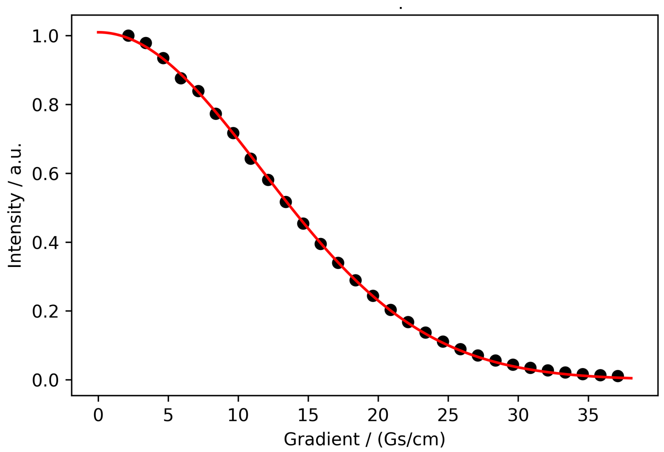

0.1 mM

Anion

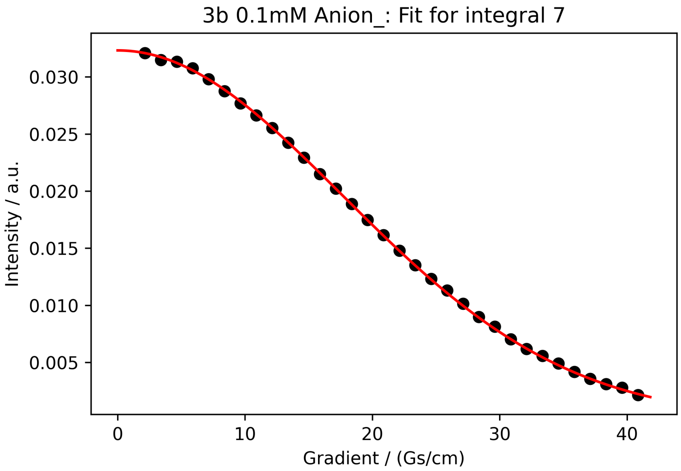

Cation

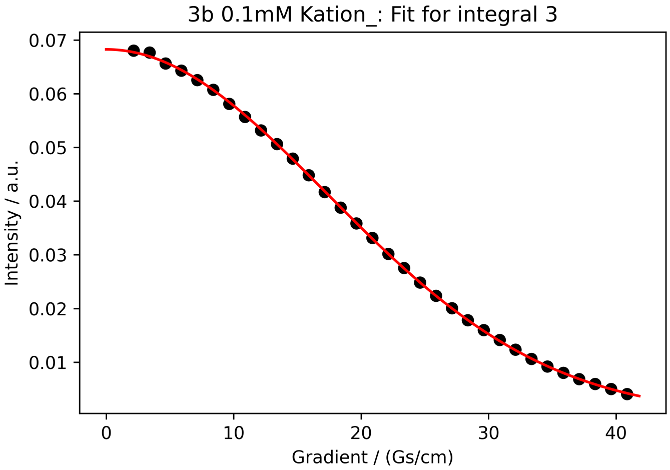

TMS

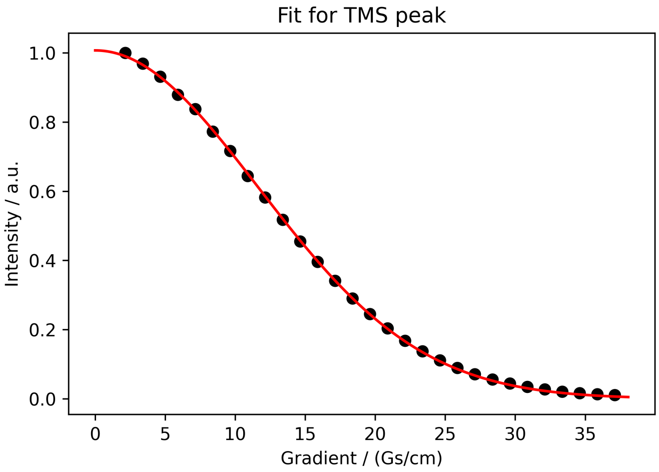

0.2 mM

Anion

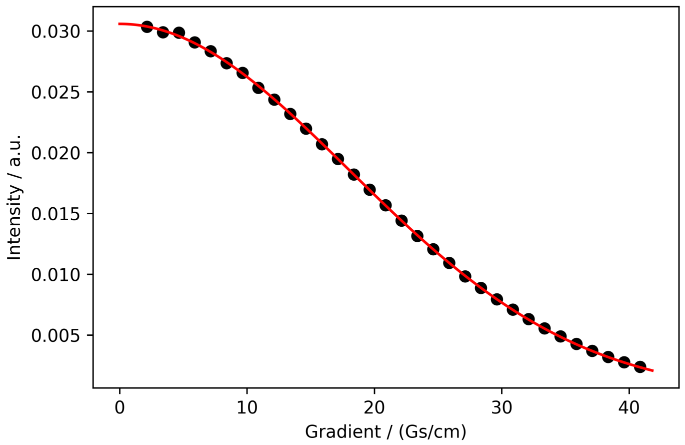

Cation

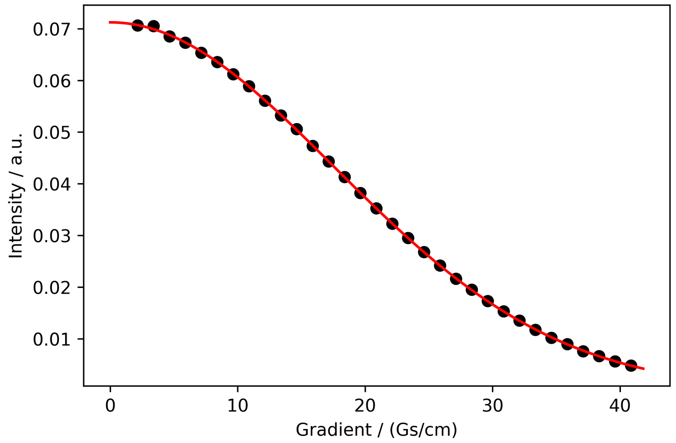

TMS

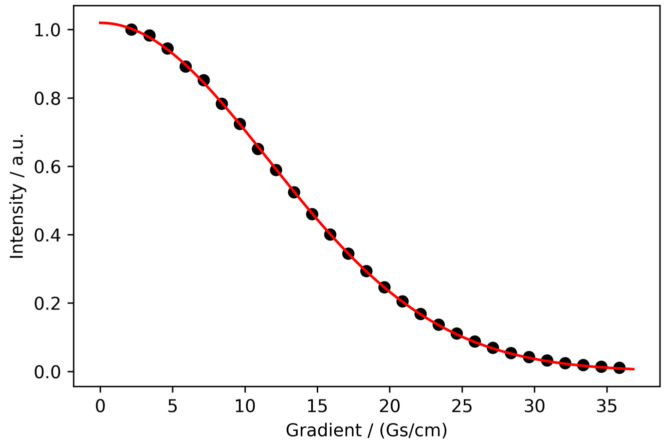

0.4 mM

Anion

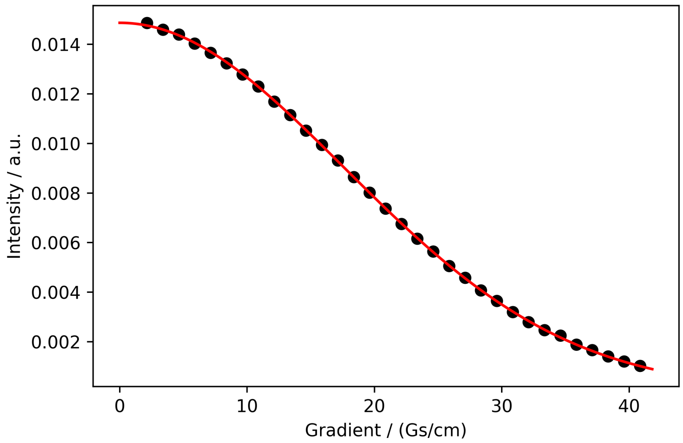

Cation

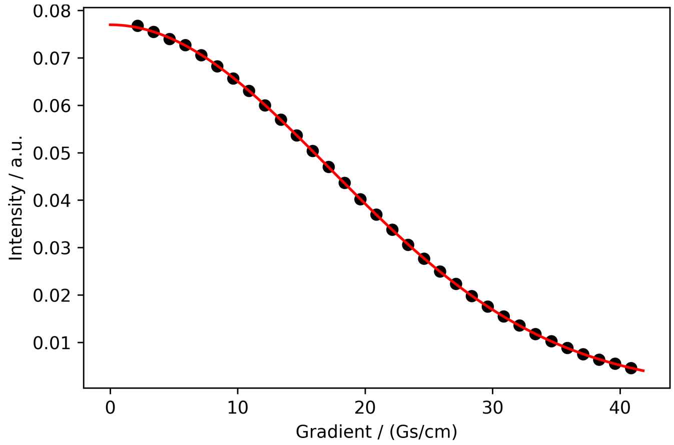

TMS

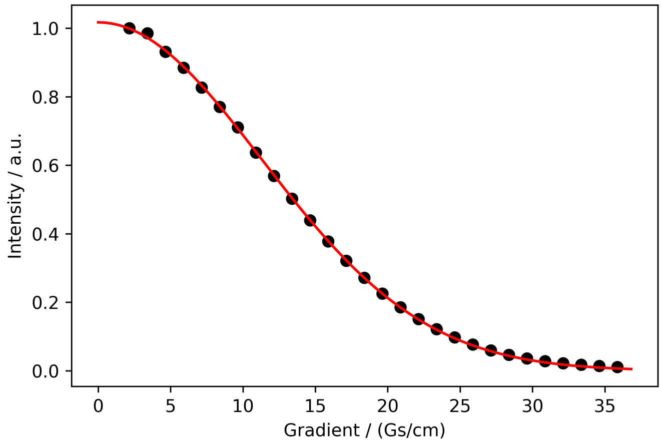

0.6 mM

Anion

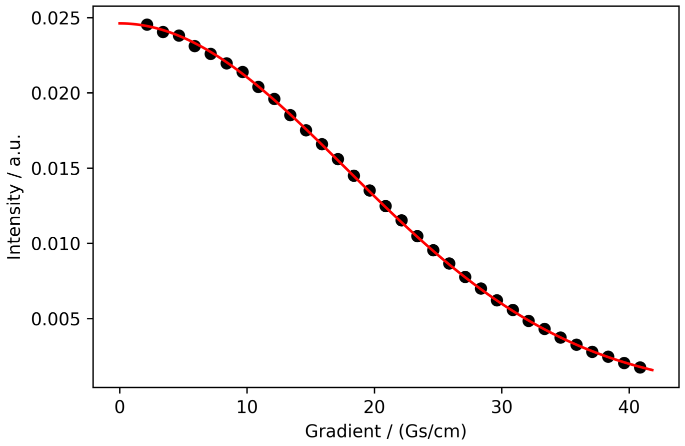

Cation

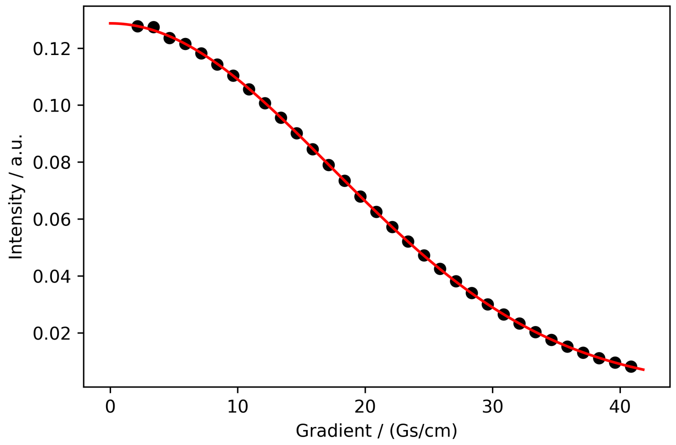

TMS

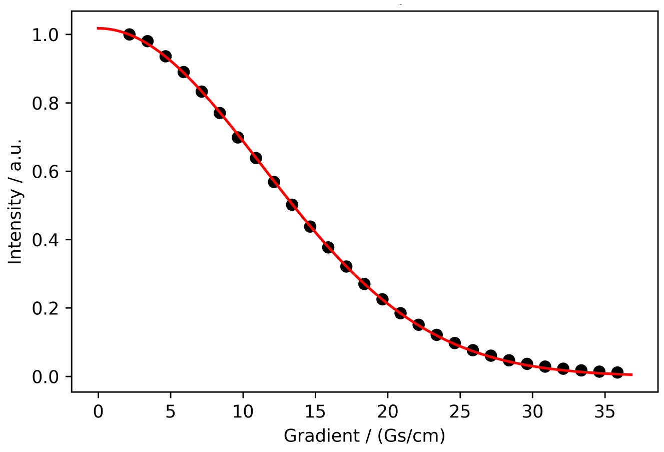

0.8 mM

Anion

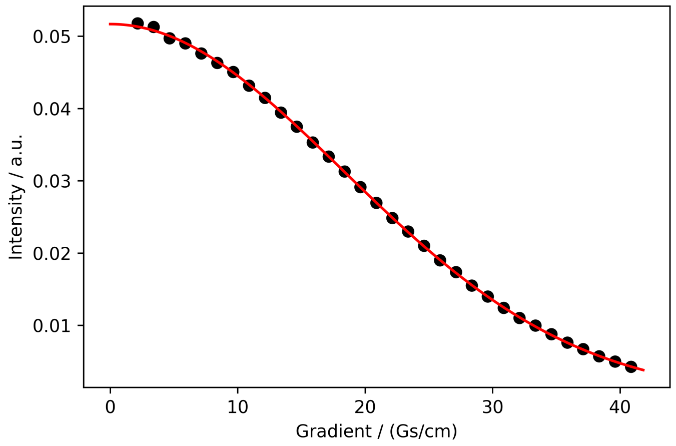

Cation

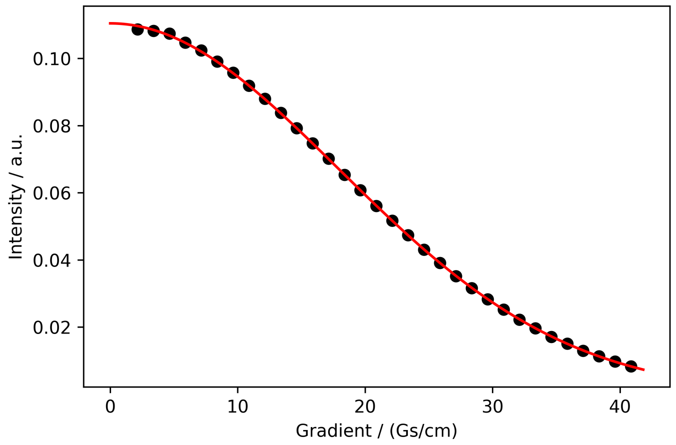

TMS

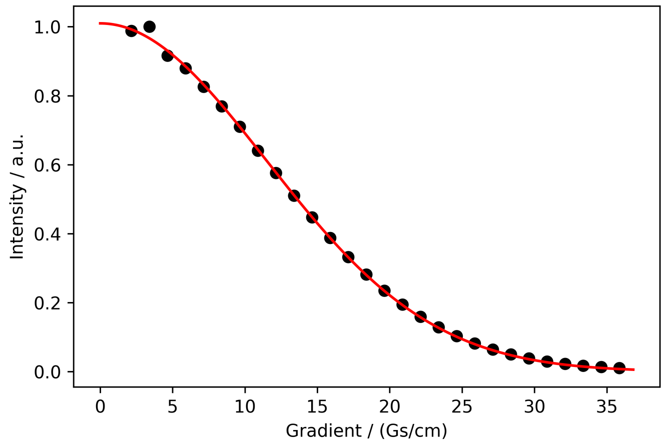

1.0 mM

Anion

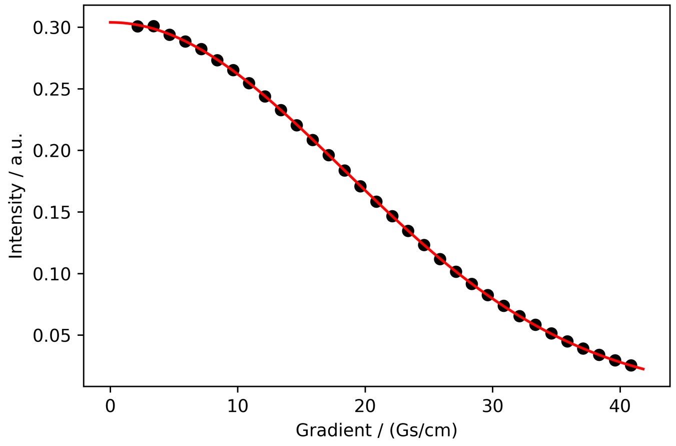

Cation

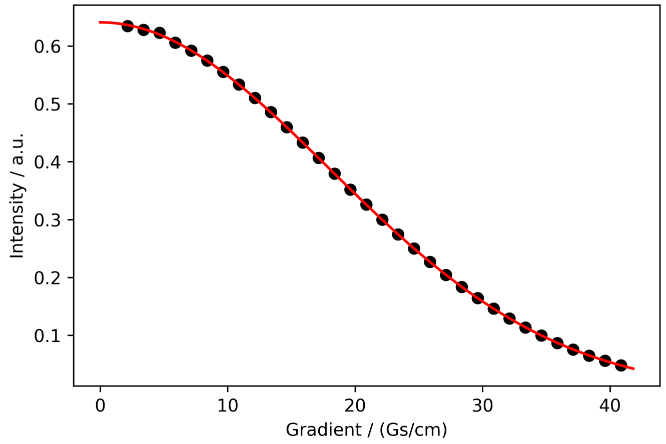

TMS

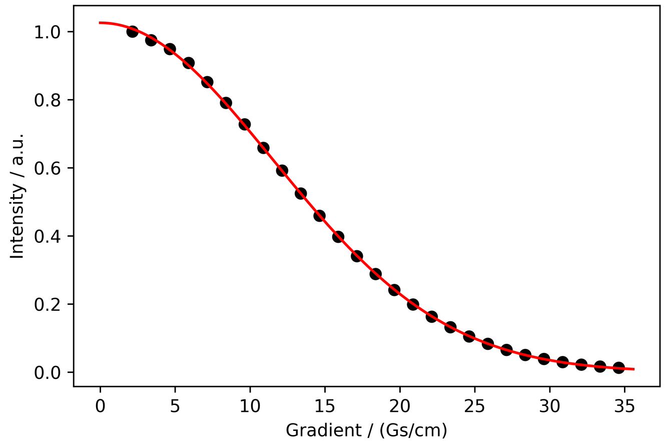

11. Computational Study

General Information

All substrate structures were optimized with the B3LYP-D3 hybrid functional[27–29] with the 6-31+G(d) basis set.[30] Solvent effects for dichloromethane and acetonitrile have been calculated with the SMD continuum solvation model.[31] This combination has worked well in previous studies of charge-separated intermediates.[32–35] Frequency and single point calculations were performed at the same level of theory. Thermochemical correction to 198.15 K has been applied to all found minima from unscaled vibrational frequencies obtained at the same level of theory. To lessen the impact of low-lying frequencies of large systems on entropy and enthalpy in an unpredictable manner, a free-rotor approximation for entropy as proposed by Grimme[36] and a quasi-harmonic treatment with a cutoff value of 100 cm^{-1} using Goodvibes[37] was applied. Free energies in solution have been corrected to the reference state of 1.0 mol L^{-1} at 298.15 K by adding 7.925 kJ mol^{-1} to the free energies ($G_{298,qh,corr}$). All reported calculations were done with Gaussian 16, Revision A.03[38] and B.01[39].

The conformational search was performed with Maestro[40]. For ion pair systems, a set of 150 starting points was obtained by using the stochastic kick procedure invented by Saunders[41] and further developed by Sakic.[42] At a time, a set of 50 starting points was generated by a combination of the best and the second-best conformer of the prior separately optimized anion and cation. The last 50 starting points were obtained from the combination of the best anion conformer with the third best cation conformer. The following kick settings were used: Distance parameter: 3 Å, Minimal Distance 1.5 Å, Number of Fragments: 1, Number of Files: 50.

Starting points for the conformers of the ion pair systems were obtained from Julian Helberg. Conformers for the new systems with tetraphenyl phosphonium as cation (**3a**) were obtained by manual modification of the corresponding triphenyl methyl phosphonium containing systems, initially calculated by Helberg[43], whereby the methyl group was replaced by a phenyl group.

Starting points for the conformers of the sandwich cation and sandwich anion were obtained by sorting the optimized structures of the respective ion pair according to the total energy. The best six conformers were chosen and in combination with the best cation **a** conformer the stochastic kick procedure invented by Saunders[41] and further developed by Sakic[42] was applied to generate 60 new substrate structures in total. The following kick settings were used: Distance parameter: 3 Å, Minimal Distance 1.5 Å, Number of Fragments: 1, Number of Files: 10.

The goal in calculating the respective sandwich conformers was to obtain the volumes based on the van der Waals cavities employed in the SMD continuum solvation model at the SMD(DCM)/B3LYP-D3/6-31+G(d) level of theory for the calculation of the volumes of ions based on the simulated concentrations in each respective conductivity model to compare the DOSY results with the results of the numerical simulations. Due to the heavy nature of the calculated systems (between 81 – 119 atoms) the less costly r^2SCAN-3c developed by Grimme[44] with Orca 5.0.3[45] was used for pre-optimizations, followed by single point calculations at the SMD(DCM)/B3LYP-D3/6-31+G(d) level of theory with Gaussian 16, Revision C.01[46].

11.1 System 3a – Computational Information

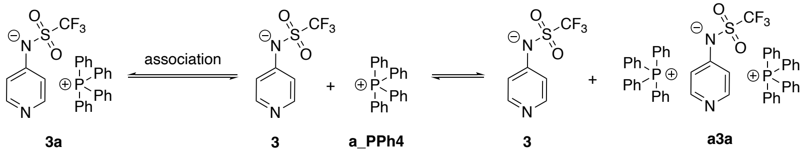

Figure S30. Ion Pair Formation of **3a** and the single ions.

The Boltzmann-averaged free reaction energy of the ion pairing of **3a** amounts to $\Delta G_{qh,298,corr}$ = +1.6 kJ mol^{-1} in DCM solution. Focusing only on the best conformers of the reactants and product the free reaction energy of the ion pairing of **3a** changes to $\Delta G_{qh,298,corr}$ = +1.1 kJ mol^{-1} in DCM solution. This result is mainly due to solvation effects since the gas phase free energy of the ion pairing amounts to $\Delta G_{qh,298}$ = -218.5 kJ mol^{-1}. In addition, we note that the contribution of the D3-dispersion correction amounts to ΔE_{disp} = -57.7 kJ mol^{-1} for the free energy in the DCM solution and in the gas phase. The blue marked cells show the Boltzmann-averaged values.

According to $\Delta G_{qh,298,corr} = -RT \ln K$ and assuming R = 8.314 J K^{-1} mol^{-1} and T = 298.15 K, the respective equilibrium constant amounts to *K*(**3a**, DCM) = 0.63 for the best conformer of **3a** and to *K*(**3a**, DCM) = 0.52 for the Boltzmann-averaged free reaction energy. In this case the equilibrium constant K corresponds to the concentration of the reactants and products in the following way: K = [**3a**]/[**3**] [**a_PPh4**] = [**3a**]/[**3**]^2.

Table S30. Energies for all systems shown in Figure S30.

System	E_{tot} SMD(DCM)/ B3LYP-D3/ 6-31+G(d)	H_{298} SMD(DCM)/ B3LYP-D3/ 6-31+G(d)	$G_{qh,298}$ SMD(DCM)/ B3LYP-D3/ 6-31+G(d)	$G_{qh,298,corr}$ SMD(DCM)/ B3LYP-D3/ 6-31+G(d)	Cavity Volume (Å^3)	Relative Population Parameter based on $G_{qh,298}$
**3**						
an3_001	**-1188.8384606**	**-1188.7186926**	**-1188.769809**	**-1188.766790**	**215**	0.91
an3_002	-1188.8357192	-1188.7160622	-1188.7673382	-1188.7643197	214	0.09
			**-1188.7693051**	**-1188.7662866**	**215**	
**a_PPh4**						
PPh4_003a	-1267.9099858	**-1267.5207058**	**-1267.5885298**	**-1267.5855113**	**360**	0.46
PPh4_001	-1267.9100736	-1267.5203936	-1267.5881566	-1267.5851381	364	0.31
PPh4_002f	**-1267.9100864**	-1267.5202704	-1267.5878314	-1267.5848129	362	0.22
			**-1267.5882580**	**-1267.5852395**	**362**	
**a3a**						
sw_a3a_010	**-3724.703838**	**-3723.800207**	**-3723.937041**	**-3723.934022**	**923**	0.53
sw_a3a_005	-3724.703051	-3723.799441	-3723.936260	-3723.933242	924	0.23
sw_a3a_org_015	-3724.702257	-3723.798129	-3723.933960	-3723.930942	928	0.02
sw_a3a_025a	-3724.701993	-3723.797917	-3723.934093	-3723.931075	930	0.02
sw_a3a_org_014	-3724.701889	-3723.798305	-3723.934938	-3723.931919	927	0.06
sw_a3a_024	-3724.701649	-3723.797640	-3723.934056	-3723.931037	929	0.02

sw_a3a_org_040	-3724.701627	-3723.797598	-3723.933697	-3723.930679	927	0.02
sw_a3a_014a	-3724.701442	-3723.798070	-3723.935298	-3723.932279	928	0.08
sw_a3a_org_012	-3724.700803	-3723.796599	-3723.933000	-3723.929982	927	0.01
sw_a3a_org_026a	-3724.699820	-3723.796143	-3723.932959	-3723.929941	924	0.01
sw_a3a_org_010	-3724.699491	-3723.795677	-3723.930122	-3723.927104	929	0.00
sw_a3a_021a	-3724.699216	-3723.795440	-3723.931151	-3723.928132	927	0.00
sw_a3a_016	-3724.698871	-3723.795018	-3723.931547	-3723.928529	930	0.00
sw_a3a_001	-3724.698495	-3723.794062	-3723.930329	-3723.927311	928	0.00
sw_a3a_org_027	-3724.697508	-3723.793778	-3723.930638	-3723.927619	928	0.00
sw_a3a_org_009a	-3724.697404	-3723.793943	-3723.931035	-3723.928016	925	0.00
sw_a3a_011	-3724.696265	-3723.793325	-3723.928885	-3723.925866	928	0.00
sw_a3a_007	-3724.696019	-3723.793061	-3723.930496	-3723.927478	928	0.00
sw_a3a_org_016	-3724.696018	-3723.792326	-3723.929178	-3723.926160	930	0.00
			**-3723.936250**	**-3723.933231**	**925**	
**3a**[b]						
cat3a_008	-2456.770628	**-2456.259420**	**-2456.354582**	**-2456.351563**	**570**	0.09
cat3a_002	-2456.770669	-2456.259399	-2456.354514	-2456.351496	571	0.08
cat3a_018	**-2456.770818**	-2456.259384	-2456.354496	-2456.351478	570	0.08
cat3a_029	-2456.770644	-2456.259350	-2456.354385	-2456.351367	571	0.07
cat3a_033	-2456.770628	-2456.259315	-2456.354345	-2456.351327	571	0.07
cat3a_036	-2456.770661	-2456.259254	-2456.354273	-2456.351255	570	0.06
cat3a_023	-2456.770337	-2456.258979	-2456.354117	-2456.351099	571	0.05
cat3a_003p	-2456.770003	-2456.258866	-2456.354031	-2456.351012	571	0.05
cat3a_003m	-2456.770027	-2456.258808	-2456.353985	-2456.350967	570	0.05
cat3a_035	-2456.769988	-2456.258756	-2456.353923	-2456.350905	570	0.04
cat3a_013	-2456.769688	-2456.258611	-2456.353788	-2456.350770	570	0.04
cat3a_010	-2456.769703	-2456.258506	-2456.353635	-2456.350617	570	0.03
cat3a_016	-2456.769943	-2456.258424	-2456.353559	-2456.350540	571	0.03
cat3a_005	-2456.769461	-2456.258282	-2456.353405	-2456.350387	566	0.02
cat3a_004a	-2456.769364	-2456.258138	-2456.353293	-2456.350275	570	0.02
cat3a_021a	-2456.769198	-2456.258025	-2456.353238	-2456.350219	571	0.03
cat3a_026b	-2456.770013	-2456.258268	-2456.353138	-2456.350119	572	0.02
cat3a_038	-2456.769095	-2456.257968	-2456.353103	-2456.350084	569	0.02
cat3a_027a	-2456.769251	-2456.257914	-2456.353035	-2456.350016	572	0.02
cat3a_006	-2456.769635	-2456.258078	-2456.352947	-2456.349928	569	0.02
**all**			**-2456.353922**	**-2456.350904**	**570**	
ΔE	-58.47	-52.57	+9.06	+1.14		
**all**			+9.56	+1.63		

Table S31. Energies of the best conformer for all systems shown in Figure S30 at different levels of theory.

System	E_{tot}[a] B3LYP-D3/ 6-31+G(d)	$G_{qh,298}$[a] B3LYP-D3/ 6-31+G(d)	E_{tot}[a] B3LYP/ 6-31+G(d)	$G_{qh,298}$[a] B3LYP/ 6-31+G(d)	E_{tot}[a] SMD(DCM)/ B3LYP/ 6-31+G(d)	$G_{qh,298,corr}$[a] SMD(DCM)/ B3LYP/ 6-31+G(d)
**3**						
an3_001	**-1188.770301**	**-1188.701344**	**-1188.753230**	**-1188.684273**	**-1188.821390**	**-1188.7494140**
**a_PPh4**						
PPh4_003a	**-1267.828115**	**-1267.506659**	**-1267.778739**	**-1267.457283**	**-1267.860609**	**-1267.5361348**
**a3a**						
sw_a3a_010	**-3724.586374**	**-3723.819577**	**-3724.415638**	**-3723.648841**	**-3724.533101**	**-3723.7632859**
**cat3a**						
cat3a_008	**-2456.707283**	**-2456.291237**	**-2456.618859**	**-2456.202813**	**-2456.682204**	**-2456.2631398**
ΔE(3a, 3, a)	-285.83	-218.53	-228.13	-160.83	-0.54	+58.83

[a] using geometries optimized at SMD(DCM)/B3LYP-D3/6-31+G(d) level.

Table S32. Charge of system **3a** and selected structural data of the best conformer and the crystal structure of system **3a**.

System	Charge SMD(DCM)/ B3LYP-D3/ 6-31+G(d)	Charge B3LYP-D3/ 6-31+G(d)	D_1 (pm) N_{py} - H_{cat}	D_2 (pm) N_{amid} - H_{cat}	D_3 (pm) O_{anion} - H_{cation}
**3**					
an3_001	-1.0000	-1.0000			
**a_PPh4**					
PPh4_003a	+1.0000	+1.0000			
**cat3a**					
cat3a_008	-0.9932 (Anion)<br>+0.9932 (Cation)	-0.9445 (Anion)<br>+0.9445 (Cation)	268.43 (α-H)	332.43 (β-H)	242.49 (α-H)

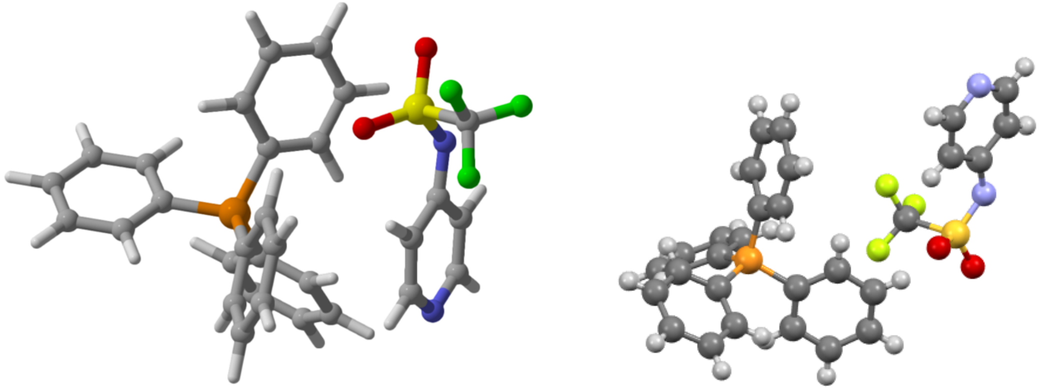

Figure S31. left: energetically best conformer of ion pair cat**3a** (SMD(DCM)/ B3LYP-D3/6-31+G(d) level of theory). Right: crystal structure of one ion pair **3a**.

Table S33. Molecular volume (in Å^3) of cation PPh_4^+ cation (**a**) calculated with different theoretical approaches[d].

System	SMD(DCM)/ B3LYP-D3/ 6-31+G(d) (SMD volume)[a]	SMD(DCM)/ M06-2X/ cc-pVDZ (SMD volume)[a]	SMD(DCM)/ M06-2X/ cc-pVDZ (SMD volume)[b]	SMD(DCM)/ B3LYP-D3/ 6-31+G(d) (MC volume)[a]	M06-2X/ cc-pVDZ (MC volume)[a]	M06-2X/ cc-pVDZ (MC volume)[c]
**Gaussian 16, Rev. C.02**						
PPh4_002f	**362.0**	362.0	360.5	440.3 (2974.9 Bohr^3)	406.5 (2746.7 Bohr^3)	444.5 (3003.1 Bohr^3)
**ORCA Rev. 5.0.2**						
PPh4_002f			356.2 (2406.9 Bohr^3)			

[a] using geometries optimized at SMD(DCM)/B3LYP-D3/6-31+G(d) level. [b] using geometries optimized at SMD(DCM)/M06-2X/cc-pVDZ level. [c] using geometries optimized at M06-2X/cc-pVDZ level. [d] A conversion factor of vol(Å^3) = 0.148 vol(Bohr^3) has been used.

Table S34. Molecular volume (in Å^3) of pyridinamide anion **3** calculated with different theoretical approaches[c].

System	SMD(DCM)/ B3LYP-D3/ 6-31+G(d) (SMD volume)[a]	SMD(DCM)/ M06-2X/ cc-pVDZ (SMD volume)[a]	SMD(DCM)/ M06-2X/ cc-pVDZ (SMD volume)[b]	SMD(DCM)/ B3LYP-D3/ 6-31+G(d) (MC volume)[a]	M06-2X/ cc-pVDZ (MC volume)[a]	M06-2X/ cc-pVDZ (MC volume)
**Gaussian 16, Rev. C.02**						
an1_001<br>an1_003<br>an1_004<br>an1_005	**214.6**	214.6	212.7			
an1_002	214.2					
**ORCA Rev. 5.0.2**						
an1_006			211.8 (Å^3) (1431.3 Bohr^3)			

[a] using geometries optimized at SMD(DCM)/B3LYP-D3/6-31+G(d) level. [b] using geometries optimized at SMD(DCM)/M06-2X/cc-pVDZ level. [c] A conversion factor of vol(Å^3) = 0.148 vol(Bohr^3) has been used.

11.2 System 4a – Computational Information

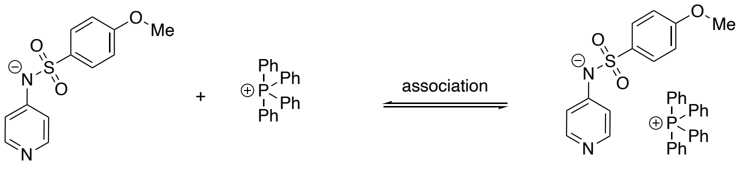

Figure S32. Ion Pair Formation of **4a** and the single ions.

The Boltzmann-averaged free reaction energy of the ion pairing of **4a** amounts to $\Delta G_{qh,298,corr}$ = -4.3 kJ mol^{-1} in DCM solution. Focusing only on the best conformers of the reactants and product the free reaction energy of the ion pairing of **4a** changes to $\Delta G_{qh,298,corr}$ = -6.7 kJ mol^{-1} in DCM solution. This result is mainly due to solvation effects since the gas phase free energy of the ion pairing amounts to $\Delta G_{qh,298}$ = -227.3 kJ mol^{-1}. In addition, we note that the contribution of the D3-dispersion correction amounts to ΔE_{disp} = +136.6 kJ mol^{-1} for the free energy in the DCM solution and in the gas phase. The blue marked cells show the Boltzmann-averaged values.

According to $\Delta G_{qh,298,corr}$ = -RT lnK and assuming R = 8.314 J K^{-1} mol^{-1} and T = 298.15 K, the respective equilibrium constant amounts to *K*(**4a**, DCM) = +15.0 for the best conformer of **4a** and to *K*(**4a**, DCM) = +5.6 for the Boltzmann-averaged free reaction energy. In this case the equilibrium constant K corresponds to the concentration of the reactants and products in the following way: K = [**4a**]/[**4**] [**a_PPh4**] = [**4a**]/[**4**]^2.

Table S35. Energies for all systems shown in Figure S32.

System	E_{tot} SMD(DCM)/ B3LYP-D3/ 6-31+G(d)	H_{298} SMD(DCM)/ B3LYP-D3/ 6-31+G(d)	$G_{qh,298}$ SMD(DCM)/ B3LYP-D3/ 6-31+G(d)	$G_{qh,298,corr}$ SMD(DCM)/ B3LYP-D3/ 6-31+G(d)	Cavity Volume (Å^3)	Relative Population Parameter based on $G_{qh,298}$
**4**						
an4_003	**-1197.397984**	**-1197.165232**	**-1197.223213**	**-1197.220195**	**285**	0.50
an4_002	-1197.397861	-1197.165102	-1197.223086	-1197.220068	285	0.44
an4_001	-1197.395672	-1197.163018	-1197.221274	-1197.218256	285	0.06
			**-1197.223034**	**-1197.220015**	**285**	
**a_PPh4**						
PPh4_003a	-1267.9099858	**-1267.5207058**	**-1267.5885298**	**-1267.5855113**	**360**	0.46
PPh4_001	-1267.9100736	-1267.5203936	-1267.5881566	-1267.5851381	364	0.31
PPh4_002f	**-1267.9100864**	-1267.5202704	-1267.5878314	-1267.5848129	362	0.22
			**-1267.588258**	**-1267.585239**	**362**	
**cat4a**[b]						
cat4a_050_dcm	**-2465.334938**	**-2464.710187**	**-2464.811281**	**-2464.808262**	**642**	0.21
cat4a_030_dcm	-2465.334896	-2464.710055	-2464.811095	-2464.808077	642	0.17
cat4a_025_dcm	-2465.333350	-2464.708548	-2464.809594	-2464.806575	646	0.03

cat4a_037_dcm	-2465.333341	-2464.708434	-2464.809435	-2464.806416	640	0.03
cat4a_108_dcm	-2465.333336	-2464.708800	-2464.810174	-2464.807155	642	0.06
cat4a_026_dcm	-2465.333194	-2464.708774	-2464.810286	-2464.807268	640	0.07
cat4a_055_dcm	-2465.332897	-2464.708336	-2464.809530	-2464.806512	640	0.03
cat4a_047_dcm	-2465.332521	-2464.708173	-2464.809641	-2464.806623	643	0.04
cat4a_018_dcm	-2465.332388	-2464.708158	-2464.809636	-2464.806617	641	0.04
cat4a_105_dcm	-2465.332334	-2464.708102	-2464.809786	-2464.806767	642	0.04
cat4a_100p_dcm	-2465.332005	-2464.707868	-2464.809694	-2464.806676	641	0.04
cat4a_032_dcm	-2465.331988	-2464.707080	-2464.808050	-2464.805031	638	0.01
cat4a_063_dcm	-2465.331873	-2464.707386	-2464.808686	-2464.805668	637	0.01
cat4a_042_dcm	-2465.331767	-2464.707018	-2464.808010	-2464.804991	636	0.01
cat4a_002_dcm	-2465.331657	-2464.707287	-2464.808608	-2464.805589	636	0.01
cat4a_089_dcm	-2465.331606	-2464.708112	-2464.808197	-2464.805178	640	0.01
cat4a_054_dcm	-2465.331512	-2464.707211	-2464.808728	-2464.805709	638	0.01
cat4a_004_dcm	-2465.331365	-2464.706715	-2464.807939	-2464.804921	636	0.01
cat4a_024_dcm	-2465.331340	-2464.706870	-2464.808260	-2464.805242	640	0.01
cat4a_051_dcm	-2465.331046	-2464.706547	-2464.807769	-2464.804751	641	0.01
**all**			**-2464.809896**	**-2464.806877**	**641**	
ΔE	-70.80	-63.67	+1.21	-6.71	-299.34	-227.32
**all**			+3.67	-4.26		

[a] using geometries optimized at SMD(DCM)/B3LYP-D3/6-31+G(d) level. [b] best 20 conformers according to E_{tot} at SMD(DCM)/B3LYP-D3/6-31+G(d) level of theory.

Table S36. Energies of the best conformer for all systems shown in Figure S32 at different levels of theory.

System	E_{tot}[a] B3LYP-D3/ 6-31+G(d)	$G_{qh,298}$[a] B3LYP-D3/ 6-31+G(d)	E_{tot}[a] B3LYP/ 6-31+G(d)	$G_{qh,298}$[a] B3LYP/ 6-31+G(d)	E_{tot}[a] SMD(DCM)/ B3LYP/ 6-31+G(d)	$G_{qh,298,corr}$[a] SMD(DCM)/ B3LYP/ 6-31+G(d)
**4**						
an4_003	**-1197.312402**	**-1197.137631**	**-1197.368222**	**-1197.193451**	**-1197.282640**	**-1197.104850**
**a_PPh4**						
PPh4_003a	**-1267.828115**	**-1267.506659**	**-1267.778739**	**-1267.457283**	**-1267.860609**	**-1267.5361348**
**cat4a**						
cat4a_050	**-2465.254530**	**-2464.730873**	**-2465.141852**	**-2464.618195**	**-2465.222260**	**-2464.6955847**
ΔE(4a, 4, a)	-299.34	-227.32	+13.41	+85.43	-207.44	-143.35

[a] using geometries optimized at SMD(DCM)/B3LYP-D3/6-31+G(d) level.

Table S37. Charge of system **4a** and selected structural data of the best conformer and the crystal structure of system **4a**.

**System**	**Charge SMD(DCM)/ B3LYP-D3/ 6-31+G(d)**	**Charge B3LYP-D3/ 6-31+G(d)**	**D_1 (pm) N_{py} - H_{cat}**	**D_2 (pm) N_{amid} - H_{cat}**	**D_3 (pm) O_{anion} - H_{cation}**
**4**					
an4_003	-1.0000	-1.0000			
**a_PPh4**					
PPh4_003a	+1.0000	+1.0000			
**4a**					
cat4a_050	-0.7949 (Anion)<br>+0.7949 (Cation)	-0.6926 (Anion)<br>+0.6926 (Cation)	292.50 (α-H)	270.23 (α-H)	280.83 (β-H)

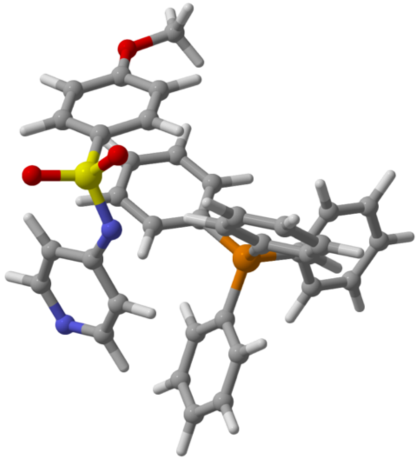

Figure S33. Structure of conformer cat4a_050 calculated at SMD(DCM)/ B3LYP-D3/6-31+G(d) level of theory.

11.3 System PPh_4BF_4 (6) – Computational Information

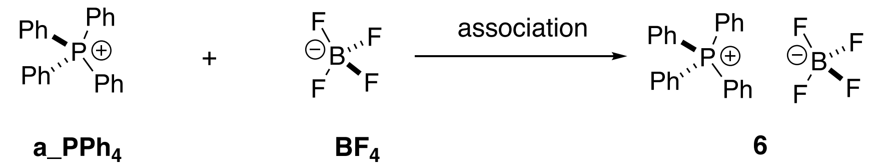

Figure S34. Ion Pair Formation of **6** and the single ions.

The Boltzmann-averaged free reaction energy of the ion pairing of **6** amounts to $\Delta G_{qh,298,corr}$ = +11.1 kJ mol^{-1} in DCM solution. Focusing only on the best conformers of the reactants and product the free reaction energy of the ion pairing of **6** changes to $\Delta G_{qh,298,corr}$ = +10.6 kJ mol^{-1} in DCM solution. The blue marked cells show the Boltzmann-averaged values.

According to $\Delta G_{qh,298,corr} = -RT \ln K$ and assuming R = 8.314 J K^{-1} mol^{-1} and T = 298.15 K, the respective equilibrium constant amounts to *K*(**6**, DCM) = 0.014 for the best conformer of **6** and to *K*(**6**, DCM) = 0.011 for the Boltzmann-averaged free reaction energy. In this case the equilibrium constant *K* corresponds to the concentration of the reactants and products in the following way: *K* = [**6**]/[BF_4] [**a_PPh4**] = [**6**]/[BF_4]^2.

Table S38. Energies for all systems shown in Figure S34.

System	E_{tot} SMD(DCM)/ B3LYP-D3/ 6-31+G(d)	H_{298} SMD(DCM)/ B3LYP-D3/ 6-31+G(d)	$G_{qh,298}$ SMD(DCM)/ B3LYP-D3/ 6-31+G(d)	$G_{qh,298,corr}$ SMD(DCM)/ B3LYP-D3/ 6-31+G(d)	Cavity Volume (Å^3)	Relative Population Parameter based on $G_{qh,298}$
**BF_4**						
borate_001_dcm	**-424.6525330**	**-424.6336440**	**-424.6644660**	**-424.6614475**	**69.7**	1.00
			**-424.6644660**	**-424.6614475**	**69.7**	
**a_PPh4**						
PPh4_003a	-1267.9099858	**-1267.5207058**	**-1267.5885298**	**-1267.5855113**	**360**	0.46
PPh4_001	-1267.9100736	-1267.5203936	-1267.5881566	-1267.5851381	364	0.31
PPh4_002f	**-1267.9100864**	-1267.5202704	-1267.5878314	-1267.5848129	362	0.22
			**-1267.5882580**	**-1267.5852395**	**362**	
**6**[a]						
add6_034_dcm	**-1692.5752397**	**-1692.1650487**	**-1692.2459257**	**-1692.2429072**	**430**	0.06
add6_146_dcm	-1692.5752471	-1692.1650451	-1692.2459061	-1692.2428876	430	0.05
add6_012_dcm	-1692.5752413	-1692.1650293	-1692.2459003	-1692.2428818	430	0.05
add6_089_dcm	-1692.5752306	-1692.1649816	-1692.2458436	-1692.2428251	430	0.05
add6_140_dcm	-1692.5752321	-1692.1649631	-1692.2458121	-1692.2427936	430	0.05
add6_006_dcm	-1692.5752514	-1692.1649734	-1692.2458104	-1692.2427919	430	0.05
add6_134_dcm	-1692.5750318	-1692.1648098	-1692.2457278	-1692.2427093	429	0.05

add6_042_dcm	-1692.5752256	-1692.1648236	-1692.2456156	-1692.2425971	430	0.04
add6_126_dcm	-1692.5750552	-1692.1646782	-1692.2455882	-1692.2425697	430	0.04
add6_051_dcm	-1692.5751517	-1692.1647177	-1692.2455337	-1692.2425152	430	0.04
add6_059_dcm	-1692.5750835	-1692.1646505	-1692.2455245	-1692.2425060	429	0.04
add6_145_dcm	-1692.5749650	-1692.1646460	-1692.2455130	-1692.2424945	430	0.04
add6_055_dcm	-1692.5749721	-1692.1646461	-1692.2455101	-1692.2424916	429	0.04
add6_056_dcm	-1692.5750124	-1692.1646304	-1692.2454694	-1692.2424509	430	0.03
add6_111_dcm	-1692.5749883	-1692.1646063	-1692.2454543	-1692.2424358	430	0.03
add6_133_dcm	-1692.5748036	-1692.1645456	-1692.2453376	-1692.2423191	430	0.03
add6_076_dcm	-1692.5746177	-1692.1644227	-1692.2453177	-1692.2422992	430	0.03
add6_136_dcm	-1692.5748232	-1692.1644452	-1692.2452622	-1692.2422437	430	0.03
add6_114_dcm	-1692.5747759	-1692.1643989	-1692.2452389	-1692.2422204	429	0.03
add6_116_dcm	-1692.5745338	-1692.1642658	-1692.2451478	-1692.2421293	429	0.02
**all**			**-1692.2454665**	**-1692.2424480**	**430**	
ΔE	-33.2	-28.1	+18.6	+10.6		
**all**			**+19.1**	**+11.1**		

[a] best 20 conformers according to $G_{qh,298}$ at SMD(DCM)/B3LYP-D3/6-31+G(d) level of theory.

11.4 Sandwich Systems – Computational Information

Table S39. Energies for all sandwich systems of ion pair **3a** optimized with SMD(DCM)/r^{2}SCAN-3c level of theory with subsequent single point calculations at SMD(DCM)/B3LYP-D3/6-31+G(d). The energies are sorted according to ΔE_{tot}.

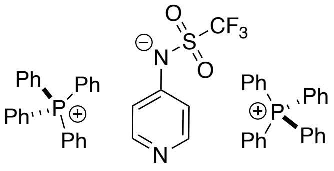 **a3a**				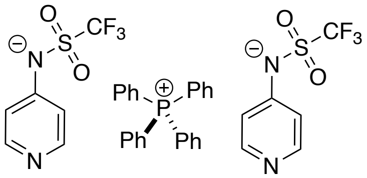 **3a3**			
**System**	**E_{tot} SMD(DCM)/ B3LYP-D3/6-31+G(d)**	**Cavity Volume (Å^3)**	**Relative Population Parameter based on E_{298}**	**System**	**E_{tot} SMD(DCM)/ B3LYP-D3/6-31+G(d)**	**Cavity Volume (Å^3)**	**Relative Population Parameter based on E_{298}**
sw_a3a_new_033b_dcm_sp	**-3724.7011753**	**925**	**0.39**	asw_3a3_058b_dcm_sp	**-3645.6244701**	**781**	**0.23**
sw_a3a_new_030a_dcm_sp	-3724.7008822	923	0.28	asw_3a3_011a_dcm_sp	-3645.6233739	780	0.07
sw_a3a_new_005na_dcm_sp	-3724.6995552	925	0.07	asw_3a3_044a_dcm_sp	-3645.6233403	781	0.07
sw_a3a_new_039a_dcm_sp	-3724.6993743	926	0.06	asw_3a3_032a_dcm_sp	-3645.6231043	783	0.05
sw_a3a_new_015b_dcm_sp	-3724.6989764	926	0.04	asw_3a3_053a_dcm_sp	-3645.6231020	783	0.05
sw_a3a_new_042a_dcm_sp	-3724.6989336	927	0.04	asw_3a3_006_dcm_sp	-3645.6230698	781	0.05
sw_a3a_new_010b_dcm_sp	-3724.6985369	927	0.02	asw_3a3_047a_dcm_sp	-3645.6230374	782	0.05
sw_a3a_new_045c_dcm_sp	-3724.6984005	926	0.02	asw_3a3_039b_dcm_sp	-3645.6229589	782	0.05
sw_a3a_new_014_dcm_sp	-3724.6980441	926	0.01	asw_3a3_045b_dcm_sp	-3645.6228896	780	0.04
sw_a3a_new_047a_dcm_sp	-3724.6976338	926	0.01	asw_3a3_049a_dcm_sp	-3645.6228146	783	0.04
sw_a3a_new_018a_dcm_sp	-3724.6975821	926	0.01	asw_3a3_048b_dcm_sp	-3645.6227627	784	0.04
sw_a3a_new_038a_dcm_sp	-3724.6975740	927	0.01	asw_3a3_057b_dcm_sp	-3645.6226849	781	0.03
sw_a3a_new_020a_dcm_sp	-3724.6971852	926	0.01	asw_3a3_050_dcm_sp	-3645.6223597	781	0.02
sw_a3a_new_004a_dcm_sp	-3724.6970783	926	0.01	asw_3a3_059b_dcm_sp	-3645.6223396	782	0.02
sw_a3a_new_003_dcm_sp	-3724.6968941	928	0.00	asw_3a3_022a_dcm_sp	-3645.6220941	781	0.02
sw_a3a_new_052b_dcm_sp	-3724.6967130	925	0.00	asw_3a3_037_dcm_sp	-3645.6219701	785	0.02
sw_a3a_new_013b_dcm_sp	-3724.6964818	925	0.00	asw_3a3_008a_dcm_sp	-3645.6217232	783	0.01
sw_a3a_new_029a_dcm_sp	-3724.6964377	925	0.00	asw_3a3_051b_dcm_sp	-3645.6217229	785	0.01
sw_a3a_new_040a_dcm_sp	-3724.6963431	924	0.00	asw_3a3_033_dcm_sp	-3645.6216873	780	0.01
sw_a3a_new_055b_dcm_sp	-3724.6963343	928	0.00	asw_3a3_004a_dcm_sp	-3645.6216535	780	0.01
	**-3724.7002363**	**925**			**-3645.6230836**	**782**	

Table S40. Energies for all sandwich systems of ion pair **4a** optimized with SMD(DCM)/r^{2}SCAN-3c level of theory with subsequent single point calculations at SMD(DCM)/B3LYP-D3/6-31+G(d). The energies are sorted according to ΔE_{tot}.

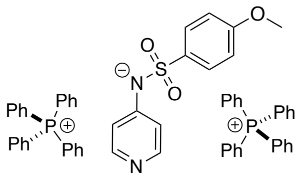 **a4a**				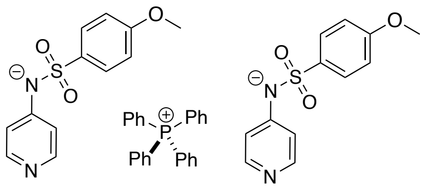 **4a4**			
**System**	**E_{tot} SMD(DCM)/ B3LYP-D3/6-31+G(d)**	**Cavity Volume (Å^3)**	**Relative Population Parameter based on E_{298}**	**System**	**E_{tot} SMD(DCM)/ B3LYP-D3/6-31+G(d)**	**Cavity Volume (Å^3)**	**Relative Population Parameter based on E_{298}**
sw_a4a_018_dcm_sp	**-3733.2653541**	**993**	**0.39**	asw_4a4_005a_dcm_sp	**-3662.7548378**	**921**	**0.53**
sw_a4a_049a_dcm_sp	-3733.2653444	995	0.39	asw_4a4_016_dcm_sp	-3662.7535339	921	0.13
sw_a4a_004b_dcm_sp	-3733.2635672	998	0.06	asw_4a4_011a_dcm_sp	-3662.7534663	920	0.12
sw_a4a_040b_dcm_sp	-3733.2629560	999	0.03	asw_4a4_015a_dcm_sp	-3662.7532492	920	0.10
sw_a4a_022_dcm_sp	-3733.2629292	997	0.03	asw_4a4_006_dcm_sp	-3662.7526111	915	0.05
sw_a4a_017a_dcm_sp	-3733.2628530	997	0.03	asw_4a4_031a_dcm_sp	-3662.7516599	923	0.02
sw_a4a_005a_dcm_sp	-3733.2626049	998	0.02	asw_4a4_024a_dcm_sp	-3662.7515888	923	0.02
sw_a4a_011a_dcm_sp	-3733.2619594	998	0.01	asw_4a4_059n_dcm_sp	-3662.7514401	922	0.01
sw_a4a_008a_dcm_sp	-3733.2613398	998	0.01	asw_4a4_001_dcm_sp	-3662.7495052	924	0.00
sw_a4a_028a_dcm_sp	-3733.2612325	995	0.01	asw_4a4_034_dcm_sp	-3662.7493973	923	0.00
sw_a4a_007_dcm_sp	-3733.2611335	999	0.00	asw_4a4_021a_dcm_sp	-3662.7488765	923	0.00
sw_a4a_039a_dcm_sp	-3733.2608890	999	0.00	asw_4a4_002_dcm_sp	-3662.7486765	922	0.00
sw_a4a_016_dcm_sp	-3733.2606781	998	0.00	asw_4a4_014_dcm_sp	-3662.7485596	922	0.00
sw_a4a_047a_dcm_sp	-3733.2603097	994	0.00	asw_4a4_023a_dcm_sp	-3662.7485027	920	0.00
sw_a4a_038b_dcm_sp	-3733.2602851	997	0.00	asw_4a4_042b_dcm_sp	-3662.7484625	918	0.00
sw_a4a_044_dcm_sp	-3733.2601809	994	0.00	asw_4a4_035a_dcm_sp	-3662.7480696	923	0.00
sw_a4a_037a_dcm_sp	-3733.2601341	995	0.00	asw_4a4_050a_dcm_sp	-3662.7480102	915	0.00
sw_a4a_013a_dcm_sp	-3733.2597333	998	0.00	asw_4a4_057n_dcm_sp	-3662.7479483	923	0.00
sw_a4a_023b_dcm_sp	-3733.2595989	996	0.00	asw_4a4_019b_dcm_sp	-3662.7477695	921	0.00
sw_a4a_015a_dcm_sp	-3733.2593335	998	0.00	asw_4a4_028b_dcm_sp	-3662.7477203	922	0.00
	**-3733.2647588**	**995**			**-3662.7539922**	**920**	

11.5 XYZ-Coordinates of Most Stable Compounds

Anion **3** (an3_001)

C	1.659230	0.571311	0.763808
C	2.953062	1.084068	0.816856
N	3.988020	0.641455	0.080433
C	3.712189	-0.375593	-0.760073
C	2.460090	-0.961272	-0.895098
C	1.371142	-0.494465	-0.121595
N	0.157074	-1.136213	-0.334395
S	-1.176585	-0.801711	0.441860
O	-2.189936	-1.835815	0.149076
O	-1.065523	-0.336514	1.846666
C	-1.908122	0.720493	-0.425945
F	-2.109437	0.485410	-1.738566
F	-3.094301	1.067656	0.117997
F	-1.079318	1.782764	-0.320890
H	0.894109	0.992334	1.404068
H	3.169391	1.905913	1.498174
H	4.544734	-0.740419	-1.359870
H	2.307104	-1.778249	-1.594938

Anion **4** (an4_003)

C	1.934458	-0.927454	0.864929
C	2.462189	-2.200489	1.058228
N	3.373116	-2.790839	0.262312
C	3.779670	-2.052681	-0.794005
C	3.318525	-0.778472	-1.086022
C	2.352139	-0.151490	-0.251882
N	1.934704	1.102967	-0.624199
S	0.781063	1.884140	0.194805
O	0.577233	3.183582	-0.499463
O	1.009994	1.968162	1.668299
C	-0.750912	0.944512	-0.015483
C	-1.472220	0.498372	1.089207
C	-2.655354	-0.229804	0.915413
C	-3.108900	-0.511180	-0.380876
C	-2.375199	-0.061585	-1.494892
C	-1.203856	0.661875	-1.312876
O	-4.242237	-1.211844	-0.666063
C	-5.035021	-1.707004	0.418114

H	1.220549	-0.538986	1.580899
H	2.130452	-2.785130	1.916144
H	4.519133	-2.518897	-1.444372
H	3.688940	-0.247603	-1.959384
H	-1.112590	0.714450	2.090221
H	-3.201670	-0.568444	1.788297
H	-2.740142	-0.291297	-2.492111
H	-0.638189	1.002228	-2.175437
H	-5.409801	-0.886010	1.042123
H	-5.876395	-2.225114	-0.046218
H	-4.465066	-2.413133	1.034848

Anion **BF_4** (borate_001_dcm)

F	0.817386	0.817386	0.817386
F	-0.817386	-0.817386	0.817386
F	0.817386	-0.817386	-0.817386
F	-0.817386	0.817386	-0.817386
B	0.000000	0.000000	0.000000

Cation **a_PPh4**: (PPh4_003a)

P	0.000913	0.002804	0.004404
C	0.983642	-1.324023	-0.749151
C	-1.009088	0.827433	-1.258878
C	-1.119053	-0.681677	1.257365
C	1.148150	1.188628	0.761980
C	-2.199560	0.215454	-1.690504
C	-2.964812	0.817786	-2.689713
C	-2.551781	2.028569	-3.256882
C	-1.371062	2.639515	-2.823819
C	-0.596320	2.043332	-1.825948
C	-0.903037	-1.961165	1.791361
C	-1.765891	-2.455292	2.772606
C	-2.840023	-1.680085	3.219894
C	-3.057140	-0.405157	2.685170
C	-2.202278	0.096780	1.703611
C	2.315856	1.550399	0.065916
C	3.191242	2.484547	0.620545
C	2.910391	3.056824	1.866328
C	1.751571	2.694898	2.559590
C	0.867609	1.761992	2.012058

C	0.628530	-1.860045	-1.996656
C	1.378588	-2.906656	-2.538412
C	2.477895	-3.417972	-1.842201
C	2.833511	-2.881685	-0.599546
C	2.092119	-1.834905	-0.050655
H	-2.530418	-0.720952	-1.250858
H	-3.883605	0.342049	-3.021303
H	-3.152622	2.497324	-4.031598
H	-1.052047	3.583210	-3.257419
H	0.314747	2.530081	-1.492650
H	-0.077543	-2.575241	1.445860
H	-1.600103	-3.448517	3.180655
H	-3.511196	-2.070374	3.980303
H	-3.893801	0.197672	3.027311
H	-2.382388	1.083451	1.285748
H	2.544750	1.104723	-0.897710
H	4.093160	2.760810	0.081331
H	3.597296	3.779998	2.297891
H	1.535107	3.132209	3.530352
H	-0.024768	1.483328	2.563160
H	-0.218216	-1.466154	-2.549699
H	1.104728	-3.316502	-3.506632
H	3.060274	-4.230433	-2.268546
H	3.689347	-3.274493	-0.057555
H	2.379234	-1.419226	0.910927

Ion Pair **3a**: (cat3a_008)

C	-2.241553	1.534490	0.715176
C	-1.677409	2.737413	1.131001
N	-1.466659	3.803915	0.337954
C	-1.844025	3.657216	-0.947686
C	-2.421209	2.505854	-1.467492
C	-2.650351	1.386611	-0.631779
N	-3.255170	0.292537	-1.232721
S	-3.511747	-1.088757	-0.511038
O	-3.947515	-2.090309	-1.504036
O	-2.532148	-1.523702	0.514563
C	-5.090025	-0.854590	0.520748
F	-6.120807	-0.453607	-0.250325
F	-5.448664	-2.008185	1.124330

F	-4.911385	0.075735	1.484788
H	-2.352935	0.728051	1.427523
H	-1.368090	2.845590	2.169362
H	-1.672123	4.513441	-1.598151
H	-2.706544	2.457755	-2.514764
P	2.200699	-0.208862	0.029089
C	1.106928	-1.071301	-1.129675
C	1.378413	0.001311	1.631406
C	2.620092	1.439523	-0.603926
C	0.355482	-0.356079	-2.074417
C	-0.511839	-1.040842	-2.926995
C	-0.628919	-2.430497	-2.842993
C	0.121178	-3.143733	-1.902090
C	0.990254	-2.470075	-1.044016
C	1.908108	0.915940	2.560226
C	1.294987	1.069777	3.803207
C	0.155041	0.320702	4.121093
C	-0.375110	-0.578634	3.192667
C	0.231544	-0.741723	1.944758
C	1.677971	2.472888	-0.463648
C	1.960179	3.741246	-0.970722
C	3.179168	3.984205	-1.612860
C	4.118790	2.956895	-1.747672
C	3.844792	1.682696	-1.244845
H	0.433686	0.723613	-2.146173
H	-1.105021	-0.484277	-3.646115
H	-1.314436	-2.956390	-3.501347
H	0.025542	-4.223600	-1.830132
H	1.569491	-3.029833	-0.315074
H	2.784578	1.508737	2.313791
H	1.701875	1.778941	4.518874
H	-0.325256	0.449486	5.087597
H	-1.273403	-1.143510	3.424821
H	-0.215273	-1.414721	1.222214
H	0.733475	2.295284	0.039469
H	1.226468	4.533797	-0.856311
H	3.398428	4.974363	-2.003836
H	5.068937	3.145506	-2.240073
H	4.584620	0.895481	-1.348213
C	3.707487	-1.205254	0.222102

C	4.306066	-1.769572	-0.918858
C	4.283176	-1.400367	1.486838
C	5.477870	-2.515717	-0.789567
H	3.860570	-1.635442	-1.900389
C	5.455792	-2.150531	1.606261
H	3.823379	-0.981985	2.376251
C	6.053876	-2.706133	0.471574
H	5.936657	-2.951247	-1.672966
H	5.895727	-2.303373	2.587966
H	6.964382	-3.291505	0.569057

Ion Pair **4a**: (cat4a_050_dcm)

C	-2.820692	-2.263320	-1.336581
C	-2.163162	-2.775532	-2.450489
N	-0.986036	-3.428482	-2.423873
C	-0.444048	-3.590593	-1.196469
C	-1.009888	-3.121588	-0.021523
C	-2.241504	-2.408458	-0.046011
N	-2.696432	-1.931248	1.155301
S	-4.040949	-1.044863	1.285658
O	-4.207149	-0.771132	2.737973
O	-5.227946	-1.610804	0.579204
C	-3.728124	0.562222	0.515849
C	-2.847892	1.450909	1.136765
C	-2.559486	2.686489	0.554829
C	-3.166306	3.035285	-0.662350
C	-4.062879	2.146549	-1.277201
C	-4.340150	0.914626	-0.692438
O	-2.944761	4.205441	-1.322809
C	-2.070255	5.171821	-0.731550
H	-3.773396	-1.766354	-1.468696
H	-2.613382	-2.647893	-3.434727
H	0.509843	-4.113090	-1.161774
H	-0.505905	-3.275842	0.928483
H	-2.385266	1.187164	2.081689
H	-1.869681	3.358327	1.052595
H	-4.528645	2.434346	-2.215545
H	-5.039339	0.232656	-1.164838
H	-2.040414	6.008465	-1.432369
H	-2.459967	5.516542	0.234474

H	-1.060067	4.766306	-0.602236
P	2.229498	0.120113	0.105863
C	2.659315	-1.513874	-0.558224
C	1.516125	-0.078631	1.763026
C	3.724626	1.140565	0.248622
C	1.062009	0.910085	-1.039309
C	2.361539	-0.091935	2.887780
C	1.822771	-0.324407	4.153924
C	0.449496	-0.550891	4.302815
C	-0.387914	-0.548763	3.183826
C	0.140844	-0.310609	1.913706
C	4.904767	0.777366	-0.418304
C	6.033003	1.596200	-0.323837
C	5.987584	2.771762	0.431201
C	4.811659	3.132958	1.098795
C	3.680353	2.321422	1.011360
C	-0.014356	0.162287	-1.549396
C	-0.910652	0.756749	-2.436486
C	-0.737430	2.088112	-2.825653
C	0.335059	2.830425	-2.324297
C	1.236473	2.247593	-1.430940
C	2.937311	-2.574458	0.318730
C	3.312291	-3.816662	-0.196067
C	3.407761	-4.004412	-1.578745
C	3.126857	-2.948676	-2.451932
C	2.752637	-1.702581	-1.947262
H	3.428680	0.077559	2.781421
H	2.477097	-0.330369	5.021586
H	0.033581	-0.734279	5.290173
H	-1.451820	-0.743795	3.280067
H	-0.527151	-0.322580	1.060633
H	4.951855	-0.135560	-1.003702
H	6.946341	1.311054	-0.838708
H	6.868137	3.404675	0.503414
H	4.774974	4.043582	1.690357
H	2.772932	2.602487	1.538854
H	-0.152359	-0.875260	-1.265540
H	-1.745527	0.175673	-2.813974
H	-1.441661	2.548300	-3.512537
H	0.472341	3.865137	-2.625598

H	2.069059	2.833487	-1.056305
H	2.851882	-2.441691	1.392901
H	3.517896	-4.638786	0.483838
H	3.689495	-4.975876	-1.976119
H	3.186820	-3.095857	-3.526559
H	2.526030	-0.890542	-2.631646

Additive **6**: (add6_034_dcm)

P	0.925127	-0.015768	-0.001218
C	2.712781	0.117555	-0.296647
C	0.074655	-0.515027	-1.522976
C	0.600821	-1.273472	1.267397
C	0.324194	1.607254	0.542516
C	0.113811	-1.863285	-1.920249
C	-0.518415	-2.254671	-3.100329
C	-1.191229	-1.309201	-3.882201
C	-1.235109	0.029384	-3.483262
C	-0.605223	0.431620	-2.303782
C	1.614492	-1.693328	2.143665
C	1.326074	-2.638685	3.130382
C	0.034451	-3.162765	3.246613
C	-0.973639	-2.744975	2.371471
C	-0.696400	-1.804588	1.378111
C	0.866519	2.771748	-0.030020
C	0.368968	4.021018	0.341892
C	-0.666256	4.113777	1.279079
C	-1.205907	2.955551	1.845385
C	-0.714322	1.700500	1.481108
C	3.301736	-0.480308	-1.421309
C	4.683480	-0.391709	-1.608890
C	5.477043	0.289153	-0.681063
C	4.889914	0.887863	0.439328
C	3.510856	0.806163	0.634430
H	0.624174	-2.604279	-1.311144
H	-0.492321	-3.297736	-3.403267
H	-1.691878	-1.619992	-4.795448
H	-1.773268	0.761661	-4.078545
H	-0.665102	1.468989	-1.991426
H	2.621796	-1.298644	2.060026
H	2.113030	-2.966772	3.804048

H	-0.185154	-3.898258	4.016134
H	-1.978779	-3.148788	2.456049
H	-1.485728	-1.493087	0.701282
H	1.669785	2.707735	-0.758321
H	0.789569	4.919539	-0.101329
H	-1.052963	5.088430	1.565040
H	-2.015979	3.021866	2.566088
H	-1.151006	0.810016	1.919788
H	2.694820	-1.004981	-2.152149
H	5.135855	-0.851296	-2.483366
H	6.551032	0.358268	-0.832641
H	5.503041	1.421740	1.160188
H	3.060943	1.281309	1.501612
F	-5.405884	-0.069253	-0.205499
F	-3.771158	0.095379	1.419165
F	-3.563623	1.280853	-0.553775
F	-3.328035	-1.016891	-0.554619
B	-4.018050	0.074513	0.024867

Cationic sandwich complex **a3a** (sw_a3a_new_033b_dcm_sp)

C	-1.351580	3.910209	1.661938
C	-1.911012	4.024461	2.925869
N	-2.020868	3.022274	3.812078
C	-1.531003	1.836435	3.411575
C	-0.951267	1.613539	2.177021
C	-0.848205	2.662130	1.240173
N	-0.253848	2.332250	0.032784
S	-0.233308	3.318328	-1.216683
O	0.161917	2.545263	-2.414513
O	-1.346874	4.283606	-1.363465
C	1.279108	4.452992	-0.967799
F	2.415755	3.735174	-0.906315
F	1.390990	5.323542	-1.987644
F	1.170620	5.156948	0.175477
H	-1.323710	4.780307	1.017361
H	-2.301004	4.992138	3.243281
H	-1.607886	1.013795	4.123961
H	-0.572566	0.629303	1.918888
P	-3.729977	-1.120693	-0.224405
C	-4.127034	0.633451	-0.043789

C	-5.256834	-2.068710	-0.419402
C	-2.884390	-1.735207	1.251912
C	-4.382389	1.165158	1.224525
C	-4.756844	2.498112	1.345529
C	-4.869192	3.299300	0.211970
C	-4.612161	2.769091	-1.049430
C	-4.242667	1.435834	-1.184988
C	-5.243146	-3.448401	-0.179272
C	-6.406059	-4.185720	-0.364753
C	-7.575817	-3.553816	-0.782556
C	-7.586338	-2.181873	-1.018778
C	-6.428036	-1.432870	-0.838911
C	-3.630495	-1.985733	2.411356
C	-2.982929	-2.412435	3.564152
C	-1.602254	-2.603382	3.561717
C	-0.866213	-2.368244	2.404384
C	-1.501546	-1.930974	1.246447
H	-4.283176	0.554112	2.115714
H	-4.946290	2.913116	2.330836
H	-5.149931	4.343946	0.312895
H	-4.691933	3.394690	-1.933102
H	-4.046929	1.027573	-2.172192
H	-4.333082	-3.942418	0.151057
H	-6.399029	-5.255514	-0.178215
H	-8.483857	-4.134008	-0.920675
H	-8.499307	-1.688927	-1.339198
H	-6.441882	-0.361561	-1.017236
H	-4.709338	-1.855302	2.414910
H	-3.560393	-2.602707	4.463929
H	-1.100992	-2.943112	4.463769
H	0.207504	-2.528428	2.394237
H	-0.918984	-1.749300	0.348663
C	-2.682296	-1.332351	-1.681471
C	-1.602053	-0.461893	-1.877853
C	-2.947128	-2.352888	-2.599722
C	-0.788699	-0.625209	-2.992741
H	-1.394873	0.344219	-1.176772
C	-2.122464	-2.506726	-3.709421
H	-3.793377	-3.018756	-2.460752
C	-1.045411	-1.646369	-3.905377

H	0.038629	0.060535	-3.146116
H	-2.330186	-3.296684	-4.425086
H	-0.407929	-1.766068	-4.777212
P	3.847558	-1.068604	0.073384
C	3.977903	-0.247632	-1.530544
C	3.275872	0.125729	1.303949
C	2.706340	-2.472189	-0.033960
C	5.463115	-1.708991	0.570466
C	2.571837	-0.317044	2.428826
C	2.210233	0.594072	3.413159
C	2.542119	1.940255	3.277406
C	3.242967	2.378909	2.157486
C	3.617052	1.475601	1.168281
C	1.757789	-2.533418	-1.058568
C	0.877309	-3.608785	-1.116059
C	0.941914	-4.619341	-0.160978
C	1.890368	-4.559581	0.857850
C	2.776097	-3.490788	0.926012
C	5.928982	-2.907019	0.014582
C	7.189240	-3.377410	0.362334
C	7.979637	-2.662476	1.260138
C	7.512830	-1.472940	1.812436
C	6.253712	-0.990153	1.470894
C	5.096930	-0.470790	-2.338958
C	5.172722	0.136055	-3.587761
C	4.142125	0.962516	-4.026509
C	3.031517	1.190408	-3.216819
C	2.942311	0.589789	-1.966460
H	2.302752	-1.362829	2.539359
H	1.656553	0.252215	4.282698
H	2.246006	2.650810	4.044083
H	3.499585	3.428528	2.048875
H	4.170459	1.822442	0.300914
H	1.705753	-1.756974	-1.815309
H	0.143908	-3.655952	-1.916053
H	0.254432	-5.459008	-0.212540
H	1.945641	-5.349009	1.601661
H	3.520367	-3.456571	1.716861
H	5.314453	-3.468727	-0.683732
H	7.552580	-4.306288	-0.067167

H	8.962467	-3.036780	1.532444
H	8.126692	-0.918412	2.515816
H	5.892212	-0.064303	1.908557
H	5.908991	-1.105677	-1.998695
H	6.043591	-0.034078	-4.213760
H	4.207082	1.438317	-5.001091
H	2.229608	1.843471	-3.545430
H	2.075589	0.784359	-1.339098

Cationic sandwich complex **a4a** (sw_a4a_018_dcm_sp)

C	-0.201815	1.972046	2.209283
C	-0.492438	1.370883	3.423647
N	-0.439820	0.050440	3.667035
C	-0.070891	-0.711987	2.617398
C	0.225310	-0.221186	1.362766
C	0.172974	1.172516	1.100088
N	0.473311	1.563917	-0.173614
S	0.466107	3.135680	-0.595297
O	0.861468	3.157572	-2.027917
O	1.239924	4.023475	0.317443
C	-1.241538	3.723716	-0.545641
C	-1.633612	4.702852	0.356776
C	-2.952932	5.154047	0.378416
C	-3.880234	4.609639	-0.514348
C	-3.477807	3.626521	-1.431950
C	-2.166343	3.190216	-1.448377
O	-5.187933	4.963097	-0.573676
C	-5.650854	5.971886	0.334107
H	-0.250478	3.052166	2.134538
H	-0.784597	2.001555	4.264954
H	-0.018108	-1.786702	2.796639
H	0.500022	-0.901157	0.560842
H	-0.908350	5.128751	1.043313
H	-3.236481	5.922397	1.088431
H	-4.208894	3.221137	-2.125748
H	-1.856662	2.439919	-2.169441
H	-5.143819	6.927859	0.153808
H	-6.717718	6.082906	0.132544
H	-5.505878	5.666635	1.377367
P	-3.813089	-1.787192	-0.156177

C	-2.714515	-1.578534	-1.578962
C	-5.260120	-2.728217	-0.696101
C	-2.946720	-2.697134	1.143759
C	-4.344324	-0.193519	0.516807
C	-5.761676	-3.765060	0.094722
C	-6.895927	-4.455734	-0.319960
C	-7.525475	-4.115315	-1.513590
C	-7.023210	-3.081499	-2.301660
C	-5.890204	-2.385567	-1.899743
C	-1.899338	-3.558436	0.803114
C	-1.277983	-4.307254	1.796223
C	-1.694242	-4.194963	3.120585
C	-2.735900	-3.334105	3.457858
C	-3.368055	-2.583263	2.473570
C	-3.472670	0.534352	1.337716
C	-3.888016	1.750371	1.867001
C	-5.160841	2.240276	1.584946
C	-6.023843	1.517636	0.766602
C	-5.621949	0.298804	0.231354
C	-1.863469	-0.472123	-1.642319
C	-0.990195	-0.336355	-2.716215
C	-0.968956	-1.296725	-3.723803
C	-1.807404	-2.407464	-3.652390
C	-2.680038	-2.556917	-2.581006
H	-5.271583	-4.039110	1.024150
H	-7.283846	-5.264673	0.291859
H	-8.409219	-4.659692	-1.834271
H	-7.512066	-2.817603	-3.234738
H	-5.498440	-1.584453	-2.520665
H	-1.567192	-3.643017	-0.227652
H	-0.462885	-4.975531	1.534646
H	-1.201556	-4.776989	3.894431
H	-3.057666	-3.242555	4.490940
H	-4.182403	-1.915854	2.740732
H	-2.480791	0.155003	1.568401
H	-3.211678	2.313191	2.502868
H	-5.481400	3.189008	2.005857
H	-7.017478	1.897158	0.547575
H	-6.308861	-0.265768	-0.390828
H	-1.868580	0.284827	-0.864498

H	-0.329472	0.525368	-2.754431
H	-0.292902	-1.182620	-4.566616
H	-1.784752	-3.161595	-4.433648
H	-3.328668	-3.426299	-2.529741
P	4.759936	-0.412017	-0.145842
C	4.155388	-0.028817	1.514347
C	6.300484	-1.342953	0.031854
C	3.565955	-1.418783	-1.061137
C	5.059158	1.104662	-1.077497
C	6.588406	-2.403222	-0.831645
C	7.796926	-3.080396	-0.705410
C	8.711958	-2.702574	0.273418
C	8.422920	-1.644555	1.133035
C	7.217982	-0.962129	1.018912
C	2.724776	-2.307473	-0.385720
C	1.883411	-3.142565	-1.112544
C	1.881822	-3.096871	-2.503855
C	2.718037	-2.208377	-3.175613
C	3.561500	-1.366597	-2.460284
C	3.964473	1.837741	-1.554108
C	4.186522	3.022665	-2.244819
C	5.487423	3.469612	-2.468229
C	6.572954	2.734564	-1.999608
C	6.365149	1.549561	-1.301330
C	3.670825	1.245661	1.817755
C	3.194299	1.510247	3.097783
C	3.194065	0.511291	4.066334
C	3.685801	-0.757681	3.764486
C	4.174667	-1.032185	2.493776
H	5.876074	-2.704832	-1.593986
H	8.019828	-3.908018	-1.372188
H	9.653287	-3.236209	0.369841
H	9.135571	-1.350563	1.897928
H	6.994743	-0.139779	1.693347
H	2.713770	-2.347742	0.698917
H	1.227966	-3.830080	-0.586521
H	1.224338	-3.753387	-3.066584
H	2.715382	-2.167824	-4.260834
H	4.214861	-0.679060	-2.989472
H	2.944429	1.499094	-1.385329

H	3.332010	3.589503	-2.600548
H	5.655162	4.395108	-3.012227
H	7.586882	3.079913	-2.178687
H	7.214890	0.977375	-0.941871
H	3.665981	2.032447	1.070350
H	2.821157	2.502199	3.333157
H	2.814654	0.722214	5.062145
H	3.695394	-1.535305	4.522517
H	4.575594	-2.017076	2.269271

Anionic sandwich complex **3a3** (asw_3a3_058b_dcm_sp)

C	-3.758678	-1.886808	0.329481
C	-2.828696	-2.851906	0.684583
N	-2.402311	-3.085177	1.936095
C	-2.940616	-2.301544	2.887714
C	-3.872406	-1.309527	2.645111
C	-4.323879	-1.065515	1.328908
N	-5.268942	-0.066276	1.190499
S	-5.800614	0.439093	-0.221377
O	-6.580783	1.676907	-0.021471
O	-4.866852	0.398486	-1.375135
C	-7.159691	-0.796348	-0.736297
F	-8.114935	-0.873630	0.207659
F	-7.741589	-0.410484	-1.888184
F	-6.655108	-2.032440	-0.918709
H	-4.030894	-1.774930	-0.712584
H	-2.395960	-3.478322	-0.096951
H	-2.602015	-2.479367	3.908949
H	-4.268294	-0.715604	3.464350
P	0.155210	1.165050	-0.101526
C	-1.290466	2.049061	0.528874
C	-0.329505	-0.036689	-1.357511
C	0.958032	0.256763	1.237124
C	-1.784890	1.799045	1.811178
C	-2.895058	2.503892	2.265718
C	-3.508146	3.448283	1.448583
C	-3.011567	3.698215	0.170464
C	-1.900667	3.005882	-0.293257
C	0.630354	-0.959790	-1.793792
C	0.285301	-1.898131	-2.757141

C	-1.009230	-1.923576	-3.275590
C	-1.961278	-1.010792	-2.831859
C	-1.627788	-0.061867	-1.869670
C	0.351847	-0.923852	1.683642
C	0.927832	-1.635683	2.727744
C	2.109931	-1.184246	3.312604
C	2.714920	-0.015227	2.859295
C	2.139437	0.713966	1.823367
H	-1.309395	1.066950	2.456519
H	-3.280142	2.310605	3.262364
H	-4.377540	3.991985	1.807145
H	-3.490197	4.434753	-0.468082
H	-1.510448	3.211314	-1.286969
H	1.640848	-0.947618	-1.394736
H	1.031338	-2.609701	-3.097985
H	-1.277061	-2.663922	-4.024561
H	-2.974333	-1.038343	-3.222246
H	-2.387612	0.625200	-1.511864
H	-0.560688	-1.287829	1.219009
H	0.457162	-2.551745	3.072687
H	2.565913	-1.751848	4.119082
H	3.644217	0.330802	3.302952
H	2.620954	1.620010	1.470745
C	1.273793	2.402835	-0.802634
C	1.525847	3.568130	-0.066183
C	1.870941	2.212854	-2.050852
C	2.385268	4.531277	-0.579860
H	1.051495	3.725446	0.899216
C	2.724642	3.187923	-2.557470
H	1.671292	1.318192	-2.631793
C	2.983360	4.342271	-1.824668
H	2.583799	5.432941	-0.007983
H	3.187535	3.040184	-3.528593
H	3.652179	5.099377	-2.224458
C	6.561485	-0.055025	0.602552
C	7.313024	0.783499	1.412248
N	7.031827	2.072956	1.657502
C	5.933398	2.552788	1.047920
C	5.115274	1.807204	0.220401
C	5.404158	0.448255	-0.029347

N	4.503138	-0.219199	-0.840123
S	4.753107	-1.693860	-1.383824
O	3.766544	-1.975950	-2.448295
O	6.152738	-2.123195	-1.612372
C	4.167701	-2.869084	-0.001677
F	2.869788	-2.660771	0.284511
F	4.310368	-4.153789	-0.379032
F	4.876844	-2.686137	1.129867
H	6.879204	-1.081692	0.466999
H	8.203472	0.384489	1.899359
H	5.693506	3.600468	1.233711
H	4.242272	2.258688	-0.241025

Anionic sandwich complex **4a4** (asw_4a4_005a_dcm_sp)

C	-2.555094	4.170689	-0.622092
C	-3.682213	3.966701	-1.401677
N	-4.803718	3.346988	-0.996285
C	-4.787349	2.912194	0.280609
C	-3.718947	3.052590	1.141733
C	-2.527933	3.699983	0.717247
N	-1.514720	3.776625	1.629466
S	-0.087383	4.454644	1.234093
O	0.733430	4.411641	2.470370
O	-0.187027	5.776856	0.554539
C	0.712872	3.338660	0.063078
C	1.032022	2.053169	0.490299
C	1.651000	1.158123	-0.372713
C	1.959773	1.562860	-1.677785
C	1.650715	2.861681	-2.100893
C	1.024200	3.744669	-1.233772
O	2.551185	0.758923	-2.594652
C	2.845688	-0.586521	-2.186707
H	-1.712326	4.703584	-1.046811
H	-3.681128	4.330170	-2.430685
H	-5.690880	2.411989	0.630026
H	-3.780198	2.665552	2.156102
H	0.806146	1.746412	1.507252
H	1.895149	0.161114	-0.024174
H	1.903895	3.161458	-3.113893
H	0.785517	4.753344	-1.557277

H	3.292435	-1.072081	-3.056728
H	3.560252	-0.595998	-1.356812
H	1.930983	-1.116192	-1.896812
P	-3.187799	-1.555379	-0.037089
C	-4.831380	-0.801261	-0.005970
C	-2.388514	-1.284356	1.564248
C	-3.371189	-3.333860	-0.311657
C	-2.228201	-0.815262	-1.379617
C	-2.529952	-2.234899	2.581364
C	-1.985397	-1.982825	3.835741
C	-1.313850	-0.787684	4.079652
C	-1.180460	0.159664	3.067674
C	-1.710456	-0.084818	1.805641
C	-4.589027	-3.851360	-0.762331
C	-4.706780	-5.217580	-0.996498
C	-3.619710	-6.060703	-0.782836
C	-2.408056	-5.541241	-0.331362
C	-2.275467	-4.178440	-0.091633
C	-2.303518	0.566479	-1.600165
C	-1.560350	1.135993	-2.626812
C	-0.744542	0.340444	-3.427963
C	-0.670926	-1.031723	-3.206368
C	-1.412256	-1.615855	-2.185014
C	-5.508018	-0.680536	1.211789
C	-6.810570	-0.195090	1.226556
C	-7.433471	0.173162	0.036374
C	-6.755274	0.057828	-1.174554
C	-5.454509	-0.431890	-1.203066
H	-3.063698	-3.163092	2.401025
H	-2.088853	-2.723663	4.623073
H	-0.892217	-0.593376	5.061833
H	-0.660647	1.094363	3.256783
H	-1.591902	0.659682	1.023685
H	-5.442228	-3.199654	-0.924354
H	-5.653116	-5.621696	-1.343640
H	-3.717856	-7.127329	-0.965141
H	-1.559603	-6.198093	-0.161444
H	-1.324720	-3.773024	0.256411
H	-2.939675	1.194584	-0.981713
H	-1.616328	2.207237	-2.793098

H	-0.163763	0.792655	-4.226623
H	-0.034759	-1.654250	-3.828724
H	-1.355496	-2.687910	-2.027604
H	-5.023275	-0.958341	2.143322
H	-7.335264	-0.096479	2.172236
H	-8.449145	0.558531	0.053214
H	-7.236934	0.352411	-2.102117
H	-4.932723	-0.527267	-2.151026
C	3.055832	-2.386616	1.161536
C	1.673166	-2.339227	1.219706
N	0.843247	-2.964270	0.368062
C	1.445369	-3.696196	-0.591478
C	2.810896	-3.808248	-0.747329
C	3.695901	-3.127118	0.131711
N	5.030310	-3.250864	-0.127091
S	6.131636	-2.416452	0.739685
O	7.455252	-2.819362	0.205115
O	5.962282	-2.502842	2.217548
C	5.906054	-0.681268	0.297103
C	5.354946	0.219768	1.198347
C	5.098638	1.535020	0.813831
C	5.400970	1.941780	-0.488535
C	5.990791	1.037936	-1.386325
C	6.236798	-0.265754	-0.996707
O	5.162820	3.184161	-0.976694
C	4.515617	4.126200	-0.109566
H	3.630703	-1.864007	1.915836
H	1.198398	-1.752190	2.008477
H	0.781564	-4.215995	-1.285466
H	3.223429	-4.406159	-1.556018
H	5.122249	-0.097547	2.209937
H	4.659061	2.218689	1.530940
H	6.233434	1.374617	-2.390207
H	6.678268	-0.967880	-1.698666
H	5.161037	4.386731	0.738956
H	4.338017	5.015077	-0.717647
H	3.558734	3.737713	0.257716

References

(1) Harris, R. K.; Becker, E. D.; Cabral De Menezes, S. M.; Goodfellow, R.; Granger, P. NMR Nomenclature. Nuclear Spin Properties and Conventions for Chemical Shifts. *Pure Appl. Chem.* **2001**, *73* (11), 1795–1818. https://doi.org/10.1351/pac200173111795.

(2) Mayer, R. J.; Ofial, A. R.; Mayr, H.; Legault, C. Y. Lewis Acidity Scale of Diaryliodonium Ions toward Oxygen, Nitrogen, and Halogen Lewis Bases. *J. Am. Chem. Soc.* **2020**, *142* (11), 5221–5233. https://doi.org/10.1021/jacs.9b12998.

(3) Wu, C.; Koch, W. F.; Pratt, K. W. Proposed New Electrolytic Conductivity Primary Standards for KCl Solutions. *J. Res. Natl. Inst. Stand. Technol* **1991**, *96* (2), 191–201. https://doi.org/10.6028/jres.096.008.

(4) Reichardt, C.; Welton, T. *Solvents and Solvent Effects in Organic Chemistry: Fourth Edition*; 2003. https://doi.org/10.1002/9783527632220.

(5) Hoops, S.; Gauges, R.; Lee, C.; Pahle, J.; Simus, N.; Singhal, M.; Xu, L.; Mendes, P.; Kummer, U. COPASI - A COmplex PAthway SImulator. *Bioinformatics* **2006**, *22* (24), 3067–3074. https://doi.org/10.1093/bioinformatics/btl485.

(6) Zott, F. *fabianzott/steadystate_analysis*. Github.com. https://github.com/fabianzott/steadystate_analysis.

(7) Tsierkezos, N. G.; Philippopoulos, A. I. Studies of Ion Solvation and Ion Association of N-Tetrabutylammonium Hexafluorophosphate and n-Tetrabutylammonium Tetraphenylborate in Various Solvents. *Fluid Phase Equilib.* **2009**, *277* (1), 20–28. https://doi.org/10.1016/j.fluid.2008.11.004.

(8) Fuoss, R. M.; Kraus, C. A. Properties of Electrolytic Solutions. IV. The Conductance Minimum and the Formation of Triple Ions Due to the Action of Coulomb Forces. *J. Am. Chem. Soc.* **1933**, *55*, 2387. https://doi.org/10.1093/nq/s6-V.129.467d.

(9) Hojo, M.; Miyauchi, Y.; Tanio, A.; Imai, Y. Specific Interactions between Anions and Cations in Protophobic Aprotic Solvents. Triple-Ion and Higher Aggregate Formation from Lithium and Tributylammonium Thiocyanates. *J. Chem. Soc. Faraday Trans.* **1991**, *87* (24), 3847–3852. https://doi.org/10.1039/FT9918703847.

(10) Boruń, A.; Bald, A. Triple Ion Formation in Solutions of [Emim][BF4] and [Bmim][BF4] in Dichloromethane at Various Temperatures. A New Method of Analysis of Conductivity Data. *Int. J. Electrochem. Sci.* **2016**, *11* (9), 7714–7725. https://doi.org/10.20964/2016.09.33.

(11) S. Boileau; P. Hemery. Conductance of Some Tetraphenylboron and Fluorenyl Salts in Tetrahydropyran. *Electrochim. Acta* **1976**, *21*, 647–655.

(12) Miyauchi, Y.; Hojo, M.; Moriyama, H.; Imai, Y. Conductometric Identification of Triple-Ion and Quadrupole Formation by the Coordination Forces from Lithium Trifluoroacetate and Lithium Pentafluoropropionate in Protophobic Aprotic Solvents. *J. Chem. Soc. Faraday Trans.* **1992**, *88* (21), 3175–3182. https://doi.org/10.1039/FT9928803175.

(13) Hojo, M.; Moriyama, H. Conductance in Isodielectric Mixed Solvents Containing Triple Ions. *J. Solution Chem.* **1996**, *25* (7), 681–694. https://doi.org/10.1007/BF00972682.

(14) Brown, A. M.; Fuoss, R. M. Conductance of Tetrabutylammonium Tetraphenylboride in Nitriles. *J. Am. Chem. Soc.* **1960**, *64*, 1341–1342.

(15) Fuoss, R. M.; Hirsch, E. Single Ion Conductances in Non-Aqueous Solvents. *J. Am. Chem. Soc.* **1960**, *82* (5), 1013–1017.

(16) Jerschow, A.; Müller, N. Diffusion-Separated Nuclear Magnetic Resonance Spectroscopy of Polymer Mixtures. *Macromolecules* **1998**, *31* (19), 6573–6578. https://doi.org/10.1021/ma9801772.

(17) Stejskal, E. O.; Tanner, J. E. Spin Diffusion Measurements: Spin Echoes in the Presence of a Time-Dependent Field Gradient. *J. Chem. Phys.* **1965**, *42* (1), 288–292. https://doi.org/10.1063/1.1695690.

(18) Macchioni, A.; Ciancaleoni, G.; Zuccaccia, C.; Zuccaccia, D. Determining Accurate Molecular Sizes in Solution through NMR Diffusion Spectroscopy. *Chem. Soc. Rev.* **2008**, *37* (3), 479–489. https://doi.org/10.1039/b615067p.

(19) Zuccaccia, D.; Macchioni, A. An Accurate Methodology to Identify the Level of Aggregation in Solution by PGSE NMR Measurements: The Case of Half-Sandwich Diamino Ruthenium(II) Salts. *Organometallics* **2005**, *24* (14), 3476–3486. https://doi.org/10.1021/om050145k.

(20) Ben-Amotz, D.; Willis, K. G. Molecular Hard-Sphere Volume. *J. Phys. Chem.* **1993**, *97*, 7736–7742.

(21) Chen, H. C.; Chen, S. H. Diffusion of Crown Ethers in Alcohols. *J. Phys. Chem.* **1984**, *88* (21), 5118–5121. https://doi.org/10.1021/j150665a063.

(22) Mayr, H.; Bug, T.; Gotta, M. F.; Hering, N.; Irrgang, B.; Janker, B.; Kempf, B.; Loos, R.; Ofial, A. R.; Remennikov, G.; Schimmel, H. Reference Scales for the Characterization of Cationic Electrophiles and Neutral Nucleophiles. *J. Am. Chem. Soc.* **2001**, *123* (39), 9500–9512. https://doi.org/10.1021/ja010890y.

(23) Brotzel, F.; Kempf, B.; Singer, T.; Zipse, H.; Mayr, H. Nucleophilicities and Carbon Basicities of Pyridines. *Chem. Eur. J.* **2007**, *13* (1), 336–345. https://doi.org/10.1002/chem.200600941.

(24) Rycke, N. De; Berionni, G.; Couty, F.; Mayr, H.; Goumont, R.; David, O. R. P. Synthesis and Reactivity of Highly Nucleophilic Pyridines. *Org. Lett.* **2011**, *13* (3), 530–533. https://doi.org/10.1021/ol1029589.

(25) Chen, G.; Guo, S.; Feng, H.; Qian, Z. Anion-Regulated Transient and Persistent Phosphorescence and Size-Dependent Ultralong Afterglow of Organic Ionic Crystals. *J. Mater. Chem. C* **2019**, *7* (46), 14535–14542. https://doi.org/10.1039/c9tc04951g.

(26) Sinnaeve, D. The Stejskal-Tanner Equation Generalized for Any Gradient Shape-An Overview of Most Pulse Sequences Measuring Free Diffusion. *Concepts Magn. Reson. Part A Bridg. Educ. Res.* **2012**, *40 A* (2), 39–65. https://doi.org/10.1002/cmr.a.21223.

(27) Becke, A. D. A New Mixing of Hartree-Fock and Local Density-Functional Theories. *J. Chem. Phys.* **1993**, *98* (2), 1372–1377. https://doi.org/10.1063/1.464304.

(28) Grimme, S. Semiempirical Hybrid Density Functional with Perturbative Second-Order Correlation. *J. Phys. Chem.* **2006**, *124* (3), 034108. https://doi.org/034108.

(29) Lee, C.; Yang, W.; Parr, R. G. Development of the Colic-Salvetti Correlation-Energy Formula into a Functional of the Electron Density. *Phy. Rev.* **1988**, *37* (2), 785–789.

(30) Spitznagel, G. W.; Clark, T.; Chandrasekhar, J.; Schleyer, P. V. R. Stabilization of Methyl Anions by First-row Substituents. The Superiority of Diffuse Function-augmented Basis Sets for Anion Calculations. *J. Comput Chem.* **1982**, *3* (3), 363–371. https://doi.org/10.1002/jcc.540030311.

(31) Marenich, A. V.; Cramer, C. J.; Truhlar, D. G. Universal Solvation Model Based on Solute Electron Density and on a Continuum Model of the Solvent Defined by the Bulk Dielectric Constant and Atomic Surface Tensions. *J. Phys. Chem. B* **2009**, *113* (18), 6378–6396. https://doi.org/10.1021/jp810292n..

(32) Marin-Luna, M.; Patschinski, P.; Zipse, H. Substituent Effects in the Silylation of Secondary Alcohols: A Mechanistic Study. *Chem. Eur. J.* **2018**, *24* (56), 15052–15058. https://doi.org/10.1002/chem.201803014.

(33) Marin-Luna, M.; Pölloth, B.; Zott, F.; Zipse, H. Size-Dependent Rate Acceleration in the Silylation of Secondary Alcohols: The Bigger the Faster. *Chem. Sci.* **2018**, *9* (31), 6509–6515. https://doi.org/10.1039/c8sc01889h.

(34) Pölloth, B.; Sibi, M. P.; Zipse, H. The Size-Accelerated Kinetic Resolution of Secondary Alcohols. *Ange. Chem. Int. Ed.* **2021**, *60* (2), 774–778. https://doi.org/10.1002/anie.202011687.

(35) Mayr, S.; Marin-Luna, M.; Zipse, H. Size-Driven Inversion of Selectivity in Esterification Reactions: Secondary Beat Primary Alcohols. *J. Org. Chem.* **2021**, *86* (4), 3456–3489. https://doi.org/10.1021/acs.joc.0c02848.

(36) Grimme, S. Supramolecular Binding Thermodynamics by Dispersion-Corrected Density Functional Theory. *Chem. Eur. J.* **2012**, *18* (32), 9955–9964. https://doi.org/10.1002/chem.201200497.

(37) Luchini, G.; Alegre-Requena, J. V; Funes-Ardoiz, I.; Paton, R. S. GoodVibes: Automated Thermochemistry for Heterogeneous Computational Chemistry Data. *F1000Res.* **2020**, *9* (291), 1–14.

(38) Gaussian 16; Revision A.03 Frisch, M. J.; Trucks, G. W.; Schlegel, H. B.; Scuseria, G. E.; Robb, M. A.; Cheeseman, J. R.; Scalmani, G.; Barone, V.; Petersson, G. A.; Nakatsuji, H.; Li, X.; Caricato, M.; Marenich, A. V.; Bloino, J.; Janesko, B. G.; Gomperts, R.; Mennucci, B.; Hratchian, H. P.; Ortiz, J. V.; Izmaylov, A. F.; Sonnenberg, J. L.; Williams-Young, D.; Ding, F., L. F., Egidi, F.; Goings, J.; Peng, B.; Petrone, A.; Henderson, T.; Ranasinghe, D.; Zakrzewski, V. G.; Gao, J.; Rega, N.; Zheng, G.; Liang, W.; Hada, M.; Ehara, M.; Toyota, K.; Fukuda, R.; Hasegawa, J.; Ishida, M.; Nakajima, T.; Honda, Y.; Kitao, O.; Nakai, H.; Vreven, T.; Throssell, K.; Montgomery Jr., J. A.; Peralta, J. E.; Ogliaro, F.; Bearpark, M. J.; Heyd, J. J.; Brothers, E. N.; Kudin, K. N.; Staroverov, V. N.; Keith, T. A.; Kobayashi, R.; Normand, J.; Raghavachari, K.; Rendell, A. P.; Burant, J. C.; Iyengar, S. S.; Tomasi, J.; Cossi, M.; Millam, J. M.; Klene, M.; Adamo, C.; Cammi, R.; Ochterski, J. W.; Martin, R. L.; Morokuma, K.; Farkas, O.; Foresman, J. B.; Fox, D. J. Gaussian 16, Revision A.03. **2016**, p Gaussian, Inc, Wallingford CT.

(39) Gaussian 16; Revision B.01; Frisch, M. J.; Trucks, G. W.; Schlegel, H. B.; Scuseria, G. E.; Robb, M. A.; Cheeseman, J. R.; Scalmani, G.; Barone, V.; Petersson, G. A.; Nakatsuji, H. ; Li, X.; Caricato, M.; Marenich, A. V.; Bloino, J.; Janesko, B. G.; Gomperts, R.; Mennucci, B.; Hratchian, H. P.; Ortiz, J. V.; Izmaylov, A. F.; Sonnenberg, J. L.; Williams-Young, D.; Ding, F.; Lipparini, F.; Egidi, F. ; Goings, J.; Peng, B.; Petrone, A.; Henderson, T.; Ranasinghe, D.; Zakrzewski, V. G.; Gao, J.; Rega, N.; Zheng, G.; Liang, W.; Hada, M.; Ehara, M.; Toyota, K.; Fukuda, R.; Hasegawa, J.; Ishida, M.; Nakajima, T.; Honda, Y.; Kitao, O.; Nakai, H.; Vreven, T.; Throssell, K.; Montgomery, J. A., Jr.; Peralta, J. E.; Ogliaro, F.; Bearpark, M. J.; Heyd, J. J.; Brothers, E. N.; Kudin, K. N.; Staroverov, V. N.; Keith, T. A.; Kobayashi, R.; Normand, J.; Raghavachari, K.; Rendell, A. P.; Burant, J. C.; Iyengar, S. S.; Tomasi, J.; Cossi, M.; Millam, J. M.; Klene, M.; Adamo, C.; Cammi, R.; Ochterski, J. W.; Martin, R. L.; Morokuma, K.; Farkas, O.; Foresman, J. B.; Fox, D. J. Gaussian 16, Revision B.01. **2016**, p Gaussian, Inc, Wallingford CT.

(40) Maestro, rev. 12.2.012; Schrödinger, New York, **2019**.

(41) Saunders, M. Stochastic Search for Isomers on a Quantum Mechanical Surface. *J. Comput. Chem.* **2004**, *25* (5), 621–626. https://doi.org/10.1002/jcc.10407.

(42) Šakić, D.; Hanževački, M.; Smith, D. M.; Vrček, V. A Computational Study of the Chlorination and Hydroxylation of Amines by Hypochlorous Acid. *Org. Biomol. Chem.* **2015**, *13* (48), 11740–11752. https://doi.org/10.1039/c5ob01823d.

(43) Helberg, J.; Ampßler, T.; Zipse, H. Pyridinyl Amide Ion Pairs as Lewis Base Organocatalysts. *J. Org. Chem.* **2020**, *85*, 5390–5402.

(44) Grimme, S.; Hansen, A.; Ehlert, S.; Mewes, J. M. R2SCAN-3c: A “Swiss Army Knife” Composite Electronic-Structure Method. *J. Chem. Phys.* **2021**, *154* (6). https://doi.org/10.1063/5.0040021.

(45) Neese, F. Software Update: The ORCA Program System—Version 5.0. *WIREs Comput. Mol Sci*. John Wiley and Sons Inc September 1, 2022. https://doi.org/10.1002/wcms.1606.

(46) Gaussian 16; Revision C.01; Frisch, M. J.; Trucks, G. W.; Schlegel, H. B.; Scuseria, G. E.; Robb, M. A.; Cheeseman, J. R.; Scalmani, G.; Barone, V.; Petersson, G. A.; Nakatsuji, H.; Li, X.; Caricato, M.; Marenich, A. V.; Bloino, J.; Janesko, B. G.; Gomperts, R.; Mennucci, B.; Hratchian, H. P.; Ortiz, J. V.; Izmaylov, A. F.; Sonnenberg, J. L.; Williams-Young, D.; Ding, F.; Lipparini, F.; Egidi, F.; Goings, J.; Peng, B.; Petrone, A.; Henderson, T.; Ranasinghe, D.; Zakrzewski, V. G.; Gao, J.; Rega, N.; Zheng, G.; Liang, W.; Hada, M.; Ehara, M.; Toyota, K.; Fukuda, R.; Hasegawa, J.; Ishida, M.; Nakajima, T.; Honda, Y.; Kitao, O.; Nakai, H.; Vreven, T.; Throssell, K.; Montgomery, J. A. , Jr.; Peralta, J. E.; Ogliaro, F.; Bearpark, M. J.; Heyd, J. J.; Brothers, E. N.; Kudin, K. N.; Staroverov, V. N.; Keith, T. A.; Kobayashi, R.; Normand, J.; Raghavachari, K.; Rendell, A. P.; Burant, J. C.; Iyengar, S. S.; Tomasi, J.; Cossi, M.; Millam, J. M.; Klene, M.; Adamo, C.; Cammi, R.; Ochterski, J. W.; Martin, R. L.; Morokuma, K.; Farkas, O.; Foresman, J. B.; Fox, D. J. Gaussian 16, Revision C.01. Gaussian, Inc.: Wallingford CT **2016**, p Gaussian, Inc, Wallingford CT.
